# Zebrafish models of *alx*-linked frontonasal dysplasia reveal a role for Alx1 and Alx3 in the anterior segment and vasculature of the developing eye

**DOI:** 10.1242/bio.059189

**Published:** 2022-05-26

**Authors:** Baul Yoon, Pan Yeung, Nicholas Santistevan, Lauren E. Bluhm, Kenta Kawasaki, Janina Kueper, Richard Dubielzig, Jennifer VanOudenhove, Justin Cotney, Eric C. Liao, Yevgenya Grinblat

**Affiliations:** 1 Departments of Integrative Biology and Neuroscience, University of Wisconsin, Madison, WI 53706, USA; 2 Genetics Ph.D. Training Program, University of Wisconsin, Madison, WI 53706, USA; 3 Center for Regenerative Medicine, Department of Surgery, Massachusetts General Hospital, Harvard Medical School, and Shriners Hospital for Children, Boston, 02114, USA; 4 Institute of Human Genetics, University of Bonn, Venusberg-Campus 1, 53127 Bonn, Germany; 5 Comparative Ocular Pathology Laboratory of Wisconsin (COPLOW), University of Wisconsin, Madison, WI 53706, USA; 6 University of Connecticut School of Medicine, Department of Genetics and Genome Sciences, Farmington, CT 06030, USA

**Keywords:** Neural crest, *ALX*, Frontonasal dysplasia, Ocular anterior segment, Fetal alcohol syndrome

## Abstract

The cellular and genetic mechanisms that coordinate formation of facial sensory structures with surrounding skeletal and soft tissue elements remain poorly understood. Alx1, a homeobox transcription factor, is a key regulator of midfacial morphogenesis. *ALX1* mutations in humans are linked to severe congenital anomalies of the facial skeleton (frontonasal dysplasia, FND) with malformation or absence of eyes and orbital contents (micro- and anophthalmia). Zebrafish with loss-of-function *alx1* mutations develop with craniofacial and ocular defects of variable penetrance, likely due to compensatory upregulation in expression of a paralogous gene, *alx3*. Here we show that zebrafish *alx1;alx3* mutants develop with highly penetrant cranial and ocular defects that resemble human *ALX1*-linked FND. *alx1* and *alx3* are expressed in anterior cranial neural crest (aCNC), which gives rise to the anterior neurocranium (ANC), anterior segment structures of the eye and vascular pericytes. Consistent with a functional requirement for *alx* genes in aCNC, *alx1; alx3* mutants develop with nearly absent ANC and grossly aberrant hyaloid vasculature and ocular anterior segment, but normal retina. *In vivo* lineage labeling identified a requirement for *alx1* and *alx3* during aCNC migration, and transcriptomic analysis suggested oxidative stress response as a key target mechanism of this function. Oxidative stress is a hallmark of fetal alcohol toxicity, and we found increased penetrance of facial and ocular malformations in *alx1* mutants exposed to ethanol, consistent with a protective role for *alx1* against ethanol toxicity. Collectively, these data demonstrate a conserved role for zebrafish *alx* genes in controlling ocular and facial development, and a novel role in protecting these key midfacial structures from ethanol toxicity during embryogenesis. These data also reveal novel roles for *alx* genes in ocular anterior segment formation and vascular development and suggest that retinal deficits in *alx* mutants may be secondary to aberrant ocular vascularization and anterior segment defects. This study establishes robust zebrafish models for interrogating conserved genetic mechanisms that coordinate facial and ocular development, and for exploring gene­–environment interactions relevant to fetal alcohol syndrome.

## Abstract (precis)

**Summary:** We show that zebrafish alx1 and alx3 function redundantly to regulate ocular and facial development, and identify a novel protective function of alx1 against ethanol toxicity.

## INTRODUCTION

The vertebrate face comprises ocular, vascular and skeletal cell lineages that are closely apposed during development. The anterior cranial neural crest (aCNC) originates from the anterior–dorsal neural tube (future forebrain and midbrain), migrates cephalad to the eyes and turns caudally toward the nascent mouth opening to give rise to facial chondrocytes (Kague et al., 2012; Kish et al., 2011; Kuratani, 2018; Serbedzija et al., 1992; Wada et al., 2005), ocular anterior segments, e.g. the cornea and iris (Cavodeassi et al., 2019; Cvekl and Tamm, 2004; Ma and Lwigale, 2019; Williams and Bohnsack, 2015), and vascular pericytes of ocular blood vessels (Gage et al., 2005; Trost et al., 2013). Despite their importance, the gene networks that regulate these aCNC-derived lineages are poorly understood.

The *Aristaless-like* (*alx*) gene family consists of *ALX1*, *ALX3* and *ALX4* in human, mouse and zebrafish; *ALX4* is represented by duplicated *alx4a* and *alx4b* in zebrafish (Dee et al., 2013). In humans, *ALX1* mutations are linked to severe frontonasal dysplasia (FND) and extreme microphthalmia, and *ALX3* and *ALX4* genes are associated with a phenotypic spectrum of hypertelorism and nasal-tip duplications (Pini et al., 2020; Uz et al., 2010). These functions of *alx* genes are conserved in other vertebrates, but the pathogenic developmental mechanisms leading to the observed craniofacial malformations remain largely unknown (Beverdam et al., 2001; Dee et al., 2013; Forsthoefel, 1963; Lakhwani et al., 2010; Lyons et al., 2016; Pini et al., 2020; Qu et al., 1999; Zhao et al., 1996). In mice, disruption of *alx1* results in anencephaly, precluding analysis of its role in facial and ocular morphogenesis, while compound mutants of *alx3* and *alx4* present with FND-like defects (Beverdam et al., 2001; Lakhwani et al., 2010).

Mouse *alx* family members, *alx1*, *alx3* and *alx4*, are co-expressed in aCNC that gives rise to the anterior neurocranium (ANC), the primary palate (Dee et al., 2013; McGonnell et al., 2011; Mitchell et al., 2021; Qu et al., 1999; ten Berge et al., 1998), anterior segment and periocular neural crest (Ma and Lwigale, 2019; Sedykh et al., 2017). Recent reports demonstrate that zebrafish *alx1* is required for regulating neural crest differentiation and migration *in vitro* and *in vivo* (Pini et al., 2020) and that zebrafish *alx3* controls ANC chondrocyte differentiation in zebrafish (Mitchell et al., 2021). However, these studies do not address the role for *alx* function in the developing eye, nor do they examine the potential for compensatory interactions between *alx1* and *alx3*.

While hereditary FND is rare, similar facial and ocular malformations are commonly associated with fetal alcohol exposure in humans (Farlie et al., 2016; Lovely et al., 2017). Genetic susceptibility to ethanol toxicity is actively studied in model organisms (Boschen et al., 2021; de la Morena-Barrio et al., 2018; Eberhart and Parnell, 2016; Kaminen-Ahola, 2020; Lovely et al., 2017), but much remains to be learned about the mechanisms of gene–environment interactions, particularly at the level of transcription.

Here we report that *ALX1* homozygous loss-of-function mutation is associated with severe ocular malformation in humans and show that *alx1* mutant fish also develop with ocular impairments. Targeted mutagenesis of a paralogous gene, *alx3*, was carried out to generate *alx1;alx3* double-mutant zebrafish and to demonstrate that variable penetrance of *alx1* mutant defects in zebrafish is due to functional redundancy with *alx3*. Our data demonstrate genetic requirements for *alx1* and *alx3* in craniofacial cartilage development, at least in part through regulating migratory aCNC that gives rise to the median element of the ANC. Importantly, we report evidence of unexpected roles for *alx1* and *alx3* in ocular vasculature and identify a role for *alx1* and *alx3* in regulating oxidative stress response and ribosome biogenesis. Consistent with these findings, zebrafish *alx1* protects ocular and facial embryonic lineages against the effects of ethanol toxicity, which acts through ethanol-induced oxidative stress in neural crest cells. Collectively, these data establish the *alx1* and *alx1;alx3* zebrafish models as unique new tools for dissecting both the genetic controls and gene-environment interactions during craniofacial and ocular development.

## RESULTS

### Human *ALX1* and zebrafish *alx1* are required for ocular development

**Fig. 1.**
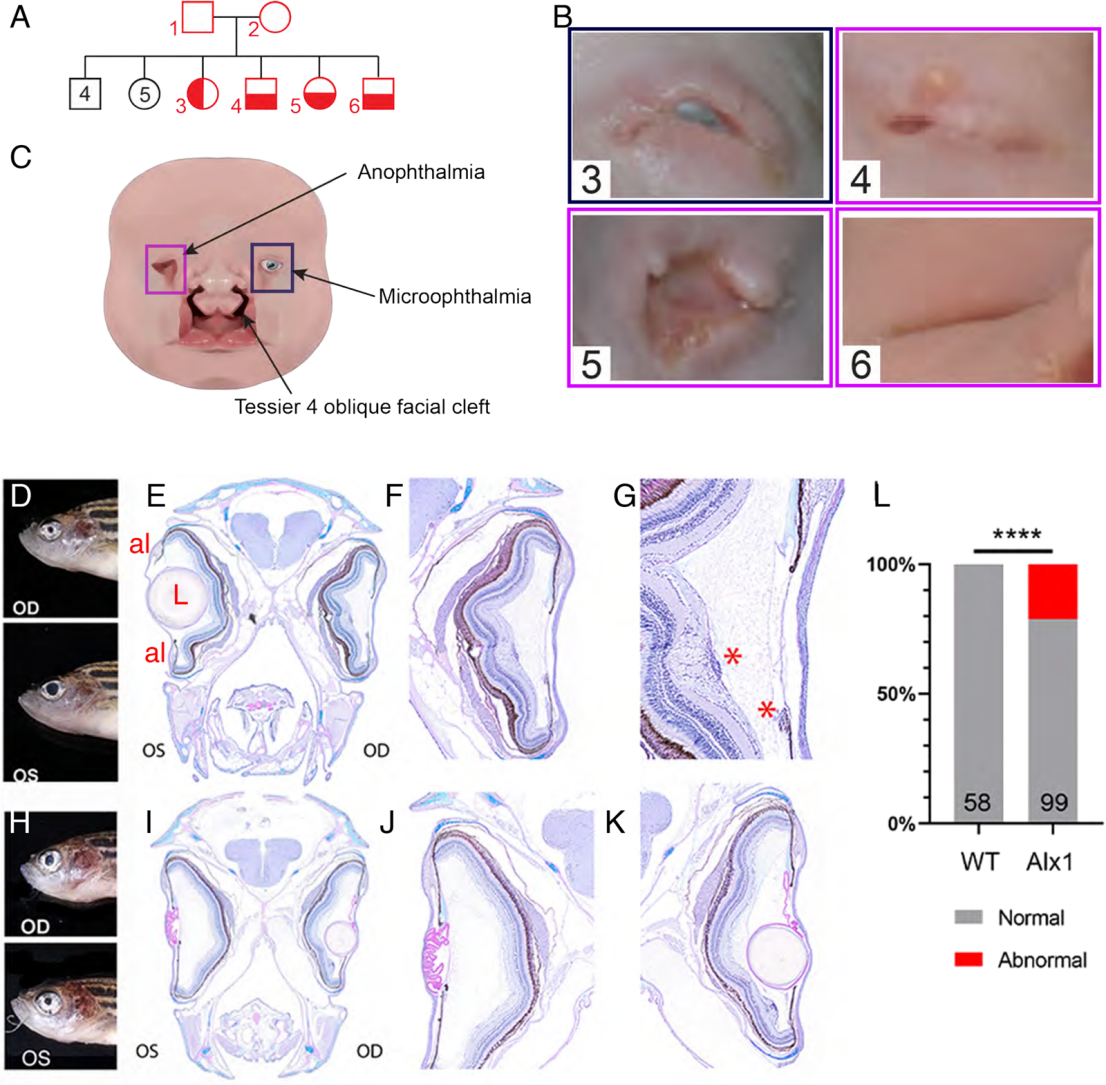
**Ocular malformations are associated with *ALX1/alx1* loss-of-function in humans and zebrafish.** (A) Consanguineous *ALX1^L165F^*/+ parents produced 13 children, four of whom were homozygous for *ALX1^L165F^* and had complex frontonasal dysplasia (FND3) (subject numbers noted in red). Unaffected individuals did not have eye or facial phenotypes suggestive of FND. (B) Diagram summarizing the anophthalmia, coloboma and bilateral Tessier oblique facial clefts (ObFC) characteristic of FND3. (C) Human subjects displayed bilateral ObFC (not shown) and a range of ocular malformations. The eldest sibling (subject 3) presented with right coloboma and left microophthalmia. The next child (subject 4) presented with bilateral anophthalmia with fused eyelids and shallow orbits. Subject 5 presented with bilateral anophthalmia with open shallow orbits. The upper and lower eyelids were absent, exposing the orbital mucosa. The nasal alae were also malformed with nodular skin tags. Subject 6 had bilateral anophthalmia, fused eyelids, and shallow orbit, similar to subject 4. (D-L) *alx1^uw2016^* adult zebrafish exhibit milder ocular defects, primarily in the anterior ocular segment. (D-G) A representative *alx1^uw2016^* adult with a unilateral ocular malformation (D). (E) Transverse section through the head of the fish in E shows a normal left eye and aphakia (absent lens) on the right. (F) The right eye lacks the lens and the ventral annular ligament but is largely normal otherwise. (G) The right eye contains mononuclear inflammatory cells near the ventral iris and the optic nerve head (asterisks). (H-K) A representative *alx1^uw2016^* zebrafish with bilateral ocular defects (H). (J) In transverse section through the head, the left eye is missing the lens (aphakia) and contains a thick, multifocally disrupted lens capsule, which is embedded in the anterior chamber. There is no annular ligament on the dorsal iridocorneal angle. (K) The right eye contains a small spherical lens nucleus inside a wrinkled lens capsule. The ventral iridocorneal angle is devoid of the annular ligament. (L) Ocular defects were observed in 21.2% of *alx1^uw2016^* homozygotes examined, and none of the wildtype fish of a similar age (****P<0.0001, Fisher's exact test). OD: *oculus dexter,* right eye; OS: *oculus sinister*, left eye; al: annular ligament; L: lens; *, mononuclear inflammatory cells.

We recently reported a pedigree with consanguineous parents and 13 children, four of whom were born with FND linked to a missense gene variant, *l165f*, in the homeodomain of *alx1* (Pini et al., 2020) (Fig. 1A). All four *ALX1*-homozygous patients presented with oblique orofacial clefts together with severe bilateral ocular deficits (Fig. 1B,C). These findings are consistent with a previous study that described patients with deletions that encompassed *ALX1* and other loci, and a patient with splice-site mutation at the *ALX1* locus (Uz et al., 2010). While the mechanism of *ALX1* function in the facial cartilage is coming to light through active investigation, the role for *ALX1* during ocular development remains unknown. We sought to address this knowledge gap by establishing zebrafish models of *ALX1*-linked FND. Using CRISPR/Cas9-mediated targeted mutagenesis, we generated loss-of-function alleles in zebrafish *alx1* and showed that the majority of *alx1* homozygotes were viable as adults, with a small proportion exhibiting craniofacial malformations that manifest during larval stages (Pini et al., 2020). On gross anatomical examination, we observed a range of ocular impairments in *alx1^uw2016^* adults. Histological analysis of four representative affected adults revealed aberrant pupils, missing lenses (aphakia) and iridial dysplasia (Fig. 1D-K), but largely normal retinae. These ocular anomalies occurred in at least one eye in a significant proportion of *alx1^uw2016^* adults (21 of 99 total, *P*<0.0001, Fig. 1L). These findings suggest a post-embryonic requirement for zebrafish *alx1* function, which may be restricted to the anterior segment lineages of the eye.

### Zebrafish *alx1* and *alx3* function redundantly during craniofacial lineage formation

*alx1* and its homologs, *alx3* and *alx4*, are co-expressed in aCNC, which gives rise to the median element of the ANC (Dee et al., 2013; McGonnell et al., 2011; Mitchell et al., 2021; Qu et al., 1999; ten Berge et al., 1998), as well as to the ocular anterior segment and periocular neural crest (Ma and Lwigale, 2019; Sedykh et al., 2017). All four single-locus mutants have been analyzed but only *alx1* mutants (Pini et al., 2020) and *alx3* mutants (Mitchell et al., 2021) exhibited embryonic defects. These defects were far milder than those reported in antisense morpholino-mediated knockdown assays (Dee et al., 2013) and did not recapitulate the severe craniofacial and ocular phenotypes in *ALX1*-linked (Pini et al., 2020; Uz et al., 2010) or *ALX3-*linked (Twigg et al., 2009) FND patients. These findings point to redundant or compensatory functions of *alx* genes in the embryonic zebrafish.

A recent study used single cell RNA-seq to show that all four zebrafish *alx* genes are expressed in frontonasal neural crest at 24 hours post fertilization (hpf) (Mitchell et al., 2021). We have also shown that *alx3* transcription is increased in *alx1* mutants (Pini et al., 2020), likely through the process of transcriptional adaptation (El-Brolosy et al., 2019). We hypothesized that the low penetrance of craniofacial malformations in *alx1* mutants is due to functional compensation by *alx3* and used CRISPR/Cas9 mutagenesis to mutagenize *alx3* in the *alx1^uw2016^* background (see Materials and Methods). Three frame-shift mutant alleles were recovered; since homozygotes for each allele developed with similar phenotypes, we chose one of the alleles, a 13-nucleotide insertion allele named *alx3^uw2113^*, for in-depth analysis. *alx3^uw2113^* is predicted to encode a non-functional protein due to premature translation termination that results from a frame-shift mutation upstream of the conserved homeodomain and transactivation domains of alx3 (Fig. S1).

**Fig. 2.**
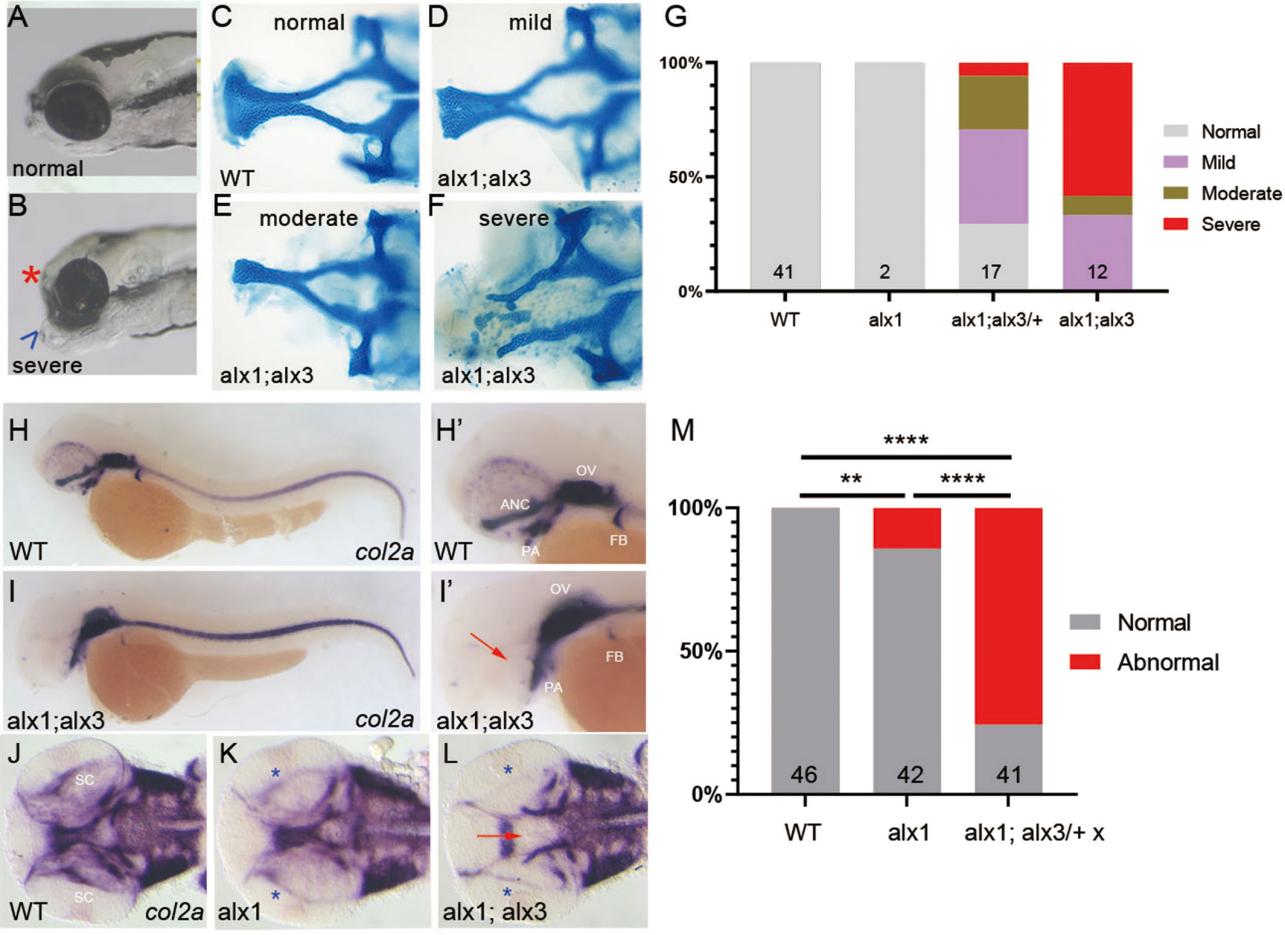
***alx1* and *alx3* function redundantly to control anterior neurocranium morphogenesis in zebrafish.** Larvae derived from a cross between *alx1;alx3/+* parents were fixed at 5 dpf. (A,B) Representative siblings with normal facial structures (A) and with aberrantly short neurocranium (asterisk) and abnormally protruding jaw cartilage (arrowhead; B). (C-F) Alcian Blue staining revealed EP anomalies that range from mild (reduced median ethmoid plate width in D), moderate (absent median and reduced lateral ethmoid plate in E), to severe (absent ethmoid plate in F). (G) Penetrance and severity of cartilage defects is increased in *alx1;alx3* compared to *alx1;alx3/+* embryos (two trials). (H-M) Embryos derived from wildtype, *alx1, or alx1;alx3/+* parental crosses were fixed at 2 dpf and stained for *col2a1a* expression using whole-mount *in situ* hybridization. (H,H′) Normal *col2a1a* expression in a wildtype embryo. (I,I′) A truncated EP in an *alx1;alx3* embryo (arrow). (J) Normal *col2a1a* expression in a wildtype embryo. (K) Normal ethmoid plate and reduced presumptive scleral staining (asterisk) in an *alx1* mutant. (L) Hypoplastic ethmoid plate (arrow) and absent presumptive scleral staining (asterisk) in an *alx1;alx3* mutant. ANC, anterior neurocranium; OV, optic vesicle; PA, pharyngeal arches; FB, fin bud; SC, sclera. M: 75% of the embryos derived from *alx1;alx3/+* have aberrant ANC and reduced scleral precursors (**P=0.0097, ****P<0.0001, Fisher's exact test; two trials for wildtype and *alx1;alx3*/+, and one trial for *alx1*). Embryos in A,B, and H-I′ are shown in lateral views, anterior to the left. Embryos in J-L are shown in ventral views, anterior to the left.

*alx1^uw2016^;alx3^uw2113^/+* and *alx1^uw2016^;alx3^uw2113^* (abbreviated *alx1;alx3/*+ and *alx1;alx3*) zebrafish were viable as adults and fertile, with *alx1;alx3* adults exhibiting a range of facial anomalies (data not shown). *alx1;alx3* larvae developed with a distinctive facial phenotype consistent with hypoplastic ANC at 5 days post-fertilization (dpf) (Fig. 2A,B). Alcian Blue staining, which labels neural crest-derived chondrocytes, confirmed ANC hypoplasia with various degrees of severity in the mutant larvae (Fig. 2C-F). Notably, *alx1;alx3/+* embryos also developed with anomalous ANC primordia, albeit milder and at a lower penetrance (Fig. 2G).

We next sought to define the earliest time that craniofacial cartilage defects manifest in *alx1;alx3* embryos. To do this, we examined expression of *col2a1a*, an early chondrocyte marker (Yan et al., 1995) in wildtype, *alx1^uw2016^* (abbreviated *alx1*), and *alx1;alx3* embryos at 2 dpf. The majority of *alx1* embryos (88%) exhibited normal expression of *col2a1a* (Fig. 2H,M), consistent with the low penetrance of deficits by 5 dpf. In contrast, only 24% of embryos derived from an *alx1;alx3/+* incross developed with normal ANC, while 76% had truncated or missing ANC at 5 dpf (Fig. 2I,I′,L,M). This phenotypic ratio indicates that the ANC primordium is aberrant in both *alx1;alx3* and *alx1;alx3*/+ embryos by 2 dpf. *col2a1a* expression was also detected in the presumptive sclera, the cartilaginous outer shell of the eye globe contiguous with the cornea, which is derived from neural crest (Gestri et al., 2012). *col2a1a* expression in putative scleral precursors was reduced in *alx1* and *alx1;alx3* embryos (Fig. 2K,L), suggesting an early requirement for *alx1* and *alx3* function in the scleral lineage.

### Zebrafish *alx1* and *alx3* function redundantly during ocular morphogenesis

**Fig. 3.**
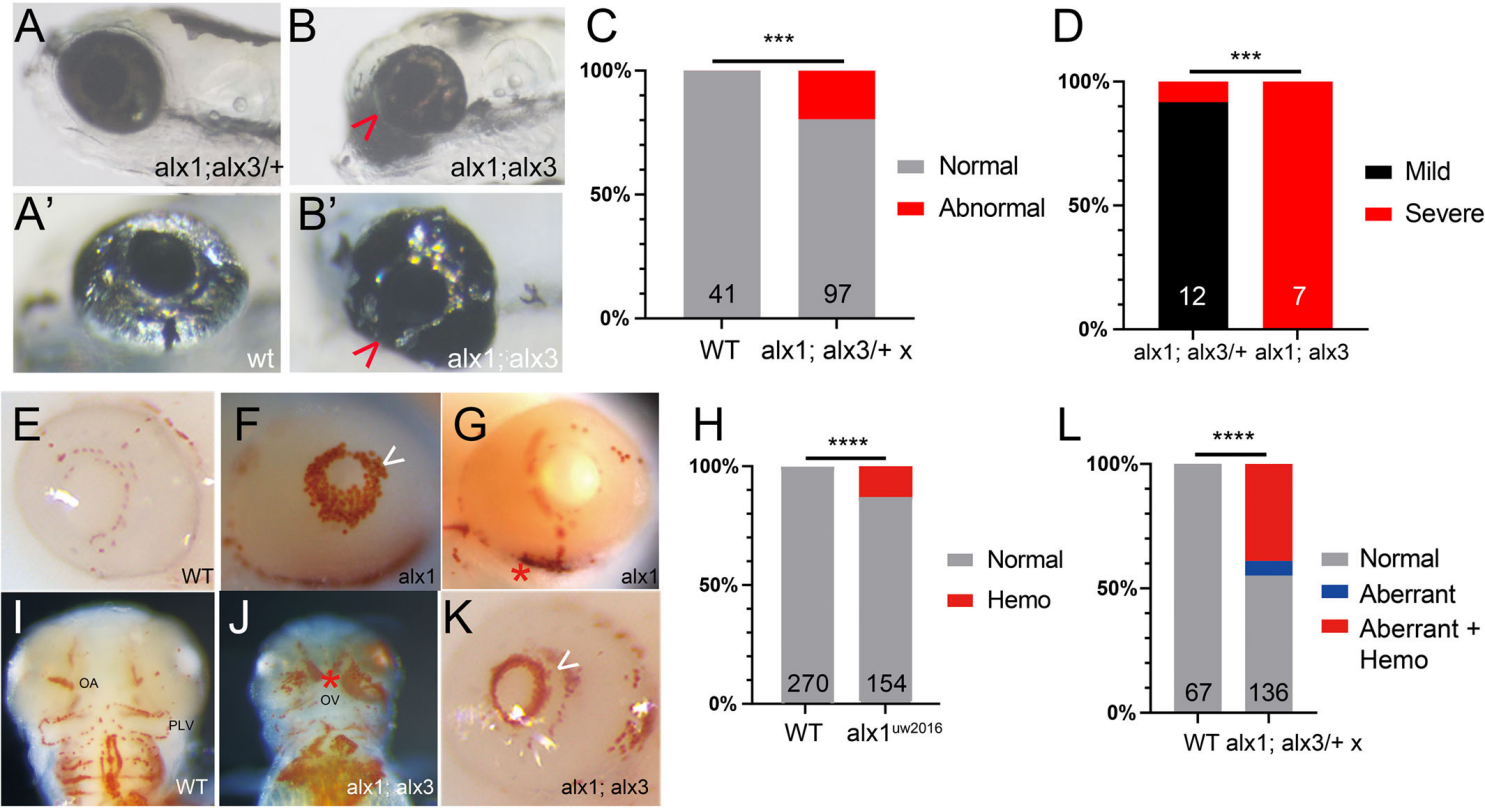
**Zebrafish *alx1* and *alx3* function redundantly during ocular morphogenesis.** (A,B) Larvae generated from a cross between *alx1;alx3/+* parents were scored at 5 dpf for ocular defects. An *alx1;alx3/+* larva with normal ocular morphology (A) similar to that of a wildtype embryo (A′). (B) An *alx1;alx3* sibling with ocular coloboma and a misshapen eye (severe defect, arrowheads in B,B′). (C) These ocular defects are restricted to progeny of *alx1;alx3/+* parents (***P=0.0009, Fisher's exact test, two trials). (D) The severe ocular defect shown in B is strongly associated with the *alx1;alx3* genotype, while the majority of *alx1;alx3*/+ embryos present with a milder misshapen eye phenotype (not shown) (***P=0.0002 using Fisher's exact test, two trials). (E-L) Embryos derived from wildtype or *alx1;alx3/+* parents were stained with *o*-dianisidine at 2-3 dpf to label hemoglobin in red blood cells. Normal hemoglobin distribution in the eye of a wildtype embryo (E) versus ocular hemorrhage (arrowhead in F) and periocular hemorrhage (asterisk in G) in *alx1* mutants. (H) Hemorrhaging is restricted to a subset of *alx1* mutants (****P<0.0001, Fisher's exact test, five trials). Normal hemoglobin distribution in the pharyngeal region of a wildtype embryo (I) versus evidence of hemorrhaging in an *alx1;alx3* embryo (J). Intraocular and periocular hemorrhages (asterisk) near the optic artery and optic vein in an *alx1;alx3* embryo (K). (L) Hemorrhaging is strongly associated with eye defects in *alx1;alx3*/+ and *alx1;alx3* embryos (****P<0.0001, Chi-square test, two trials). Embryos are shown in lateral views, anterior to the left except in I and J, which are ventral views, anterior to the top. OA, optic artery; OV, optic vein; PLV, palatocerebral vein.

We next asked if *alx3* function compensated for the loss of *alx1* during ocular morphogenesis. Embryos obtained from a cross between *alx1;alx3/+* parents exhibited aberrantly shaped (elongated) eye capsules covered by abnormally patchy iridophores at 5 dpf (Fig. 3A-C). Genotypic analysis confirmed this phenotype, which we termed severe, to be strongly linked to the *alx1;alx3* genotype. We also observed a milder manifestation of this ocular phenotype in *alx1;alx3* larvae (Fig. 3D).

Unexpectedly, we also observed sporadic intraocular and periocular hemorrhage in living a*lx1* and *alx1;alx3* embryos at 2-3 dpf. We employed *o*-dianisidine, a histological stain for erythrocytes, to visualize the aberrantly pooled erythrocytes and to delineate the vascular network. At this stage in development, ocular blood supply is carried by a branched hyaloid network that starts forming around the lens at 32 hpf (Hartsock et al., 2014). In wildtype embryos, erythrocytes were distributed in a pattern that resembled normal hyaloid vasculature (Fig. 3E) (Detrich et al., 1995). This was also true for most *alx1* mutants, with a minority exhibiting patches of erythrocytes suggestive of hyaloid hemorrhaging (Fig. 3F-H). In *alx1;alx3* homozygotes, *o*-dianisidine staining revealed extensive and penetrant ocular and periocular hemorrhage (Fig. 3I-K). Ocular hemorrhage was present in ∼50% of *alx1;alx3/+* incross progeny and was strongly associated with ocular dysmorphology (Fig. 3L). Thus, hyaloid hemorrhage is a highly penetrant phenotype linked to *alx1;alx3* homozygosity. Notably, this defect emerges contemporaneously with the loss of *col2a1a* expression in ANC precursors and in the sclera (see Fig. 2L, asterisk).

To examine hyaloid vasculature directly, we took advantage of endogenous alkaline phosphatase activity in the vascular endothelium surrounding the lens (Alvarez et al., 2007). Larvae derived from an *alx1;alx3/+* incross were sorted by ocular morphology at 5 dpf and stained for alkaline phosphatase. Examination of dissected lenses revealed a range of vascular defects, from mild disorganization to complete absence (Fig. S2). Surprisingly, severity of vascular defects did not correlate with presence or severity of ocular dysmorphology. Together, these data indicate a novel function for the *alx* family in the developing hyaloid vasculature.

### *alx1* and *alx3* are largely dispensable for early retinal development

**Fig. 4.**
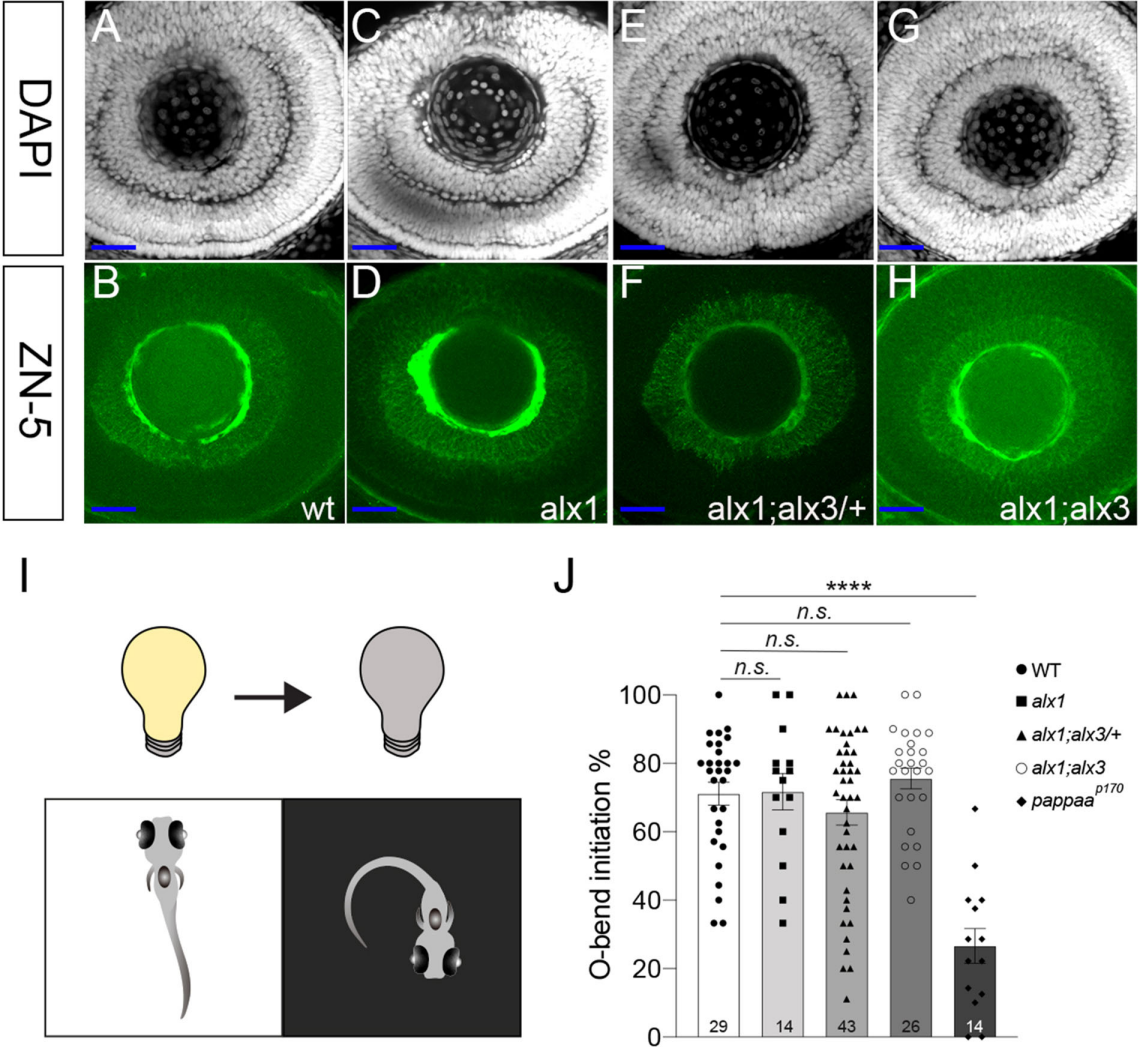
***alx1* and *alx3* functions are dispensable for retinal ganglion cell differentiation and retinal function in zebrafish larvae.** Embryos derived from wildtype or *alx1;alx3/+* incrosses were fixed at 2 dpf and stained with zn5, a retinal ganglion cell (RGC) marker, and DAPI, a nuclear co-stain (A-H). (A,B) A representative wildtype eye shows strong zn5-labeled RGCs and a characteristic layered nuclear organization (of four embryos). (C,D) Normal RGC and nuclear patterns in an *alx1* mutant (of two embryos). (E,F) Normal RGC and nuclear patterns in a representative *alx1;alx3/+* retina (of four embryos). (G,H) Normal RGC and nuclear patterns in a representative *alx1;alx3* retina (of two embryos). Left eyes are shown in lateral views, anterior to the left. Scale bar, 25 µm. (I) Diagram summarizing the dark flash assay, with a lights-off stimulus eliciting O-bend turn responses in 5 dpf larval zebrafish. (J) Mean frequency of O-bend initiations to a series of 10 dark flash stimuli was measured in larvae derived from wildtype or *alx1;alx3/+* crosses, and larvae were genotyped *post-hoc*. n.s.: *P*>0.9999 versus wildtype control; ****P<0.0001 versus wildtype control. ANOVA with Kruskal–Wallis test. Error bars indicate s.e.m.

Having documented ocular defects in supporting ocular tissues, we asked if retinal development depended on *alx1* and *alx3* function. *alx1;alx3/+* incross progeny were fixed at 2 dpf and immunostained with an anti-zn5/alcama antibody, which labeled retinal ganglion cell (RGC) neurons, and counter-stained with the nuclear stain DAPI to reveal retinal layers. Confocal imaging revealed a normal pattern of RGCs and correct overall layering in the retinae of *alx1, alx1;alx3/+,* and *alx1*;*alx3* larvae (Fig. 4A-D).

Next, we tested visually triggered behaviors to assess retinal function in *alx1;alx3* larvae at 5 dpf. When presented with an abrupt absence of light (a dark flash), larval zebrafish execute a turning behavior termed the ‘O-bend’, in which they turn their bodies ∼180 degrees (Burgess and Granato, 2007) (Fig. 4I). We exposed 5 dpf larvae to 10 dark flashes and evaluated the probability of O-bend initiation in response to the abrupt absence of light. We found that *alx1*, *alx1;alx3*, and *alx1;alx3*/+ larvae initiated the O-bend at the same frequency as the wildtype larvae (Fig. 4J), while visually impaired *pappaa^p170^* mutants showed a marked reduction in O-bend initiation compared to wildtypes (Miller et al., 2018). This analysis shows that *alx1;alx3* larvae were able to detect and respond to luminescence changes in their visual field, suggesting their retinae are functional at 5 dpf.

**Fig. 5.**
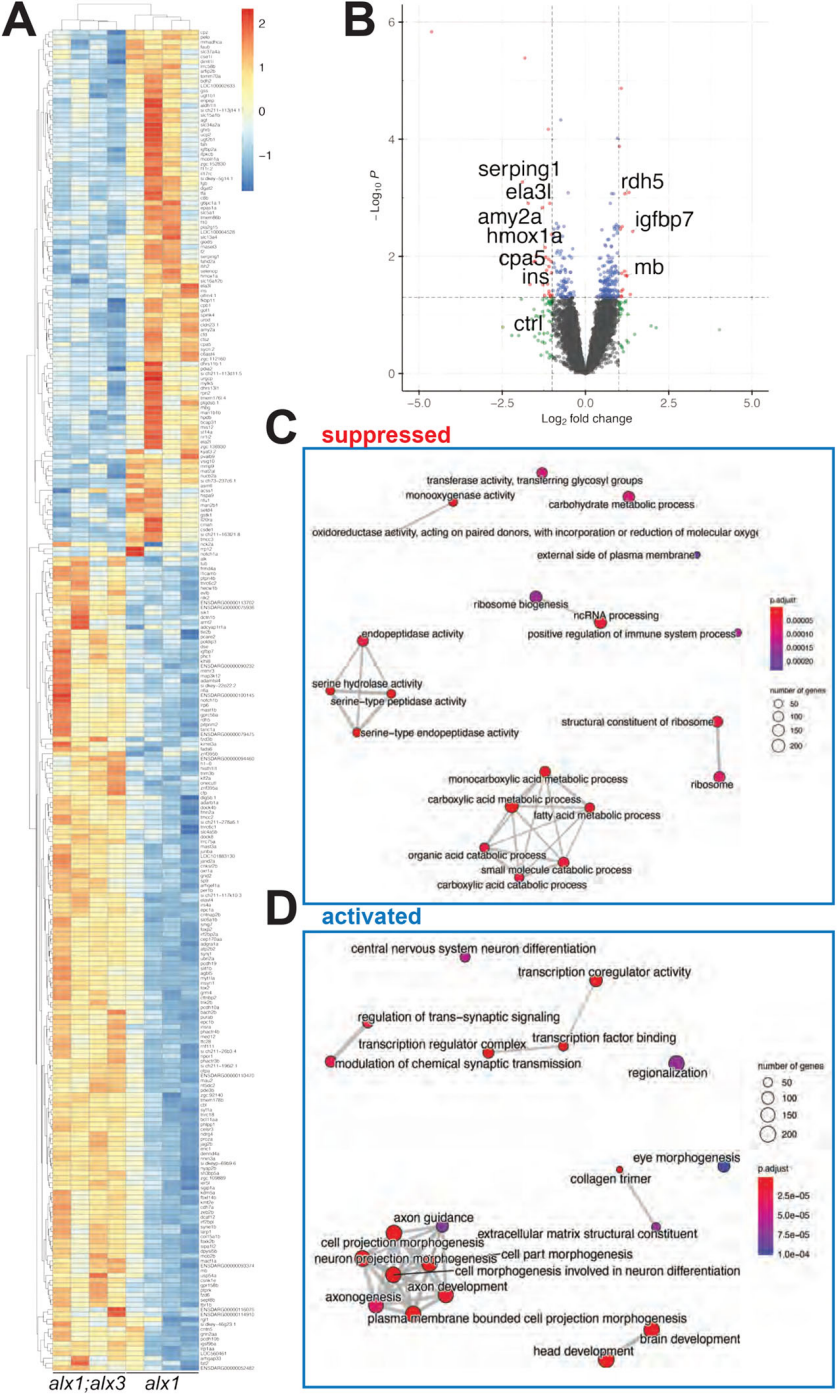
**RNAseq transcriptome analysis identifies perturbation of ocular development pathways in *alx1;alx3* mutants.** Larvae derived from *alx1^uw2016^;alx3^2113^/+* parents were lysed individually at 5 dpf for RNA extraction and Illumina high-throughput sequencing. A heat map (A) of significantly dysregulated genes (FDR<0.5) in *alx1uw2016;alx3uw2113* double mutants relative to *alx^uw2016^* siblings. A volcano plot (B) of statistical significance against fold-change in *alx1^uw2016^;alx3^uw2113^* mutants relative to *alx^uw2016^* siblings. Vertical lines indicate −1-fold and +1-fold cut-offs and the horizontal line indicates the FDR=0.05 cut-off. (C,D) The top 20 enriched and top 20 suppressed terms in *alx1^uw2016^;alx3^uw2113^* double mutants relative to *alx^uw2016^* siblings, identified by GSEA.

We next set out to identify transcriptional targets that depend on *alx1* and *alx3* functions in the post-embryonic zebrafish larvae. Embryos were derived from an *alx1;alx3/+* incross and RNA was harvested from individual siblings without pooling at 5 dpf, followed by RNA-seq. For each sample, sequence tracks were examined for the presence of the 13 base-pair insertion in the *alx3^uw2113^* allele to deduce the embryonic genotype. 12 samples (four each of *alx1*, *alx1;alx3/+* and *alx1;alx3*) were selected for pairwise comparisons as detailed in Materials and Methods. This approach identified a compact set of 275 differentially expressed genes (DEGs) between *alx1;alx3* and their *alx1* siblings (Fig. 5A, Table S1), and 34 genes differentially expressed between *alx1;alx3/+* larvae and *alx1* siblings (Fig. S3); six of the differentially expressed genes were shared between the two sets. Among DEGs with lineage-restricted expression patterns were several retinal markers that were significantly increased in *alx1;alx3* versus *alx1* homozygotes at 5 dpf. These differentially expressed genes included *cels3*, expressed in the amacrine and ganglion cells of the zebrafish retina (Lewis et al., 2011), and *rdh*5, expressed in retinal pigment epithelium (RPE) (Nadauld et al., 2006). Rdh5 is a retina-specific retinol dehydrogenase required for ventral retinal morphogenesis; its upregulation in *alx1;alx3* mutants is consistent with expansion of the RPE. *igfpb7*, which is highly expressed in vascular endothelium (Abu-Safieh et al., 2011), was also upregulated in *alx1;alx3* and in *alx1;alx3/+* siblings (Fig. 5B, Fig. S3).

The downregulated DEG list was enriched for pancreatic and liver markers, namely, *serping1, hmox1a, ela2l, cpa5, ctrl, ela3l, ins and amy2a* (Cheng et al., 2006; Holowiecki et al., 2017) suggesting an unexpected role for *alx1* and *alx3* in these primordia. Two of these genes, *serping1* and *ela2l*, were also downregulated in *alx1;alx3/+* versus *alx1* siblings (Fig. 5B, Fig. S3).

We next used gene set enrichment analysis (GSEA) (Subramanian et al., 2005) to deeply analyze molecular pathways that require *alx1* and *alx*3 function. This analysis identified several molecular pathways that were suppressed or activated in *alx1;alx3* larvae. Ribosome biogenesis was identified as significantly suppressed (Fig. 5C, Fig. S4), while brain and head development, neuronal development and eye morphogenesis were notable among those activated in *alx1;alx3* larvae compared to *alx1* siblings (Fig. 5D, Fig. S5, Table S2).

### Zebrafish *alx1* and *alx3* function during migration of presumptive anterior neurocranium progenitors

**Fig. 6.**
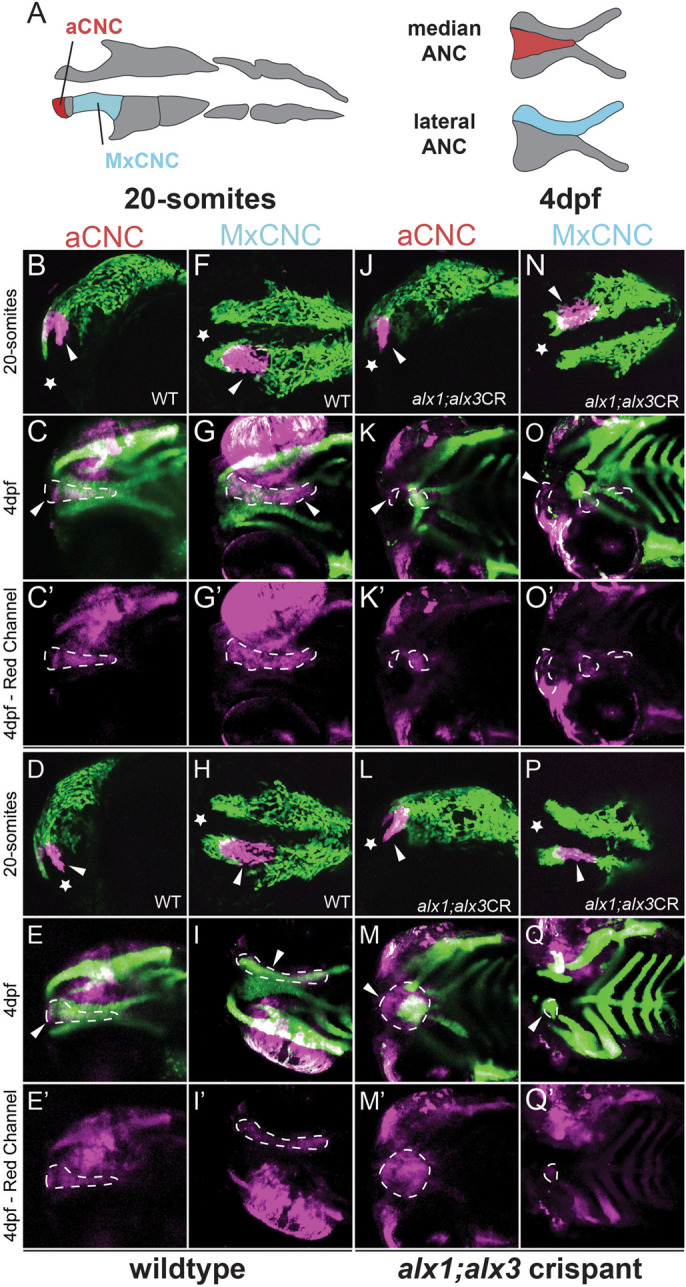
**Zebrafish *alx1* and *alx3* functions are required in migratory anterior cranial neural crest.** (A) A schematic fate map of 20-somite-stage cranial neural crest at 4 dpf. The anterior cranial neural crest (aCNC) contributes to the median element of the anterior neurocranium (ANC) while the maxillary stream of migrating CNC (MxCNC) contributes to the maxillary prominence and subsequently coalesce to form the lateral element of the ANC. B-Q: aCNC and MxCNC were labeled by kaede photoconversion (magenta) at 20 somites and imaged immediately, allowed to develop until 4 dpf and imaged again (20-somites images: star sign marks the anterior; 4 dpf images: anterior to the left). *alx1-/-;alx3* crispants exhibit a midline cleft (K,M,O,Q) with malformed median and lateral elements. The aCNC and MxCNC migrating streams failed to reach their destinations and ectopically localized along the migratory path (arrowheads in K,M,O,Q). Note that ocular fluorescence is due to the presence of iridophores and retinal pigment epithelium in the eye, not to kaede fluorescence at 4 dpf. Arrowheads point to photoconverted cells and their progeny. Dotted line outlines the shape of the photoconverted cells at 4 dpf. Scale bar: 100 µm.

To gain additional insight into the requirement for *alx1* and *alx3* in the aCNC lineage, we performed *in vivo* lineage-tracing analysis. To do this efficiently, we adapted CRISPR-driven G0 knockout in zebrafish (Wu et al., 2018) to generate somatic mutations in the *alx3* locus in the *alx1* homozygous mutant background (*alx1;alx3CR*, also referred to as ‘crispants’). The *alx1;alx3CR* larvae exhibited midline clefts, abnormal trabeculae and abnormal anterior neurocranium (ANC) formation (Fig. S6) that resembled the severe defects observed in *alx1;alx3* embryos (see Fig. 2A-F). In contrast, *alx1* mutants and *alx3CR* in the wildtype background developed with normal craniofacial cartilages (Fig. S6). Groups of cells in the anterior/frontonasal (aCNC) or maxillary stream (MxCNC), fated to give rise to the ANC (Fig. 6A) (Knight and Schilling, 2006), were labeled by kaede photoconversion in *Tg(sox10:kaede)* (Fig. 6B,D,F,H) or *alx1;alx3CR; Tg(sox10:kaede)* embryos (Fig. 6J,L,N,P) at the 20 somite stage and imaged immediately. At 4 dpf, the larvae were imaged again to determine labeled aCNC locations post-migration. Wildtype larvae developed with normal cartilages (labeled in green) and showed normal post-migratory localization of the photo-converted red-kaede-labeled cells (magenta channel) in the median and lateral element of the ANC (Fig. 6C,E,G,I). In *alx1;alx3CR*s with severely abrogated ANC, red-kaede-labeled cells were found in ectopic locations proximal to the eyes (arrowheads in Fig. 6K,M,O,Q; 17 of 23 total, 2 trials). The median element of the ANC is missing and the lateral element is malformed (Fig. S6). These observations suggest that *alx1* and *alx3* affect the migration of the frontonasal and maxillary streams of cranial neural crest cells. There also appeared to be fewer crispant cells reaching their destination, consistent with a potential proliferative deficit. Notably, while expression of *alx1* and *alx3 is* primarily NCC-restricted (Dee et al., 2013; Sedykh et al., 2017), these findings do not rule out a non-cell-autonomous function of these transcription factors in tissues adjacent to the migrating neural crest.

### Transcriptomic analysis identifies candidate targets of *Alx1* and *Alx3* in the developing embryo

**Fig. 7.**
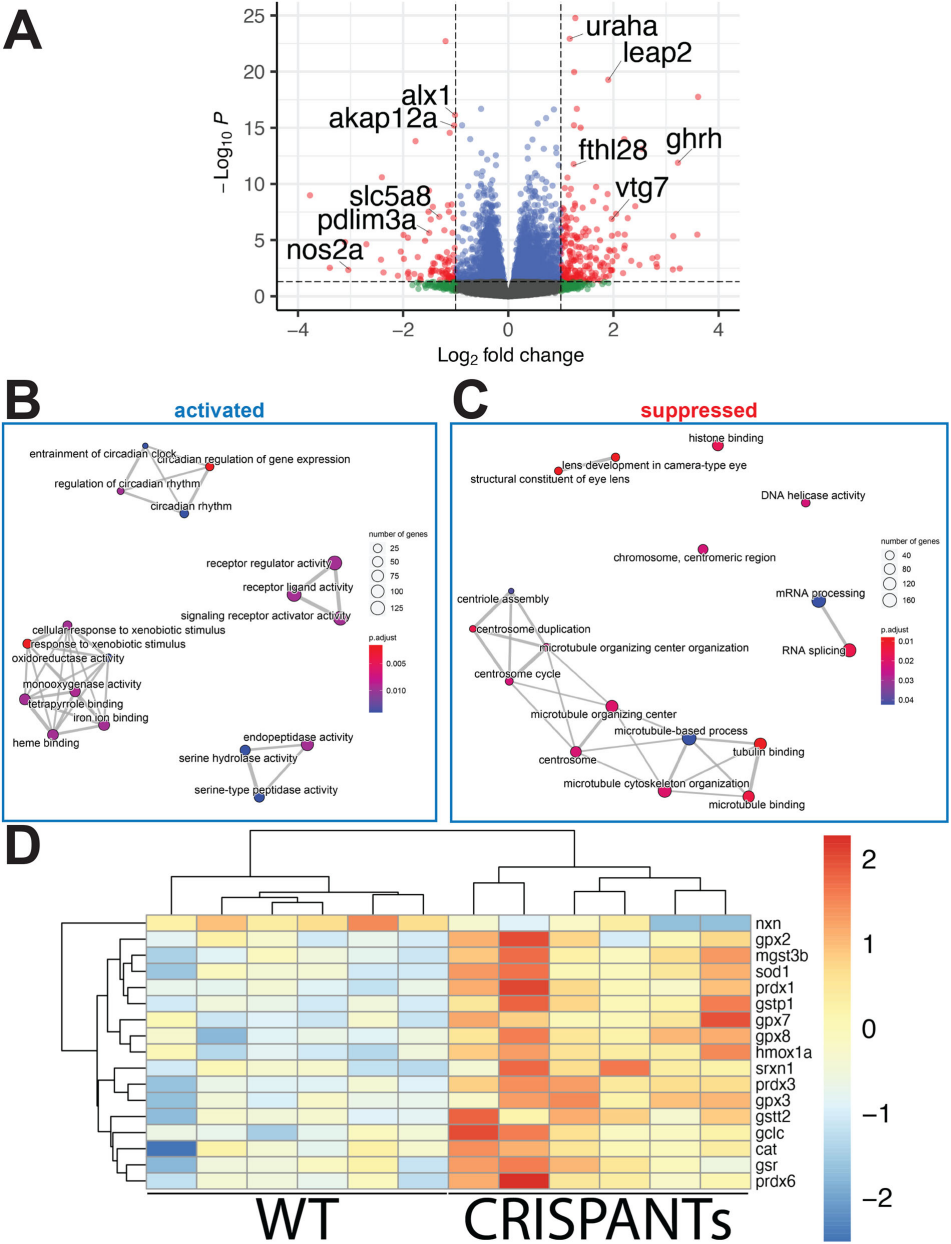
**RNAseq transcriptome analysis identifies a role for *alx1* and *alx3* in oxidative stress response regulation.** *alx1^uw2016^;Tg(sox10:kaede)* embryos were injected with alx3 CRISPR RNPs to induce somatic mutations at the *alx3* locus to generate *alx1;alx3CR* embryos. At 3 dpf,*alx1;alx3*CR embryos with characteristic craniofacial defects and age-matched *Tg(sox10:kaede)* embryos were lysed. A total of six replicate groups of 20 embryos from each genotype were collected in two independent experiments for RNA extraction and Illumina high-throughput sequencing. (A) Volcano plot shows the distribution of genes that are differentially expressed in *alx1;alx3*CR*;sox10:GFP* embryos relative to *sox10:GFP* embryos at 3 dpf. Vertical lines indicate −1.5-fold and +1.5-fold change cut-offs, and the horizontal line indicates the FDR=0.05 cutoff. (B,C) molecular pathways that are significantly suppressed (B) or activated (C) in *alx1;alx3CR;sox10:GFP* embryos were identified using GSEA, with top 20 activated and top 20 suppressed terms shown as a 2D map to emphasize overlaps. (D) Heatmap summary of differentially expressed oxidative stress response genes shows increased expression levels of these genes in *alx1;alx3CR;sox10:GFP* embryos.

Having demonstrated essential roles for zebrafish *alx1* and *alx3* in the forming frontonasal cartilage, anterior ocular segment and ocular vasculature, we sought to address the underlying molecular mechanisms through transcriptomic analysis. We surveyed transcriptome changes that result from loss of *alx1* and *alx3* at 3 dpf*,* when ocular and ANC defects in mutant embryos become evident. a*lx1;alx3CR* embryos were generated as detailed above, and age-matched wildtype controls were subjected to RNA isolation and high-throughput sequencing. Bioinformatic analysis identified a large set of differentially expressed genes (4426 genes with adjusted *P*-value <0.05), which included 207 strongly upregulated (Log_2_ fold change >1) and 93 downregulated (Log_2_ fold change <−1) genes (Fig. 7A, Table S3).

GSEA analysis of this data set showed suppression of lens development, the only pathway relevant to ocular development, in crispants (Fig. 7B, Fig. S7), while a group of pathways that include monooxygenase activity and xenobiotic metabolism was activated in crispants (Fig. 7C, Fig. S8, Table S4). Notably, several of the leading-edge gene sets contain *heme oxygenase 1* (*hmox1a*), also known for its critical functions in the cellular response to oxidative stress in several tissues, including the vascular endothelium (Fuse et al., 2015; Holowiecki et al., 2017). On further examination of the DEG set, a number of key oxidative-stress-response genes (Nakajima et al., 2011), summarized in the heatmap in Fig. 7D, were found to be upregulated in crispants. These data indicate novel requirements for *alx1* and *alx3* function in the developing lens, and in regulating the oxidative stress response program.

### Zebrafish *alx1* protects frontonasal and ocular lineages against ethanol toxicity

**Fig. 8.**
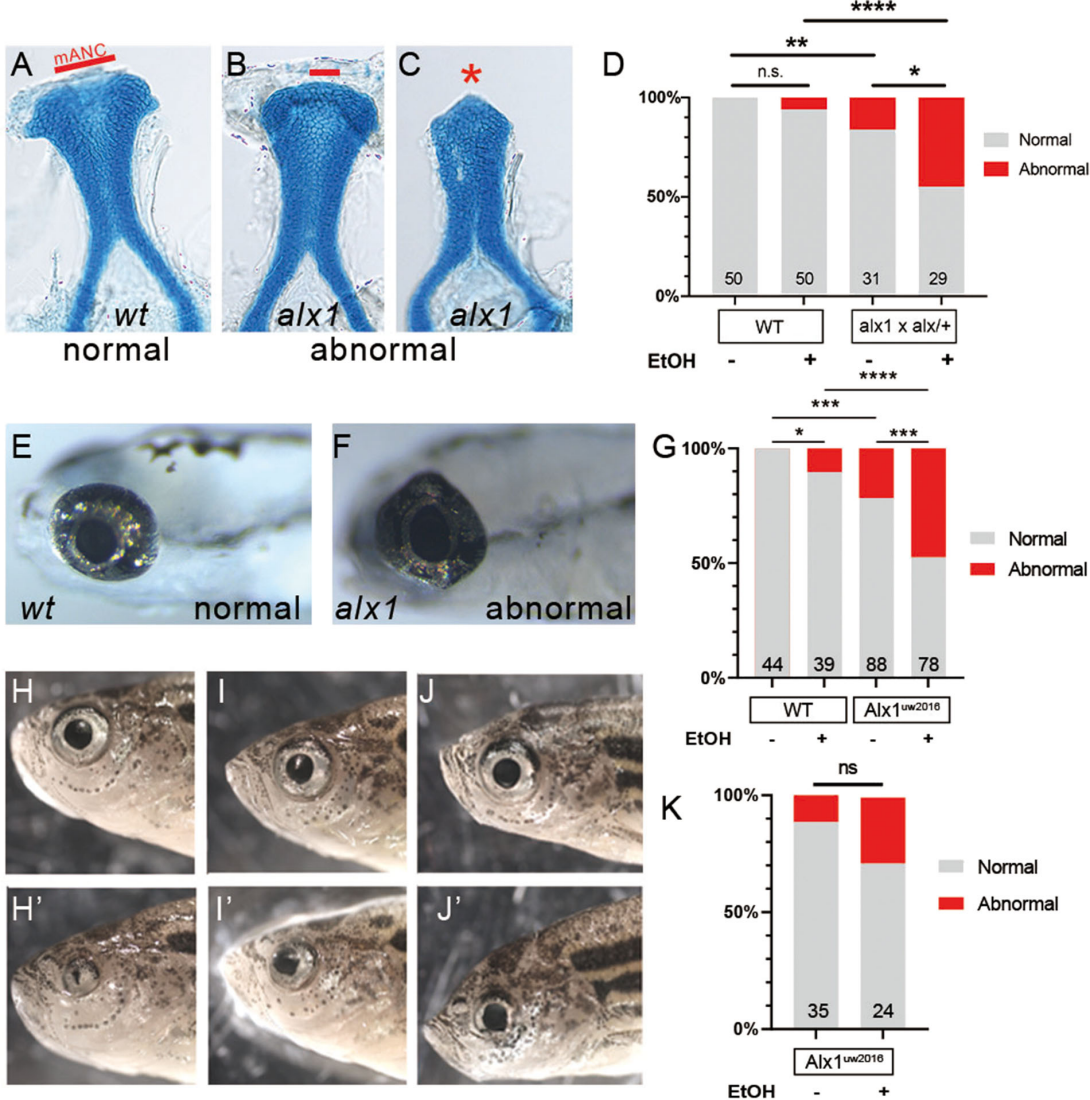
**Zebrafish *alx1* protects craniofacial and ocular lineages against ethanol toxicity.** Embryos derived from wildtype, *alx1^uw2016^*, or *alx1^uw2016^/+* parents were exposed to 0.5% ethanol from 6 hpf until 48 hpf and examined for craniofacial and ocular abnormalities at 5 dpf. (A-C) Embryos were stained with Alcian Blue to visualize anterior neurocranium (ANC) chondrocytes. Examples of normal ANC morphology (A) in an ethanol-treated wildtype larva, mildly dysmorphic median ANC in an ethanol-treated *alx1^uw2016^* mutant (B), and severely abrogated ANC with missing median element in an ethanol-treated *alx1^uw2016^* mutant. (D) Ethanol exposure does not increase penetrance of craniofacial defects in wildtype (n.s., not significant; *P*=0.2424) whereas it increases in progeny from *alx1*×*alx1/+* (*P=0.0237). Compared to untreated or treated wildtype, craniofacial defects are more penetrant in untreated or progeny from *alx1*×*alx1/+* (**P=0.0066, ****, *P*<0.0001), respectively. Fisher's exact test was used to measure statistical significance (three trials for wildtype and two trials for *alx1*×*alx1/+*). (E-G) Embryos were exposed to ethanol and examined for ocular anomalies at 5 dpf. (E) A representative 5 dpf wildtype larva with normal ocular morphology. (F) Misshapen eye in a representative *alx1* homozygote. (G) Ethanol exposure increases penetrance of ocular abnormalities in wildtype (*P=0.0448) and in *alx1^uw2016^* mutants (***P=0.0005). Compared to wildtype, abnormal ocular phenotype is more penetrant in both untreated *alx1* mutants (***P=0.0003) and in ethanol-treated ones (****P<0.0001). Fisher's exact test was used to measure statistical significance (one trial for wildtype and two trials for *alx1* mutants). (H-J) Three representative *alx1^uw2016^* adults exposed to 0.5% ethanol from 6 hpf until 48 hpf and raised to adulthood (2 months of age) exhibit unilateral ocular anomalies. (K) Penetrance of ocular defects increased following embryonic exposure to ethanol but did not rise to significance (n.s., *P*=0.1022. Fisher's exact test, one trial). (A-C) dissected ANC preparations, anterior to the top. (E,F) lateral views, anterior to the left. H and H′ are different sides of the same fish, as are I and I′, and J and J′, shown in lateral view. mANC: median ANC.

The aCNC is particularly sensitive to environmental influences and ethanol, a common environmental toxin and teratogen, is known to impact neural-crest-derived lineages through increasing oxidative stress and disrupting ribosome biogenesis (Kiecker, 2016; Lovely, 2020; Smith et al., 2014). We hypothesized that *alx1* function protects aCNC lineages against ethanol toxicity and took advantage of the low penetrance of defects in zebrafish *alx*1 mutants to test this hypothesis. Embryos derived from *alx1*×*alx1/+* crosses were exposed to 0.5% ethanol from 6 hpf until 48 hpf and stained with Alcian Blue at 5 dpf to visualize craniofacial cartilage (Fig. 8). The exposure dose and duration were selected based on a previous study that demonstrated their minimal effects on embryogenesis in wildtype embryos (McCarthy et al., 2013). As expected, a small proportion of untreated *alx1* larvae developed with hypoplastic ANC (Fig. 8A-D). Notably, the penetrance of ANC defects increased significantly in ethanol-exposed *alx1* larva (Fig. 2D, *P*=0.024), while wildtype ANC formation was not significantly affected by ethanol exposure (Fig. 2D, *P*=0.24).

*alx1^uw2016^*, but not wildtype, embryos exhibited dysmorphic eyes at 5 dpf (Fig. 8E,F). Ethanol exposure increased the penetrance of this phenotype in *alx1* larvae, and caused it to emerge in wildtype larvae, albeit at low penetrance (Fig. 8G). To assess the effect of ethanol exposure on post-embryonic ocular development, ethanol-treated and untreated control *alx1^uw2016^* siblings were raised to adulthood and evaluated for ocular defects at 2 months of age. Ocular defects similar to those documented in Fig. 1 were observed in both groups (Fig. 8H-J). Ethanol-treated *alx1^uw2016^* adults exhibited a higher penetrance of ocular defects than wildtype siblings, but this difference did not rise to significance (*P*=0.10, Fig. 8J). Together, these data support a novel role for *alx1* in conferring robustness to ethanol toxicity on both craniofacial and ocular lineages. Collectively, our findings support crucial functions for *alx1* and *alx3* in the zebrafish ANC and in several ocular lineages and identify a robust set of candidate molecular mechanisms that underlie these important functions.

## DISCUSSION

Here, we show a redundant genetic requirement for zebrafish *alx1* and *alx3* in several midface structures derived from the aCNC: the ANC, the ocular anterior segment, and ocular blood vessels. We identify a protective role for zebrafish *alx1* in ocular and facial embryonic lineages against ethanol toxicity, which, together with the evidence from transcriptome analysis of *alx1;alx3* mutants, supports a function for *alx1* and *alx3* in regulating oxidative stress response and ribosome biogenesis. These findings extend and complement our previous human iPSC studies (Pini et al., 2020) and validate the first animal model of ALX-linked FND with ocular involvement, crucial for understanding conserved roles for *ALX* genes during vertebrate development.

### Complex roles of *alx* genes during development

Loss of *alx1* function has distinct consequences in different vertebrate species. Humans homozygous for *ALX1* mutations develop with fully penetrant, severe FND and extreme microopthalmia (Uz et al., 2010; Pini et al., 2020), as do *alx1*-mutant cats (Lyons et al., 2016). In contrast, mouse *alx1* mutants exhibit an earlier and more profound deficit, failure of cranial neural tube closure (Zhao et al., 1996), while zebrafish *alx1* mutants develop with variably penetrant, mild craniofacial phenotypes (Pini et al., 2020). Thus, loss of *ALX1* function always affects midface development in these vertebrates, but with varying severity and penetrance. These differences may reflect evolutionary divergence in functional specializations of individual *ALX* genes, common after gene duplication. Comparative genomic studies reveal substantial variability among chordates in the number of *alx* paralogs, ranging from one in hemichordates to four in zebrafish, likely a result of gene duplication events (Khor and Ettensohn, 2020; Lamichhaney et al., 2015; McGonnell et al., 2011). This variability is thought to have driven emergence of skeletogenesis strategies during chordate evolution (Khor and Ettensohn, 2020). Allelic variation at the *alx1* locus is associated with diversity in beak morphologies in Darwin's finch species, thus contributing to rapid evolution (Lamichhaney et al., 2015).

Species-specific differences in *alx1* gene functions may also reflect divergence in genetic compensation strategies employed by these organisms. In mouse, *alx4* compensates for loss of *alx3* since *alx3;alx4* double mutants develop with severe orofacial clefts and single mutants do not (Beverdam et al., 2001; Lakhwani et al., 2010). In zebrafish, single *alx4a* and *alx4b* mutants develop normally, while *alx3* mutants exhibit medial ANC defects (Mitchell et al., 2021). Notably, while Mitchell et al. did not observe craniofacial phenotypes in *alx1* mutants (Mitchell et al., 2021), we showed that *alx1* mutants develop with partially penetrant ANC defects (Pini et al., 2020). This discrepancy may be due to genetic background variation, which implies the existence of genetic modifiers of *alx* function that will be important to examine in future studies.

Here we show that genetic removal of both *alx1* and *alx3* functions significantly increases the penetrance and severity of craniofacial defects compared to *alx1* (Pini et al., 2020) and to *alx3* single mutants (Mitchell et al., 2021). It is important to note that we did not examine the phenotype of *alx3* single mutants in our genetic background, leaving open the possibility that alx3 interacts strongly with genetic modifiers and that the deficits we report here are due entirely to *alx3* loss-of-function. However, the absence of defects in *alx3* crispants (Fig. S6) argues strongly against this possibility. It is more likely that the relative severity of *alx1;alx3* mutant phenotypes reflect functional compensation between *alx1* and *alx3,* potentially through the mechanism of transcriptional adaptation triggered by nonsense-mediated RNA decay (El-Brolosy et al., 2019; Jakutis and Stainier, 2021; Kontarakis and Stainier, 2020; Sztal and Stainier, 2020). This mechanism may also explain why *ALX3* does not functionally compensate for the loss of *ALX1* function in human *ALX1-*linked *FND*, since the disease-linked missense mutations in *ALX1* are less likely to trigger nonsense-mediated RNA decay. Collectively, the *alx* gene family offers an excellent paradigm for asking how genetic compensation is employed by different organisms.

### How does *alx* function in the developing eye?

*alx* genes are robustly expressed in neural-crest-derived periocular mesenchyme (Dee et al., 2013; Sedykh et al., 2017) that gives rise to the supporting tissues of the eye, including the anterior segment and ocular vascular pericytes (Gage et al., 2005; Jo et al., 2013; Johnston et al., 1979; Le Lievre and Le Douarin, 1975; Trost et al., 2013), but not in retinal lineages. This suggests that the retinal deficits in *ALX1* homozygotes are secondary to aberrant formation of supporting structures, and our study provides the first experimental support for this hypothesis. Ocular deficits in post-embryonic *alx1* and *alx1;alx3* zebrafish appear to be restricted to vascular and anterior segment-derived structures, including the lens, while retinal function, assessed by visually-triggered behaviors, is not affected by loss of *alx1* and *alx3* (Fig. 4). In contrast, human newborns homozygous for the *ALX1* loss-of-function allele present with reduced or absent retina (Fig. 1), and morpholino-mediated *alx1* knockdown in zebrafish results in retinal malformations and anterior segment defects, which are likely secondary to retinal defects (Dee et al., 2013). Functional compensation by the remaining zebrafish paralogs, *alx4a* and *alx4b*, is a plausible explanation for this discrepancy that can be tested by generating triple and quadruple *alx* mutants.

It is perhaps not surprising that defects in ocular vasculature have not been observed in *ALX1*-linked FND patients, since hyaloid vasculature is transient in mammals and regresses prior to birth (Selvam et al., 2018). Our demonstration of a novel role for *alx* function in hyaloid vessel formation suggests the possibility of a transient defect during early ocular vascular development of FND patients, which could in turn impact retinal development by disrupting endothelial-to-retina signaling recently shown to be required for retinal neurogenesis in zebrafish (Dhakal et al., 2021, 2015). Impaired ocular blood flow may also affect retinal development in mammals which, unlike zebrafish, rely on ocular blood flow for early retinal gas exchange (Dhakal et al., 2015; Pelster and Burggren, 1996).

Neural crest that populates the anterior segment is highly heterogenous; its influence on retinal development during embryogenesis is poorly understood and likely to vary across vertebrates (Takamiya et al., 2020; Van Der Meulen et al., 2020; Williams and Bohnsack, 2015, 2020). It is also likely that the ocular anterior segment offers differing degrees of protection to the developing retina in humans versus lower vertebrates. However, congenital anterior segment dysgenesis (ASD) (Evans and Gage, 2005; Hendee et al., 2018; Portal et al., 2019; Reis and Semina, 2011) is associated with postnatal death of retinal ganglion cells, but not with prenatal retinal defects (Mao et al., 2011, 2017; Martino et al., 2016).

*alx1* is also expressed in mouse and chick sclera, a largely overlooked ocular lineage that derives from neural crest and forms adjacent to the retinal pigment epithelium (Thompson et al., 2010). The sclera produces cartilage in birds, while mouse embryos initiate the program but stop short of cartilage production in the sclera (Thompson et al., 2010). Notably, microcalcification of the sclerae and cystic lesions in the anterior chamber have been reported in a patient with splice-site mutation in *ALX1* (Uz et al., 2010), lending support for the hypothesis that a role for *alx* genes in these ocular lineages has been conserved and can be effectively modeled in animals.

### Fetal alcohol syndrome and *ALX* function

Fetal alcohol spectrum disorder (FASD) is a common condition estimated to affect 5% of live births in the US. It presents with disabling neurological defects and is frequently associated with facial and ocular deficits that contribute to the heavy health burden of FASD (Brennan and Giles, 2014; Gupta et al., 2016; Lovely et al., 2017; Lovely, 2020). Emerging evidence indicates that fetal genotype strongly influences the outcome of prenatal ethanol exposure, since many children exposed to high levels of ethanol *in utero* do not develop FASD (Lovely, 2020). Naturally occurring alleles that confer robustness or sensitivity to alcohol toxicity can be identified epidemiologically, but model organism studies are essential for sustained progress toward a complete understanding of gene-environment interactions that contribute to FASD (de la Morena-Barrio et al., 2018; Eberhart and Parnell, 2016; Kaminen-Ahola, 2020; Lovely et al., 2017).

Model organism studies have shown that neural crest-derived lineages are particularly vulnerable to ethanol toxicity (Smith et al., 2014). Neural crest cells give rise to most craniofacial structures, including the face and the anterior segment of the eyes, and indirectly influence formation of the retina and early brain development. In other words, neural crest vulnerability to ethanol toxicity may account for the majority of defects observed in FASD.

Studies in animal models have offered several mechanistic hypotheses for fetal ethanol toxicity: an increase in reactive oxygen species (ROS), resulting in increased oxidative stress (Brocardo et al., 2011; Dong et al., 2010; Lovely et al., 2017; Wang et al., 2015; Wentzel and Eriksson, 2011), disruption of ribosome biogenesis (Garic et al., 2014) interference with retinoic acid signaling (Williams and Bohnsack, 2019) and apoptosis (McCarthy et al., 2013). Here we show increased vulnerability of facial and ocular lineages to ethanol toxicity in the absence of functional *alx1* in zebrafish, and a requirement for *alx1* and *alx3* function in regulating two of the pathways implicated in FASD: ribosome biogenesis (Fig. 5) and oxidative stress response (Fig. 7). Moreover, mouse *Alx3* has been shown to regulate oxidative stress response through activating transcription of *Foxo1*, a key regulator of the antioxidant pathway (Garcia-Sanz et al., 2017). Notably, the penetrance of ocular defects is lower than the penetrance of craniofacial defects in ethanol treated *alx1* mutant embryos, consistent with reports that anterior segment lineages are less susceptible to oxidative stress than craniofacial neural crest (Eason et al., 2017). Our study is the first to demonstrate a protective function for *alx1* in the zebrafish model. Real-time *in vivo* imaging with lineage-specific transgenic reporters, together with pharmacological manipulation of the oxidative stress response pathway, will allow us to rigorously test these functions in future studies. Ultimately, animal models studies will be essential for elucidating the complex, poorly understood processes that contribute to frontonasal development (Farlie et al., 2016).

## MATERIALS AND METHODS

### Zebrafish strains and embryo manipulation

Adult zebrafish were maintained according to established methods (Westerfield, 1993). All experimental protocols using zebrafish, including adult euthanasia, were approved by the University of Wisconsin Animal Care and Use Committee and carried out in accordance with the institutional animal care protocols. Embryos were obtained from natural mating and staged according to Kimmel (Kimmel et al., 1995). The following mutant strains of zebrafish were used: *alx1^uw2016^* (Pini et al., 2020) and *alx1uw2016;alx3uw2113/+* generated in the course of this study (see below) and *pappaa^p170^* (Wolman et al., 2015). Embryos were exposed to 0.5% ethanol diluted in E3 starting at 6 hpf. At 2 dpf, embryos were transferred to fresh E3 and raised until 5 dpf to score for craniofacial defects, or until 2 months to score for ocular defects.

### CRISPR/Cas9 mutagenesis and mutant allele identification

*alx1^uw2016^;alx3^uw2113^*/+ zebrafish *were* obtained through *alx3*-targeted CRISPR/Cas9 mutagenesis in the *alx1^uw2016^* mutant background. Design of *alx3* CRISPR site 5′-GGAGTCCCCAGTCAAGCCGT-3′ in exon 2 and mutagenesis were carried out as previously described (Sedykh et al., 2016, 2017), and the recovered mutant alleles were initially characterized by sequencing PCR-amplified genomic DNA. To detect the presence of *alx1^uw2016^* and *alx3^uw2113^* mutant alleles, the relevant region was PCR amplified from genomic DNA extracted from adult tail clips or individual embryos, and subjected to PCR with the following primers: *alx1* forward: 5′-CGTGACTTACTGCGCTCCTA-3′ (Pini et al., 2020), *alx1* reverse: 5′-CGAGTTCGTCGAGGTCTGTT-3′ (Pini et al., 2020), *alx3* forward: 5′-CTATCCCGCTCTGGACTCAG-3′, *alx3* reverse: 5′-TCCTCCAGTTGAAAGGTGCT-3′. To characterize mutant alleles, *alx3* PCR fragments were subcloned via TA cloning into pGEMT-Easy (Promega) and sequenced. To determine *pappaa^p170^* genotype, DNA was extracted from individual larvae and subjected to PCR with the following primers: *pappaa^p170^* forward: 5′-CACTCTGGAGCCTCCAGCTTGCGGT-3′ and *pappaa^p170^* reverse: 5′-TTGCTGACGTTGTGTACG-3′ (Wolman et al., 2015). The PCR product was digested with Mse1 (New England Biolabs), cleaving the mutant allele and producing a 245 bp fragment distinguishable from the 271 bp wildtype allele. Subsequently, metaphor (Lonza) gel electrophoresis was used to genotype individual embryos and adult fish.

### Immunohistochemistry

*alx1;alx3/+* incross progeny were fixed at 2 dpf in 4% paraformaldehyde (PFA)/PBS overnight. Their trunk/tail portions were reserved for genotyping and the heads were exposed to 1% KOH/6% H_2_O_2_ to remove pigmentation and washed with PBST-X (1X PBS with 0.5% TritonX-100), followed by a 5-min proteinase-K (1:5000) treatment and 30-min 4% PFA/PBS post-fix at room temperature. Embryos were blocked with PBSTD-X (1X PBST, 1% DMSO, 10% BSA, 10% goat serum) for 2 h, at room temperature, incubated with mouse-anti-zn5 primary antibody (Zebrafish International Resource Center A28175, 1:100) and then Alexa-488-conjugated goat anti-mouse secondary antibody (Invitrogen AB_10013770, 1:500). Nuclei were labeled with DAPI (Molecular Probes; 1:5000). For imaging, embryos were mounted in VectaShield (Vector laboratories) and imaged on an Olympus IX81 inverted confocal microscope with the Fluoview 1000 confocal package, using a 60x water immersion objective (NA 1.10).

### *In situ* hybridization and Alcian Blue staining

*In situ* hybridization was carried out as previously described (Gillhouse et al., 2004) using a probe against *col2a1a* (Yan et al., 1995). After whole-mount *in situ* hybridization, embryos were mounted in 100% glycerol and imaged on a Leica MZ FLIII stereo microscope equipped with Leica DFC310 FX camera and LAS v4.0 software. For cartilage staining, larvae were fixed at 5 dpf in 4% PFA/PBS overnight and stained with Alcian Blue according to Kimmel et al. (1998). Stained larvae were further dissected using forceps to isolate jaw cartilage and anterior neurocranium, and imaged using a NIKON eclipse E600 microscope equipped with Q Imaging QIclick 1.4MP CCD Monochrome Microscope camera and NIS-elements software.

### *O*-dianisidine staining and alkaline phosphatase staining

Staining erythrocytes with *o*-dianisidine was carried out as previously described (Paffett-Lugassy and Zon, 2005). To visualize the intraocular red blood cell population, retinal pigment was removed by exposure to 1% KOH/6% H_2_O_2_ and embryos were mounted in 100% glycerol and imaged on Leica MZ FLIII stereo microscopes equipped with Leica DFC310 FX camera and LAS v4.0 software. Endogenous alkaline phosphatase activity in the anterior segment and hyaloid vessels was detected as previously described in (Alvarez et al., 2007; Goehlert et al., 1981; Lessell and Kuwabara, 1964; Vorontsova et al., 2019). Briefly, zebrafish larvae were fixed at 5 dpf in 4% PFA/PBS overnight and eyes were pierced with a sharpened tungsten microneedle and forceps prior to staining. After staining, the retina and sclera were removed with tungsten needles to release the lens, and dissected lenses were imaged using NIKON eclipse E600 microscope equipped with Q Imaging QIclick 1.4MP CCD Monochrome Microscope camera and NIS-elements software.

### Adult ocular histology

Adult *alx1^uw2016^* zebrafish were euthanized and preserved in 4% paraformaldehyde fixative. Each fish was photographed on both sides to document eye morphology, and the entire body was immersed for 30 min in a solution of 12% hydrochloric acid (Decal II, Surgipath) to decalcify the bone. Transverse sections were made through the head. The resulting head blocks containing the eyes were submitted for paraffin embedding and step sectioned at 100 μm to sample beyond the midway of the globes. Extra slides were saved at each step. The selected sections were stained using standard protocol with hematoxylin and eosin. Rough measurements were made of each globe in the axial plane and in the vertical plane using the histology slide.

### Lineage tracing and fluorescent imaging

Embryos derived from *alx1^uw2016^*; *Tg(sox10:kaede)* were injected with ∼2 nl of 3 mM gRNA:Cas9 RNP complex at the 1-cell stage, as described in a previous study (Hoshijima et al., 2019) to generate *alx3* crispants. Injected embryos were raised until the 20-somite stage, whereupon they were dechorionated, selected for strong fluorescent expression, and mounted in low melting agarose containing 0.013% tricaine. Crispants were photoconverted using the 405 nm UV laser on a Leica scanning confocal microscope, with regional selectivity accomplished by using region of interest (ROI) features. Selected cells were exposed to the photoconverting laser at full power for 3-5 min, depending on the size of the region, or until red kaede was observed. Crispants were imaged to record initial position of the photoconverted cells and raised until 4 dpf, when they were once again mounted in low melting agarose with tricaine and imaged on the confocal microscope. For fluorescent imaging of the ANC and Meckel's cartilage, both uninjected siblings and crispants at 4 dpf with different genotypes on a *Tg(sox10:kaede)* background were dissected and flat-mounted for imaging.

### RNA-Seq transcriptome analysis

For the *alx1;alx3* mutant dataset, embryos produced from a cross between *alx1;alx3/+* parents were flash-frozen individually at 5 dpf, and RNA was prepared using the RNeasy Plus Mini Kit (Qiagen 74134) and a protocol adapted from (de Jong et al., 2010). Genomic DNA contamination was removed using the DNase Max Kit (Qiagen 15200-50). cDNA libraries were prepared using the TruSeq stranded mRNA library preparation protocol with poly-A selection and sequenced on the Illumina HiSeq 2500. Prior to analysis, each sample was genotyped from raw sequencing data alignments using the Integrative Genomics Viewer (Robinson et al., 2011).

For the *alx1;alx3*CR dataset, embryos derived from *alx1uw2016;Tg(sox10:kaede)* parents were injected with *alx3* sgRNA;Cas9 RNP complex, raised until 3 dpf and screened under fluorescence to select those exhibiting malformed ANC. Embryos derived from *Tg(sox10:kaede),* fertilized within 2 h and raised under the same conditions were used as age-matched controls. Twenty embryos were combined into each biological replicate, and six replicates for each genotype were collected in two independent experiments. RNA was prepared using the RNeasy Plus Mini Kit (Qiagen 74134) and RNA quality was assessed by Agilent High Sensitivity RNA ScreenTape (Agilent, 5067-5580). cDNA libraries were prepared using the NEBNext^®^ Ultra™ II Directional RNA Library kit (NEB, E7765S) with NEBNext^®^ Poly(A) mRNA Magnetic Isolation Module (E7490S) and sequenced (75 bp paired-end) on the Illumina Nextseq 550.

Bioinformatic analysis of transcriptomic data adhere to recommended ENCODE guidelines and best practices for RNA-Seq (Encode Consortium, 2016). For the *alx1;alx3*-mutant RNA-seq dataset, alignment of adapter-trimmed (Jiang et al., 2014) (Skewer v0.1.123) 2×150 (paired-end; PE) bp strand-specific Illumina reads to the *Danio rerio* GRCz11genome (assembly accession GCF_000002035.6) was achieved with the Spliced Transcripts Alignment to a Reference (STAR v2.5.3a) software (Dobin et al., 2013), a splice-junction aware aligner. Expression estimation was performed with RSEM v1.3.0 (RNASeq by Expectation Maximization) (Li and Dewey, 2011). To test for differential gene expression among individual group contrasts, expected read counts obtained from RSEM were used as input into Deseq2 (Love et al., 2014). Pairwise contrasts between *alx1;alx3* mutants and age-matched *alx1^uw2016^* siblings were conducted to generate differentially expressed gene lists.

For the *alx1;alx3*CR RNA-seq dataset, two 75 bp (paired-end; PE) strand-specific Illumina Reads were trimmed to remove Illumina adapter sequences and low-quality sequence with Trimmomatic (version 0.36) (Bolger et al., 2014). Reads were aligned to danRer10 using STAR (version 2.7.1a) (Dobin et al., 2013) with custom built index based on Ensembl v91 gene annotations. Gene expression counts were extracted using featureCounts from the subread package (version 2.0) (Liao et al., 2014) using Ensembl v91 gene annotations. To test for differential gene expression among individual group contrasts, read counts obtained were used as input into Deseq2 (Love et al., 2014). Pairwise contrasts between CRISPants and age-matched wildtype were conducted to generate differentially expressed gene lists. Gene set enrichment was then determined by gene set enrichment analysis (GSEA) (Subramanian et al., 2005) using the Gene Ontology database (Ashburner et al., 2000; Gene Ontology Consortium, 2020).

### Behavior testing, video recording and analysis

Zebrafish larvae were raised at 29°C in E3, on a 14:10 h light/dark cycle starting at 1 dpf. On the day of behavioral testing (5 dpf), 60 mm-wide Petri dishes with 25 larvae/dish in 10 ml E3, were placed on a white light box (800 µW/cm^2^) for at least 1 h. Larvae where transferred to a 4×4 grid and illuminated from above with a mounted LED light (MCWHL5 6500K LED, powered by LEDD1B driver, Thorlabs), and from below with an infrared light source (IR Illuminator CM-IR200B, C&M Vision Technologies). In the grid, larvae were acclimated to the illuminated testing stage (85 µW/cm^2^) for 5 min before exposure to a series of 10 dark flash stimuli. The O-bend behavior was elicited using an automated behavioral platform in which the mounted LED light and the timing of dark flashes could be controlled (Burgess and Granato, 2007; Wolman et al., 2011). Stimuli were presented at 30 s interstimulus intervals, with each stimulus lasting for 1 s. We used Flote to analyze video images responses in an experimenter-independent, automated manner (Burgess and Granato, 2007). Flote tracks the position of individual larvae frame by frame and characterizes locomotor maneuvers according to predefined kinematic parameters that distinguish these maneuvers. After testing in the 4×4 grid, larvae were transferred to a 96-well plate and genotyped for *alx1*, *alx3*, or *pappaa^p170^ post-hoc.*

### Statistics

Statistical analyses, including calculation of means, SEM, and ANOVA, Fisher's exact test and the Chi-square test, were performed using GraphPad Prism software (GraphPad Software). *P*-values below 0.05 were considered statistically significant.

## Supplementary Material

Supplementary information
