# Supplementary material for: Zebrafish models of alx-linked frontonasal dysplasia reveal a role for Alx1 and Alx3 in the anterior segment and vasculature of the developing eye
Source: Biol Open. 2022 May 26;11(5):bio059189. doi: 10.1242/bio.059189 (PMC9167625; doi:10.1242/bio.059189)
Supplement: Supplementary information [file biolopen-11-059189-s1.pdf]

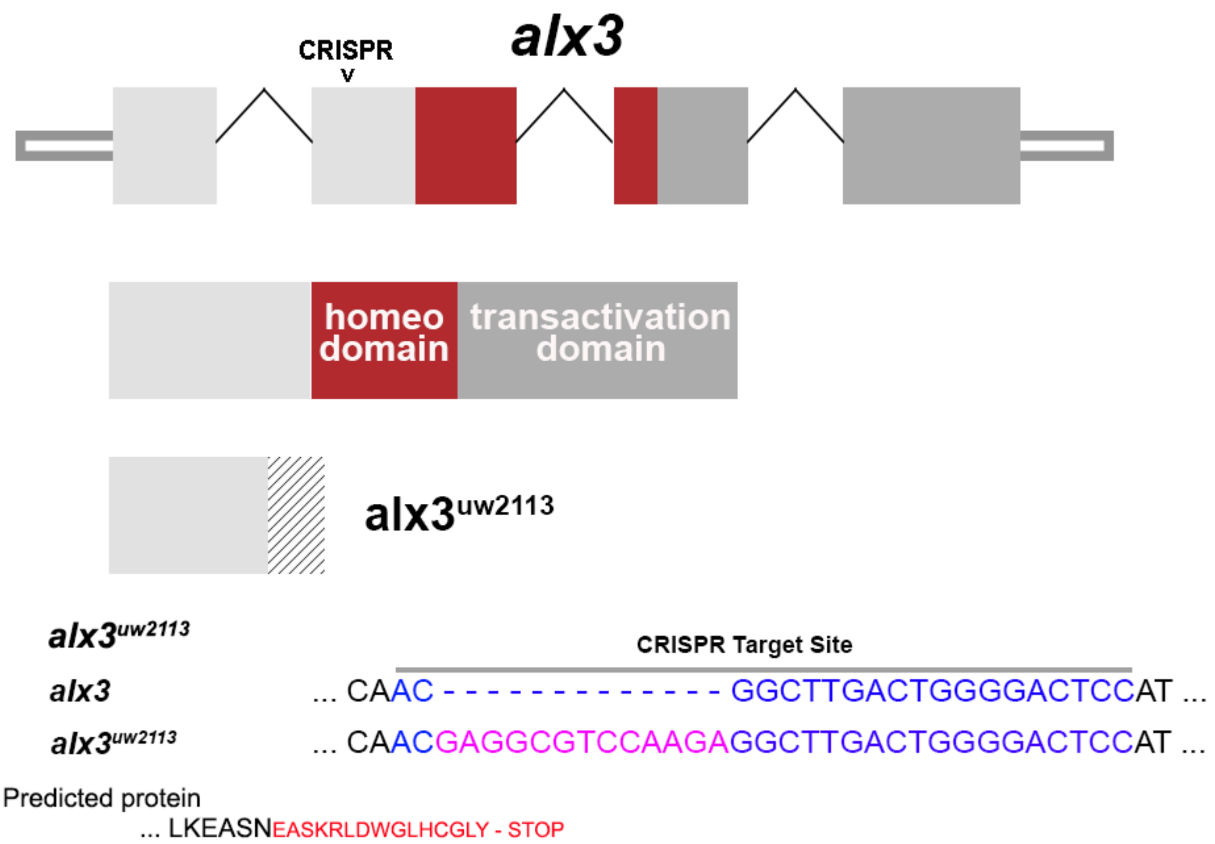

Fig. S1. Generation of mutant alleles at the zebrafish *alx3* locus. *alx3* mutations were induced by CRISPR/Cas9 mutagenesis targeted to the second exon of the alx3 locus. *alx3*^{uw2113} allele contains a net insertion of 13 nt (magenta letters) which is predicted to cause a frameshift and a premature stop (red letters).

Biology Open • Supplementary information

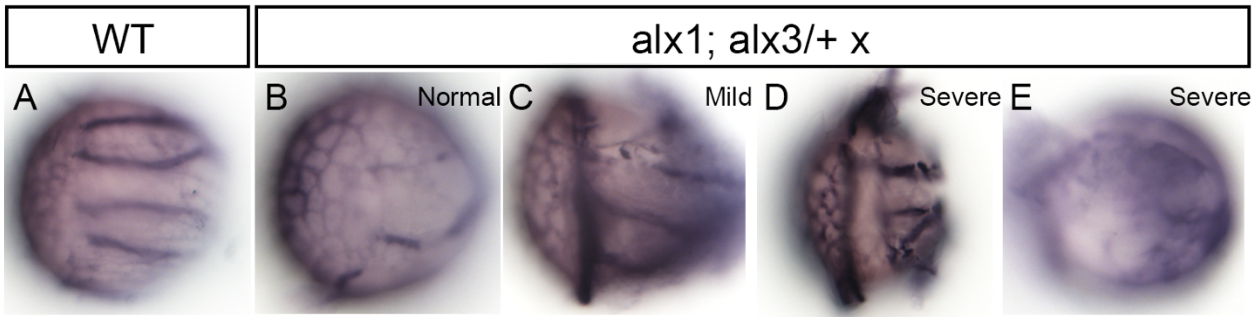

Fig. S2. *alx1* and *alx3* are required for hyaloid vascular patterning. Embryos derived from WT (A) or *alx1; alx3/+* (B – E) parents were first scored for the presence of misshapen eyes and stained for alkaline phosphatase to visualize hyaloid vasculature that surrounds the lens at 5 dpf. A: Normal hyaloid pattern in WT (6/6), B: Reduced hyaloid network formation in embryo with normally shaped eyes (5/6), C: Embryos with mildly misshapen eyes and disorganized hyaloid pattern (5/6). D-E: Embryos with severely misshapen eyes and disorganized (D; 2/5) or no hyaloid vasculature (E; 2/5 lens).

Biology Open • Supplementary information

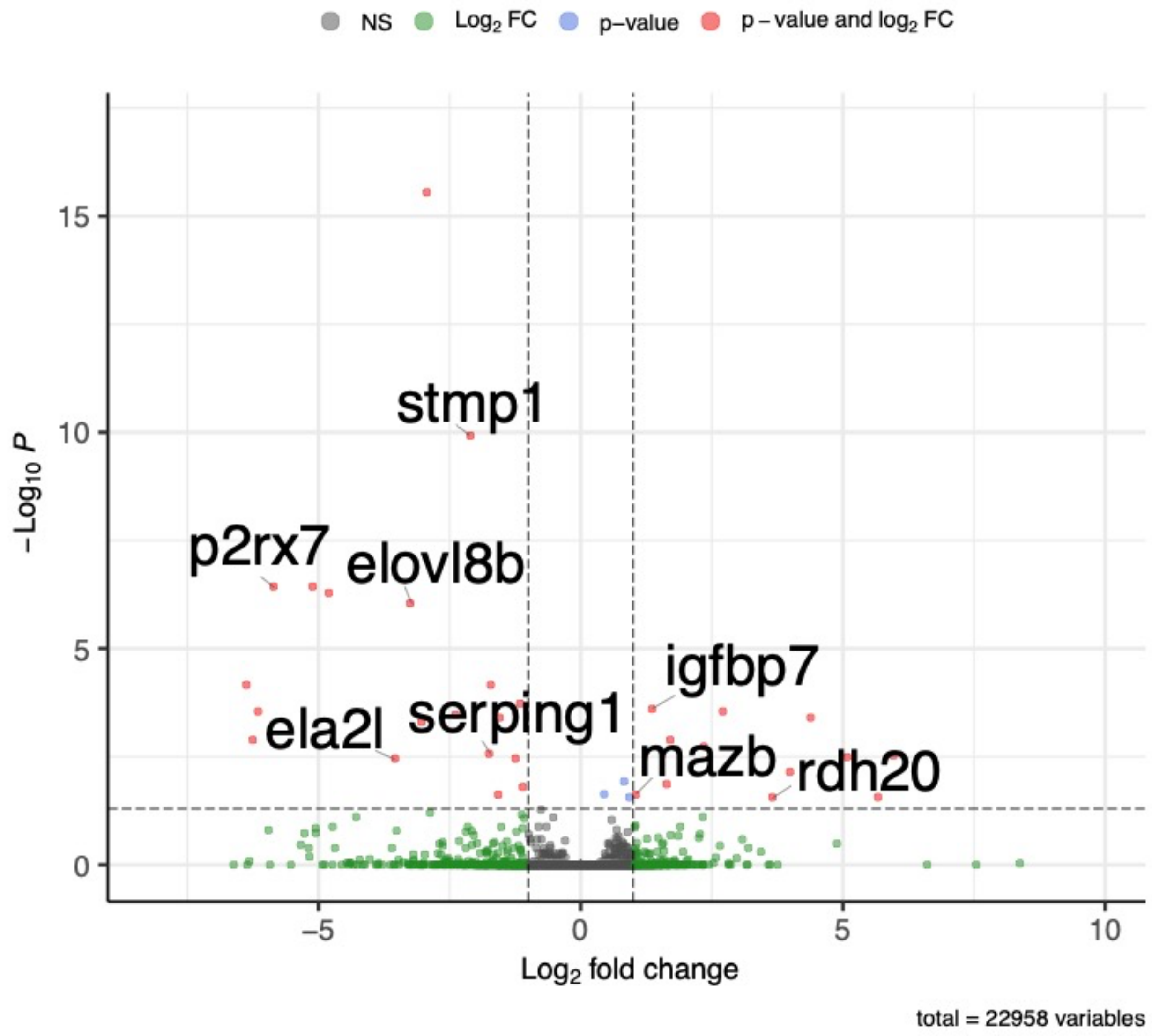

Fig. S3. Differentially expressed genes in alx1;alx3/+ mutants relative to wildtype siblings. A heat map (A) and volcano plot diagram (B) of differentially expressed genes in *alx1;alx3/+* mutants relative to wildtype siblings (see Figure 6 for details).

Biology Open • Supplementary information

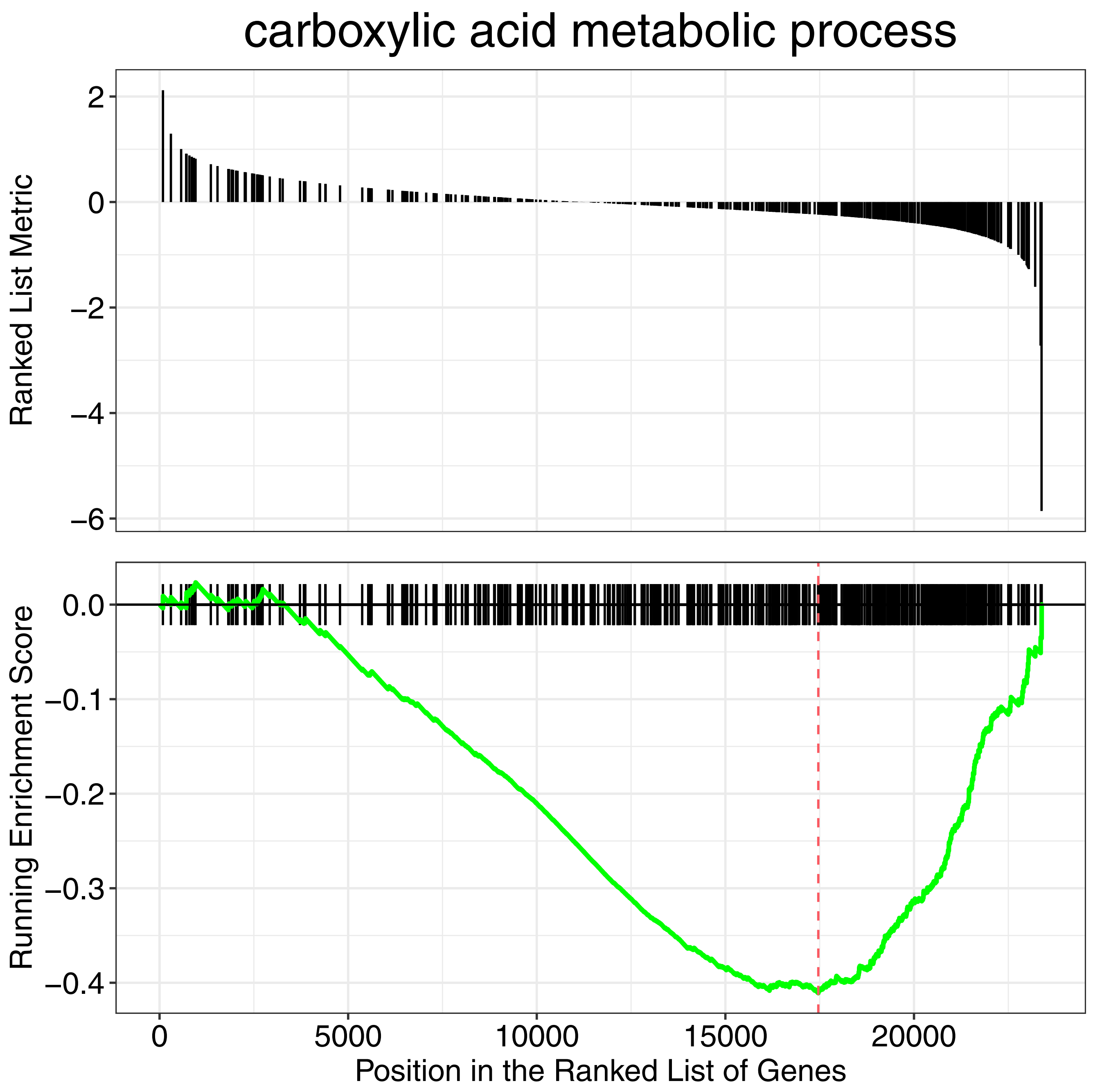

Biology Open • Supplementary information

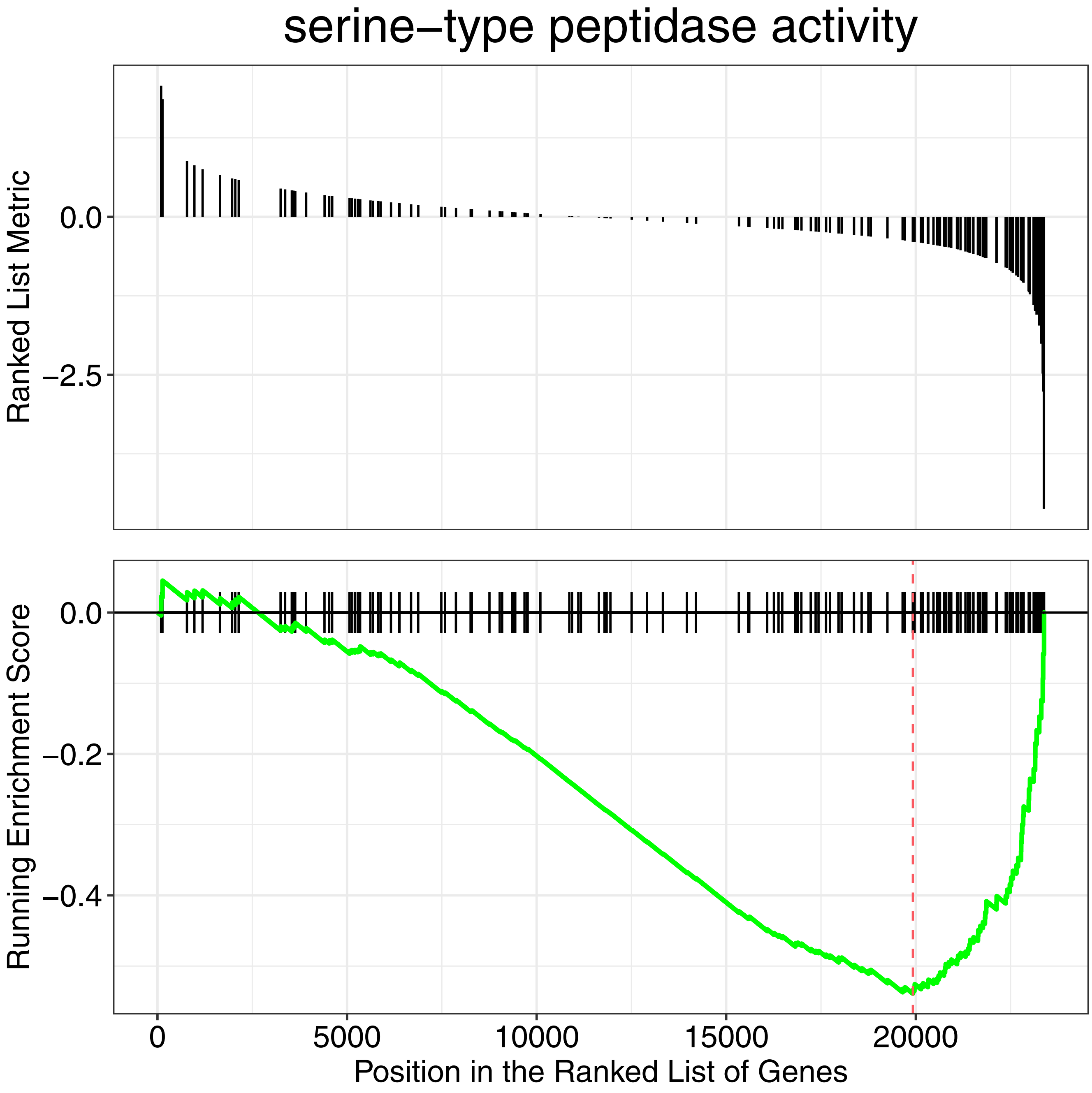

Biology Open • Supplementary information

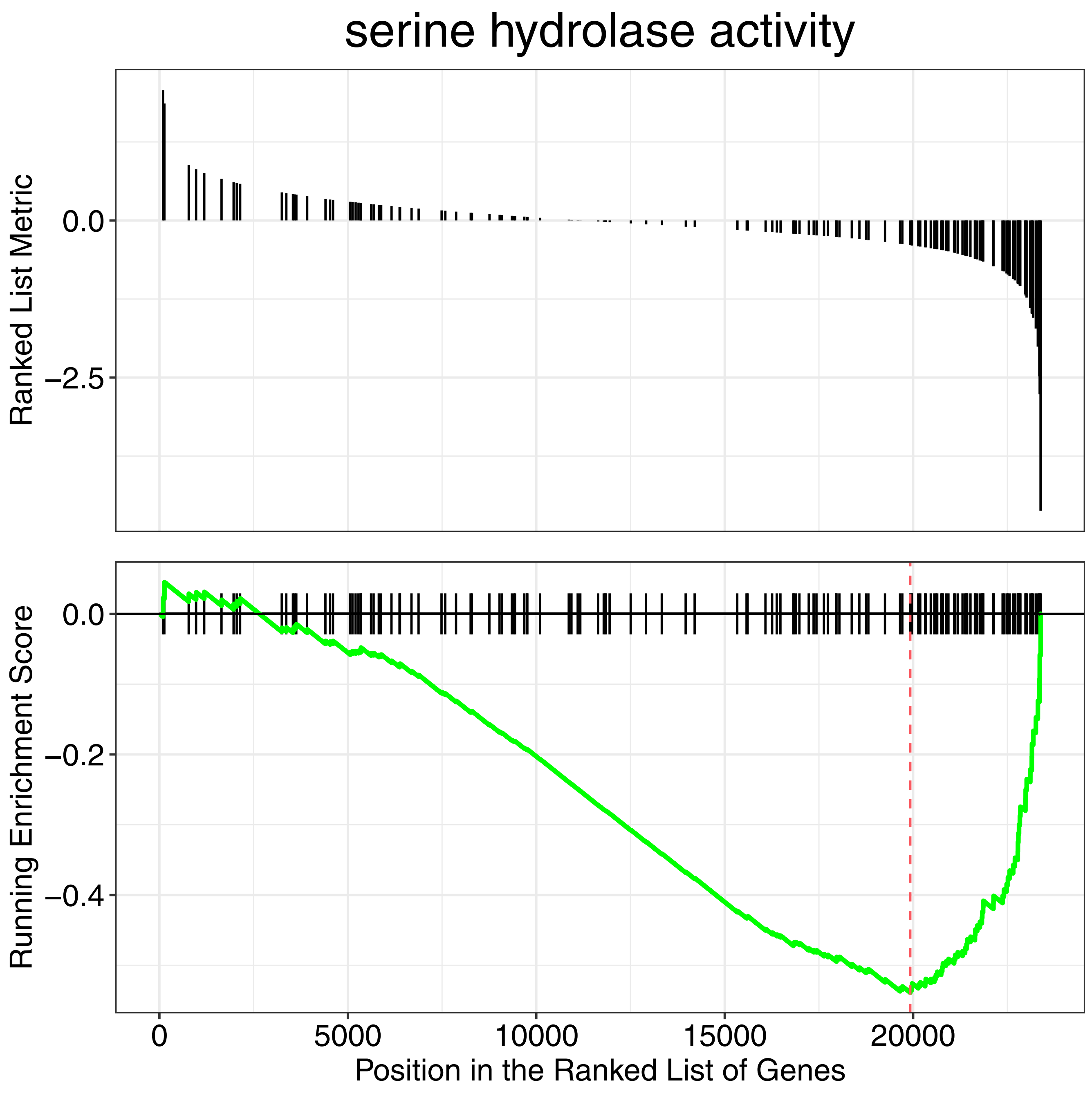

Biology Open • Supplementary information

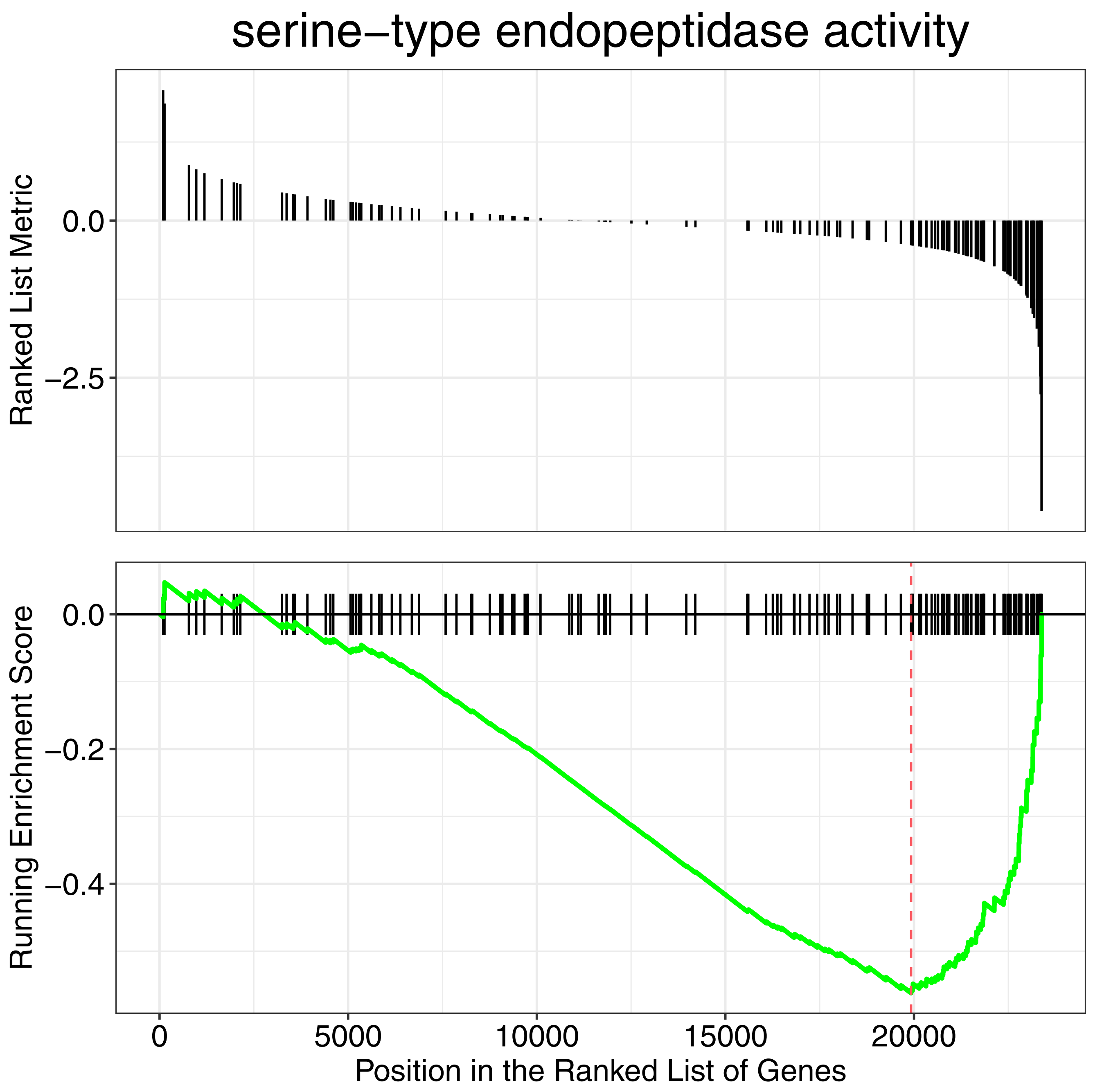

Biology Open • Supplementary information

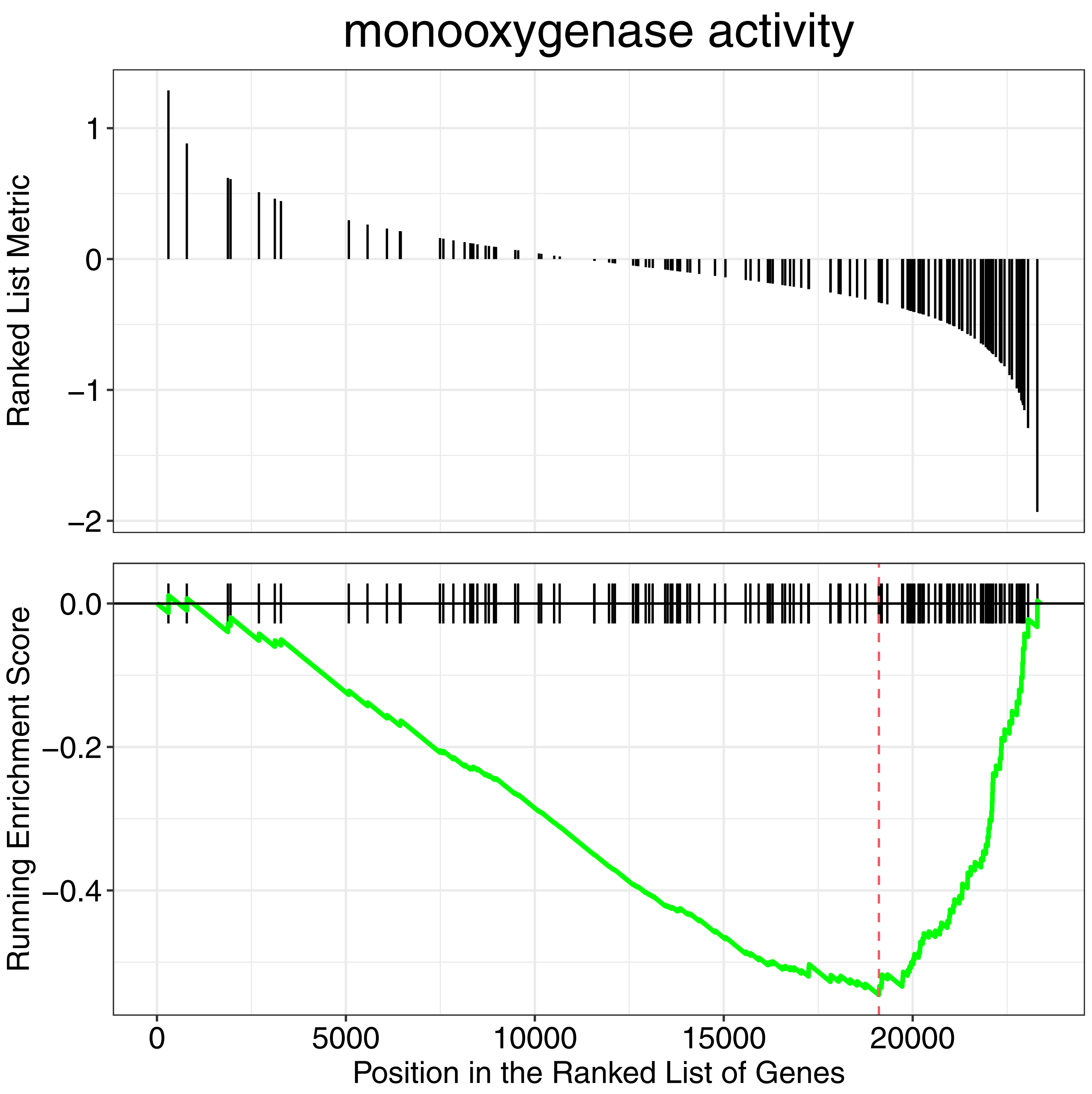

Biology Open • Supplementary information

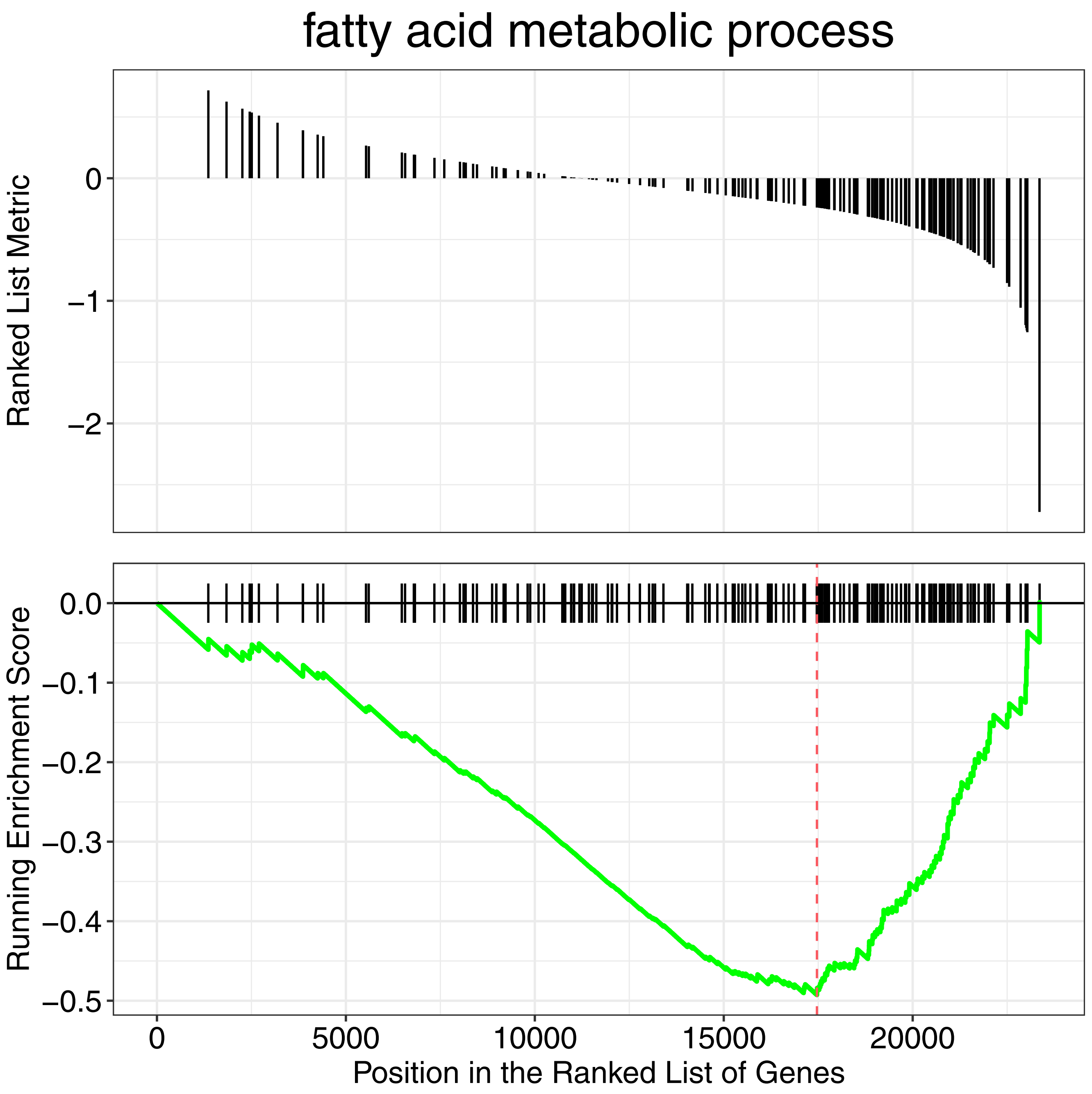

Biology Open • Supplementary information

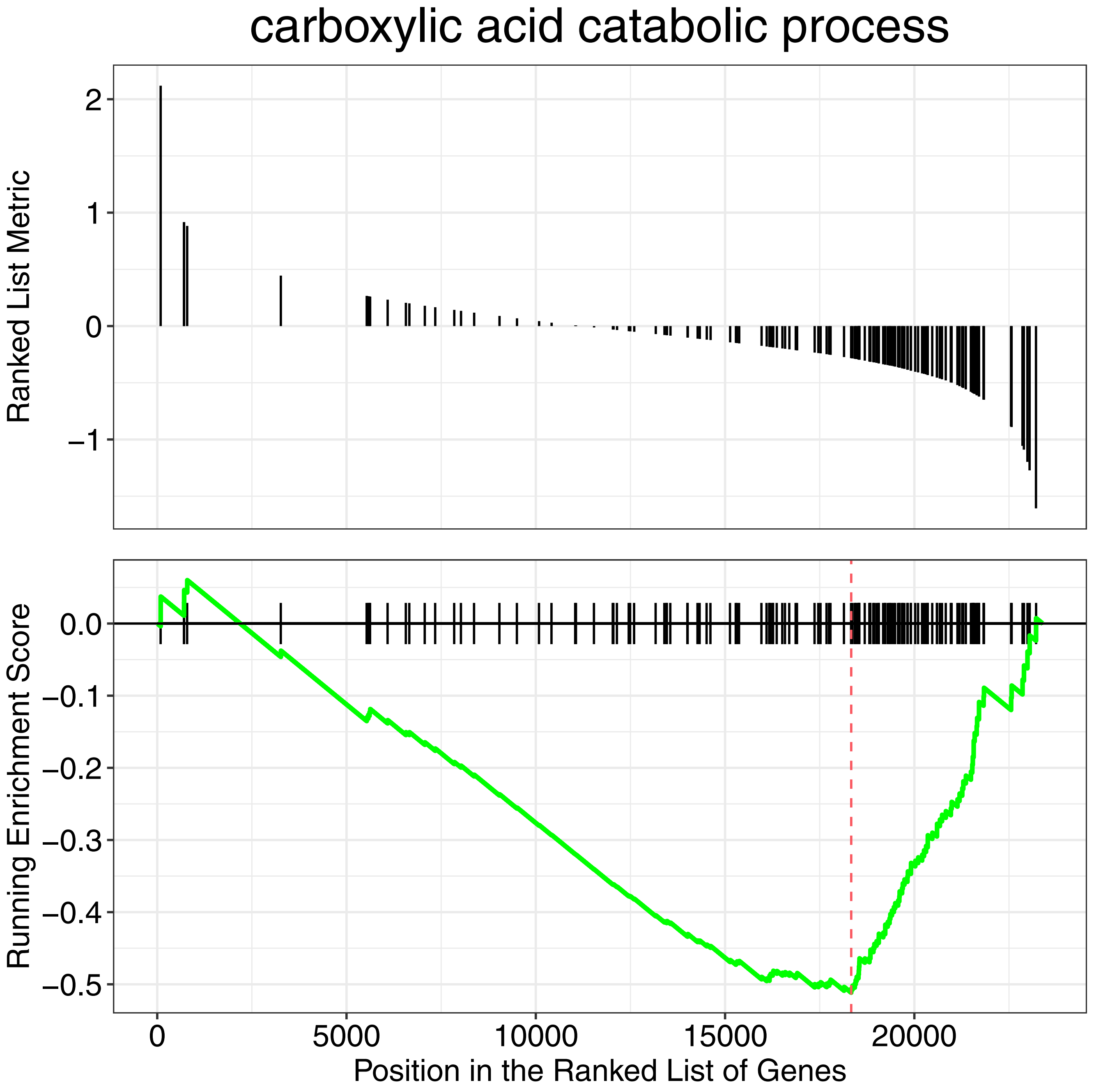

Biology Open • Supplementary information

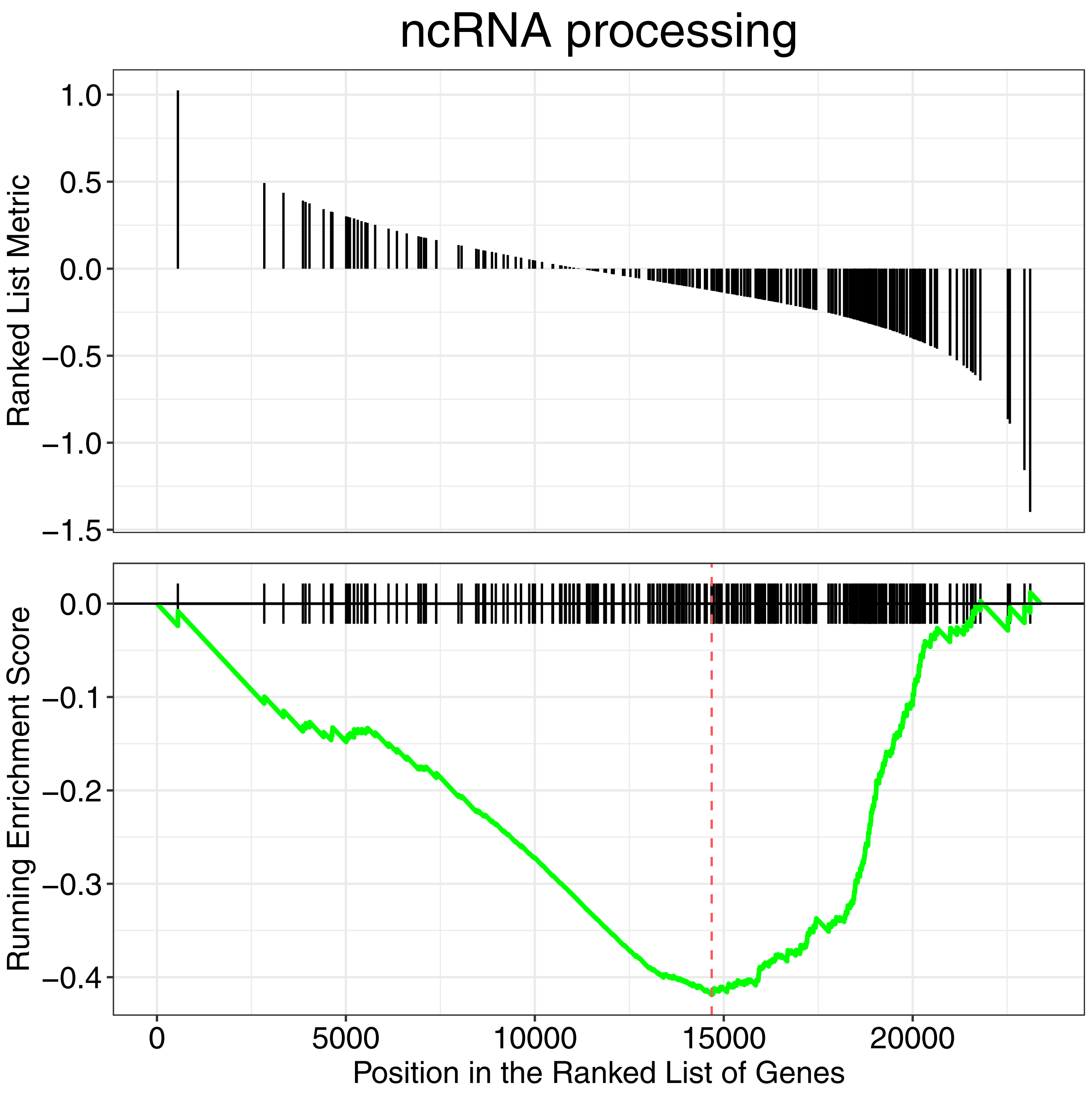

Biology Open • Supplementary information

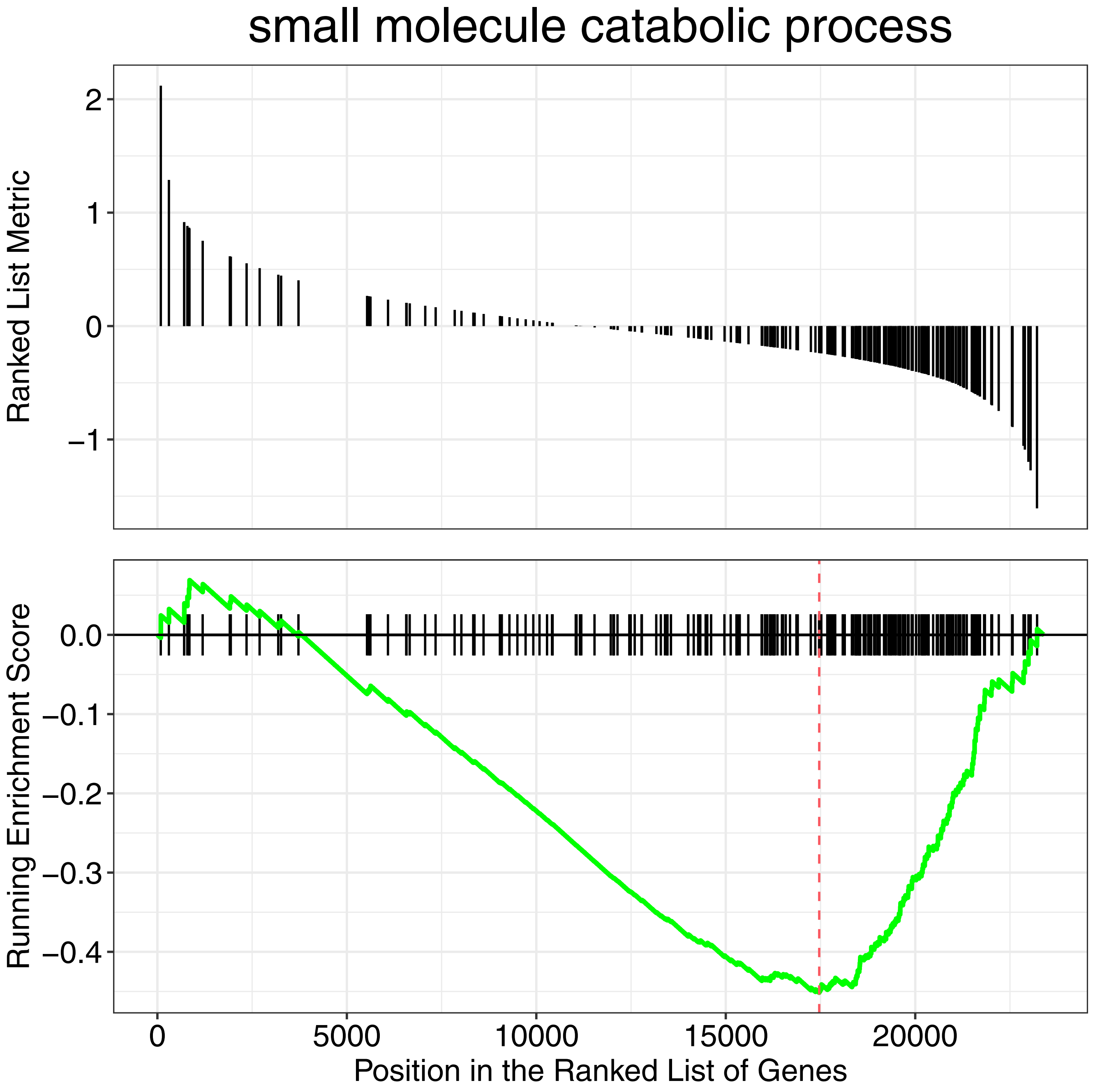

Biology Open • Supplementary information

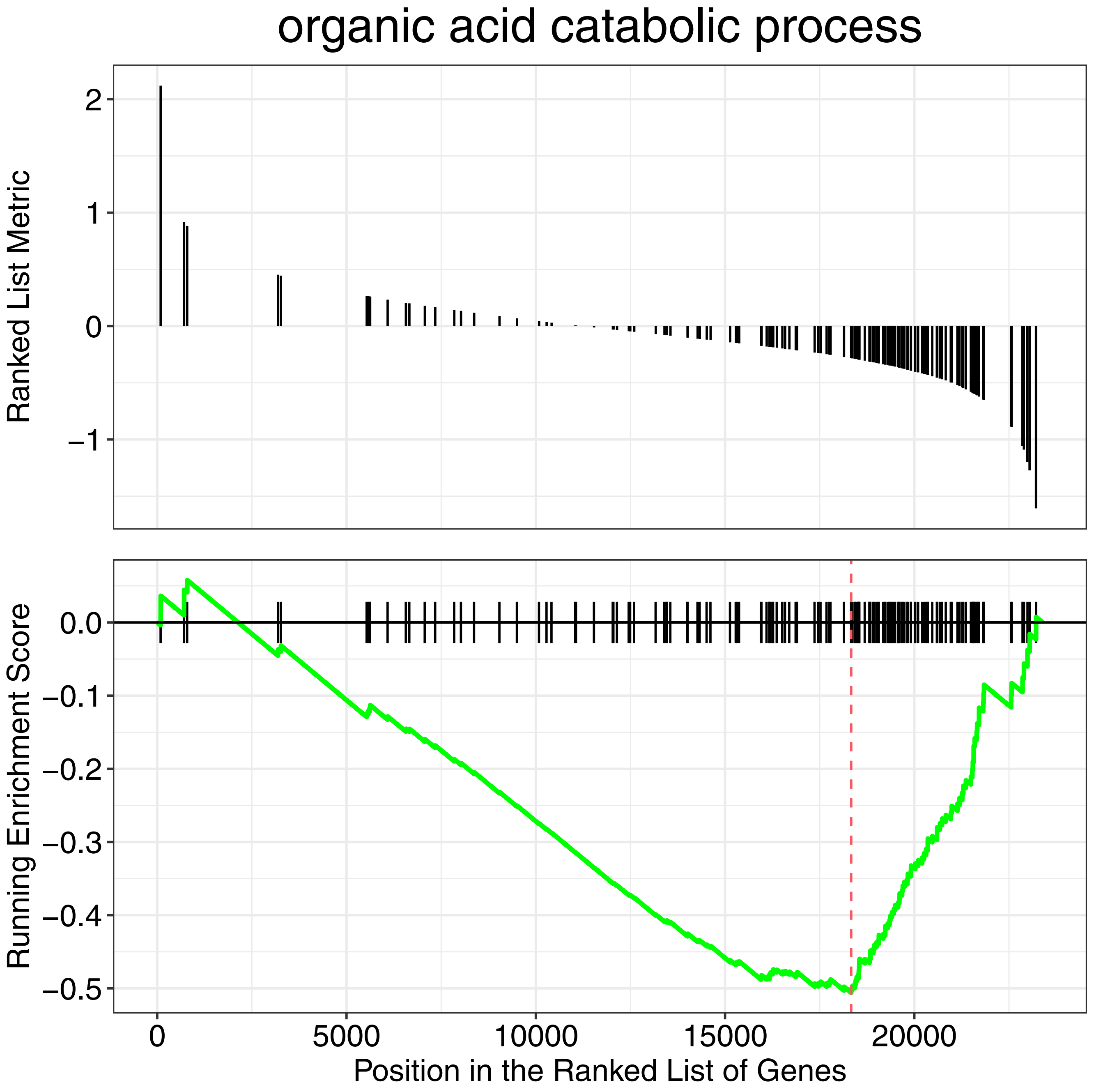

Biology Open • Supplementary information

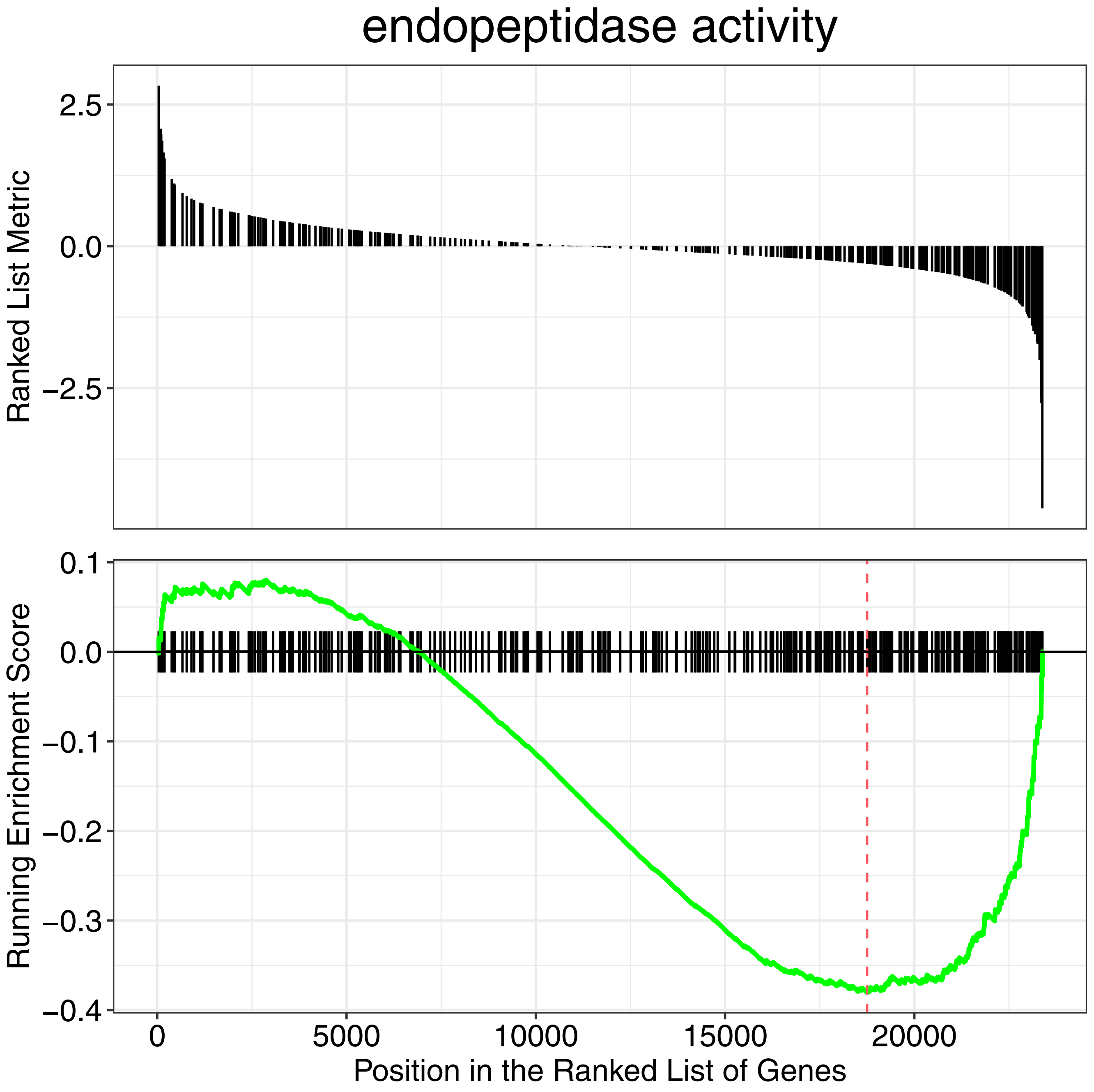

Biology Open • Supplementary information

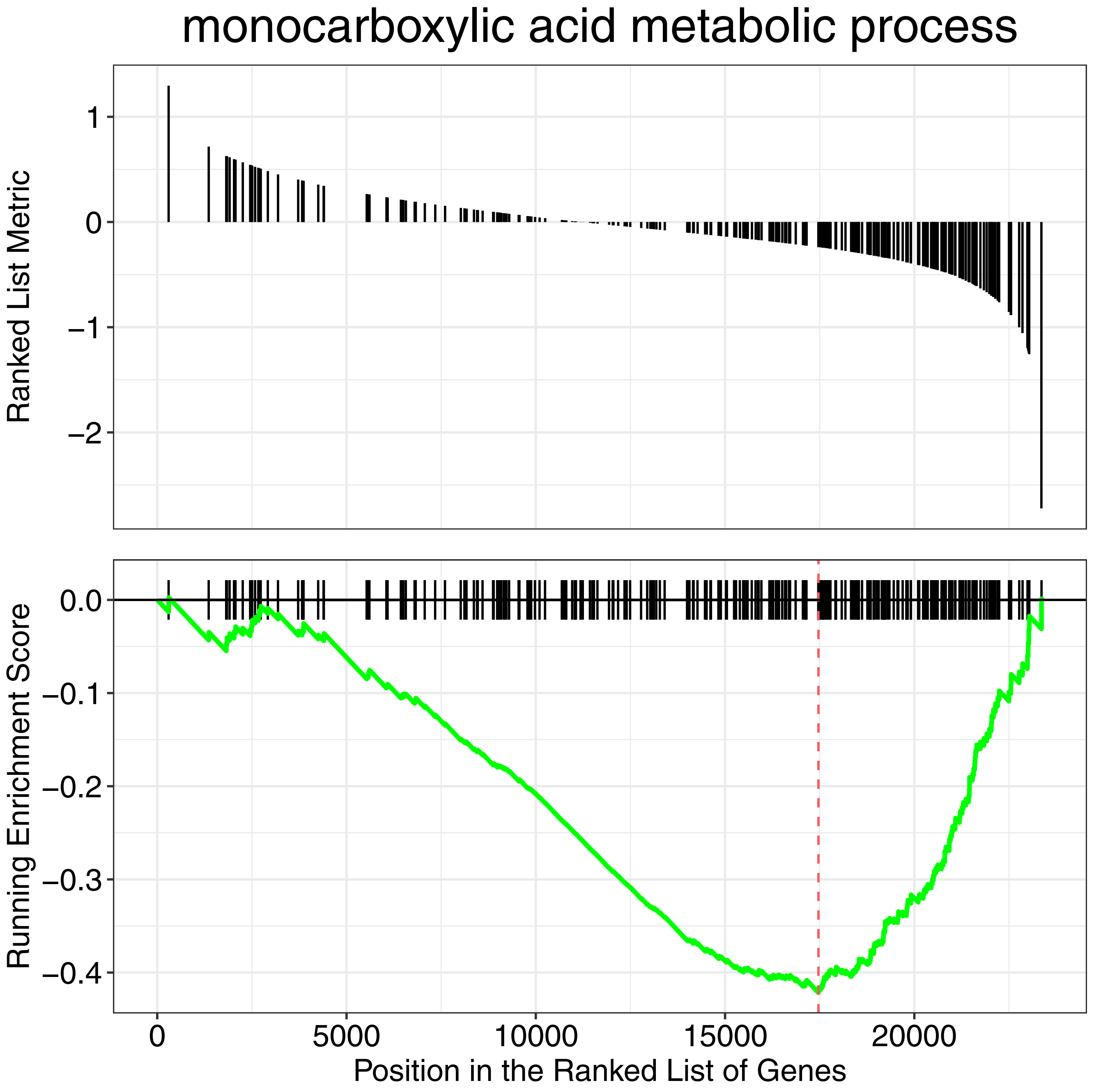

Biology Open • Supplementary information

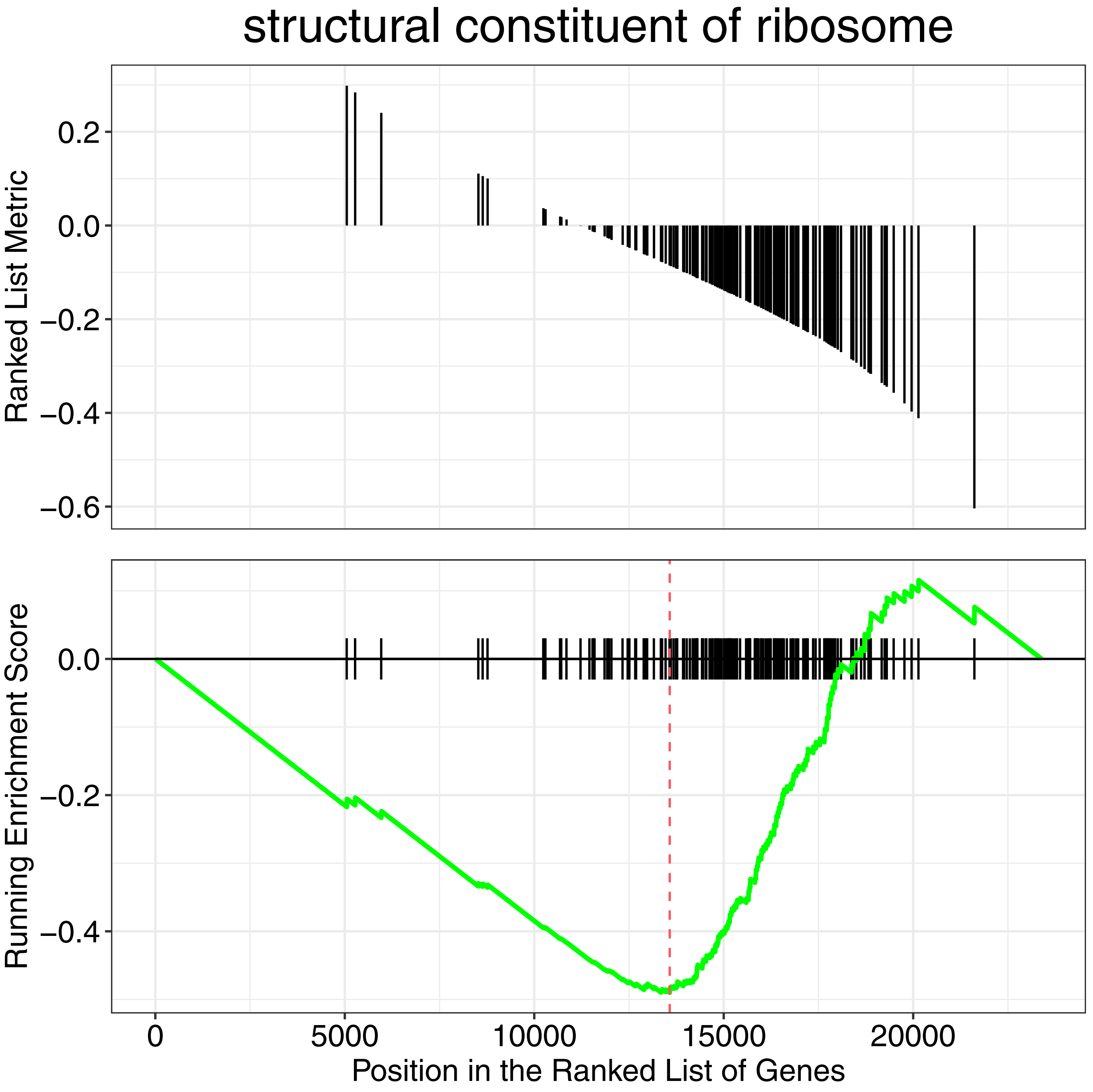

Biology Open • Supplementary information

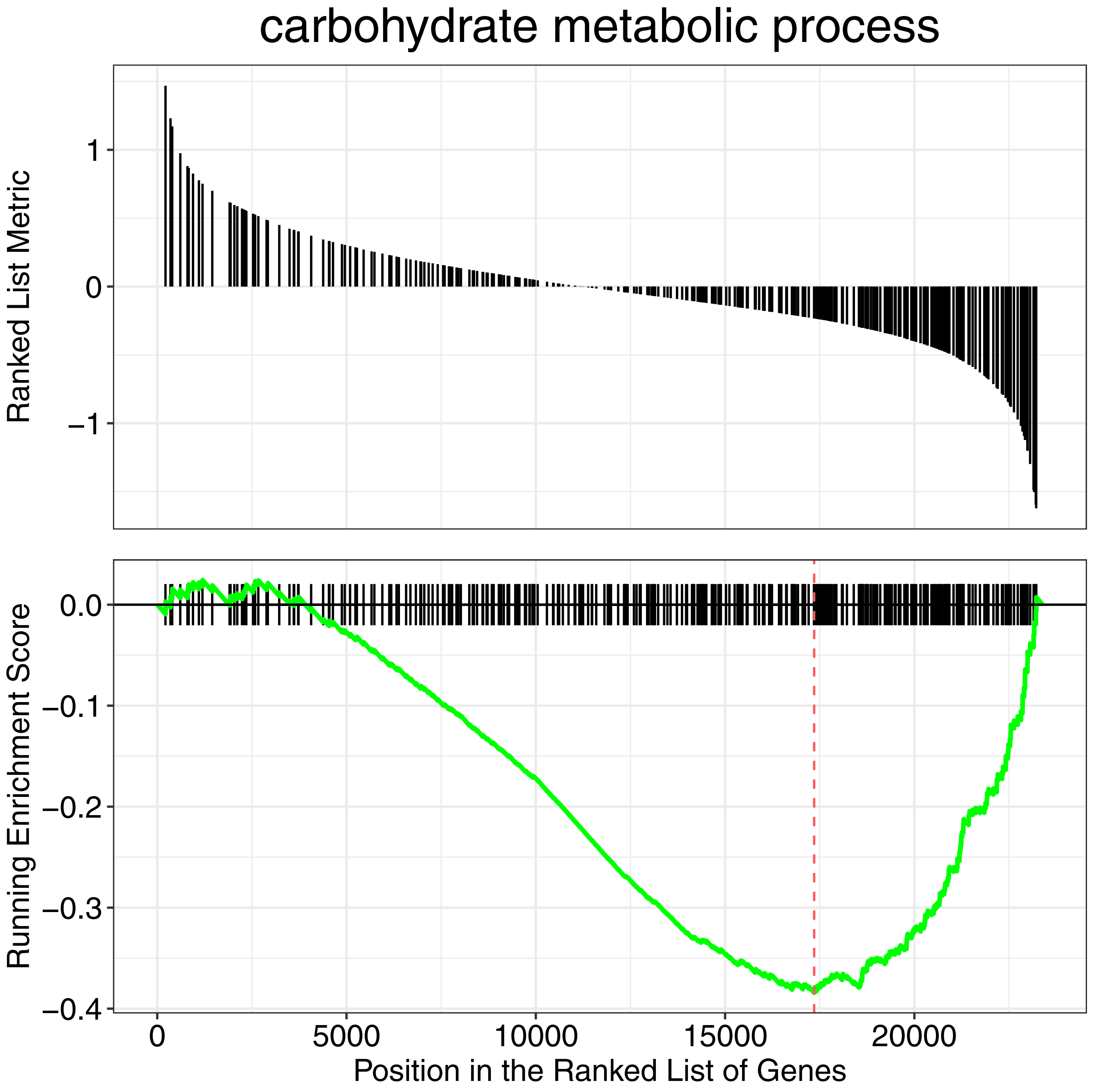

Biology Open • Supplementary information

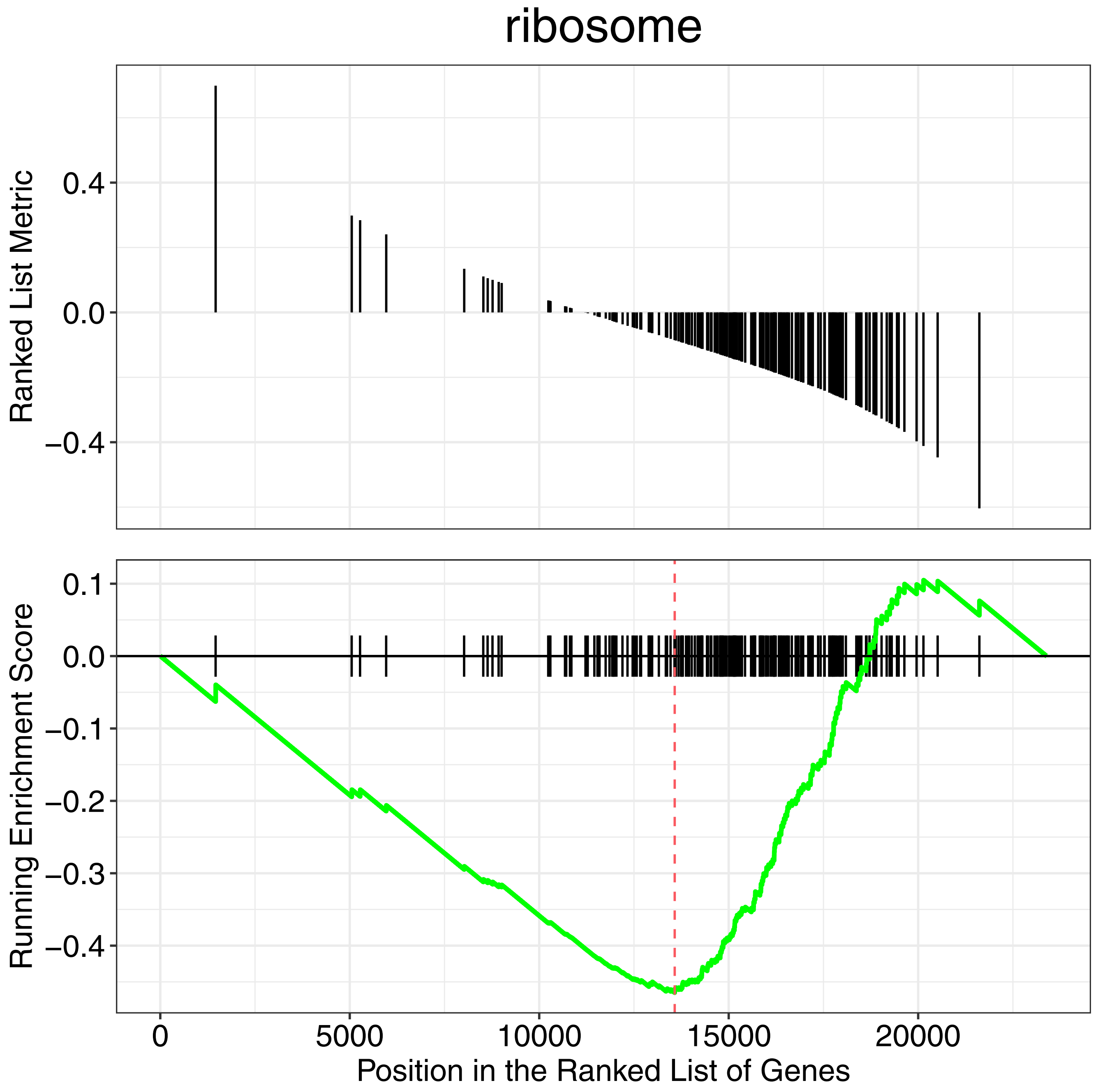

Biology Open • Supplementary information

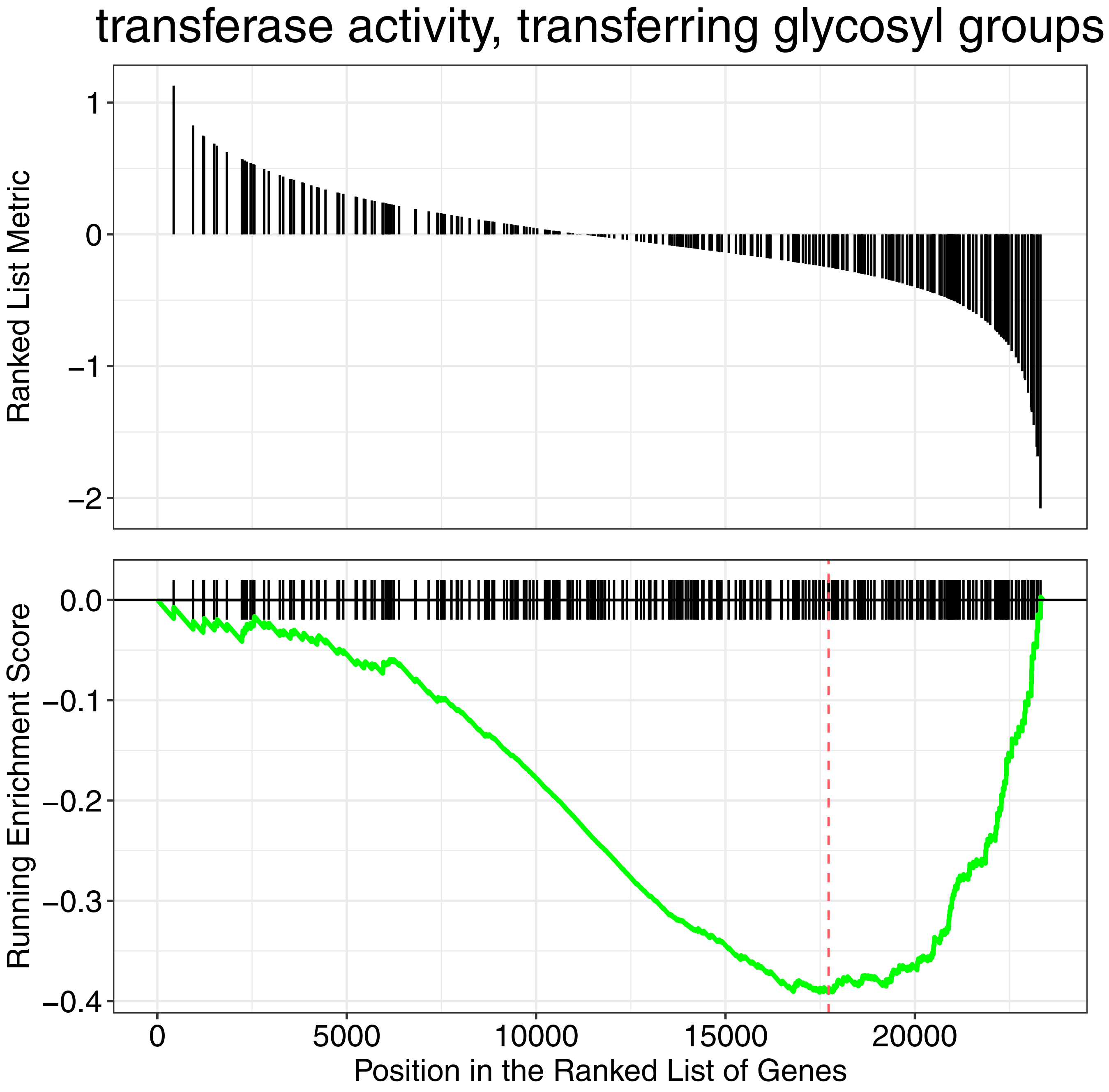

Biology Open • Supplementary information

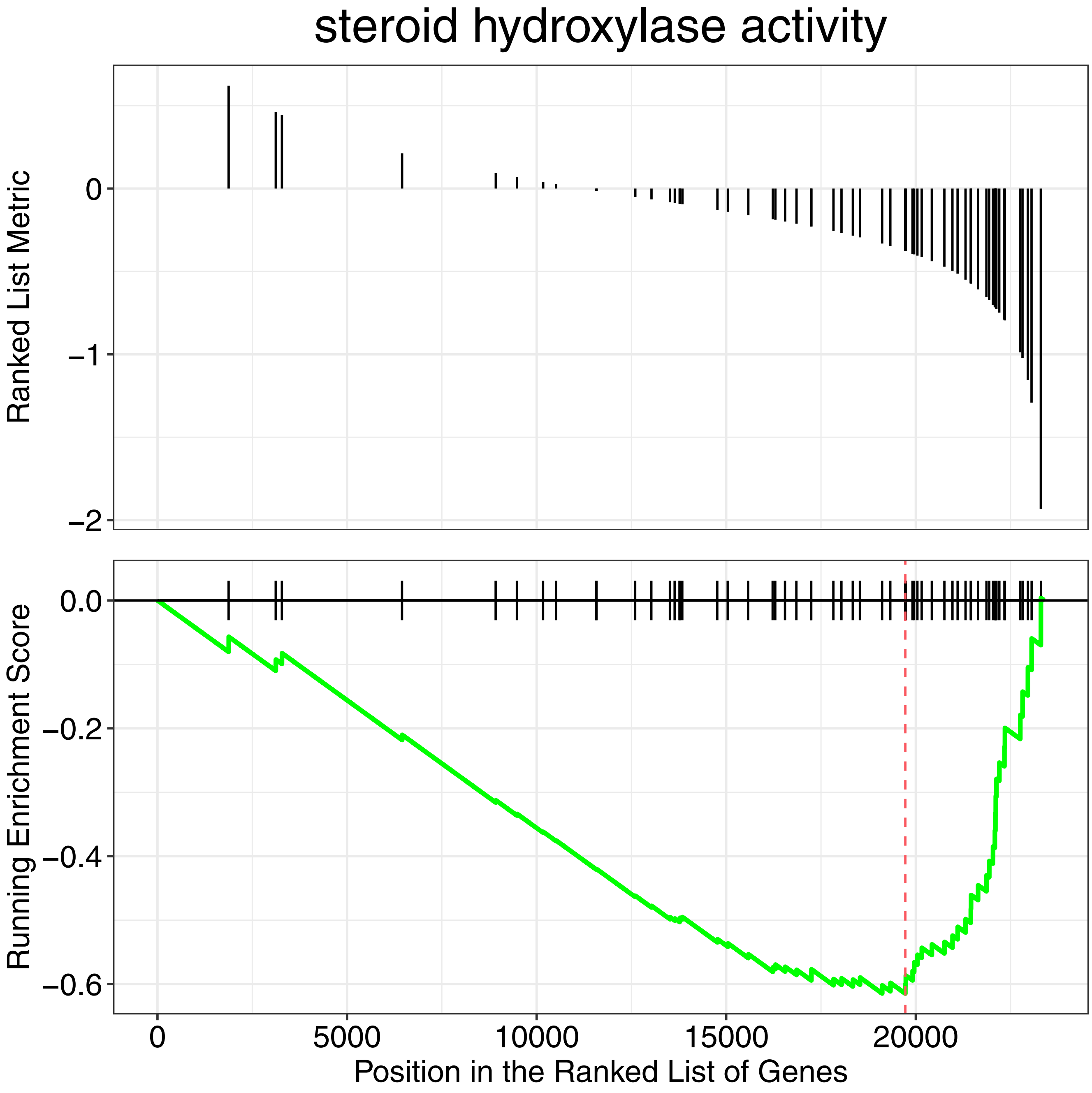

Biology Open • Supplementary information

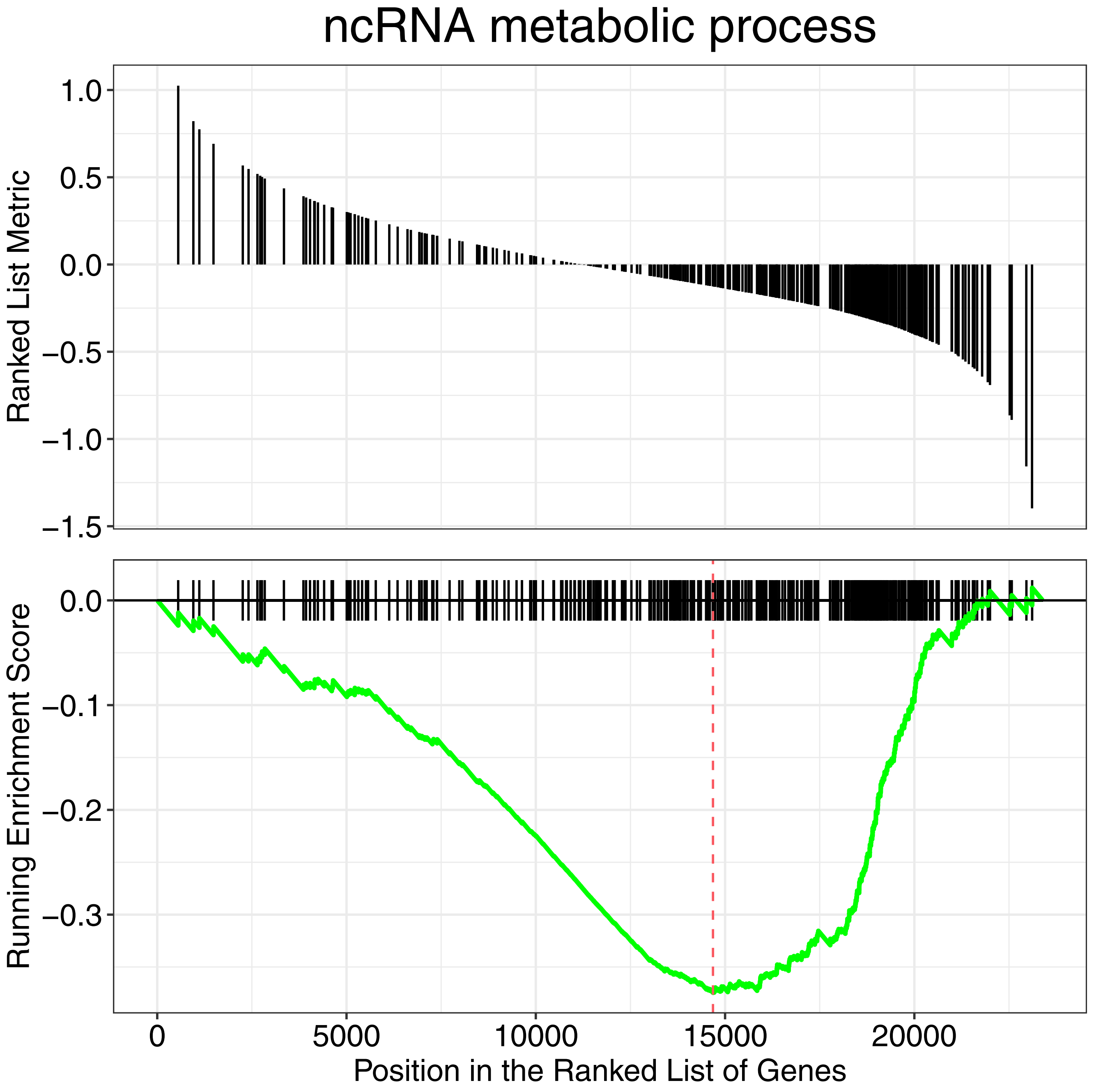

Biology Open • Supplementary information

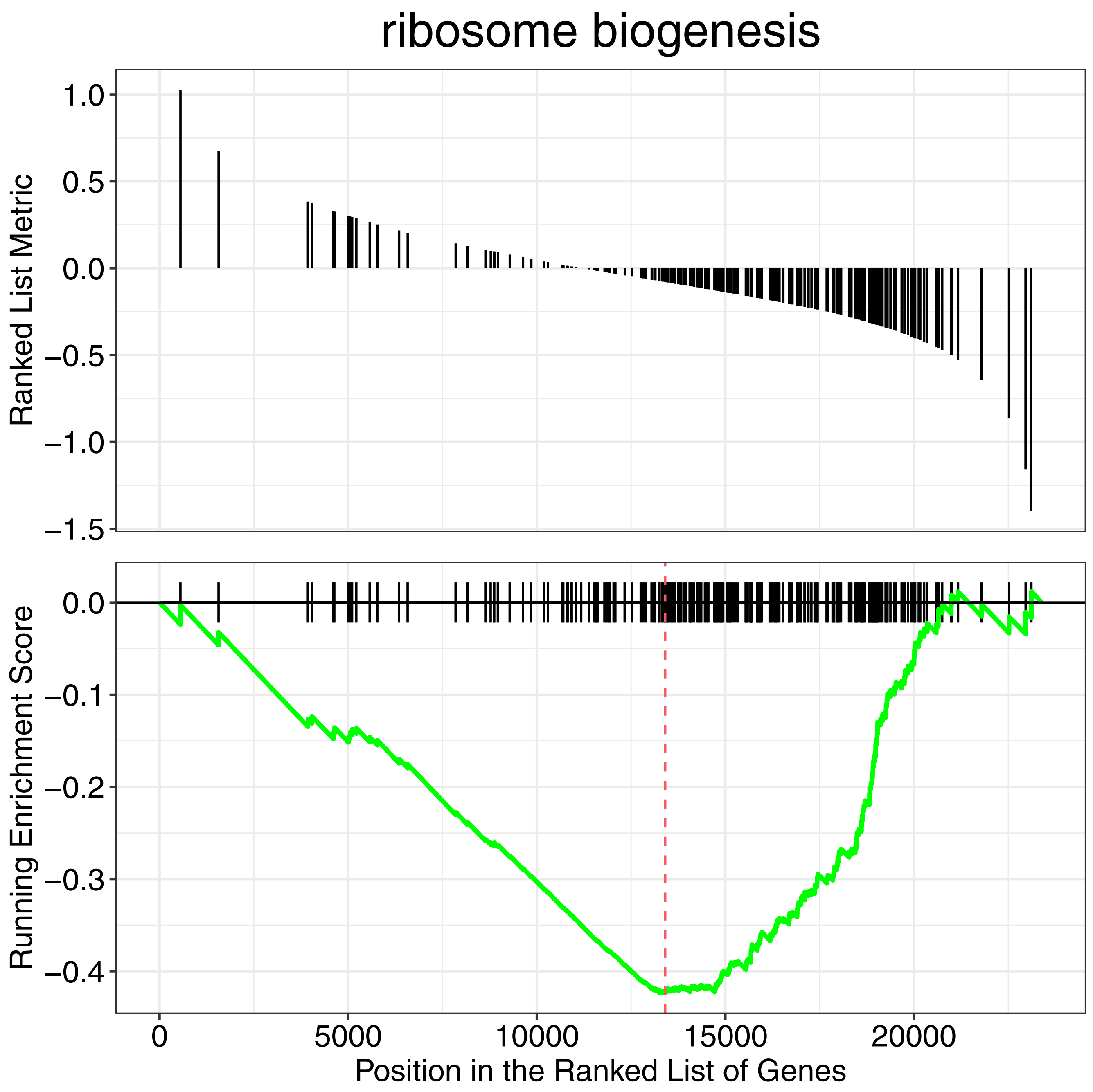

Biology Open • Supplementary information

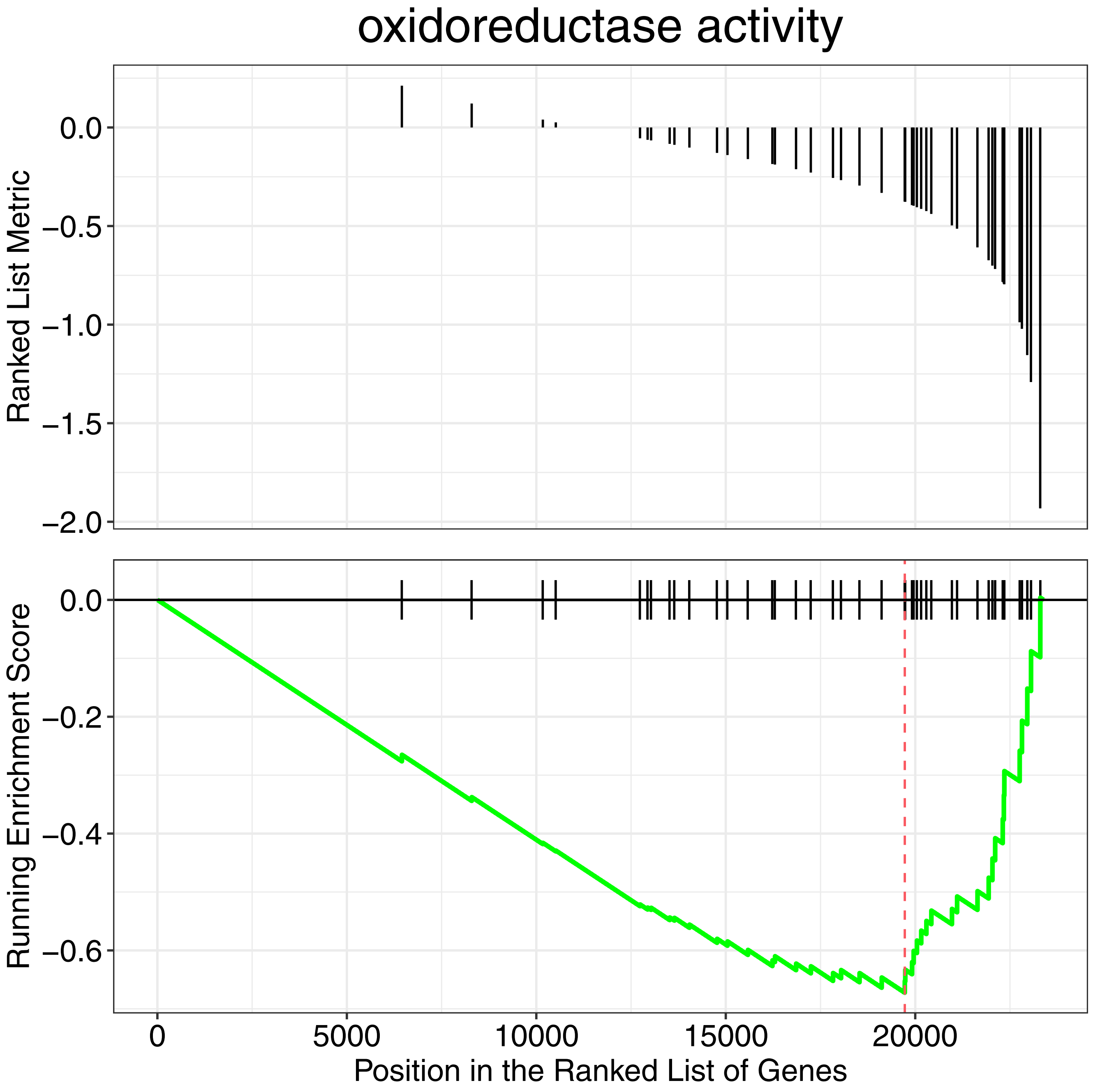

Fig. S4. Enrichment plots of Gene set enrichment analysis (GSEA) of differentially expressed genes in alx1;alx3 mutants. Running score plot and pre-ranked list of top 25 suppressed GSEA terms shown in Figure 5C.

Biology Open • Supplementary information

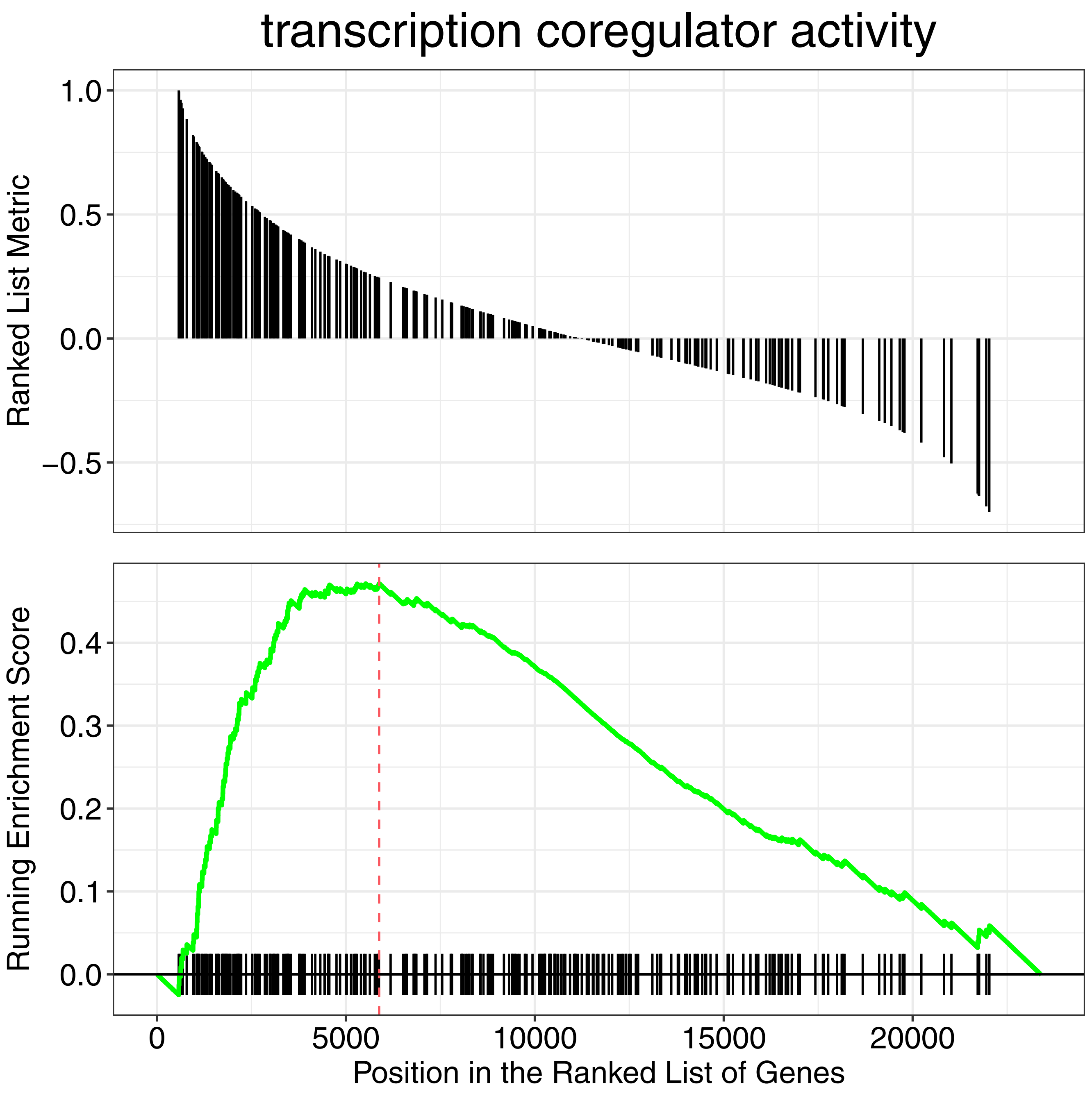

Biology Open • Supplementary information

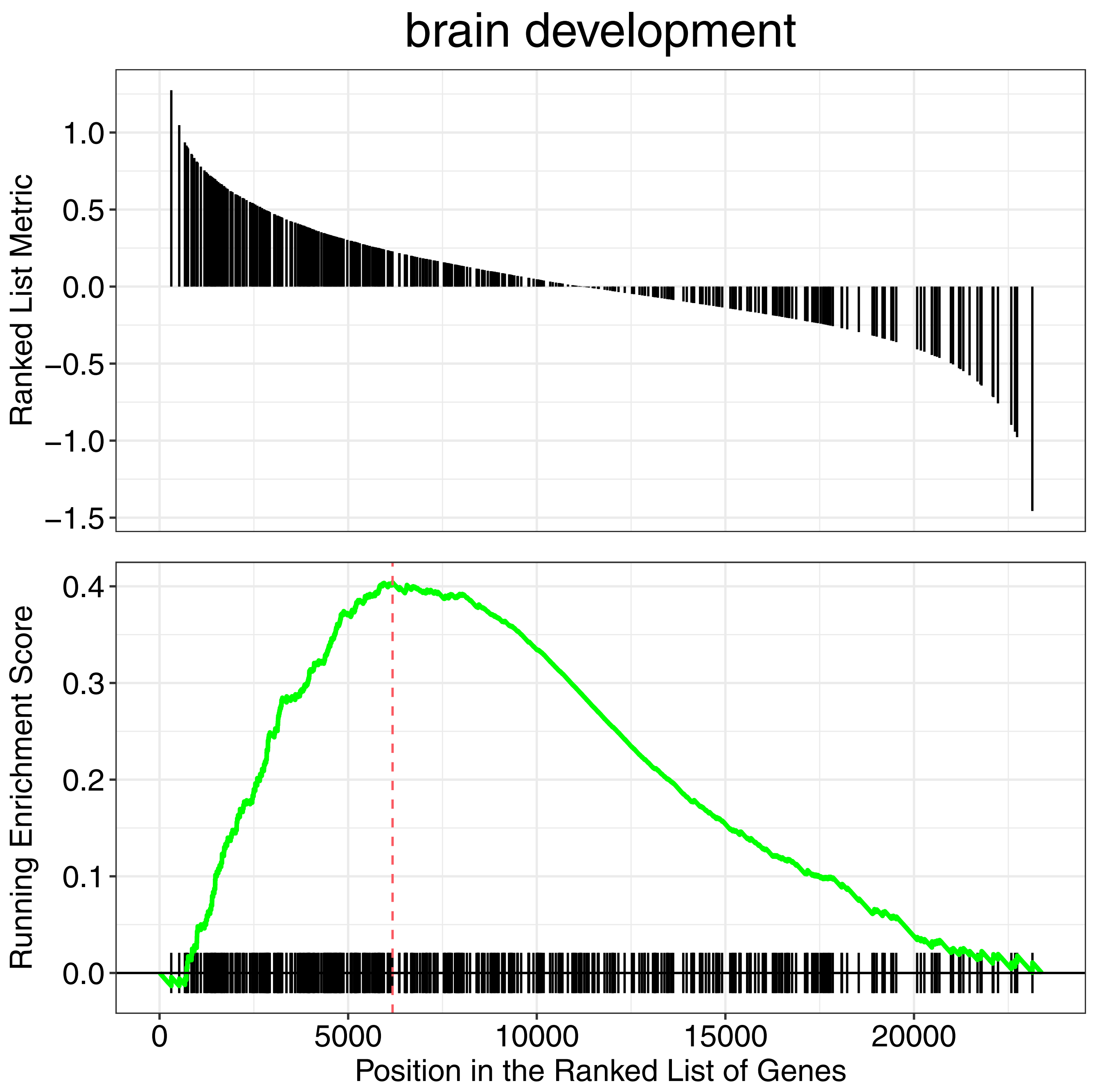

Biology Open • Supplementary information

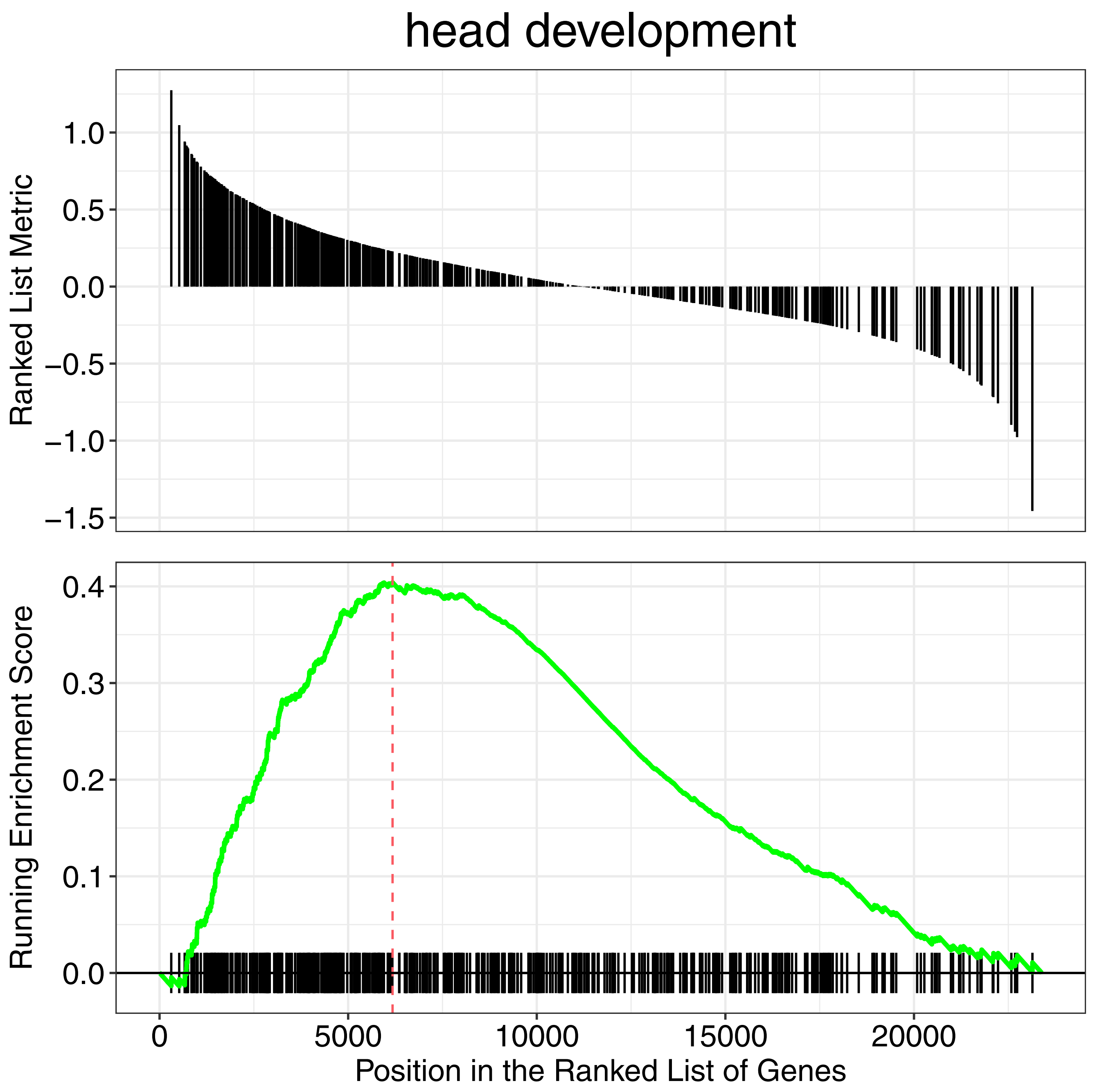

Biology Open • Supplementary information

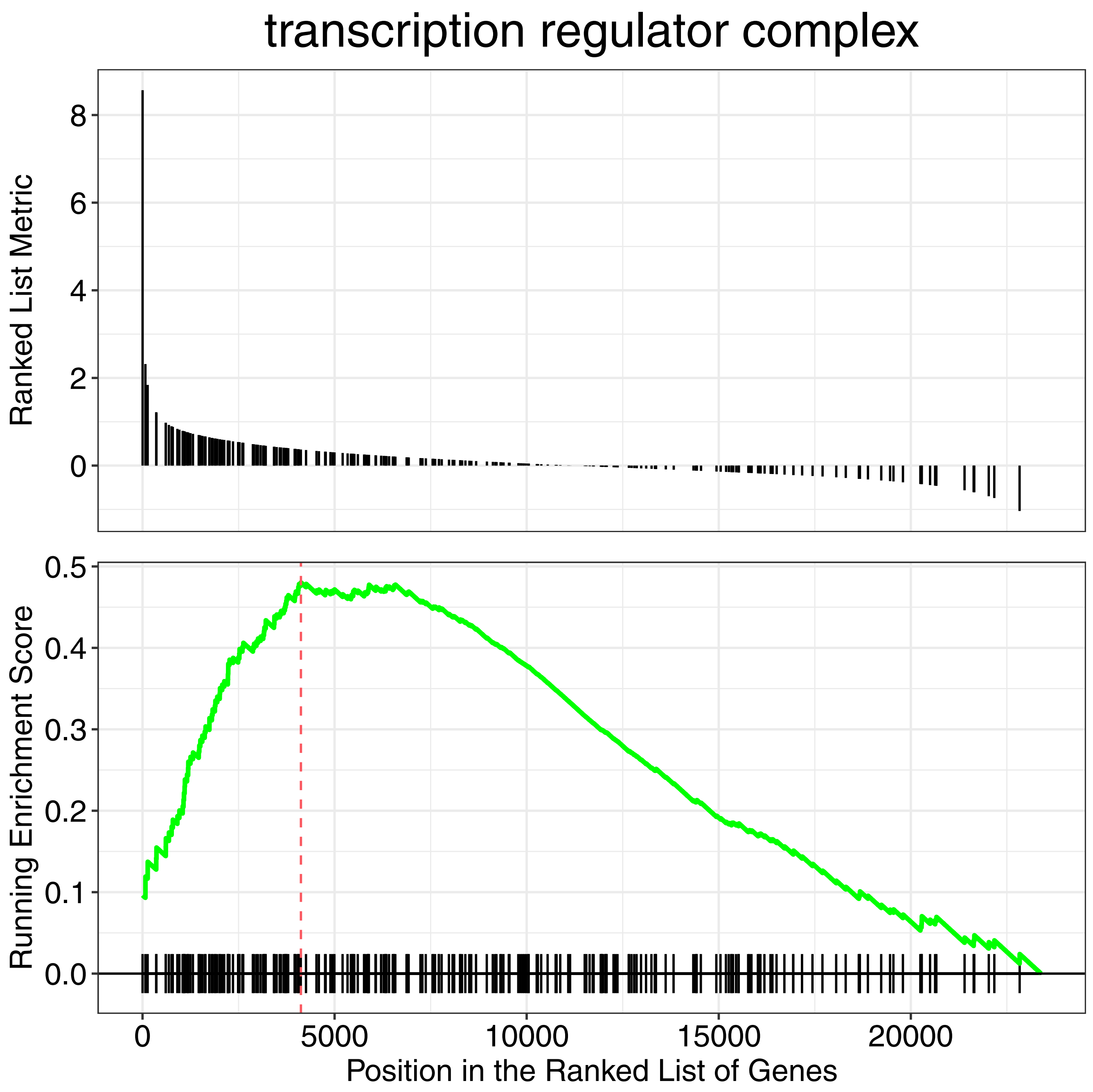

Biology Open • Supplementary information

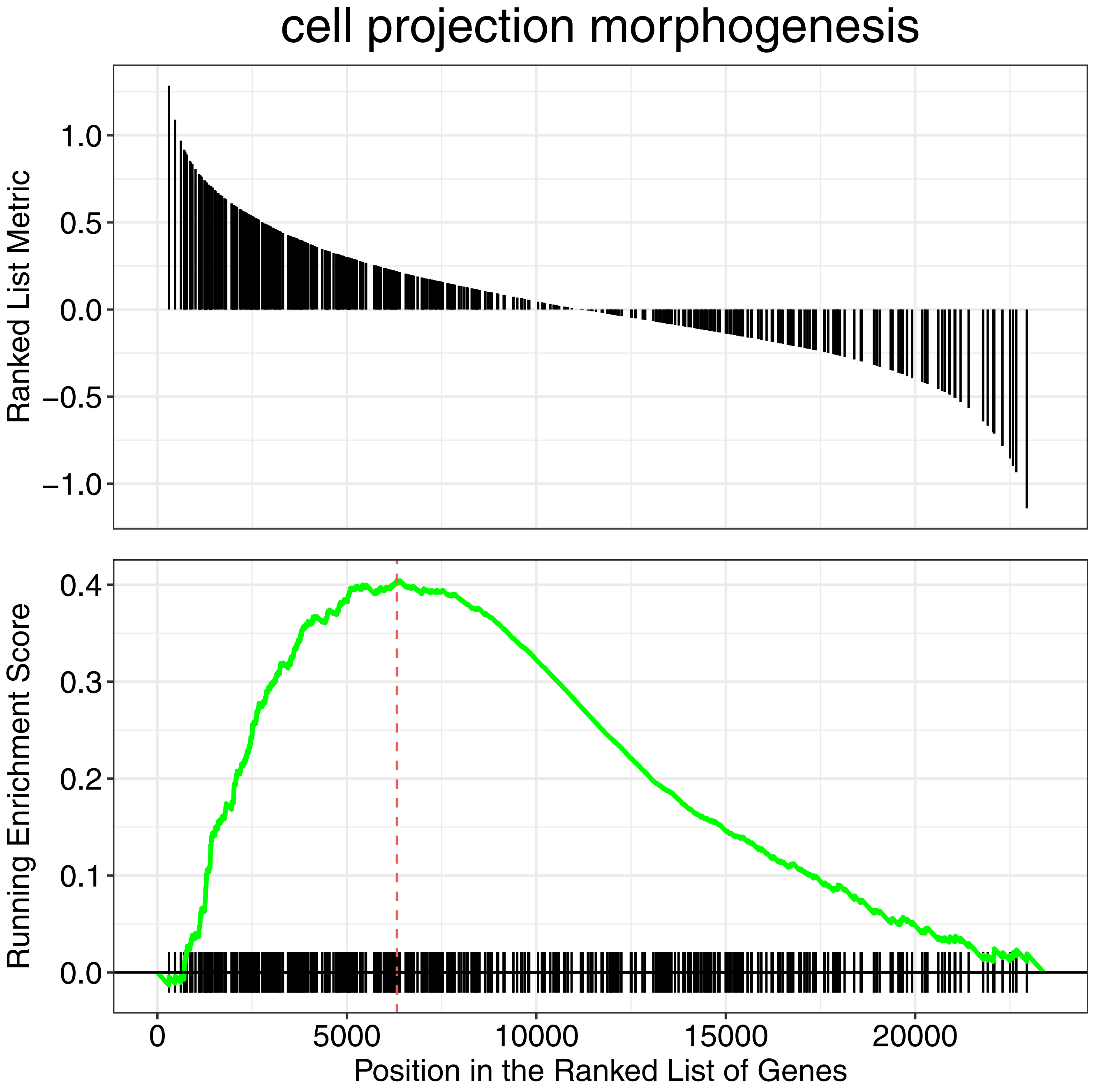

Biology Open • Supplementary information

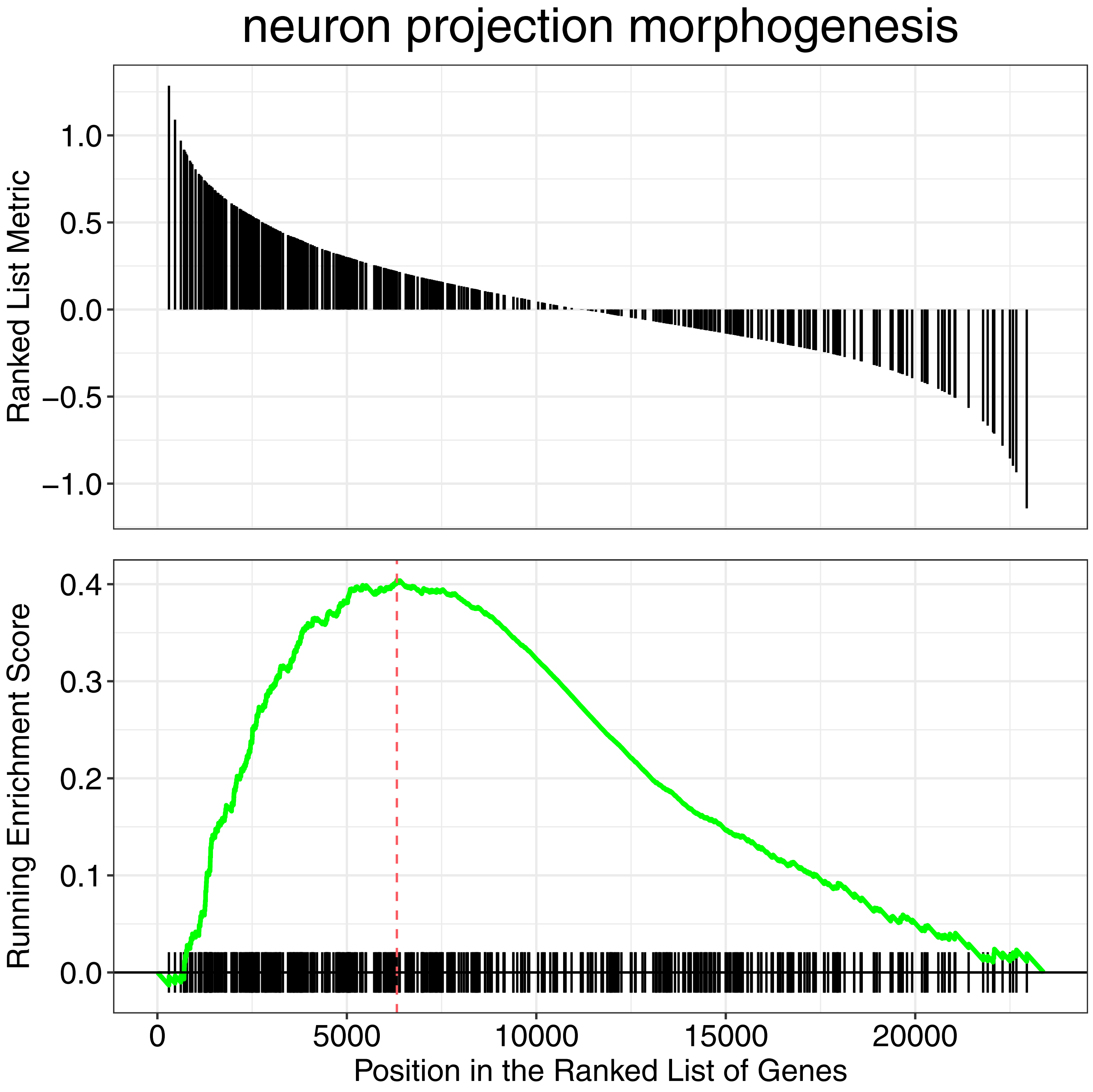

Biology Open • Supplementary information

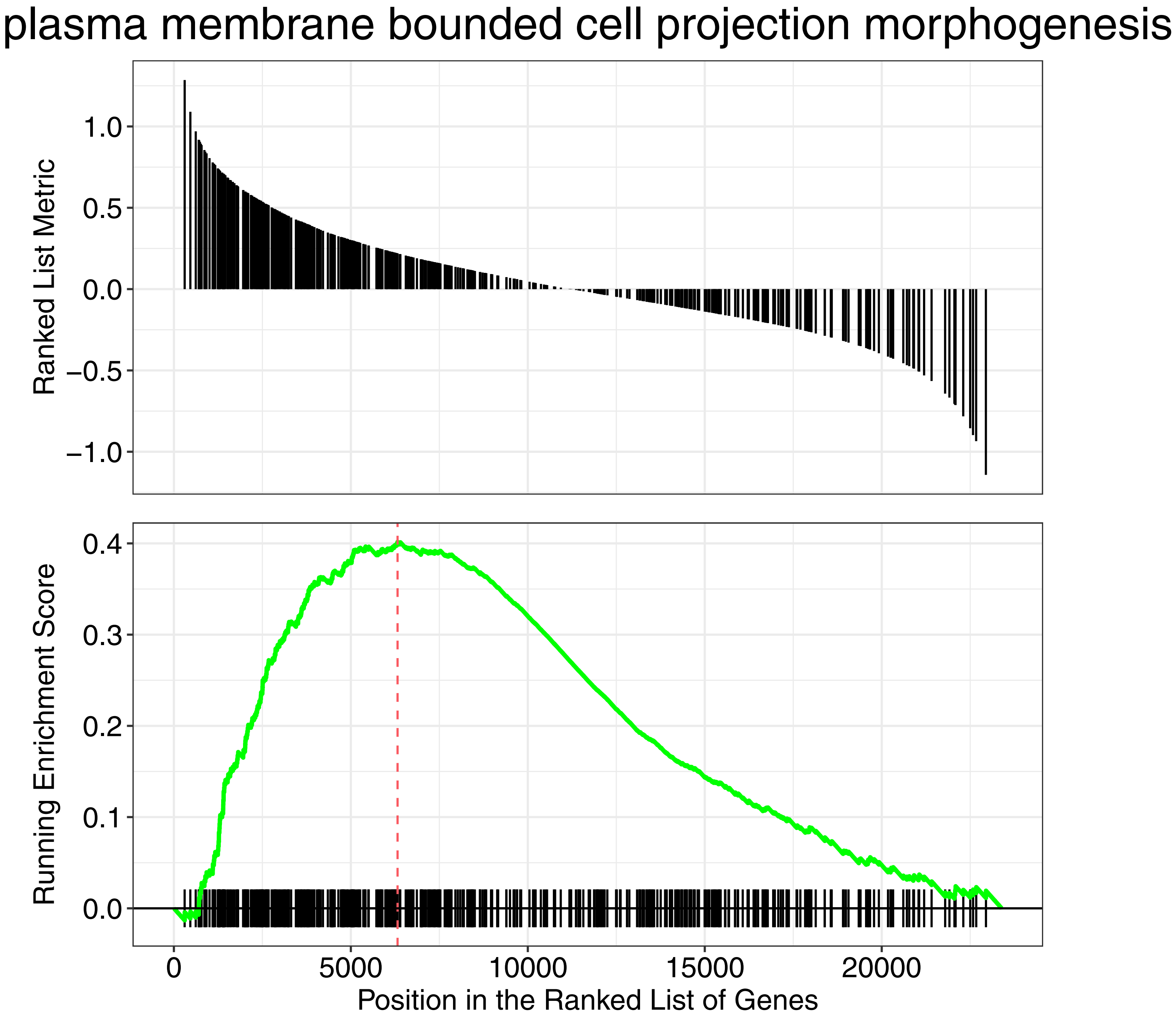

Biology Open • Supplementary information

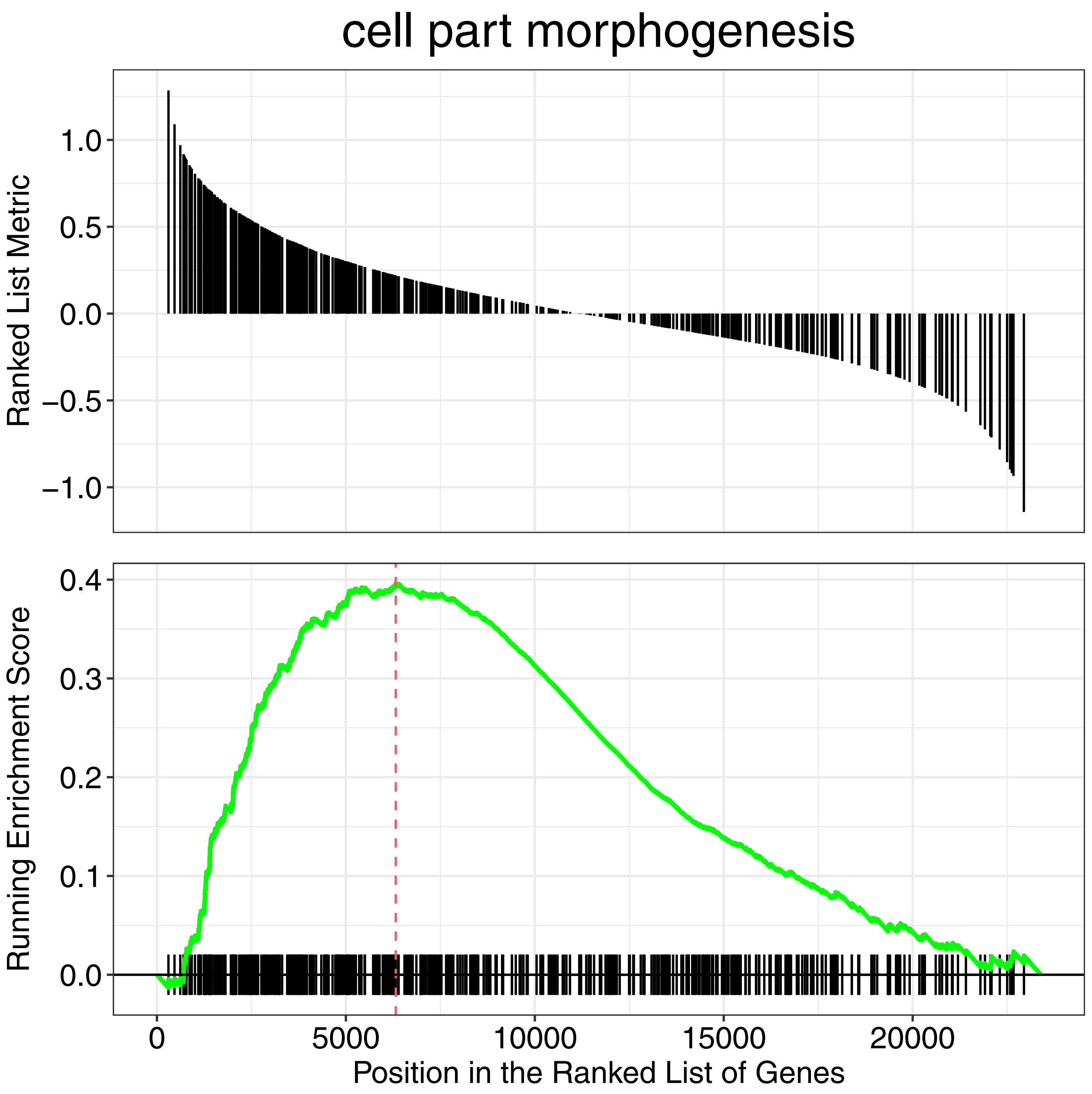

Biology Open • Supplementary information

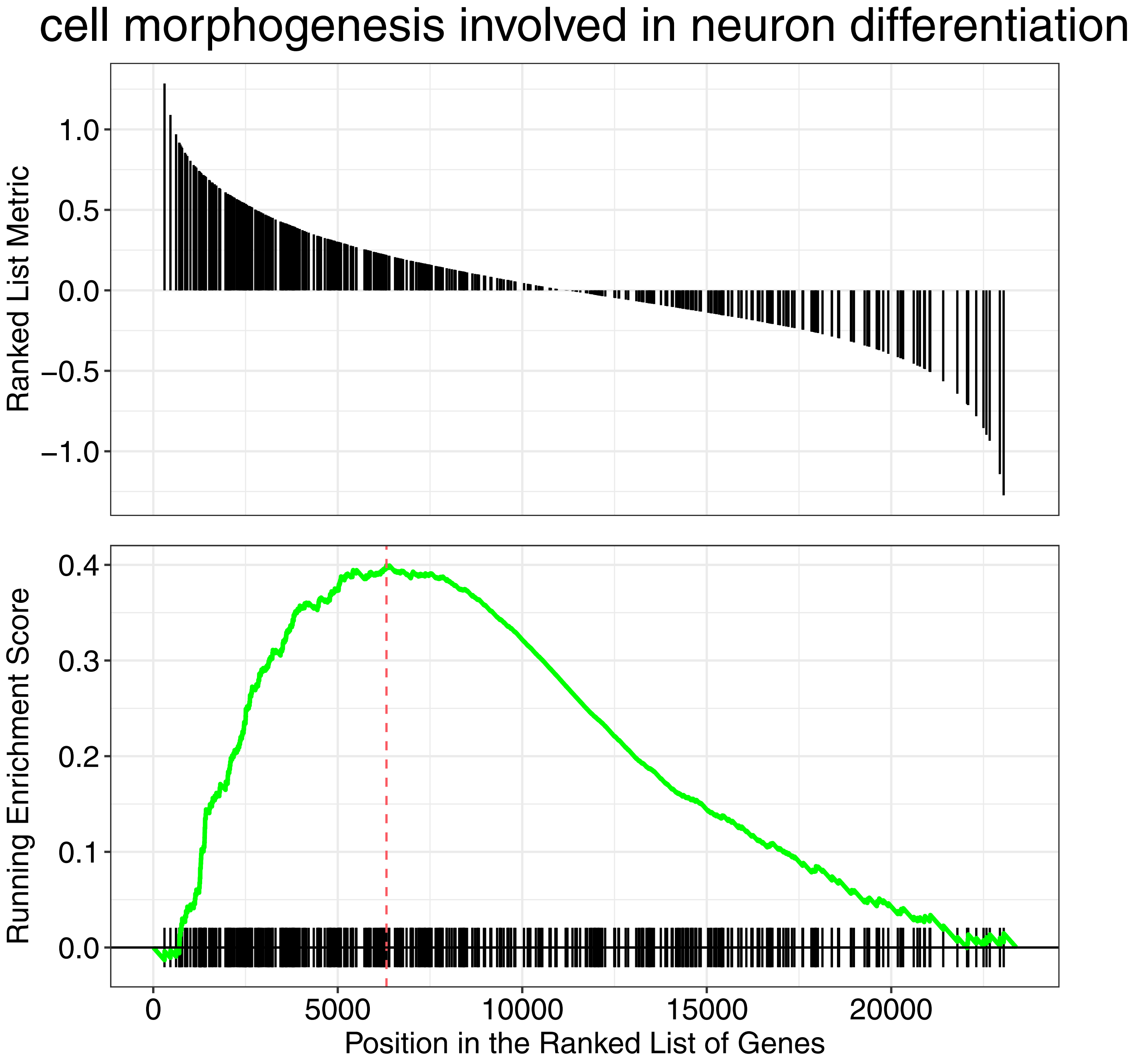

Biology Open • Supplementary information

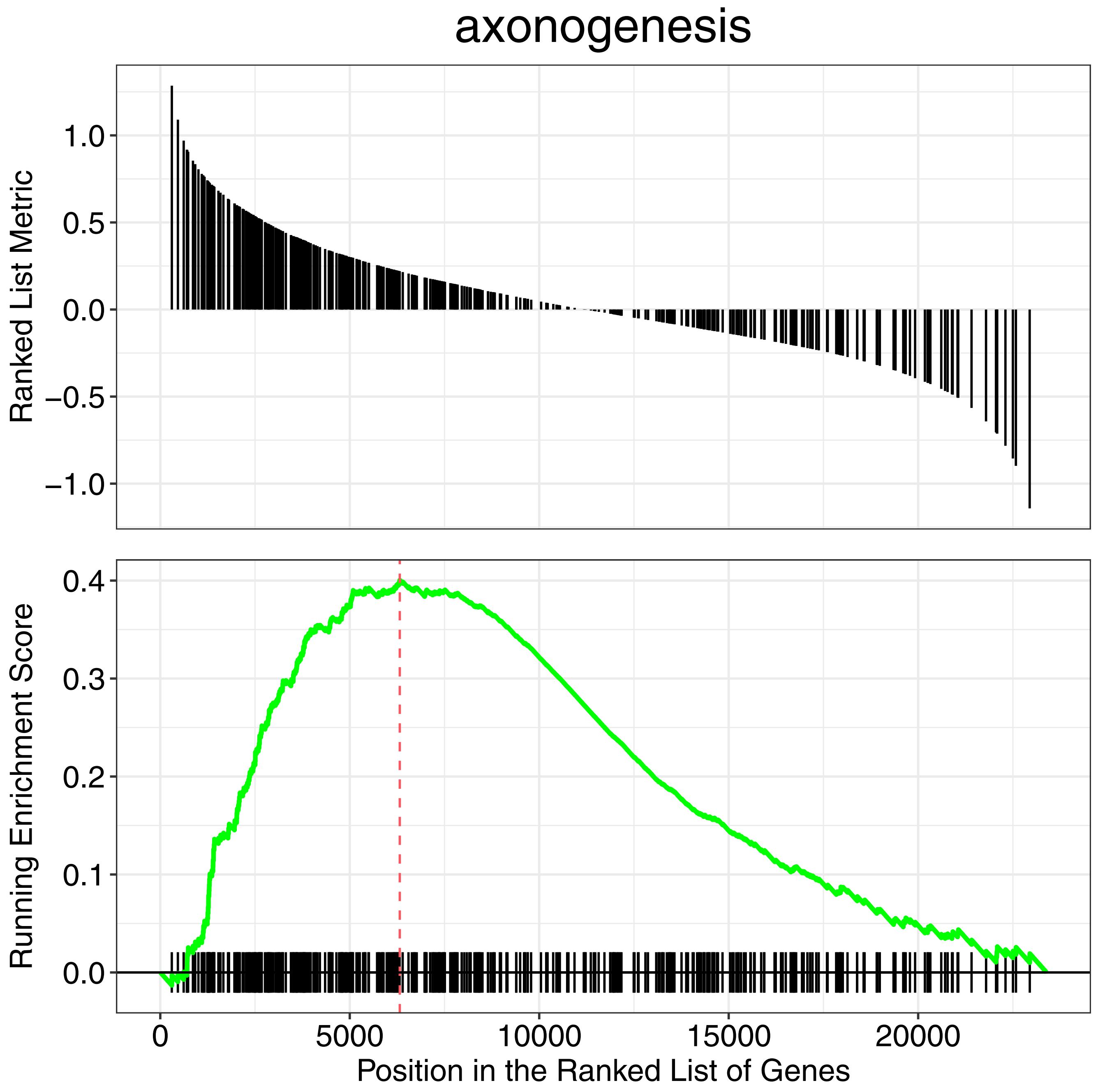

Biology Open • Supplementary information

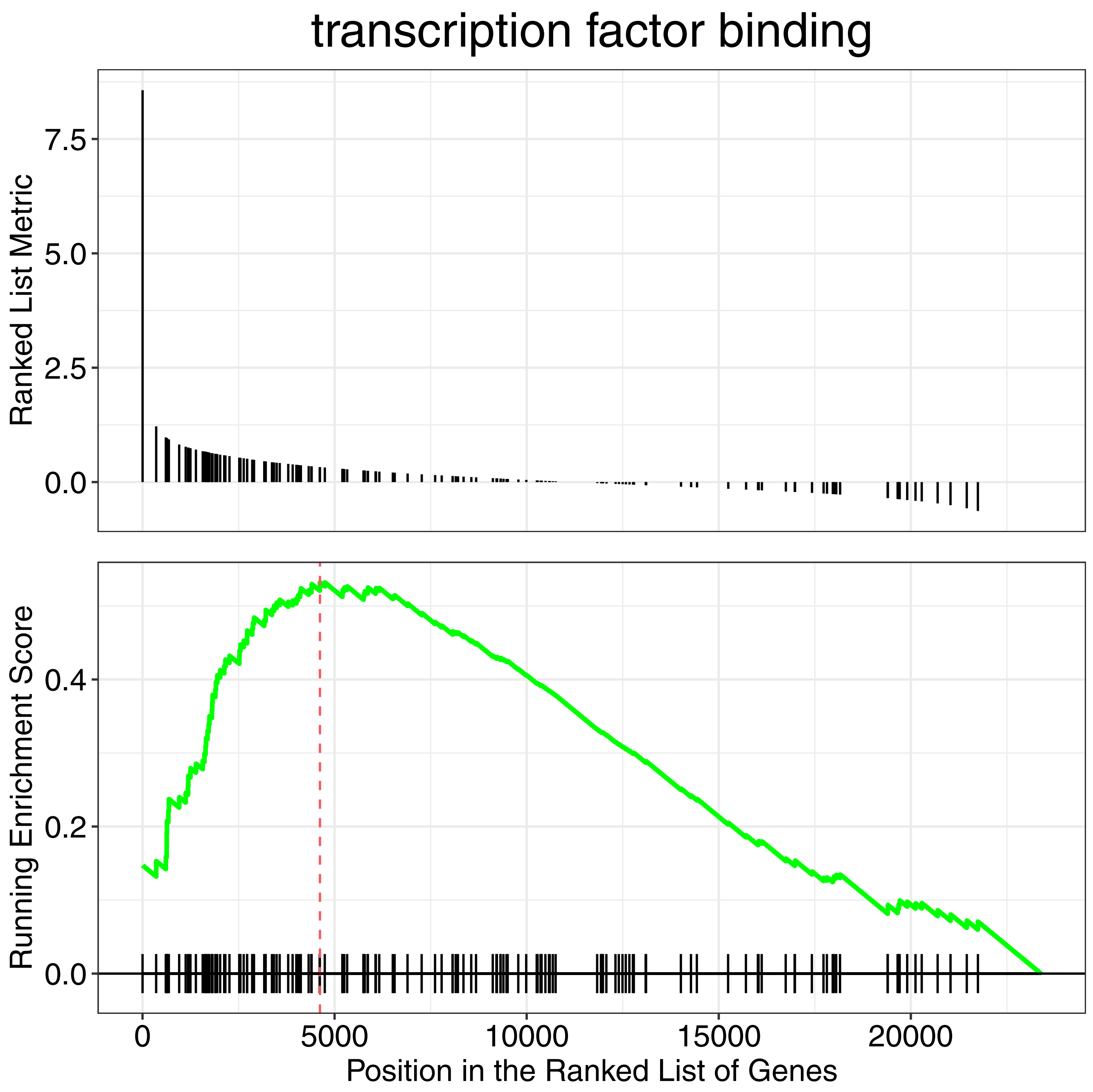

Biology Open • Supplementary information

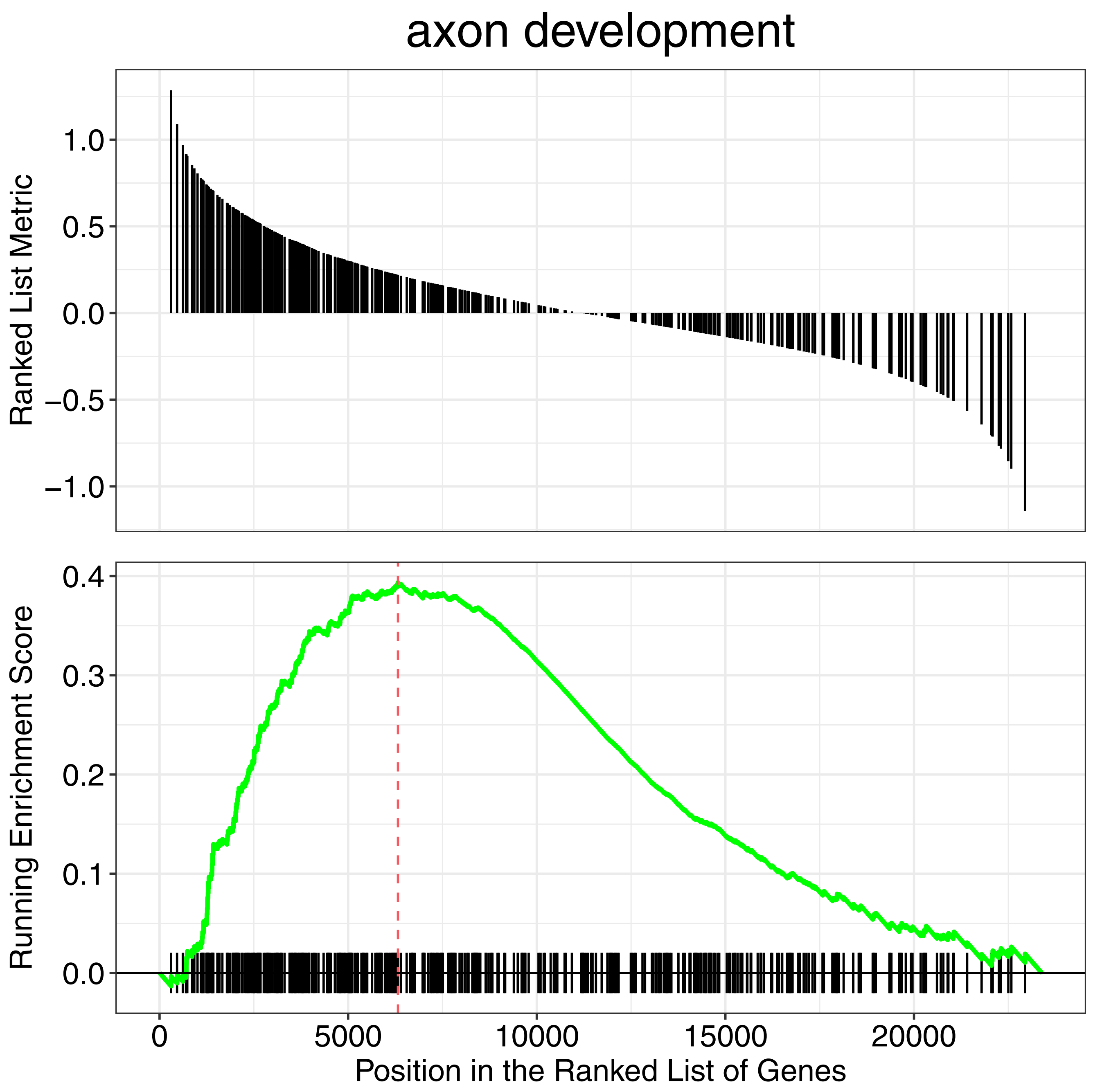

Biology Open • Supplementary information

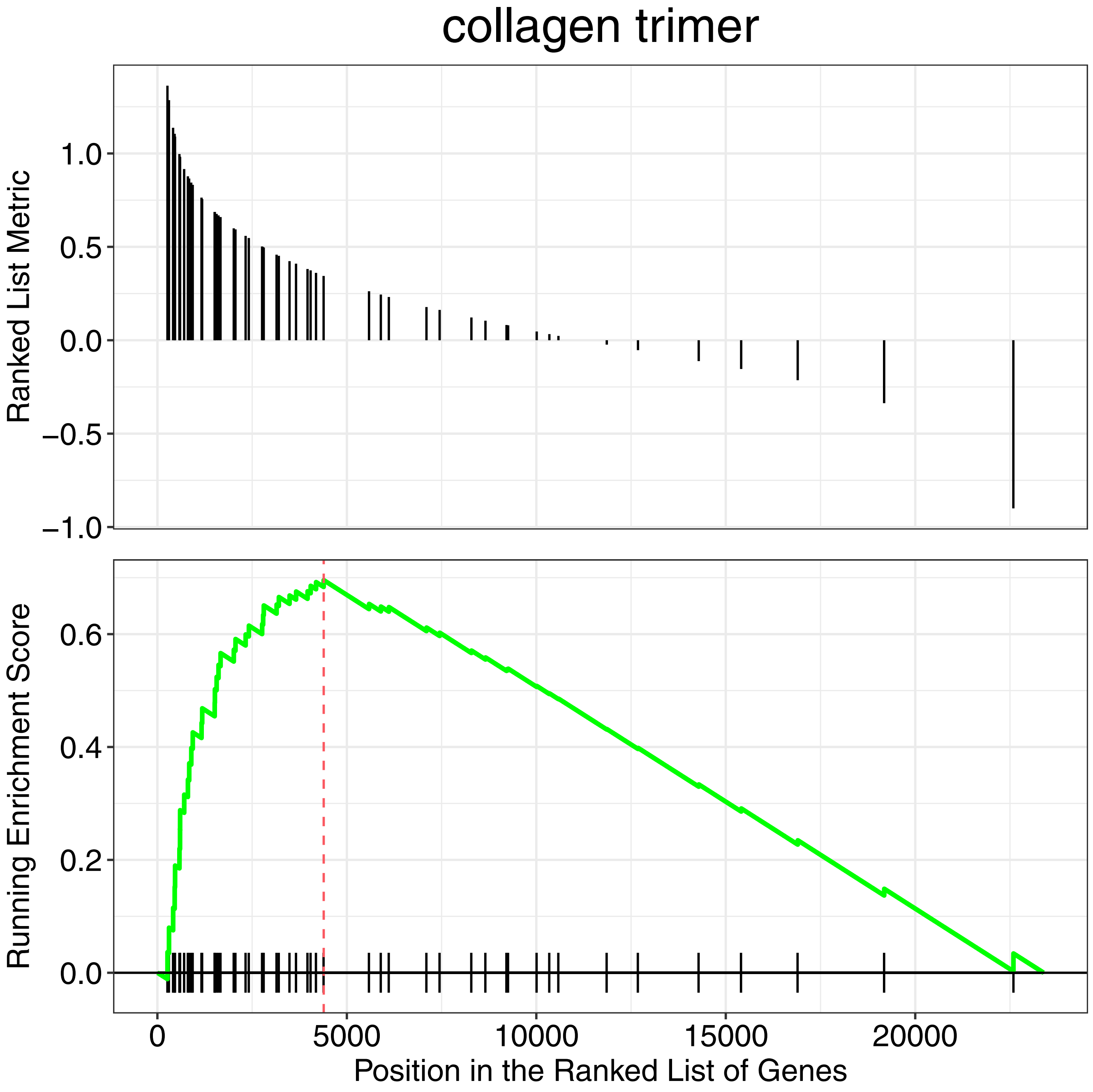

Biology Open • Supplementary information

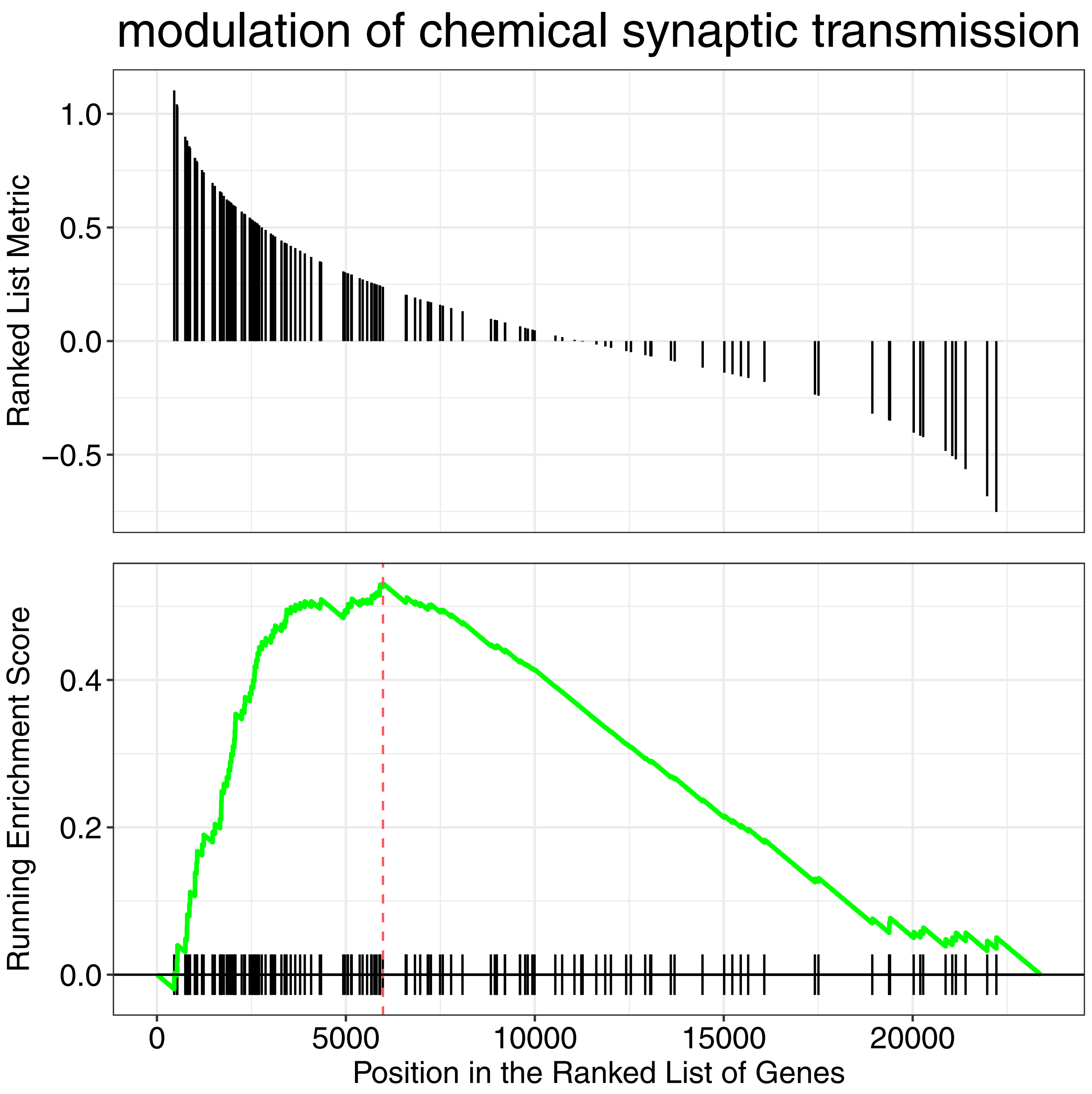

Biology Open • Supplementary information

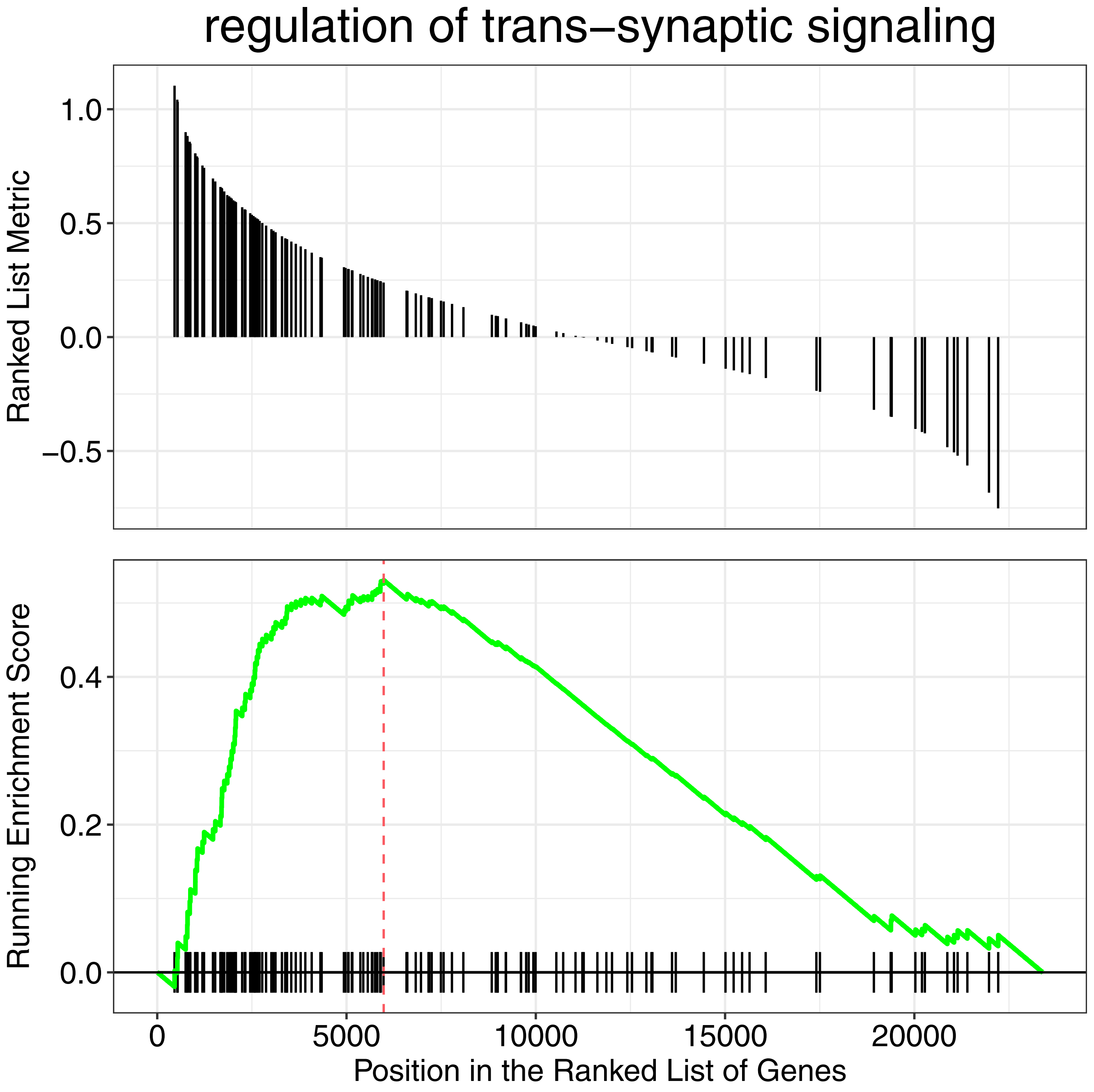

Biology Open • Supplementary information

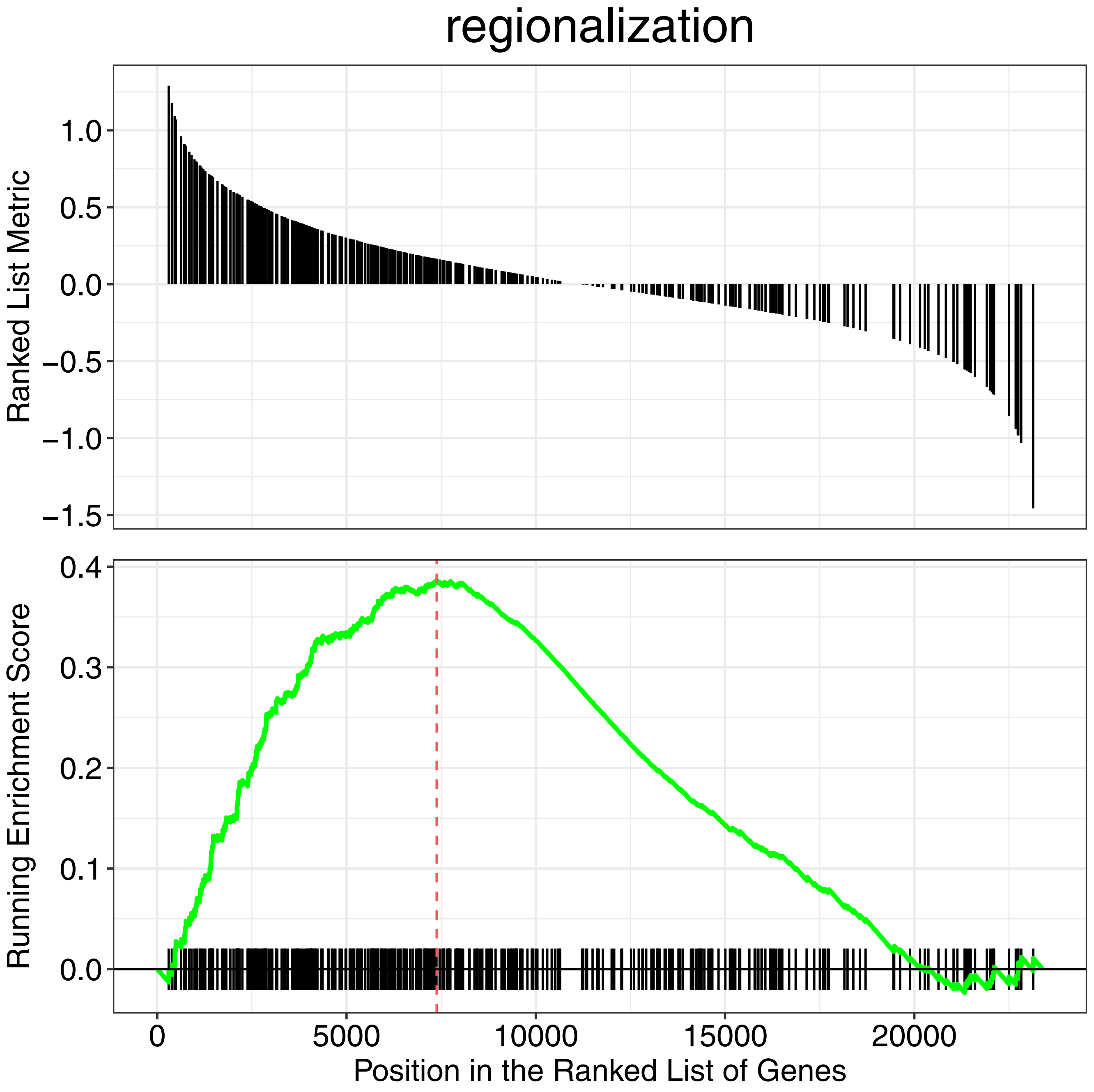
regionalization
Ranked List Metric
1.0
0.5
0.0
−0.5
−1.0
−1.5
Running Enrichment Score
0.4
0.3
0.2
0.1
0.0
0
5000
10000
15000
20000
Position in the Ranked List of Genes

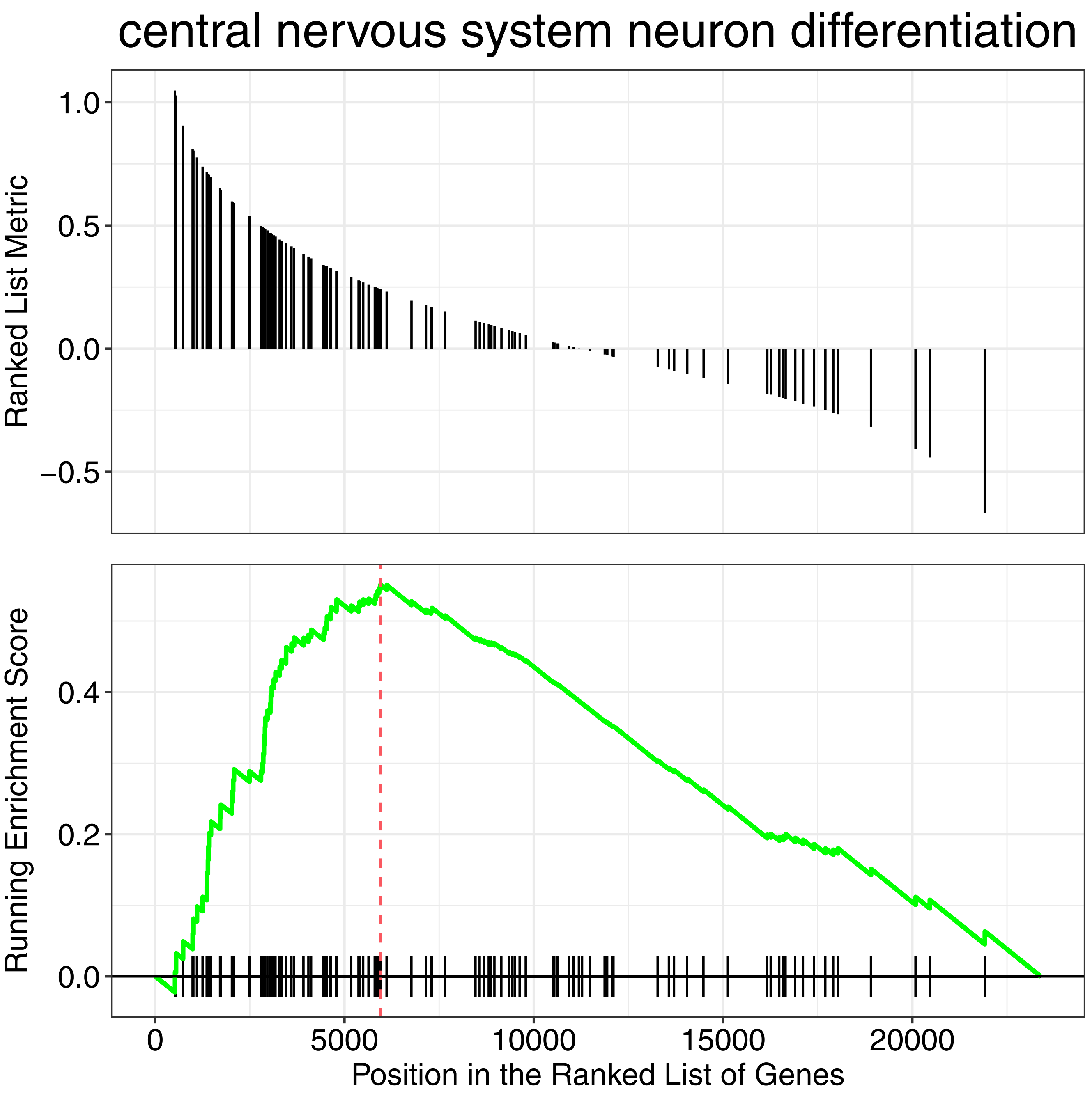

Biology Open • Supplementary information

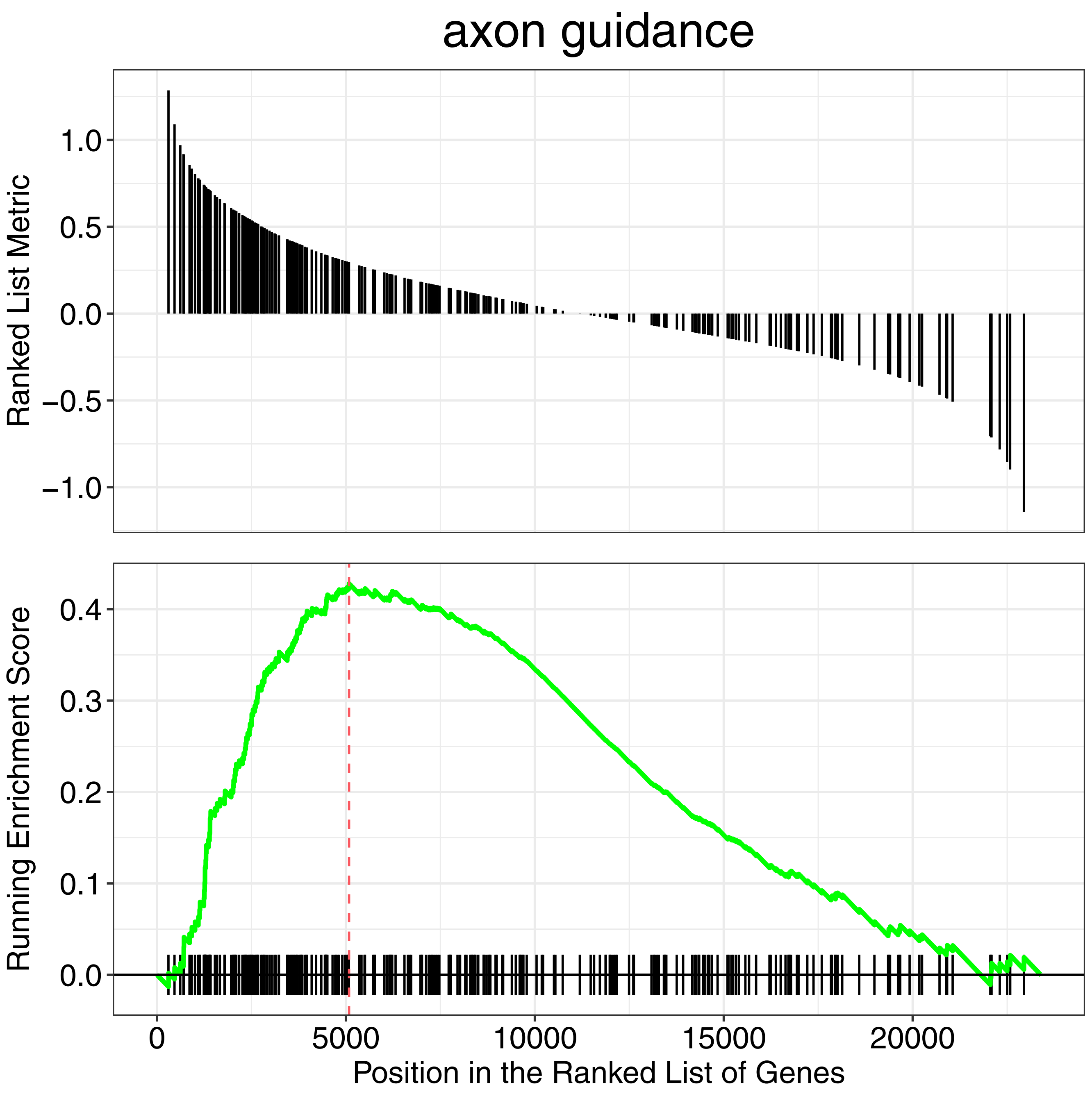

Biology Open • Supplementary information

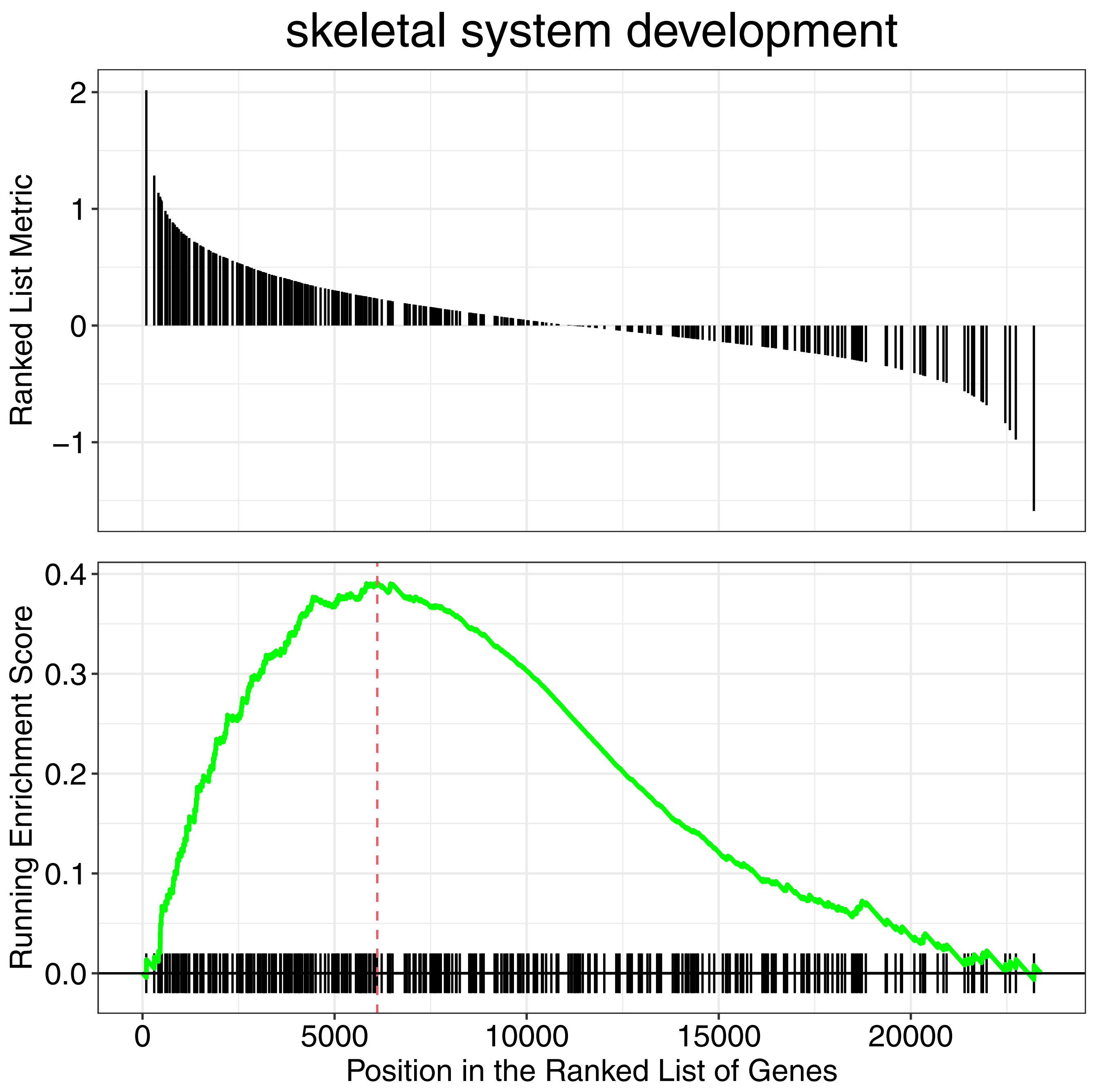

Biology Open • Supplementary information

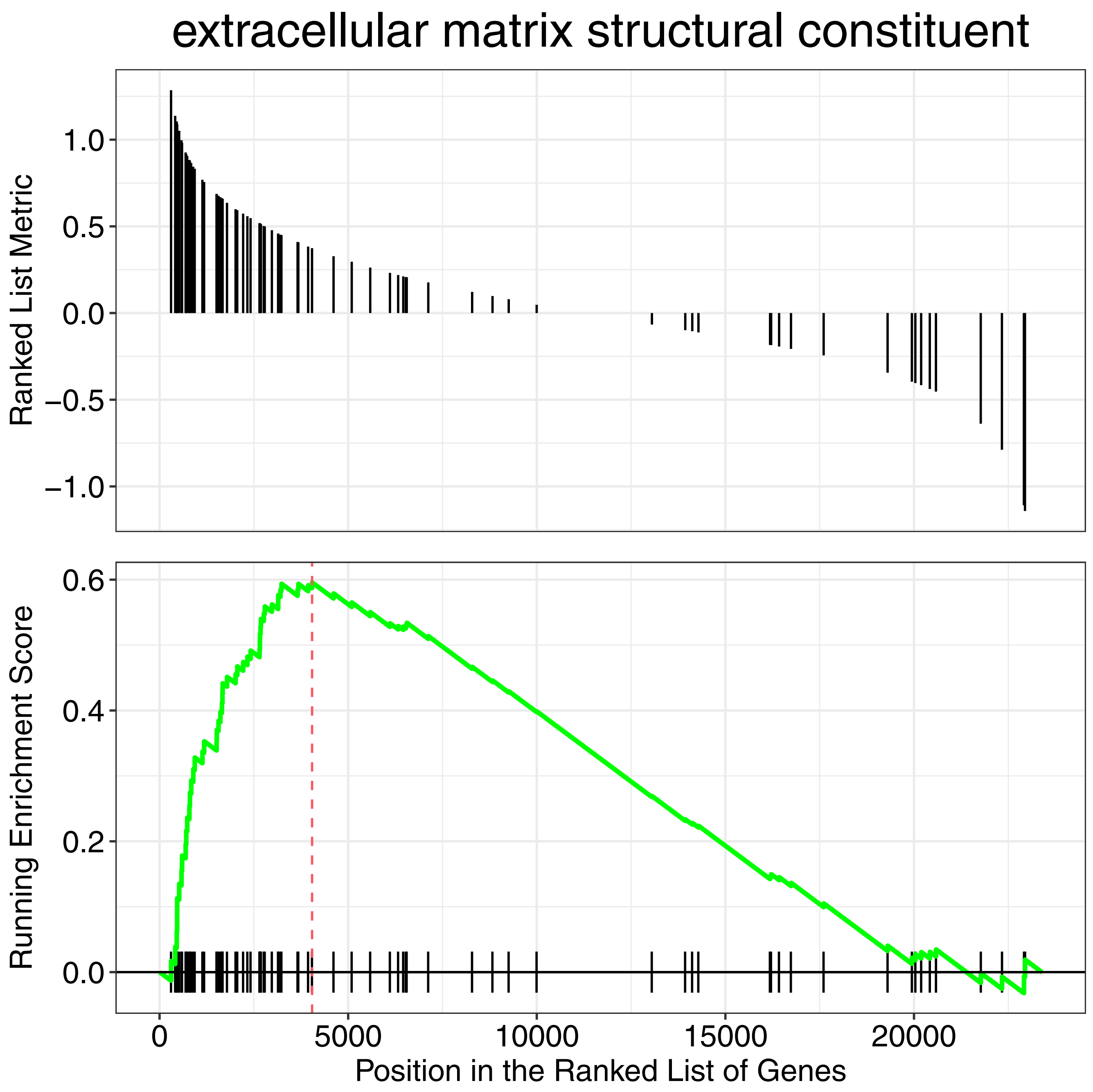

Biology Open • Supplementary information

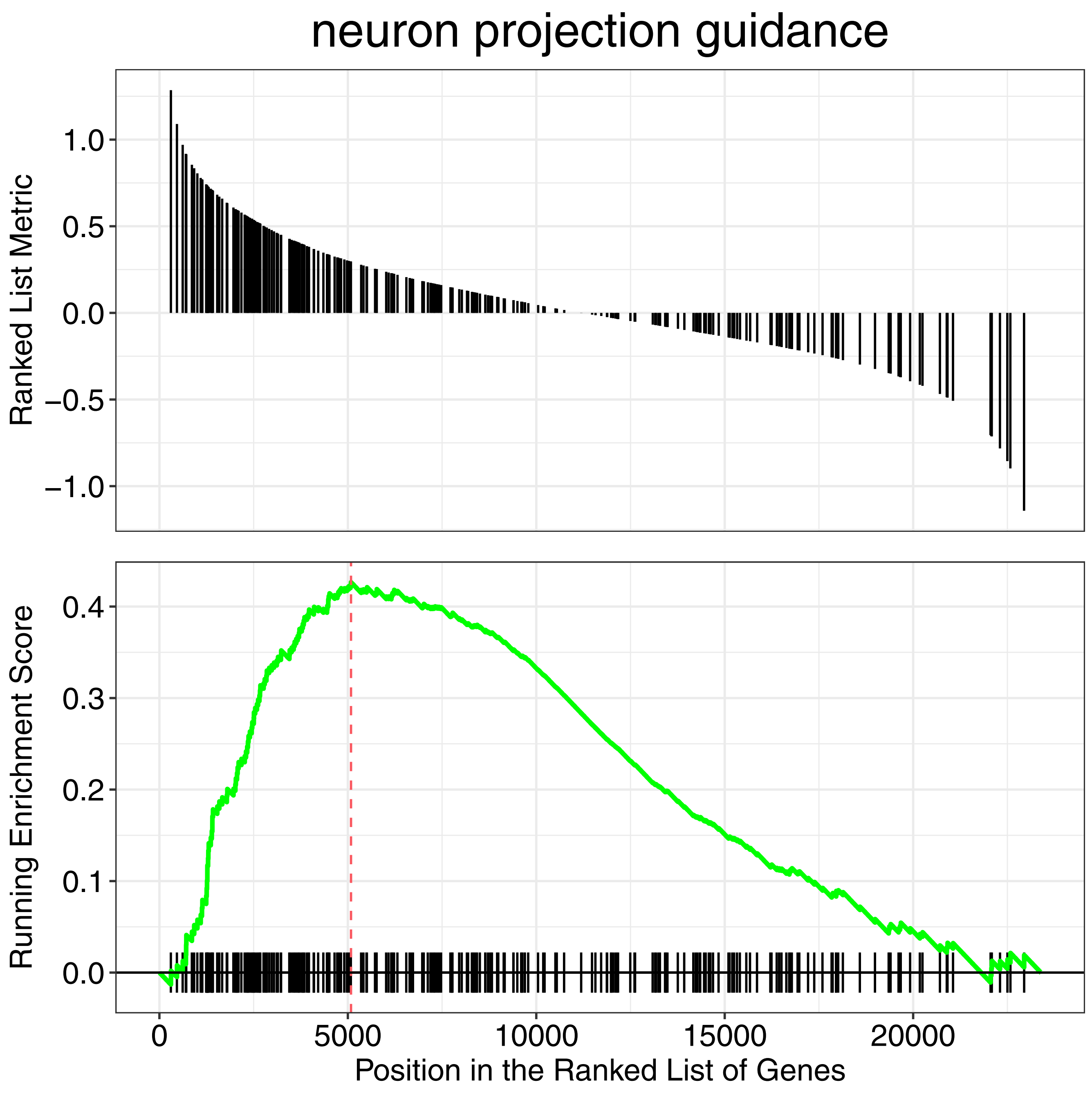

Biology Open • Supplementary information

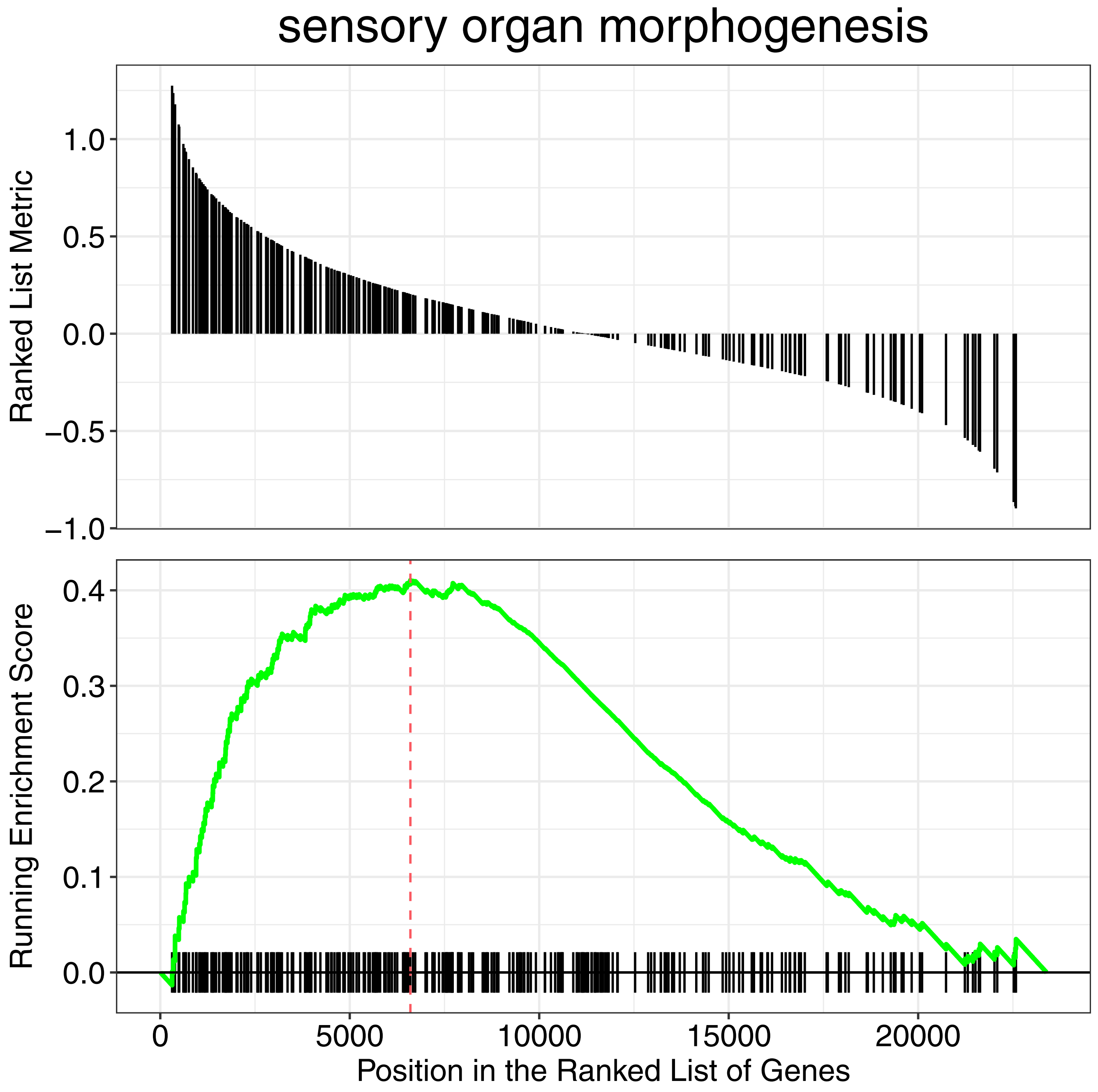

Biology Open • Supplementary information

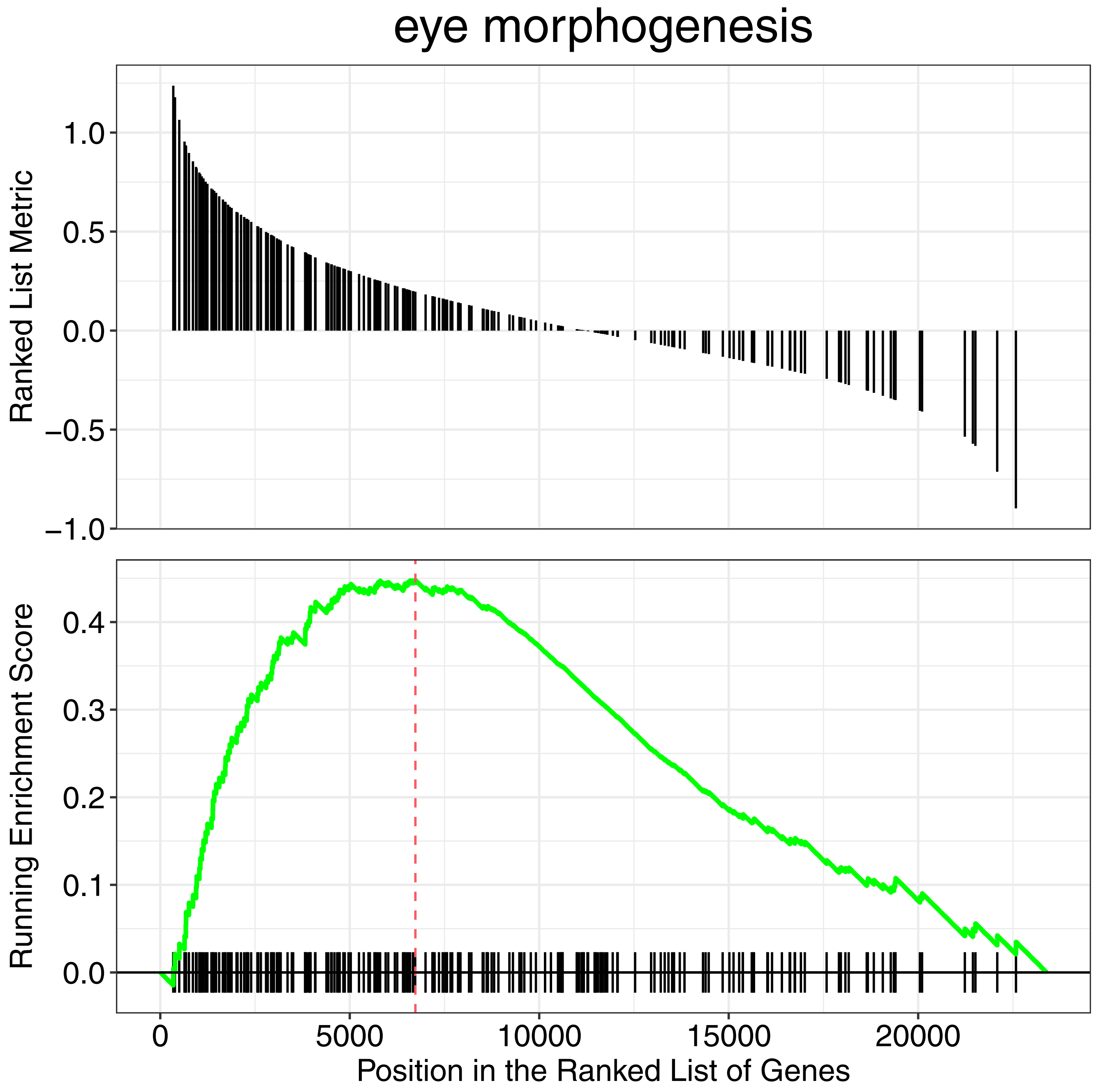

Biology Open • Supplementary information

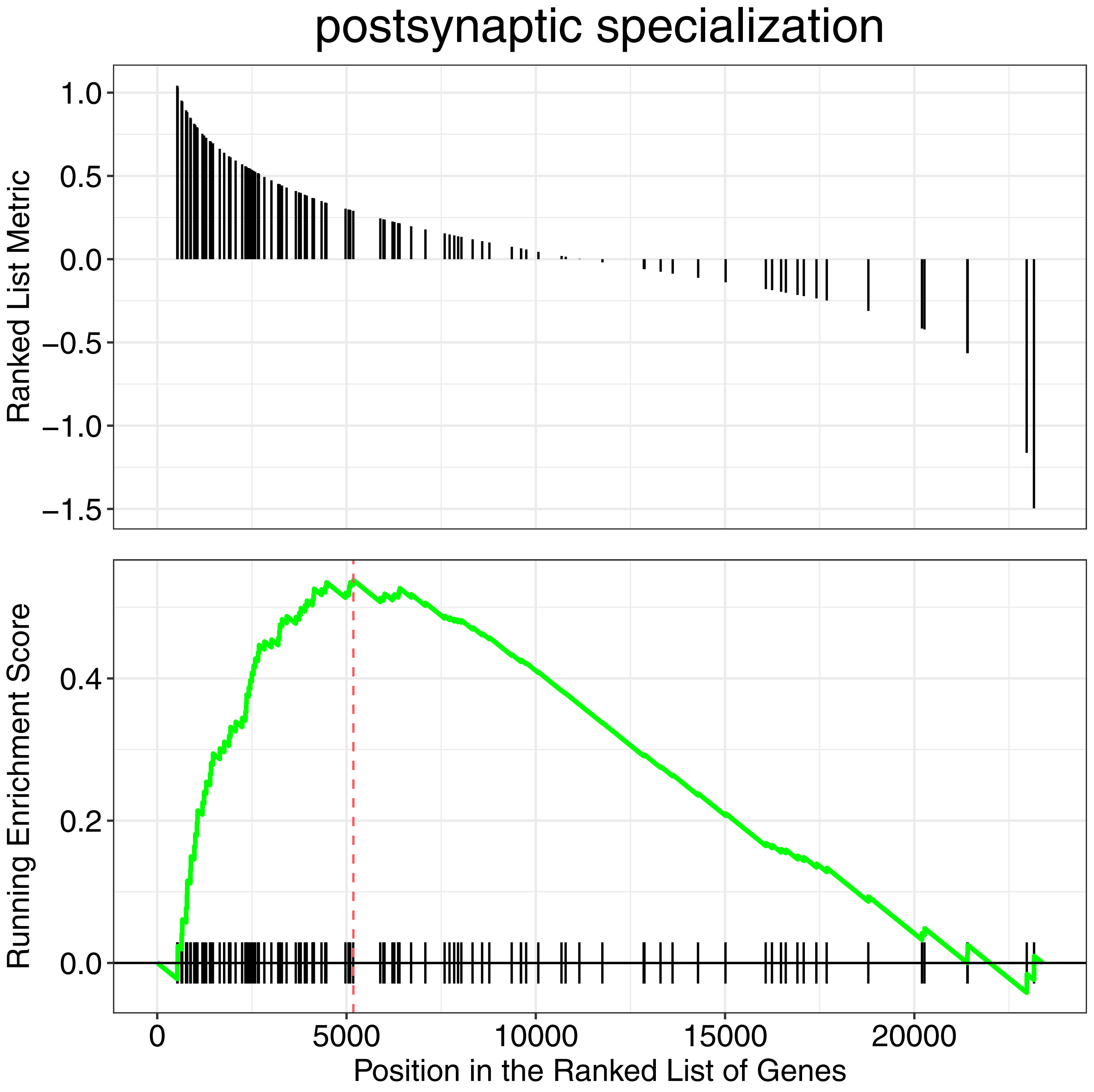

Biology Open • Supplementary information

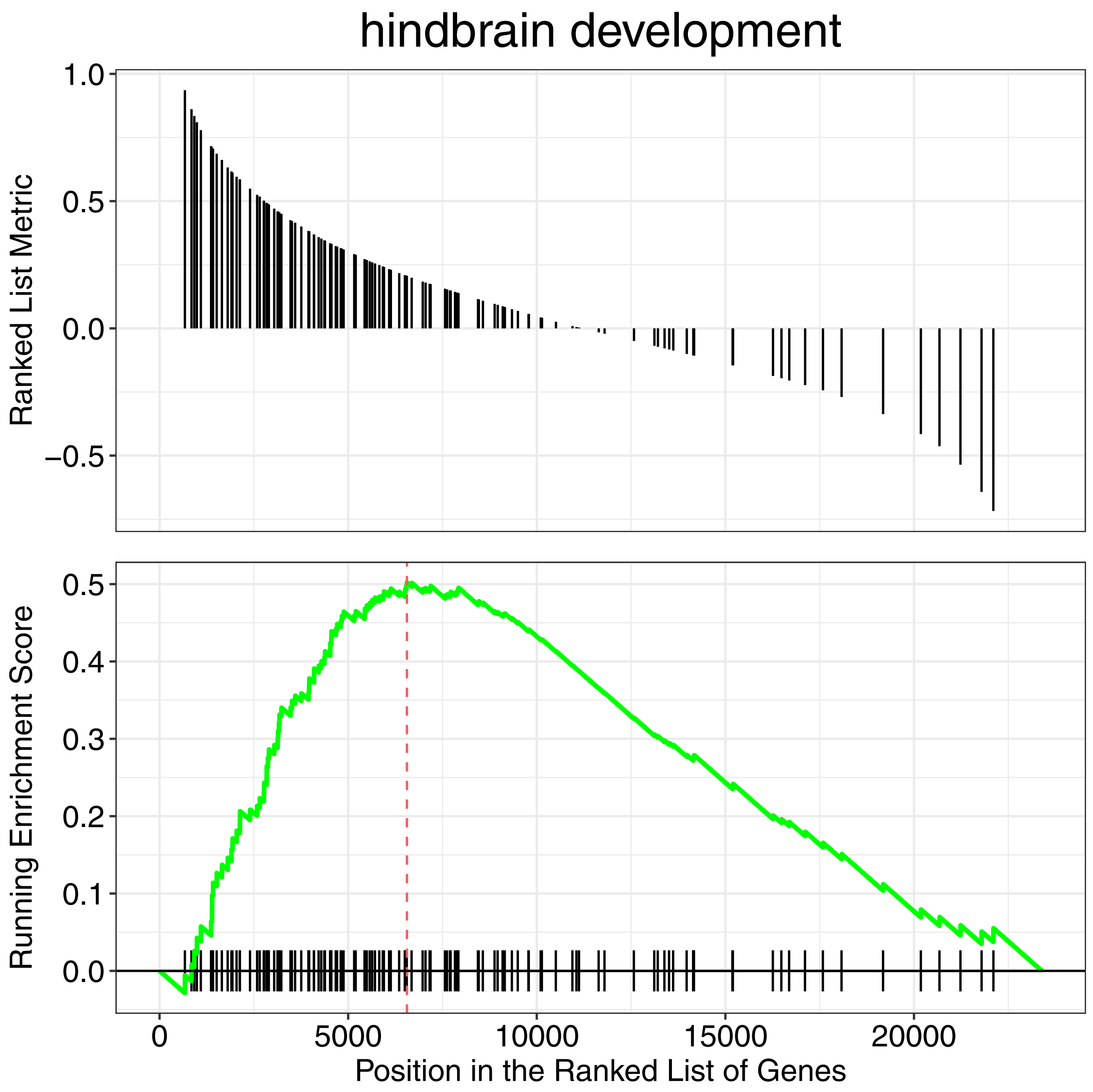

Fig. S5. Enrichment plots of Gene set enrichment analysis (GSEA) of differentially expressed genes in alx1;alx3 mutants. Running score plot and pre-ranked list of top 25 activated GSEA terms shown in Figure 5D.

Biology Open • Supplementary information

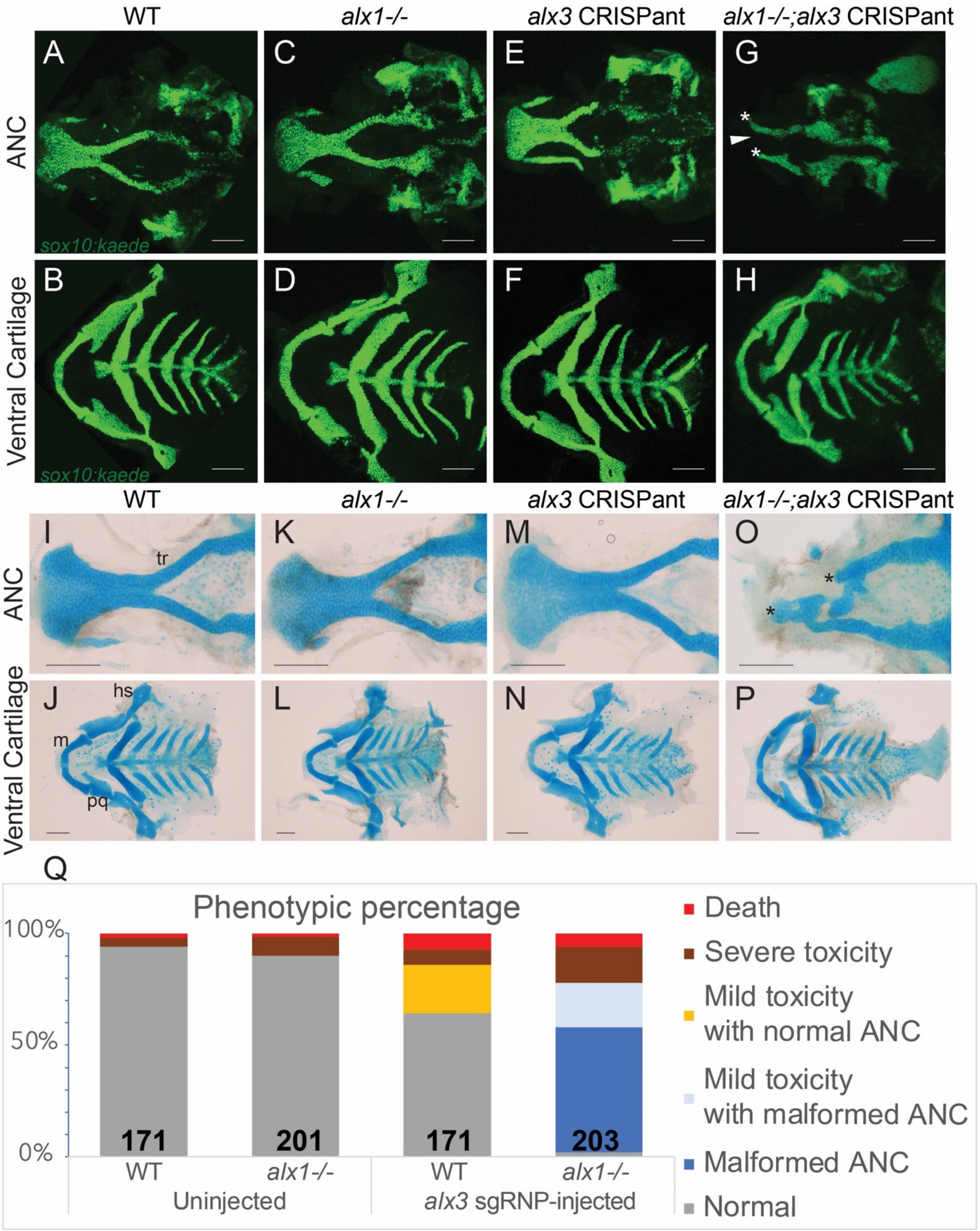

WT
alx1-/-
alx3 CRISPant
alx1-/-;alx3 CRISPant
ANC
Ventral Cartilage
A
B
C
D
E
F
G
H
sox10:kaede
I
J
K
L
M
N
O
P
tr
hs
m
pq
Q
Phenotypic percentage
100%
50%
0%
171
201
171
203
WT
alx1-/-
Uninjected
alx3 sgRNP-injected
Death
Severe toxicity
Mild toxicity with normal ANC
Mild toxicity with malformed ANC
Malformed ANC
Normal

Fig. S6. alx1; alx3CR phenocopy alx1; alx3 double mutants. Embryos derived from Tg(sox10:kaede) embryos or $alx1^{uw2016}$; Tg(sox10:kaede) were injected with alx3 CRISPR/Cas9 and fixed at 4 dpf. Embryos were dissected, and flat-mounted in order to image the anterior neurocranium (ANC) and ventral pharyngeal cartilage. *alx1; alx3CR* developed with midline cleft (arrowhead in G), with the lateral elements (asterisks in G) separated due to the missing median element. I – P: Dissected flat-mount Alcian Blue-stained cartilage in larvae of different genotypes, anterior to the left. I: WT embryo with normal paired trabeculae (tr) and a normal ANC. O: *alx1; alx3CR* with defective trabeculae fusion (asterisks) and depleted ethmoid plate. Q: Percentage of embryos displaying malformed anterior neurocranium (ANC) phenotype in larvae of different genotypes. All embryos are shown with anterior to the left. Scale bar: 100μm.

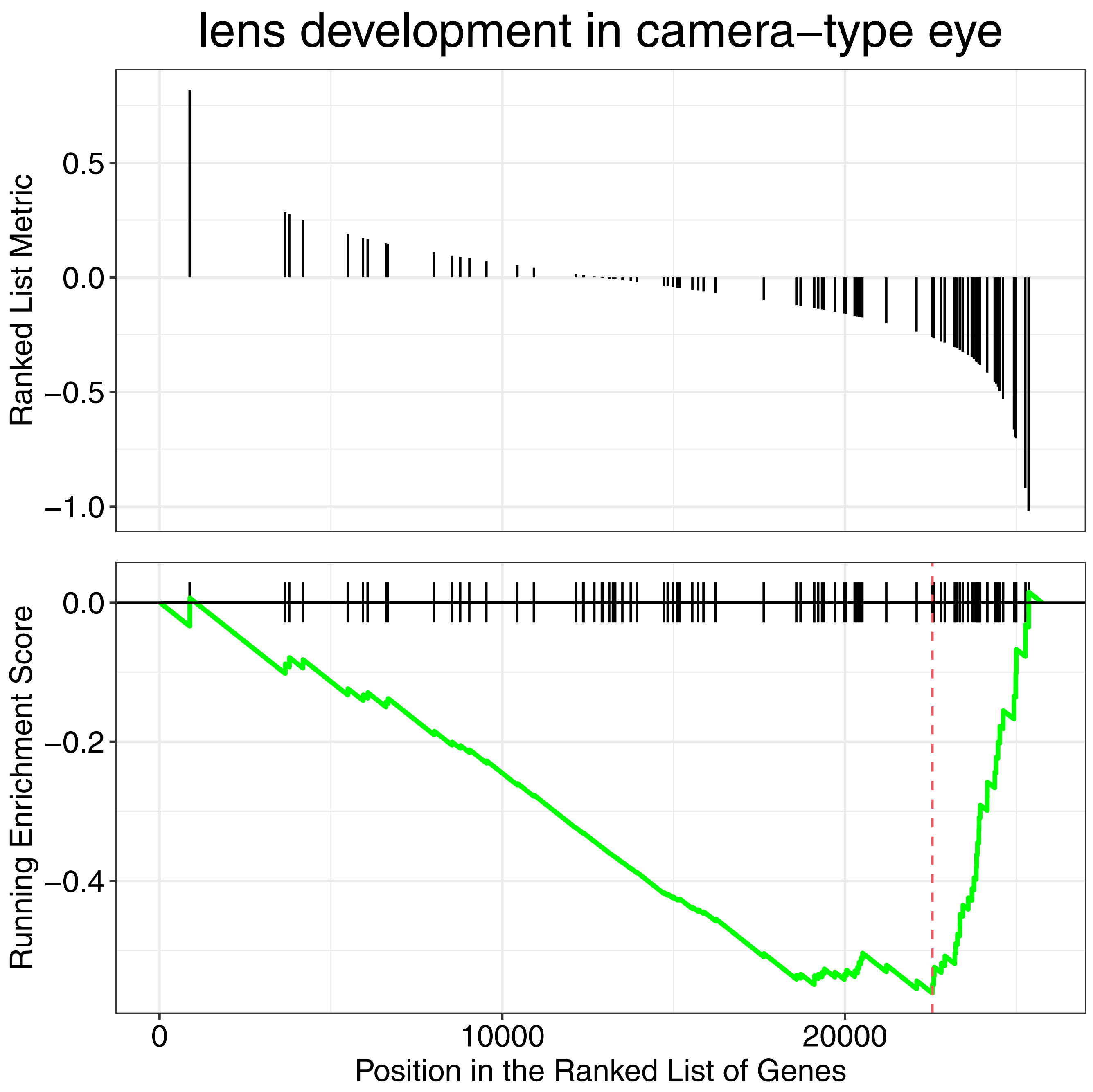

Biology Open • Supplementary information

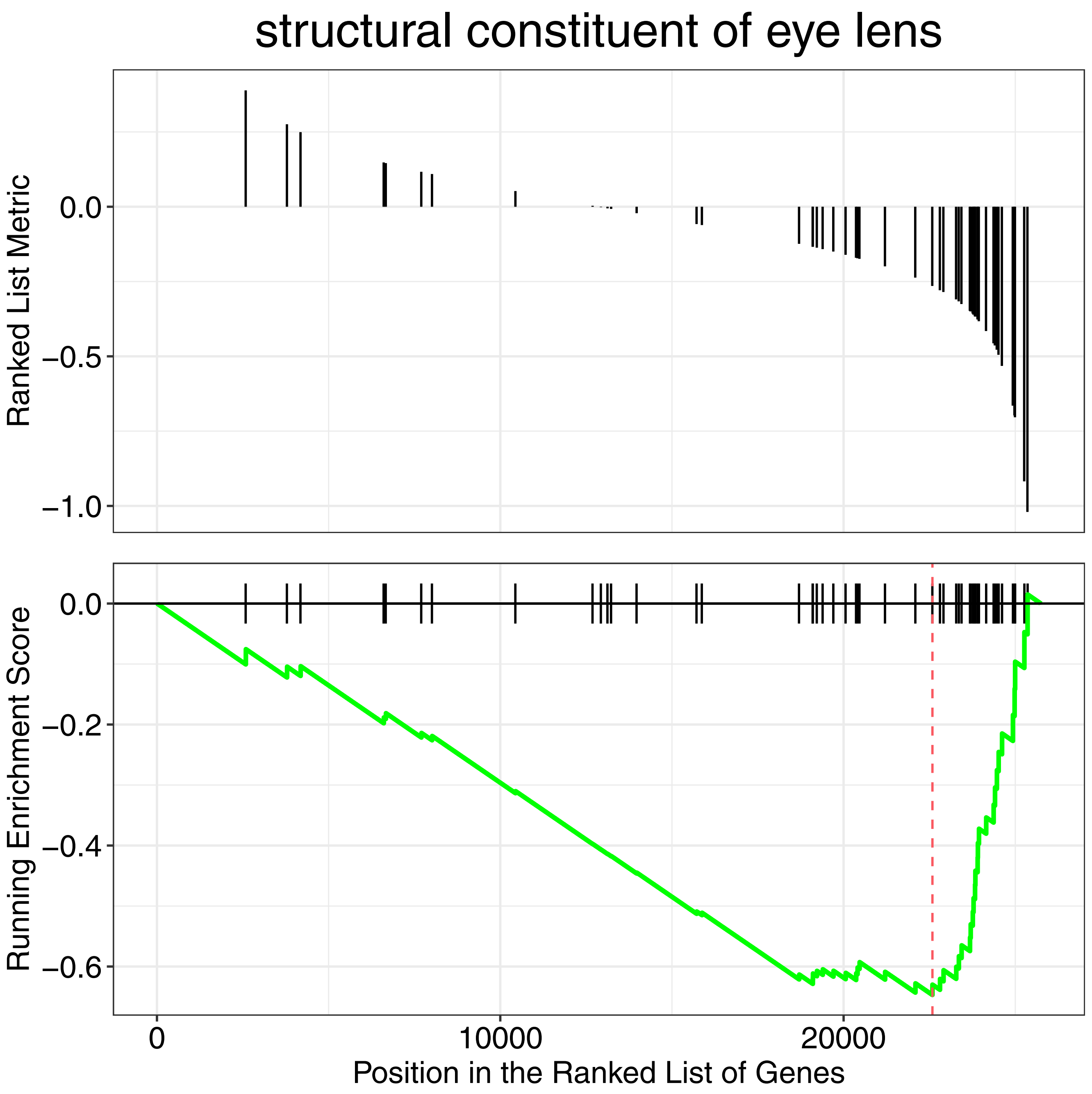

Biology Open • Supplementary information

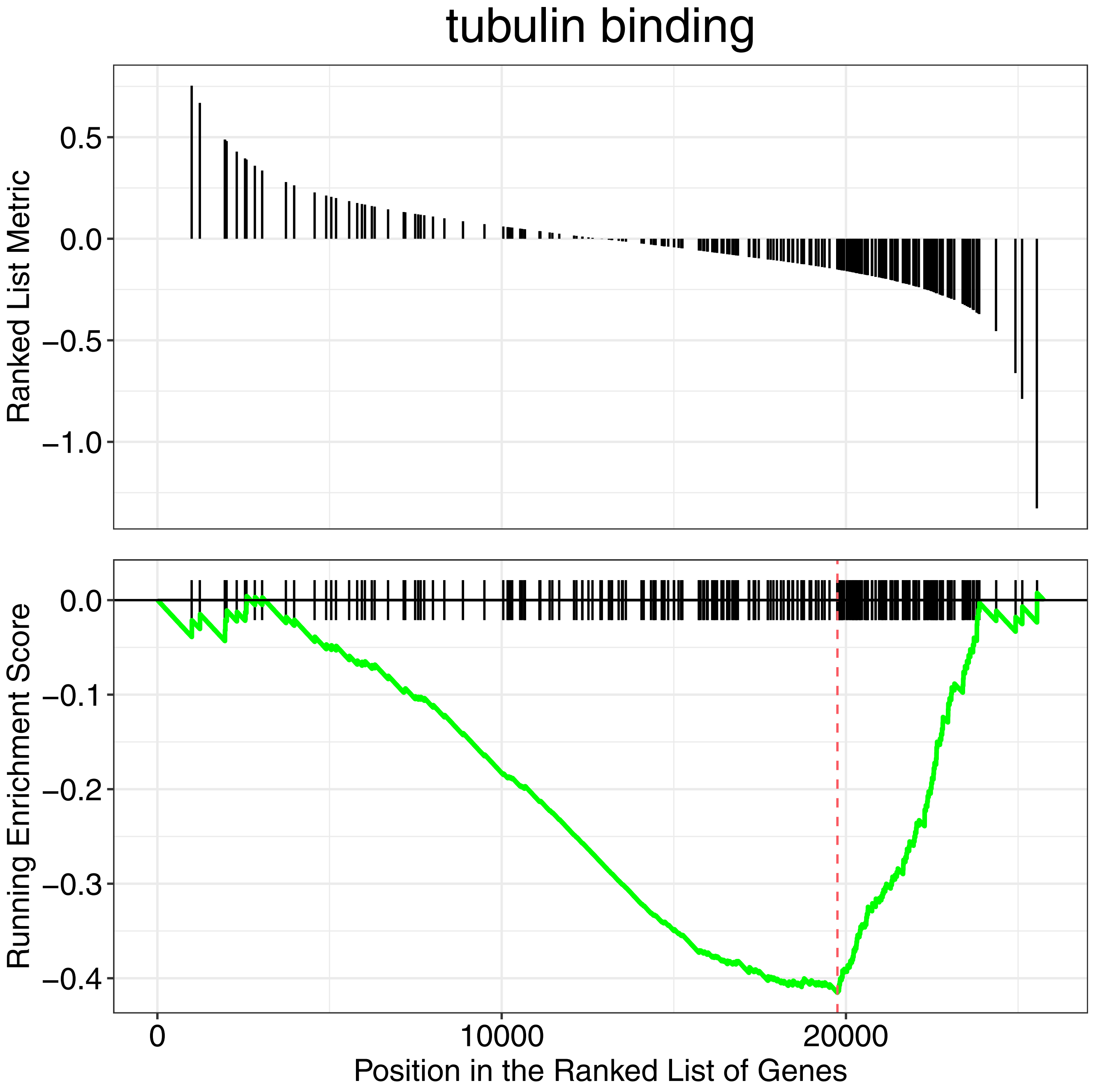

Biology Open • Supplementary information

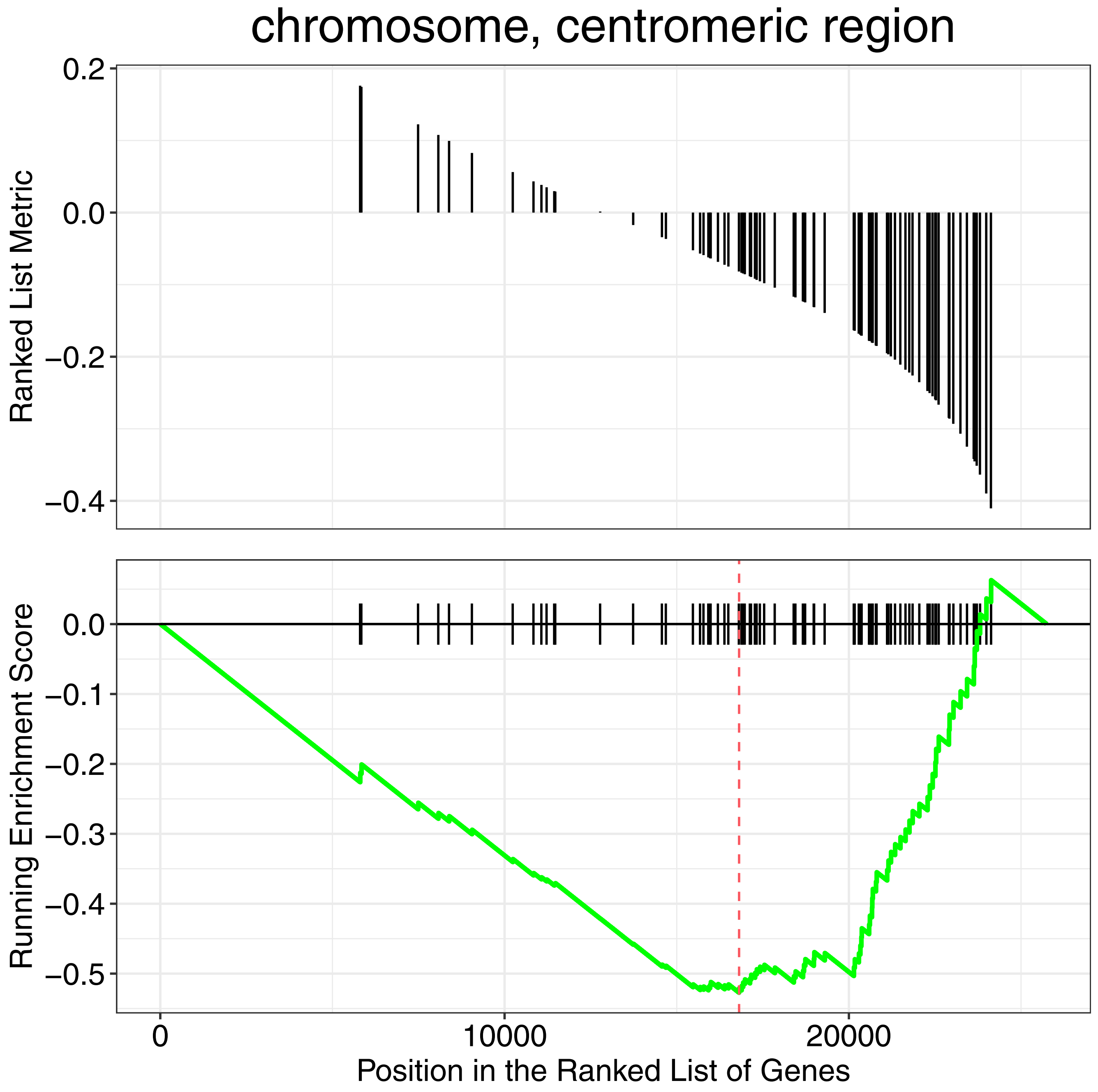
chromosome, centromeric region
Ranked List Metric
0.2
0.0
−0.2
−0.4
Running Enrichment Score
0.0
−0.1
−0.2
−0.3
−0.4
−0.5
0
10000
20000
Position in the Ranked List of Genes

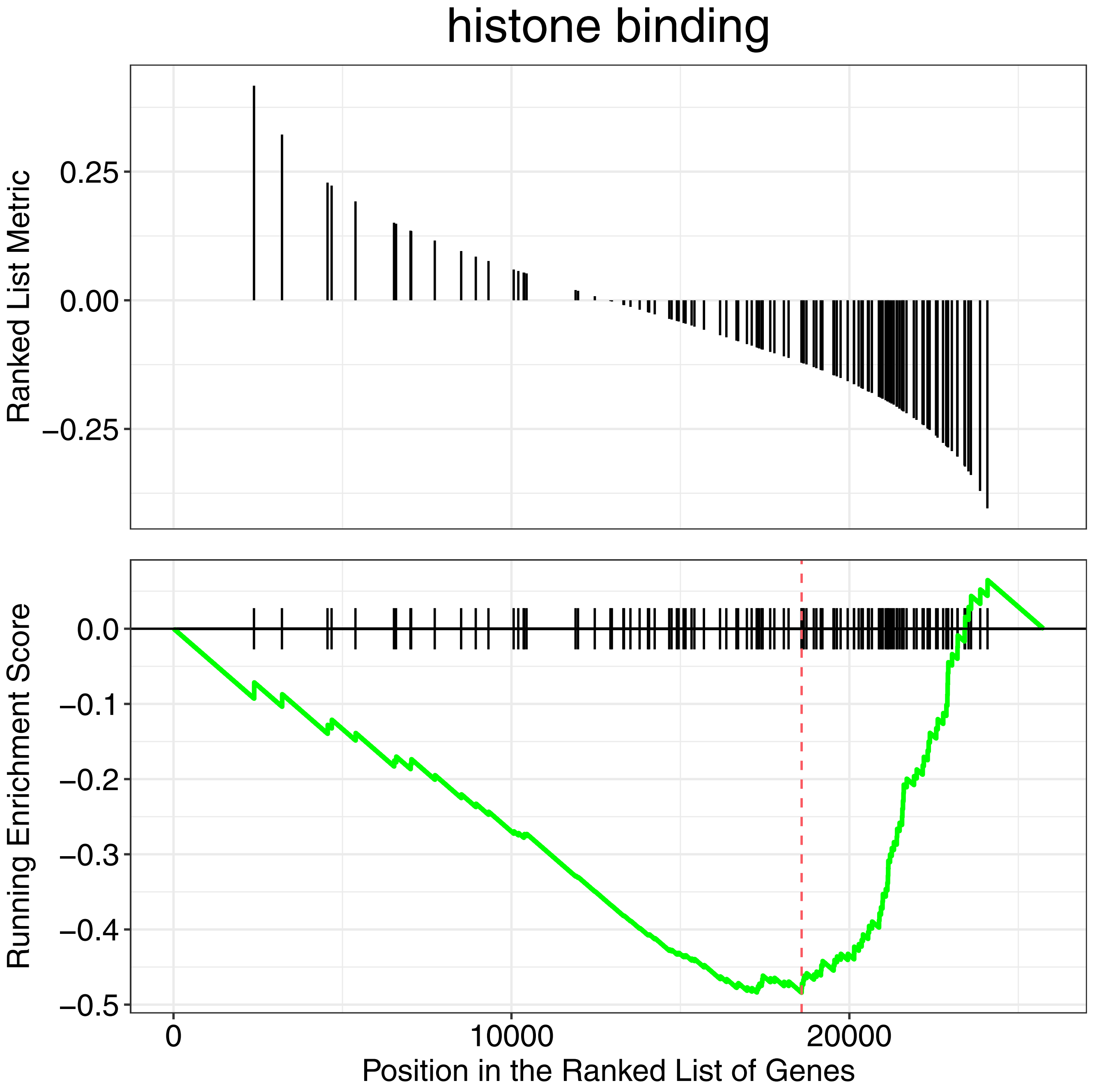

Biology Open • Supplementary information

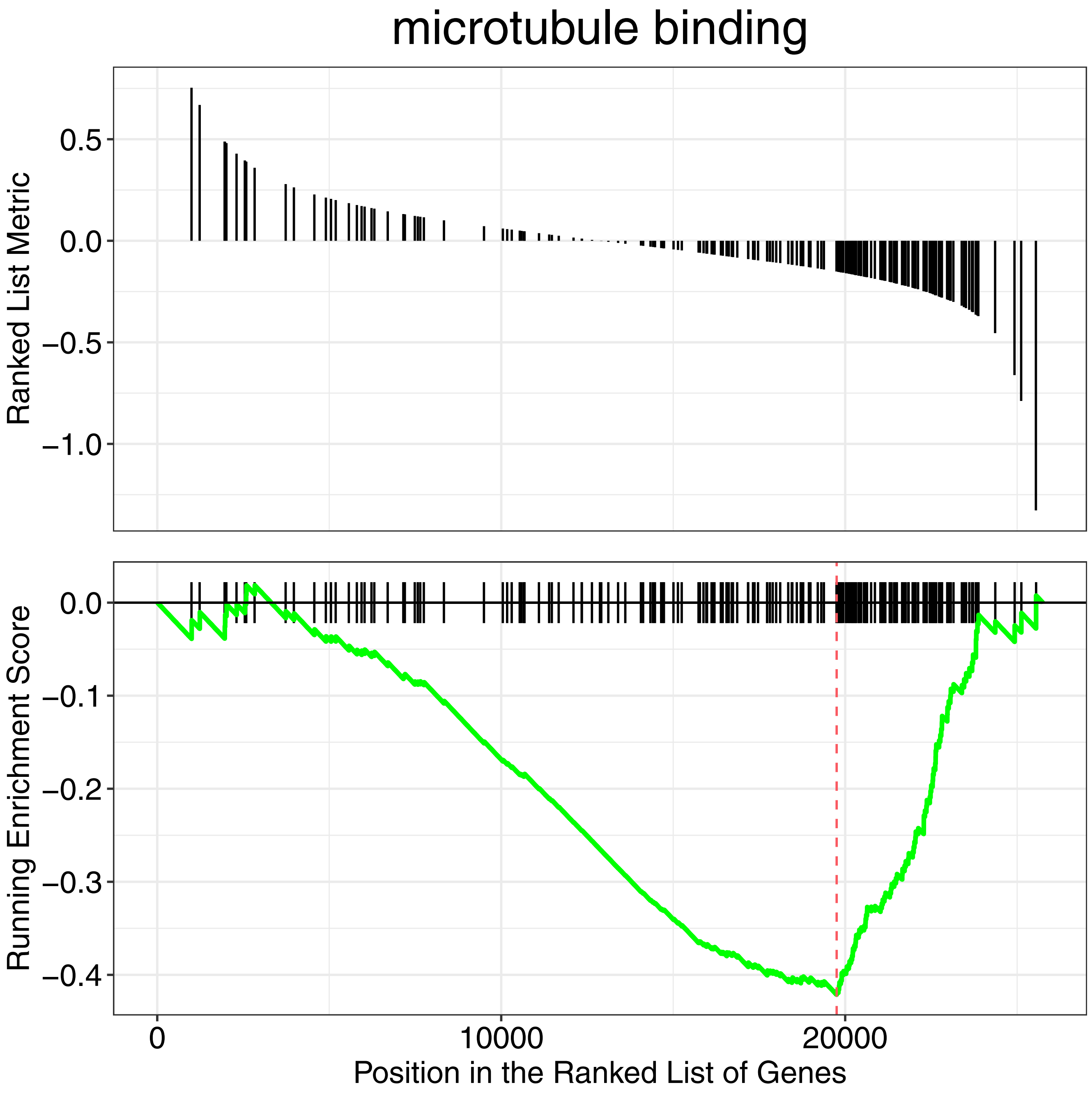

Biology Open • Supplementary information

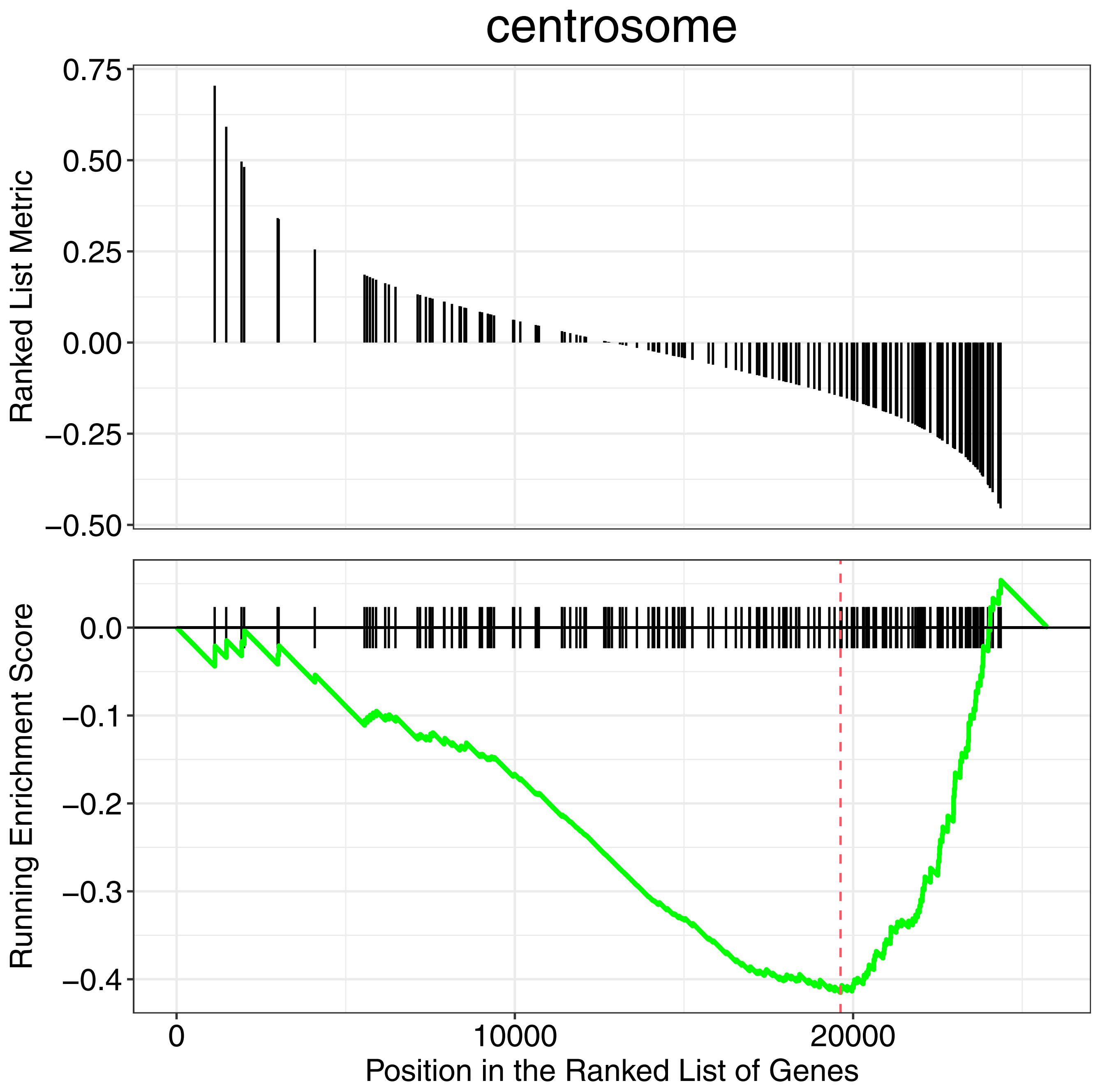

Biology Open • Supplementary information

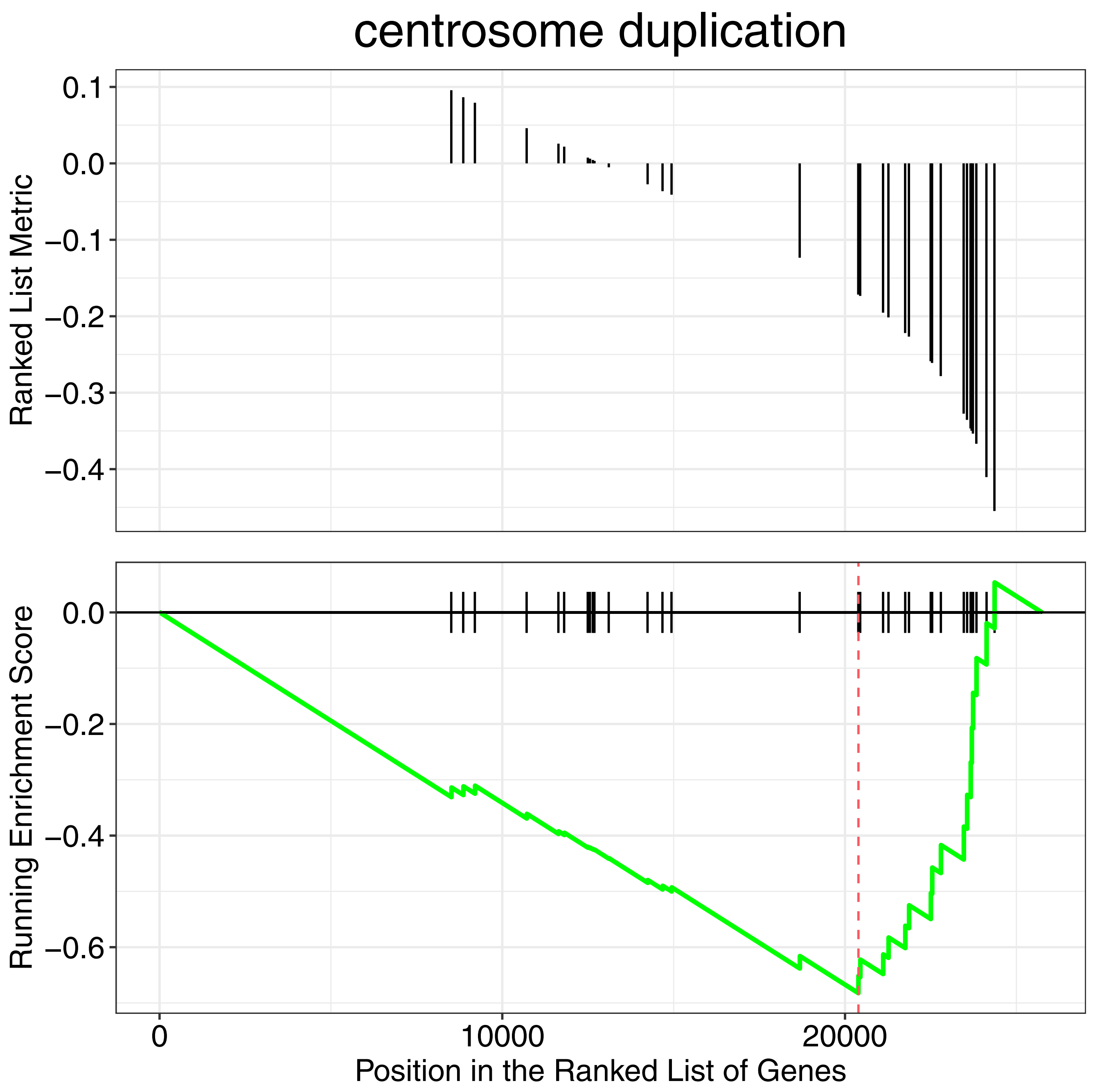

Biology Open • Supplementary information

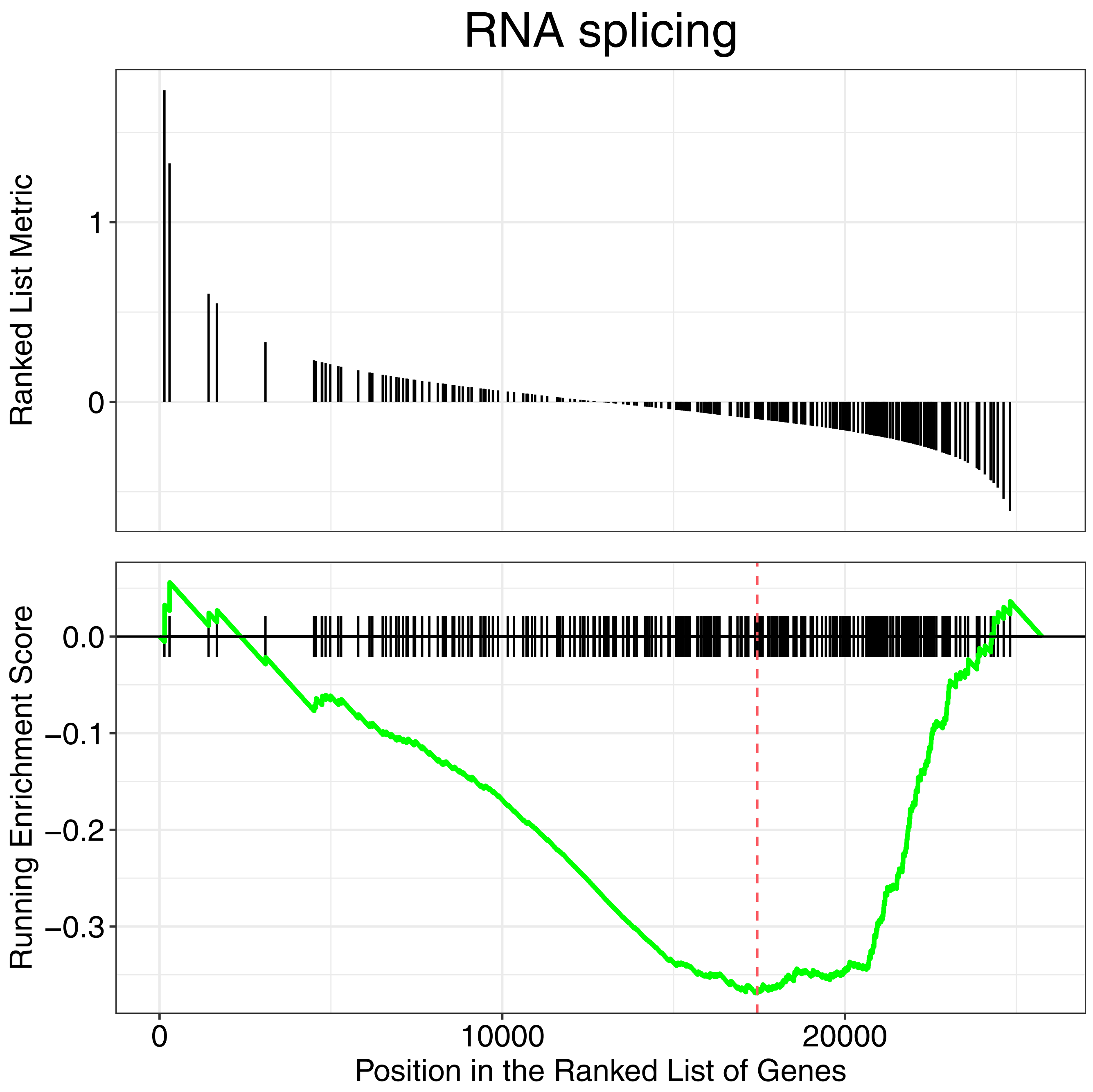

Biology Open • Supplementary information

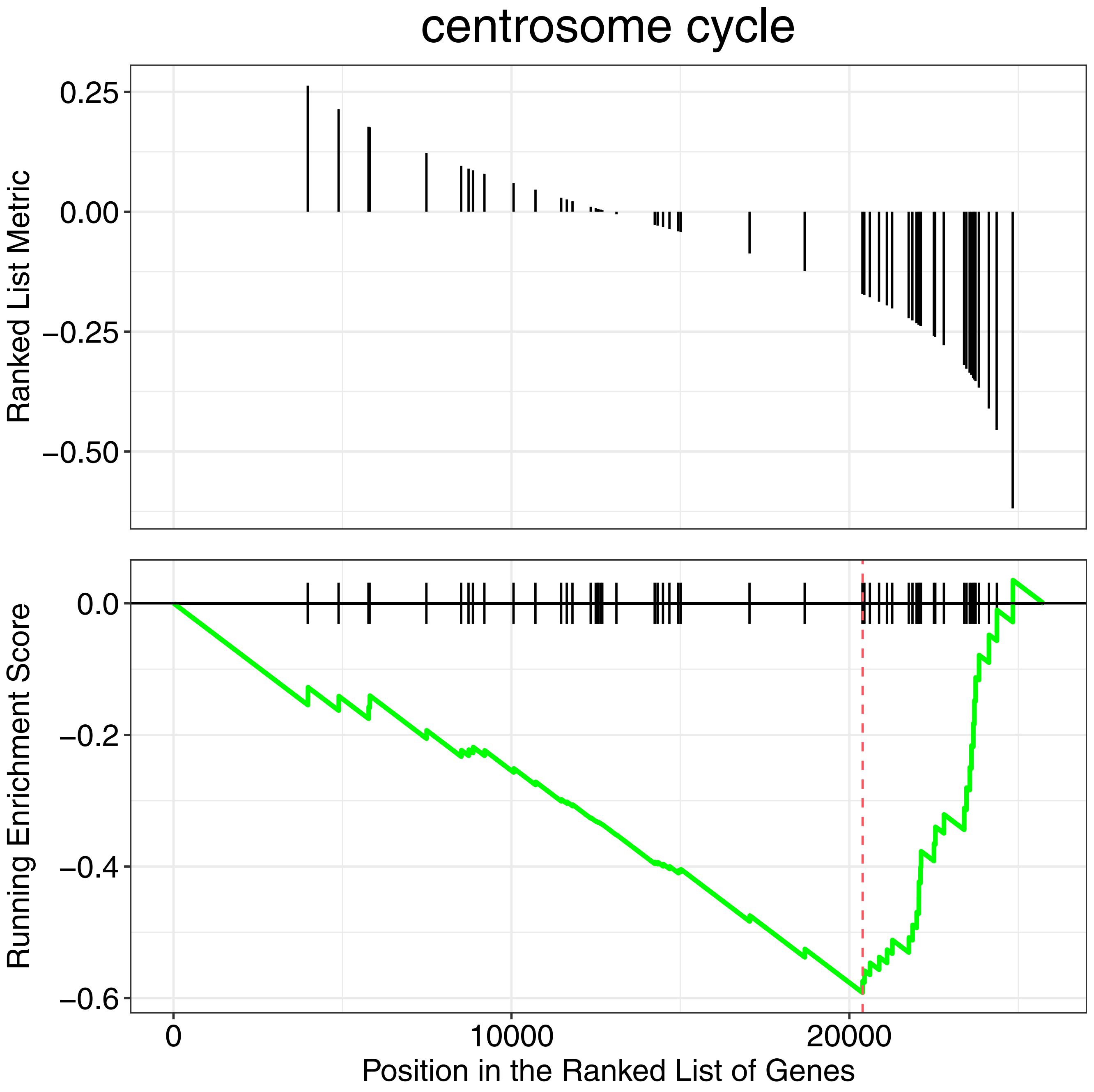

Biology Open • Supplementary information

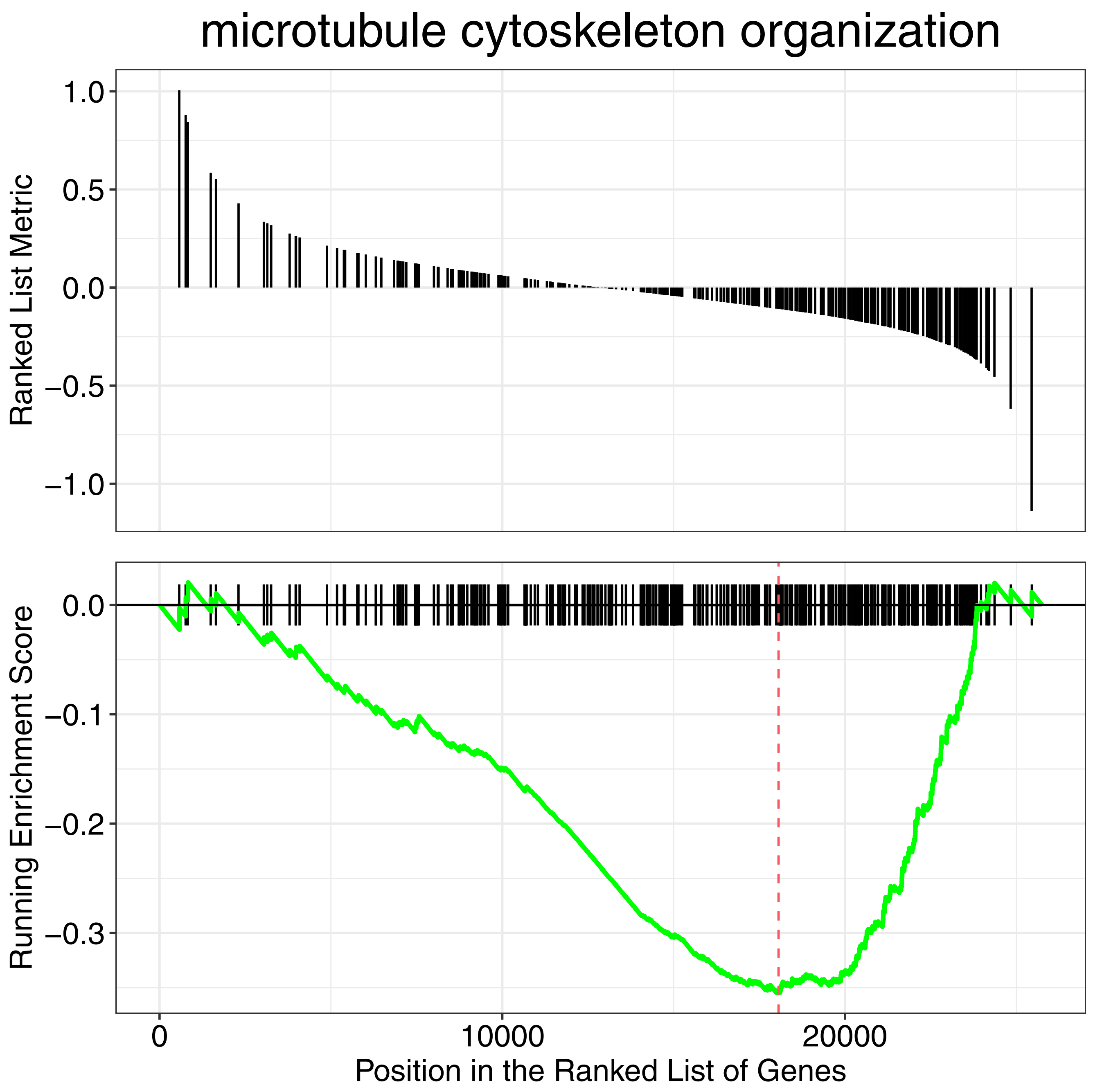

Biology Open • Supplementary information

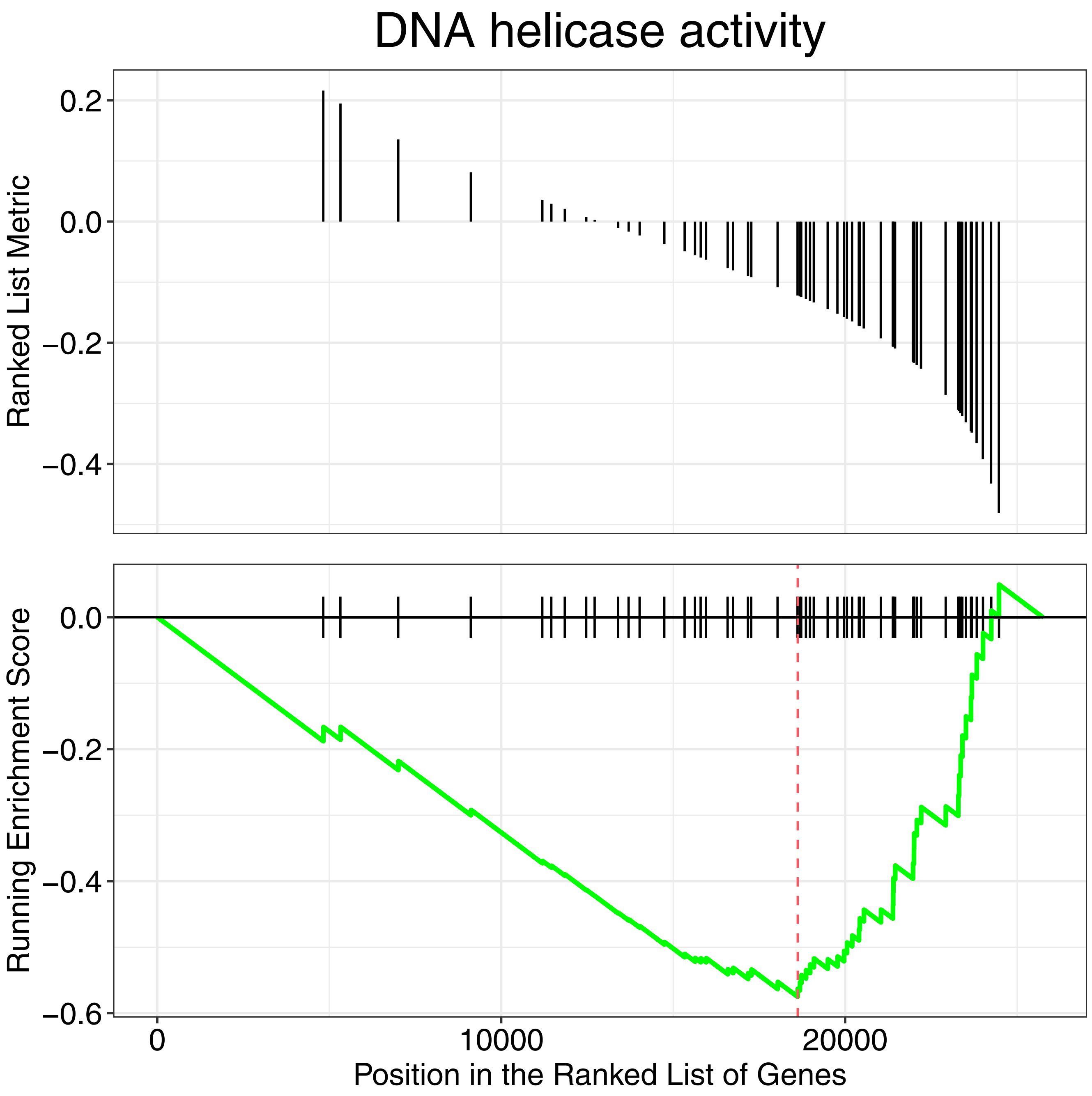

Biology Open • Supplementary information

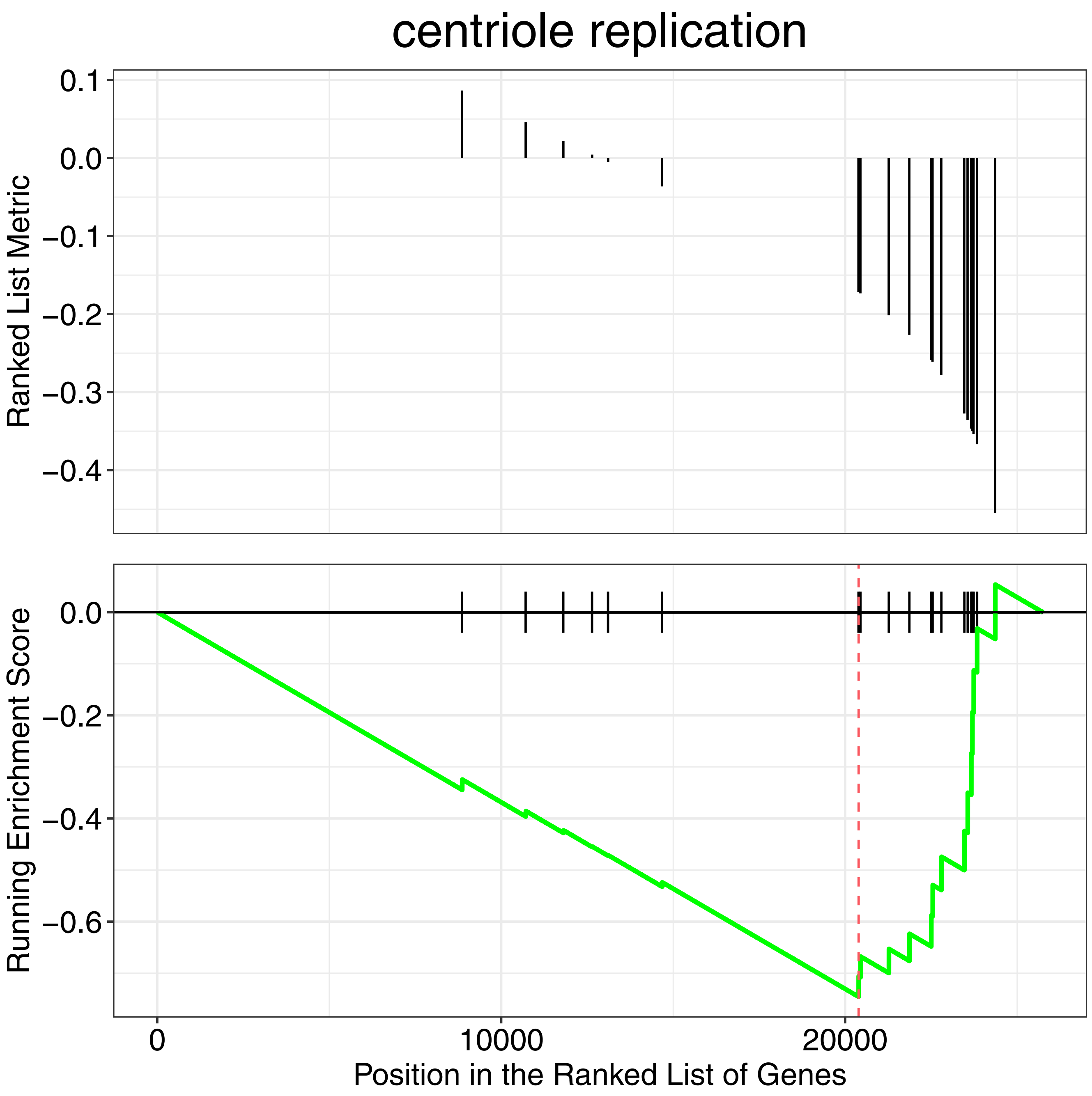

Biology Open • Supplementary information

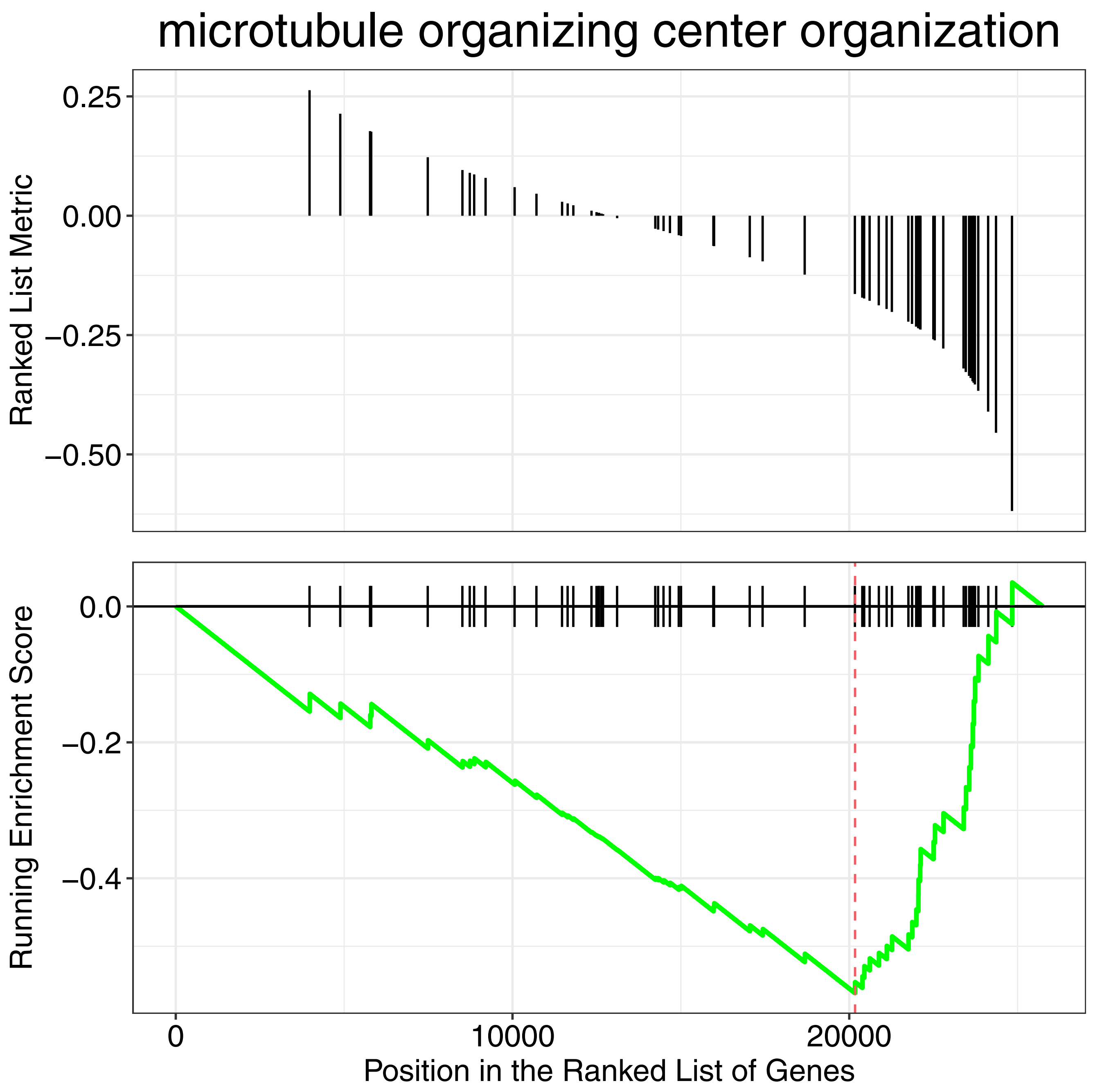

Biology Open • Supplementary information

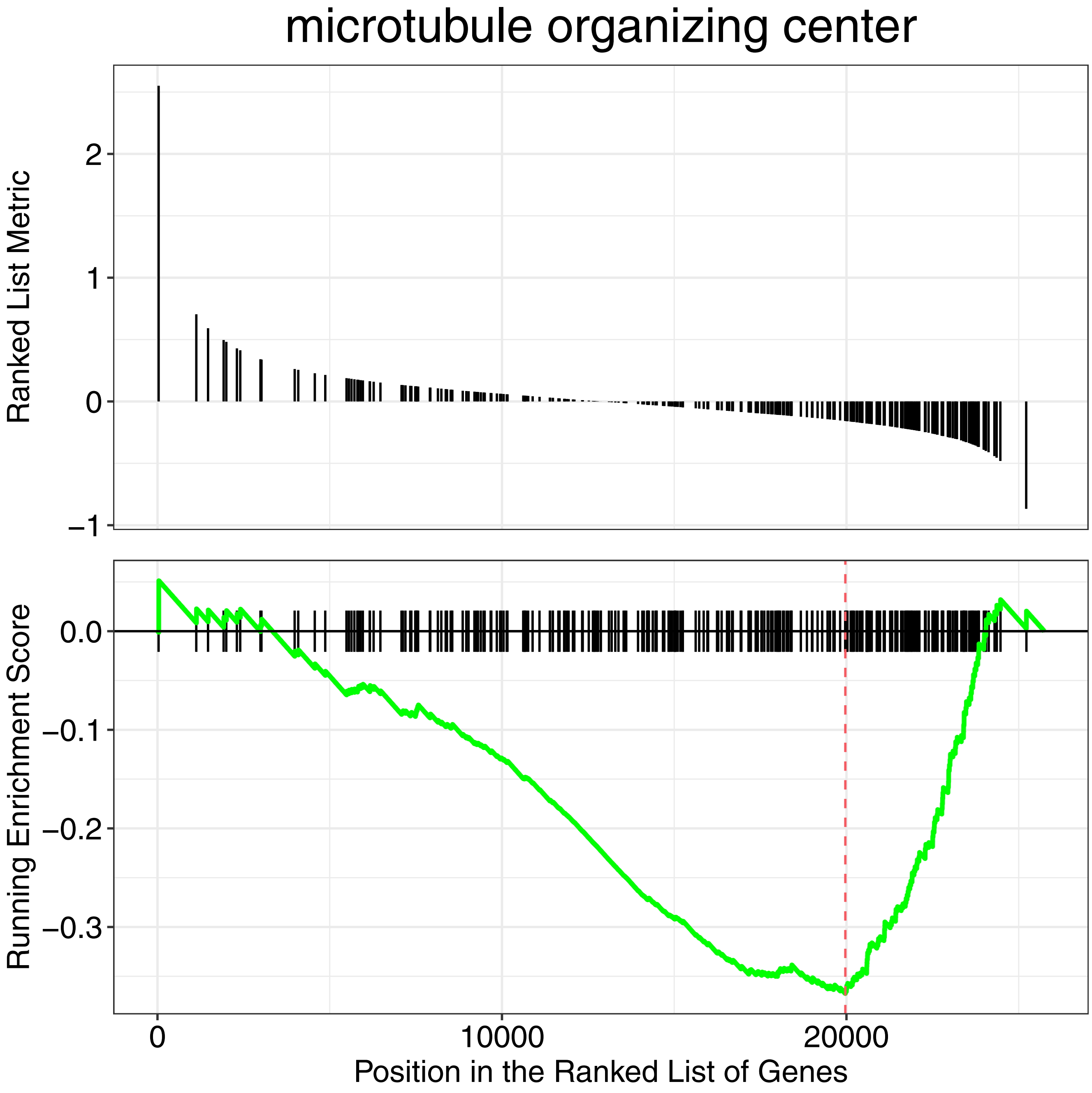

Biology Open • Supplementary information

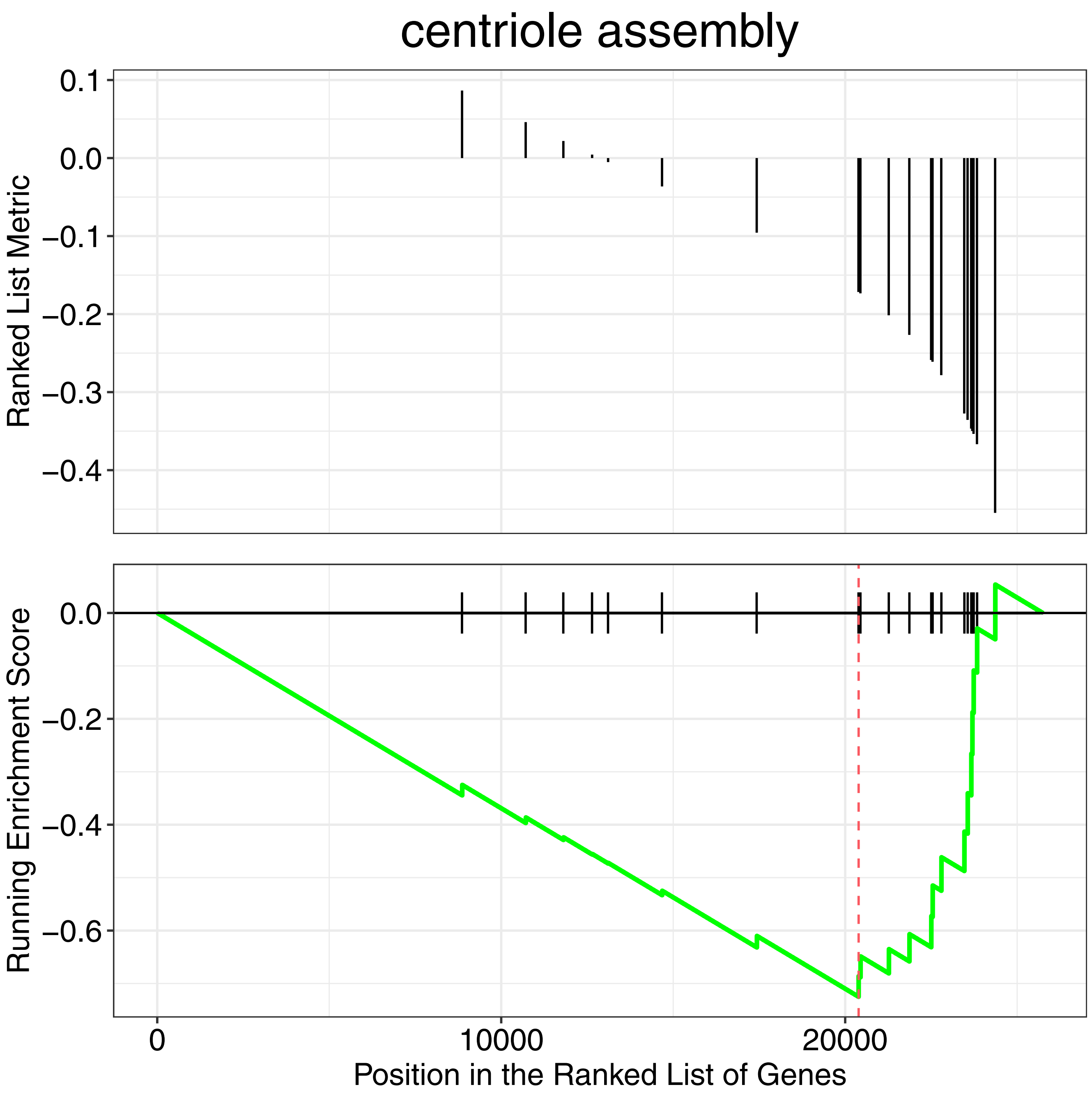

Fig. S7. Enrichment plots of Gene set enrichment analysis (GSEA) of differentially expressed genes in alx1;alx3 crispants. Running score plot and pre-ranked list of all suppressed GSEA terms shown in alx1;alx3 crispants.

Biology Open • Supplementary information

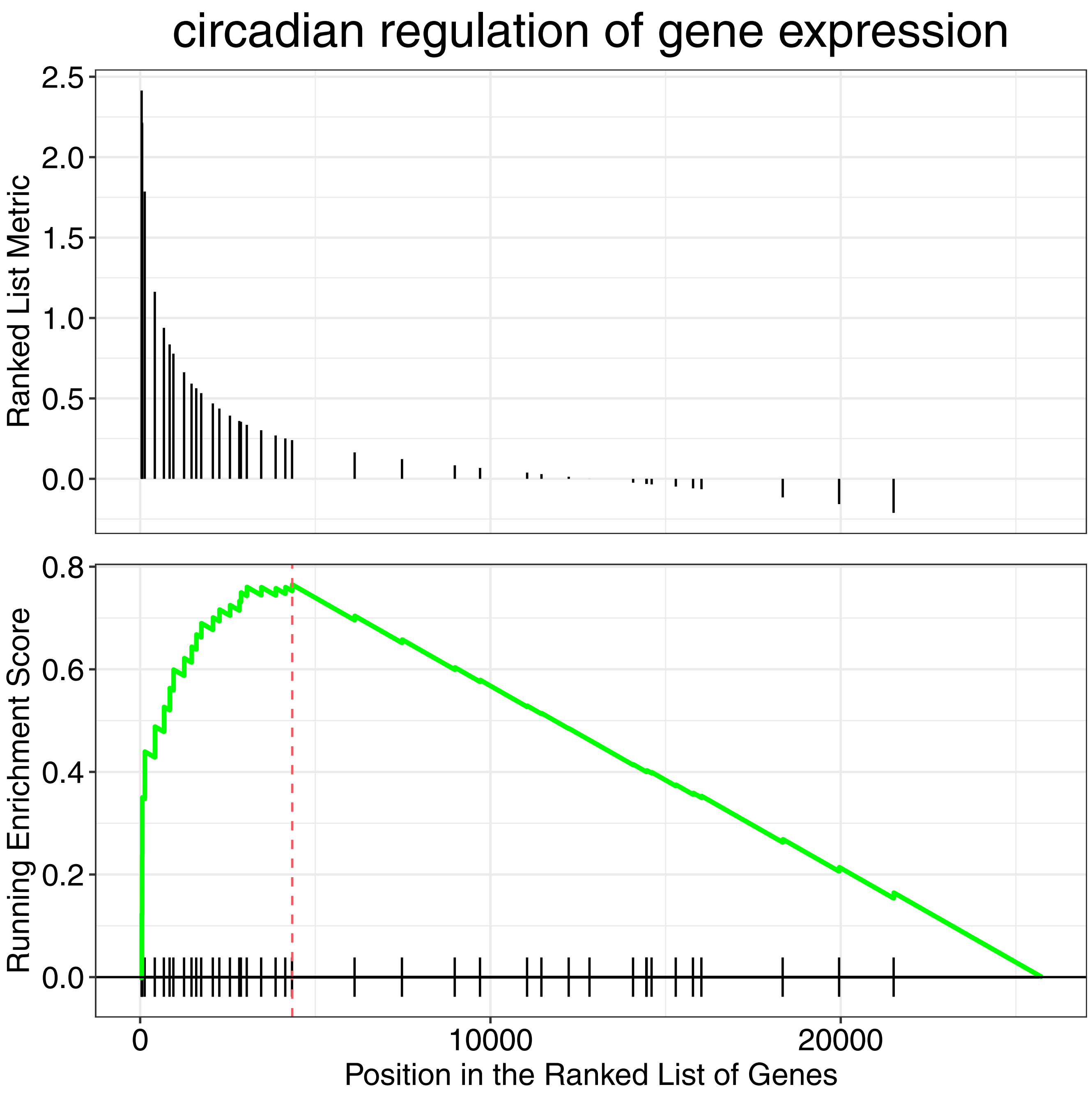

Biology Open • Supplementary information

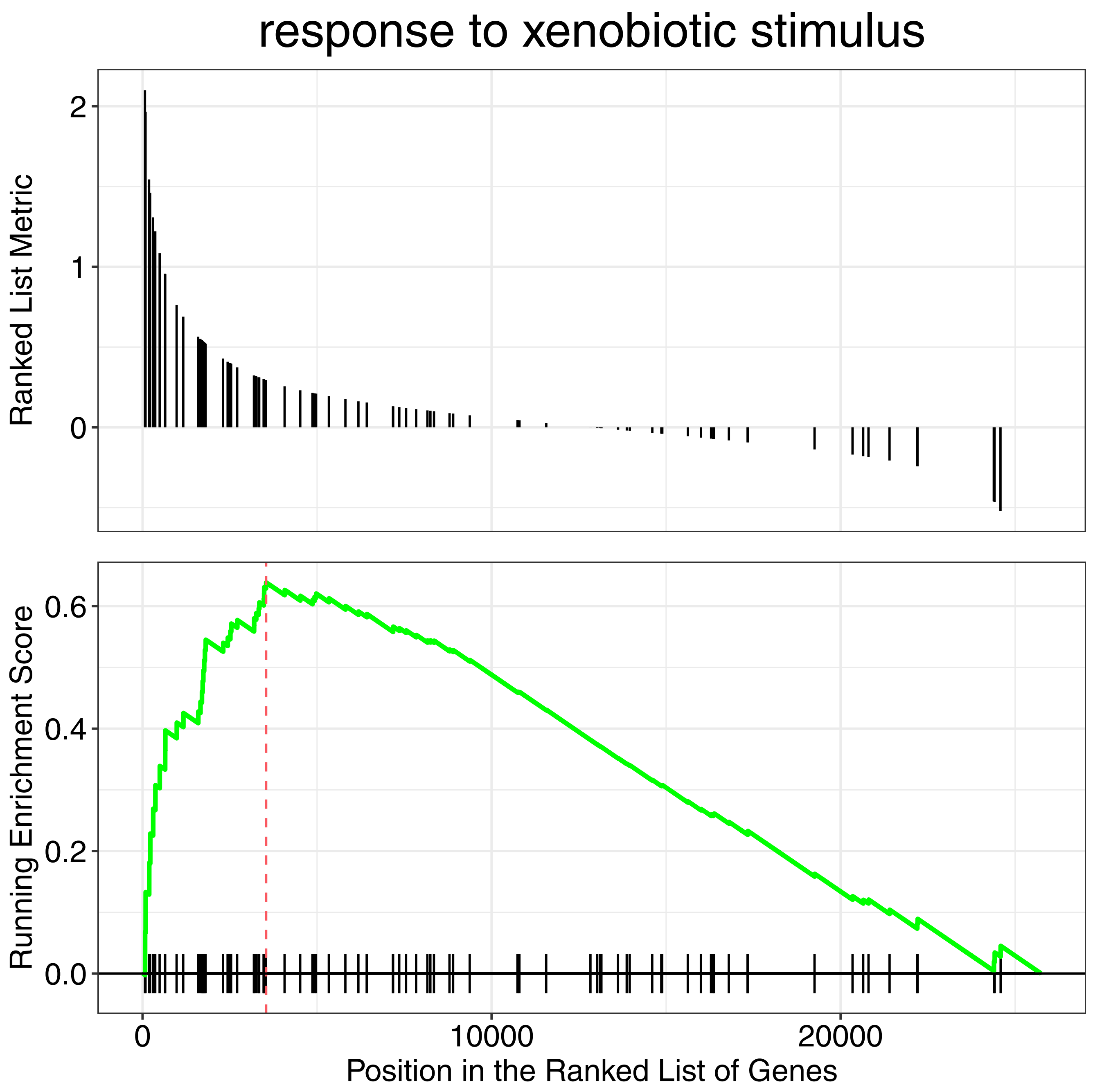

Biology Open • Supplementary information

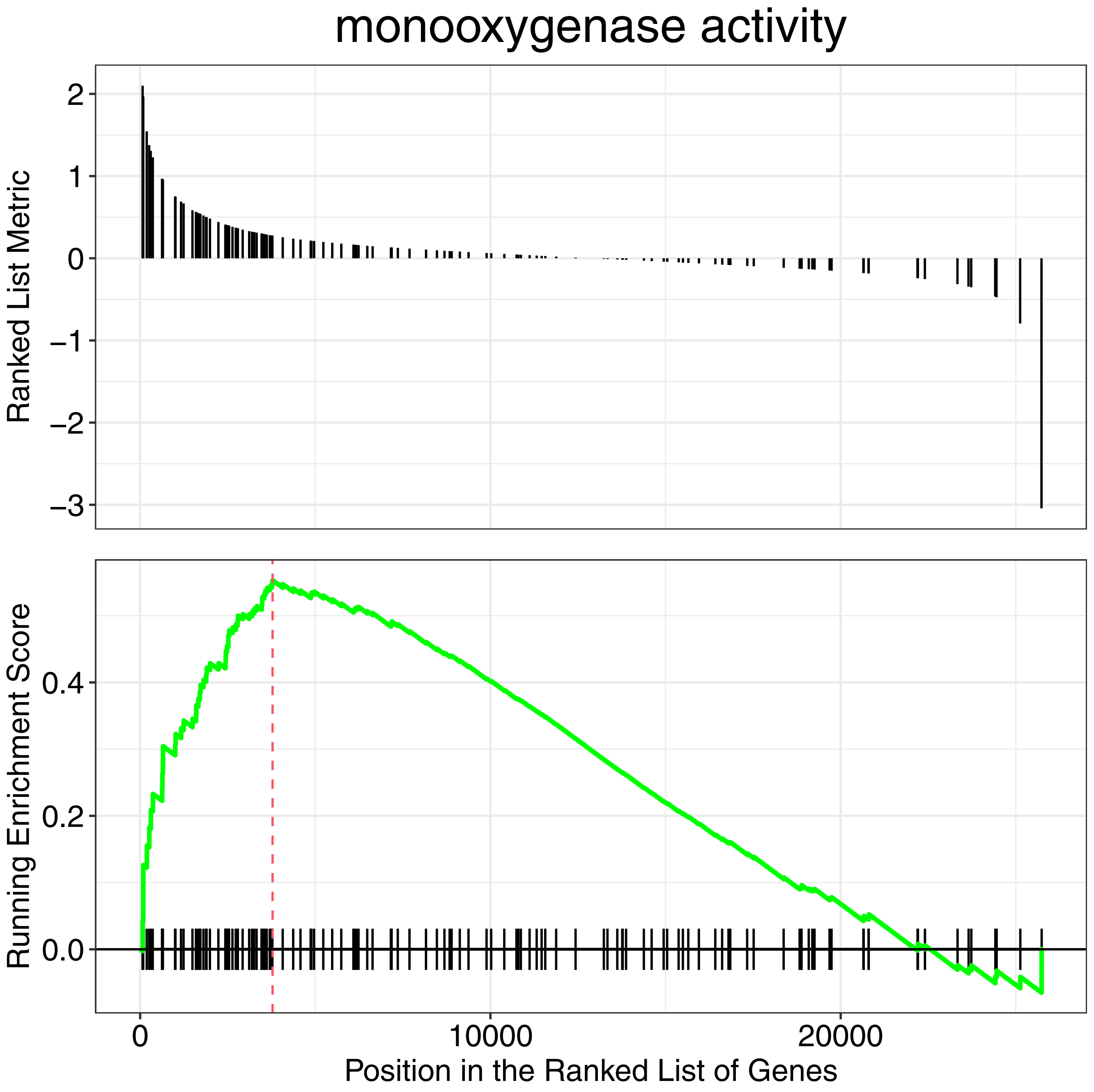

Biology Open • Supplementary information

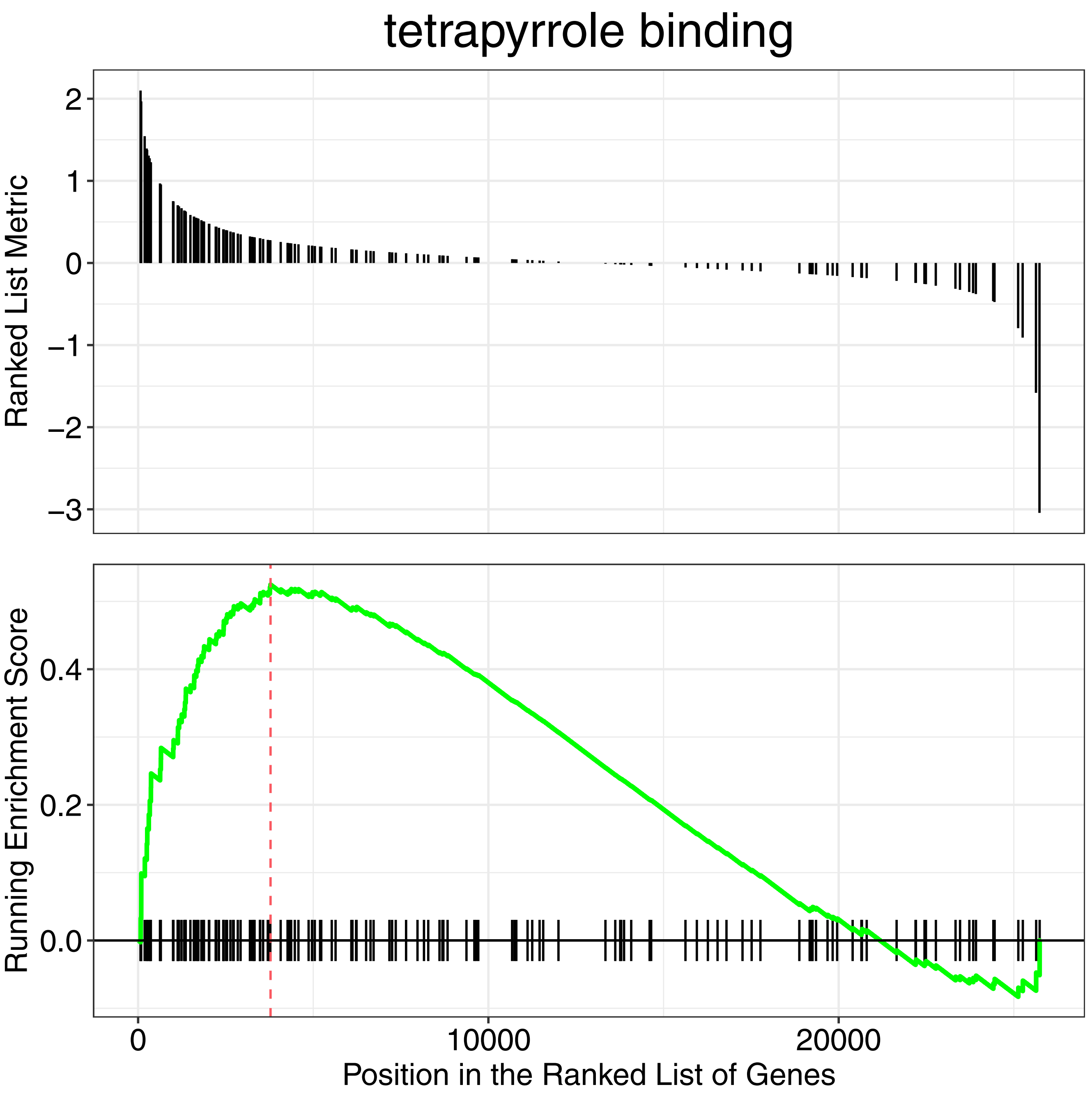

Biology Open • Supplementary information

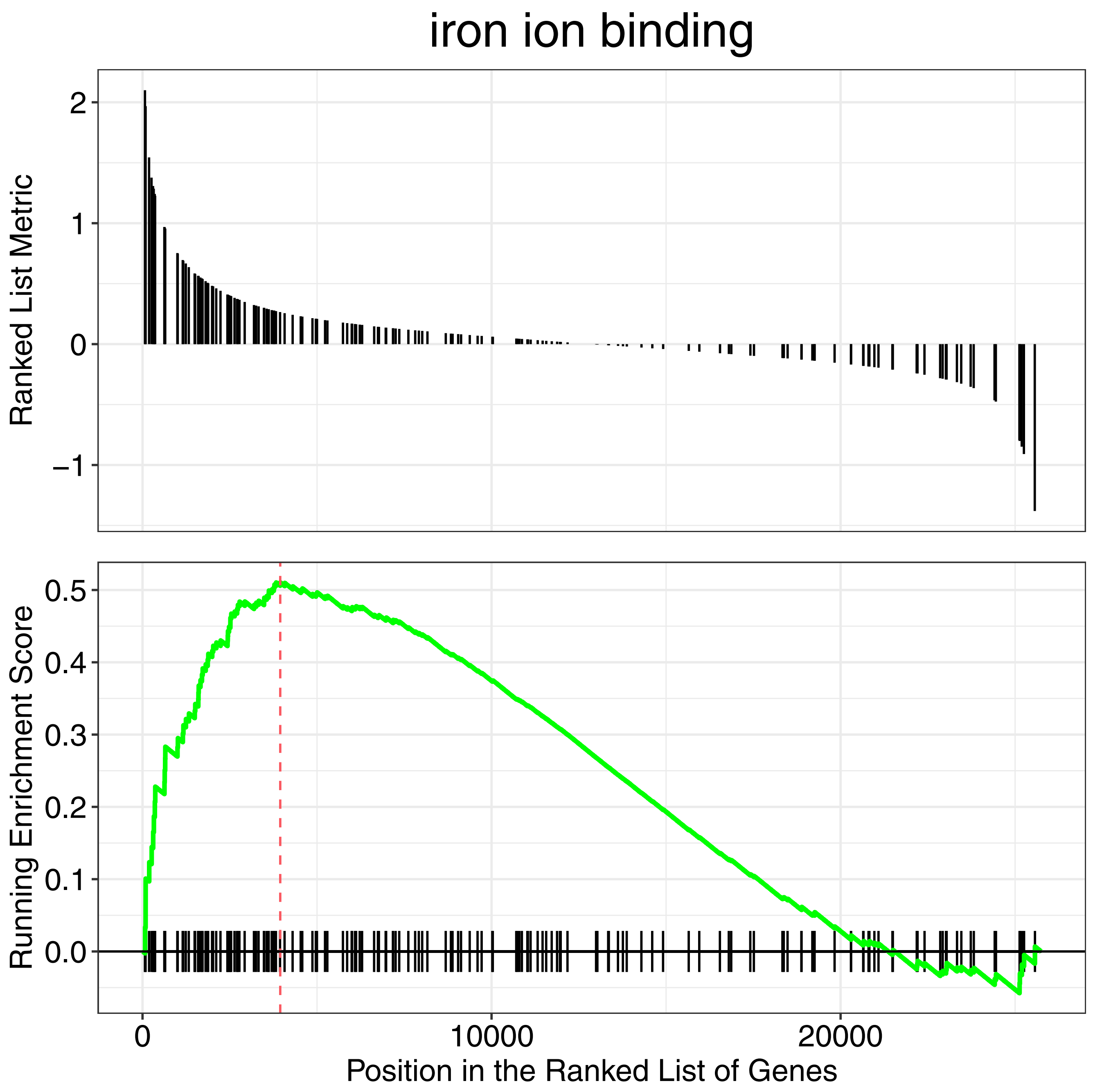

Biology Open • Supplementary information

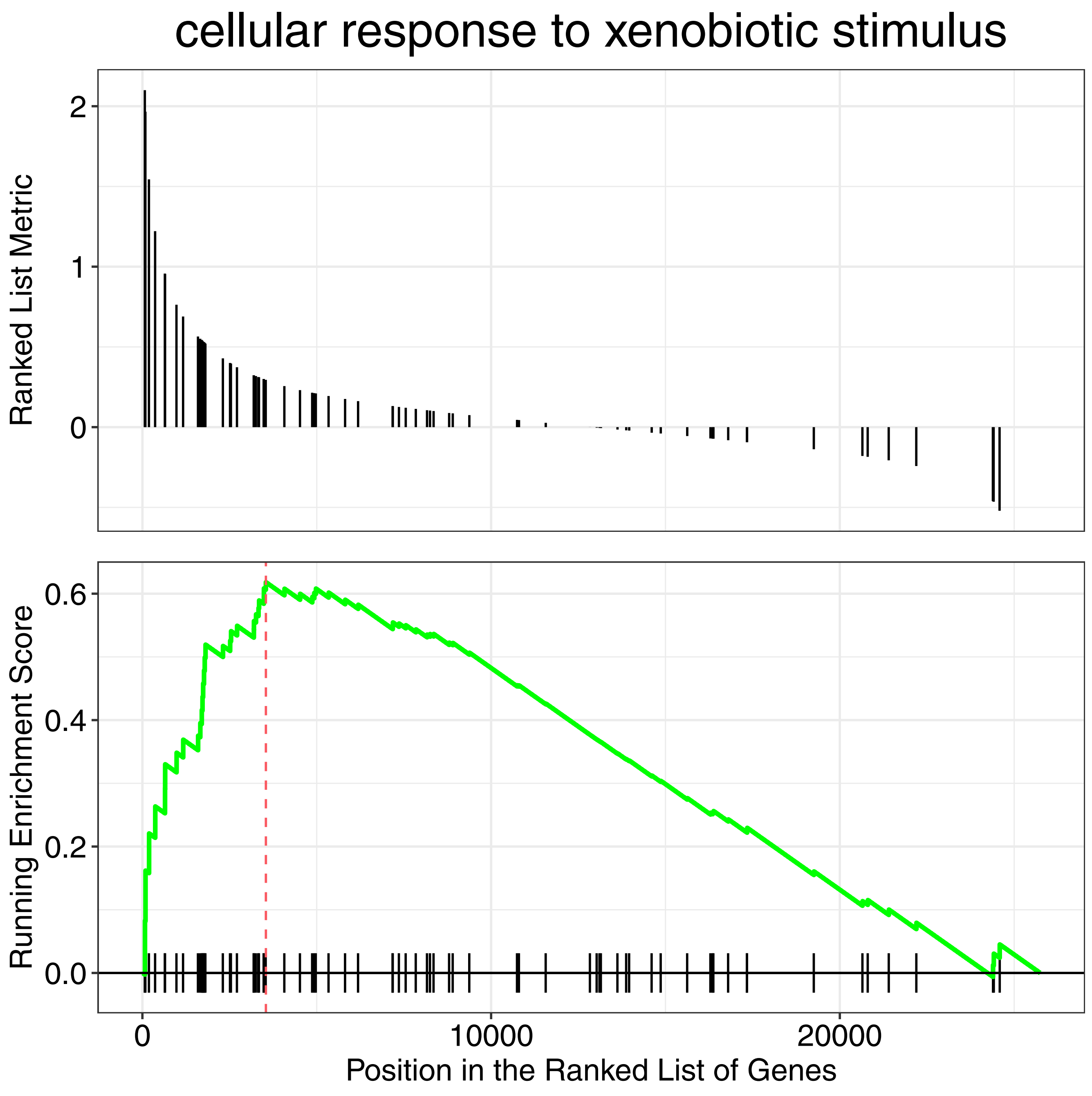

Biology Open • Supplementary information

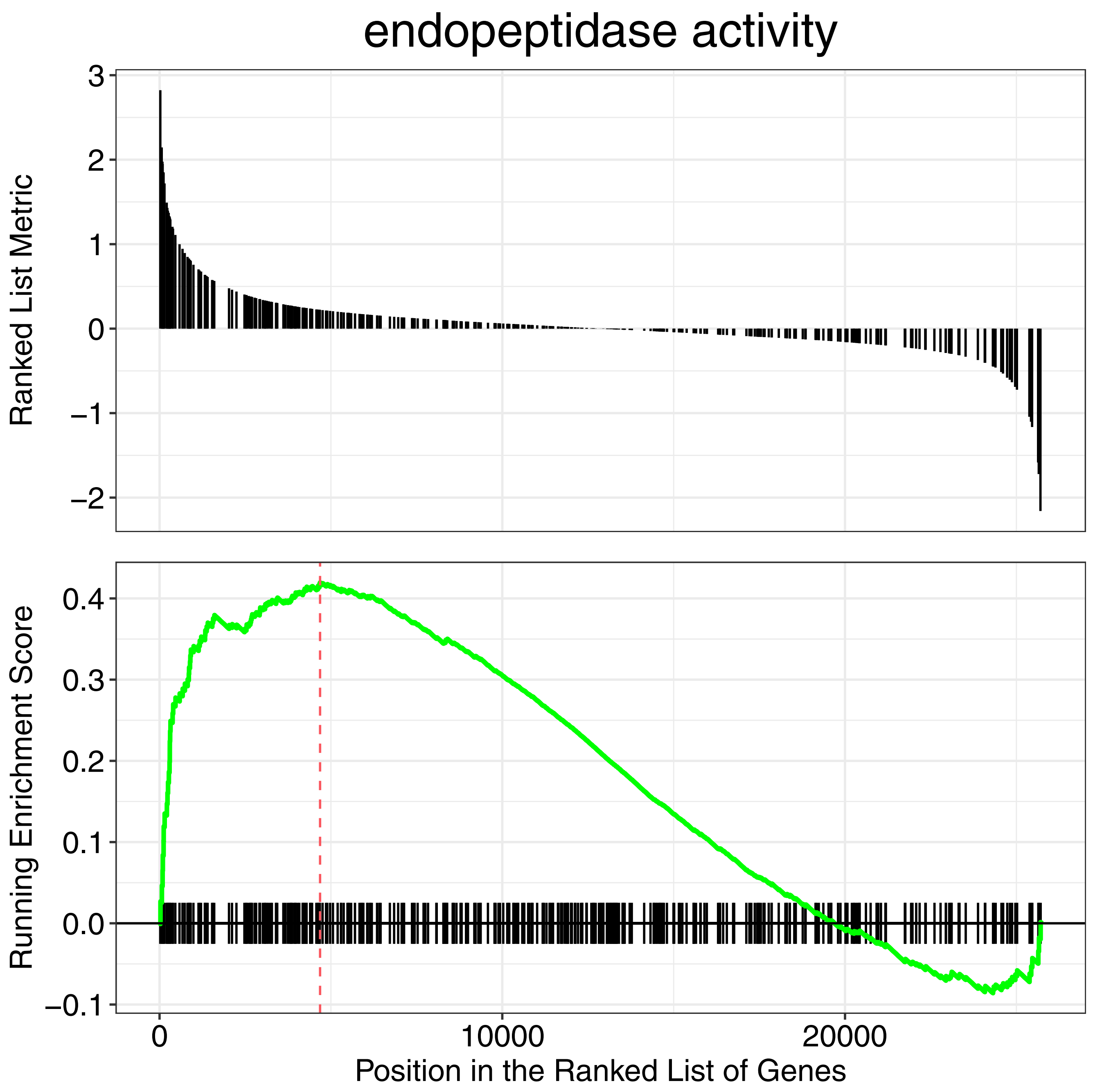

Biology Open • Supplementary information

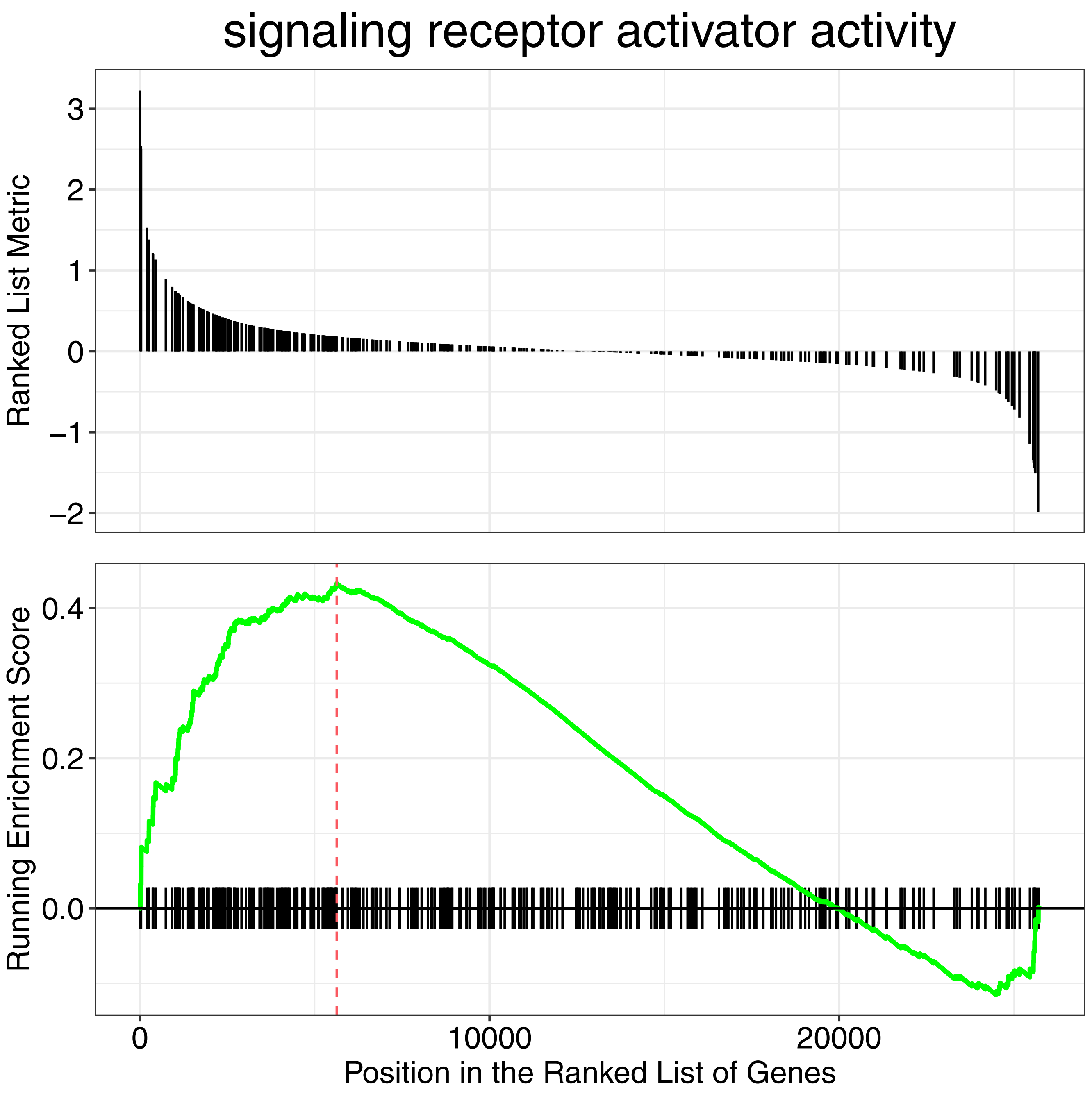
signaling receptor activator activity
Ranked List Metric
3
2
1
0
−1
−2
Running Enrichment Score
0.4
0.2
0.0
0
10000
20000
Position in the Ranked List of Genes

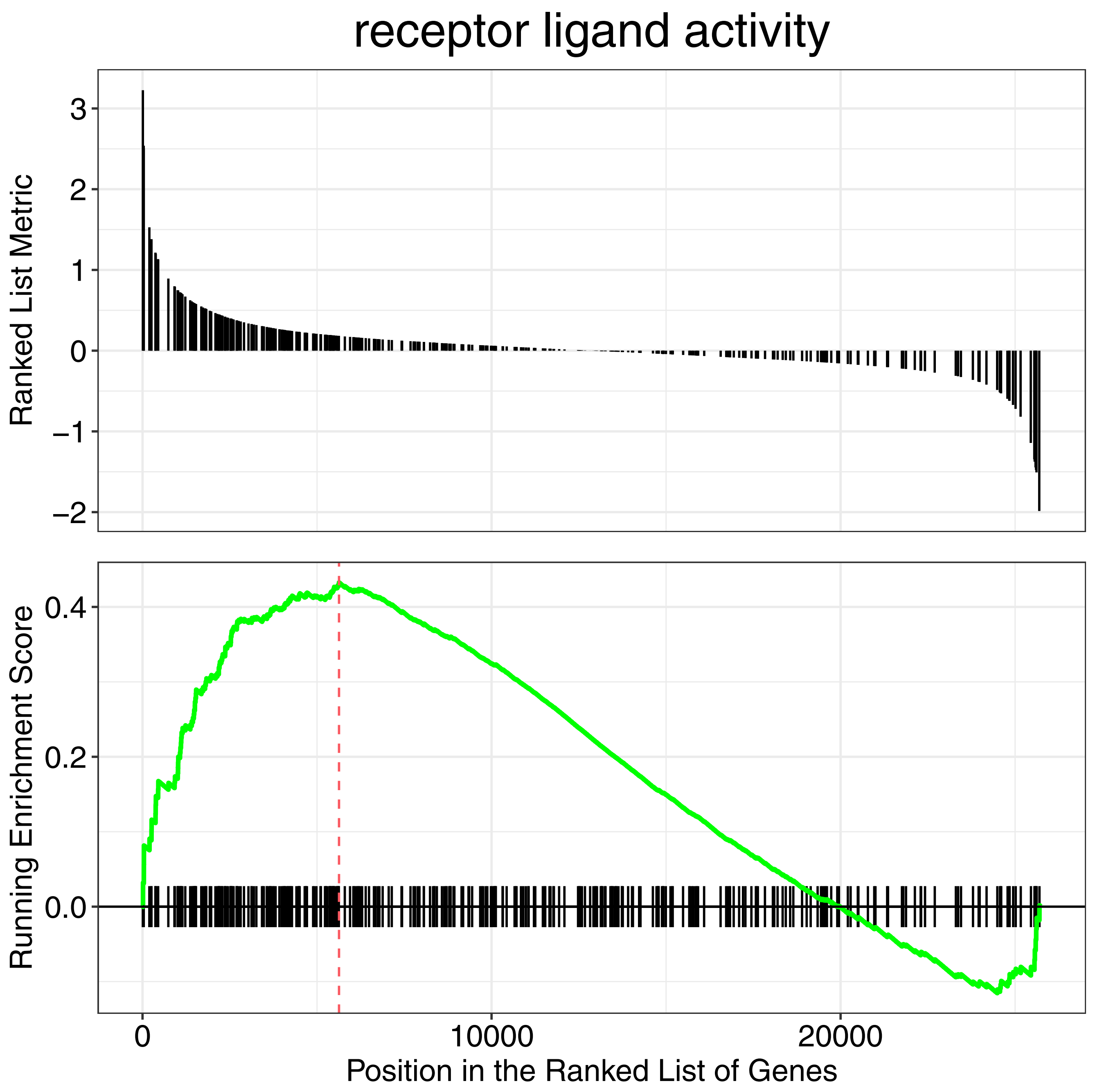

Biology Open • Supplementary information

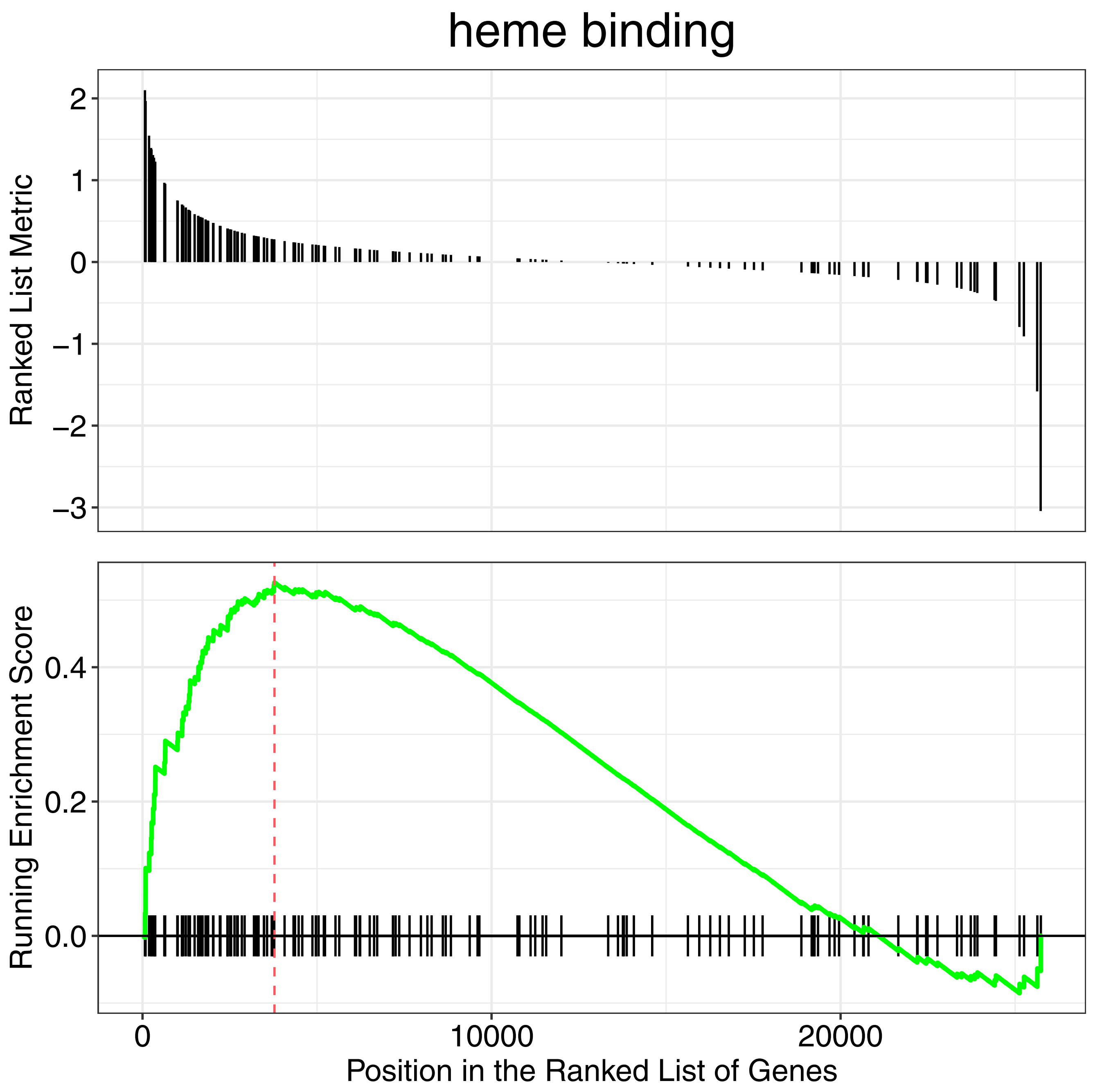

Biology Open • Supplementary information

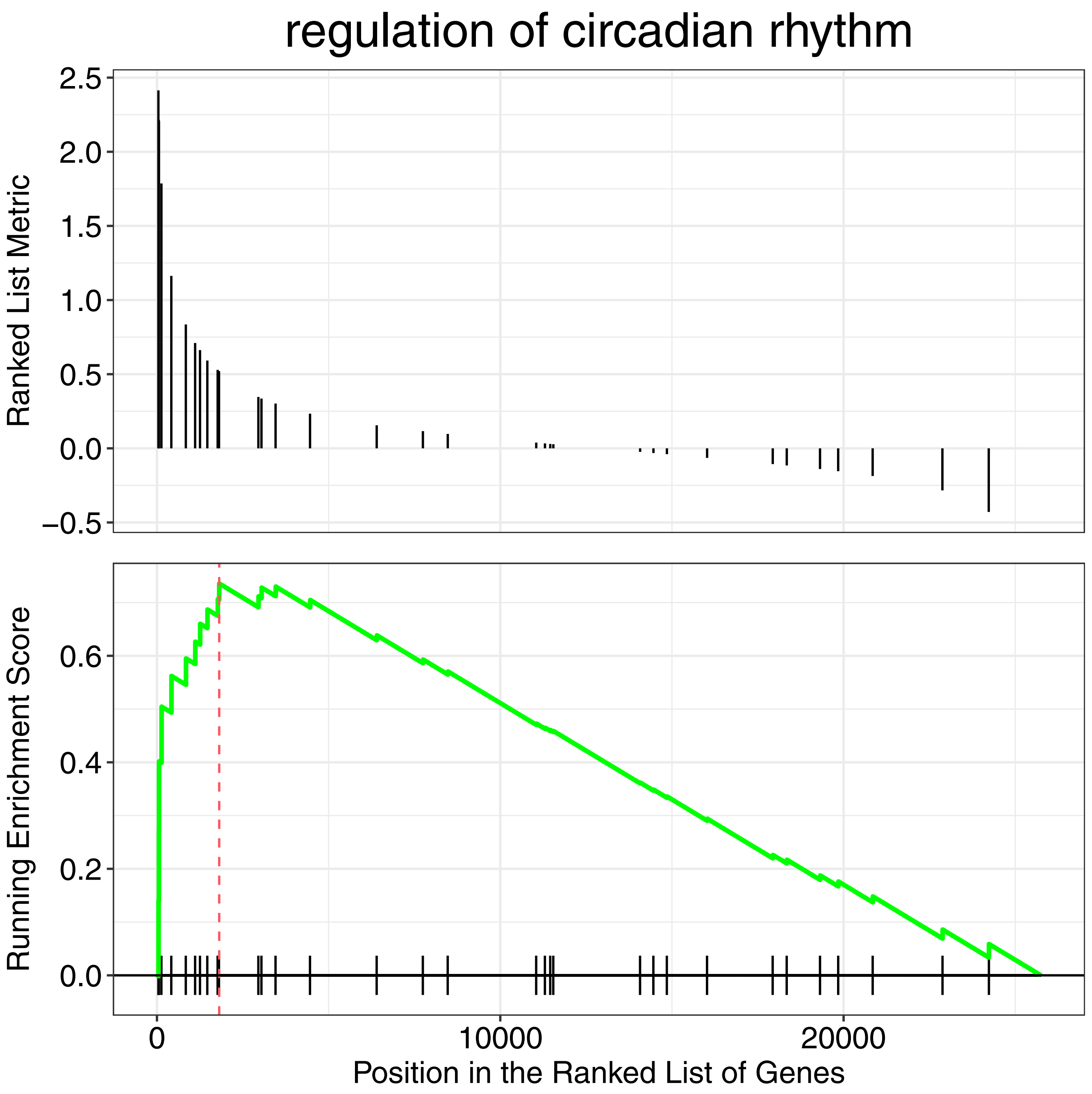

Biology Open • Supplementary information

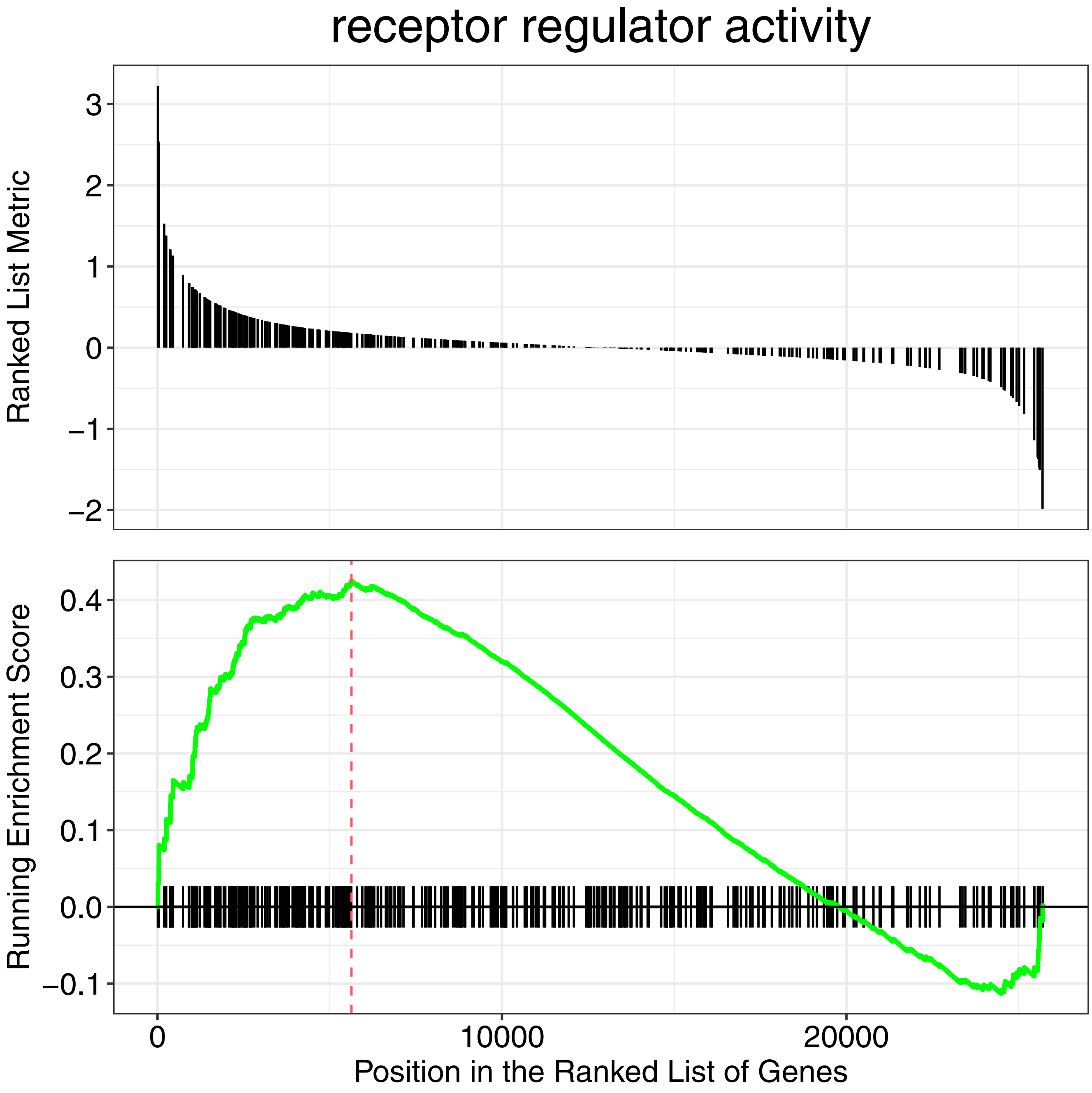

Biology Open • Supplementary information

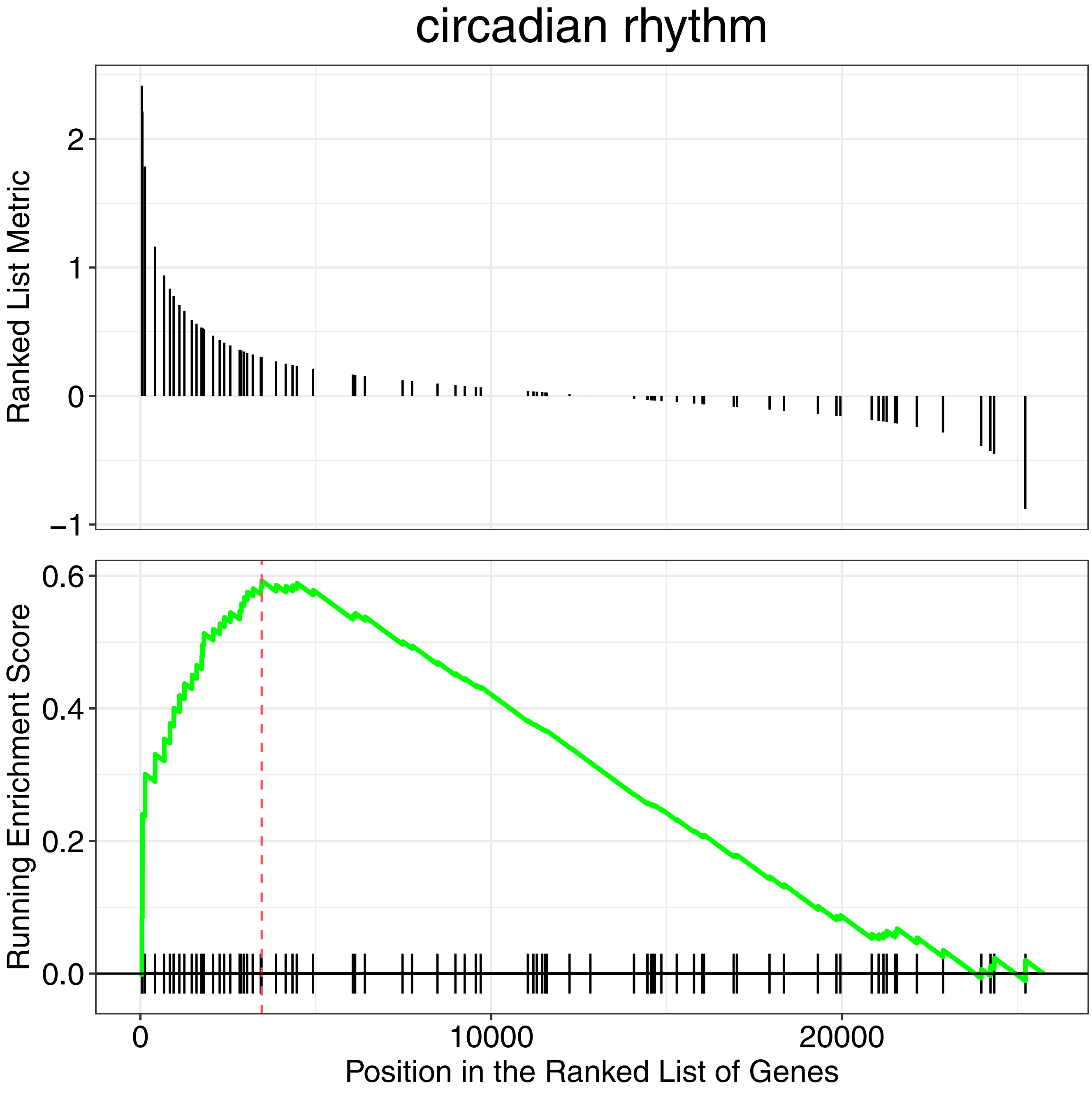

Biology Open • Supplementary information

incorporation or reduction of molecular oxygen, reduced flavin or flavopr[illegible]

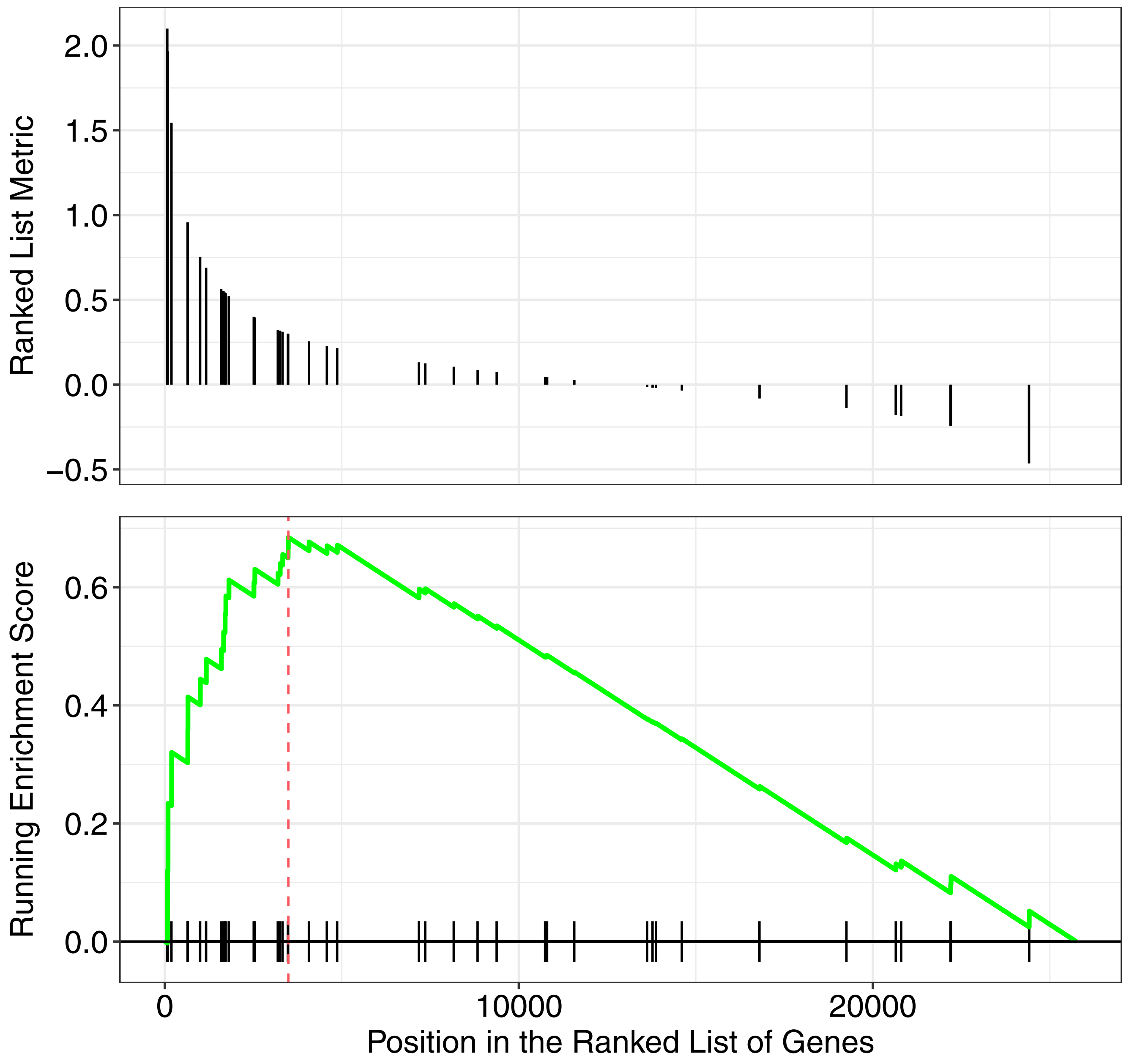

Biology Open • Supplementary information

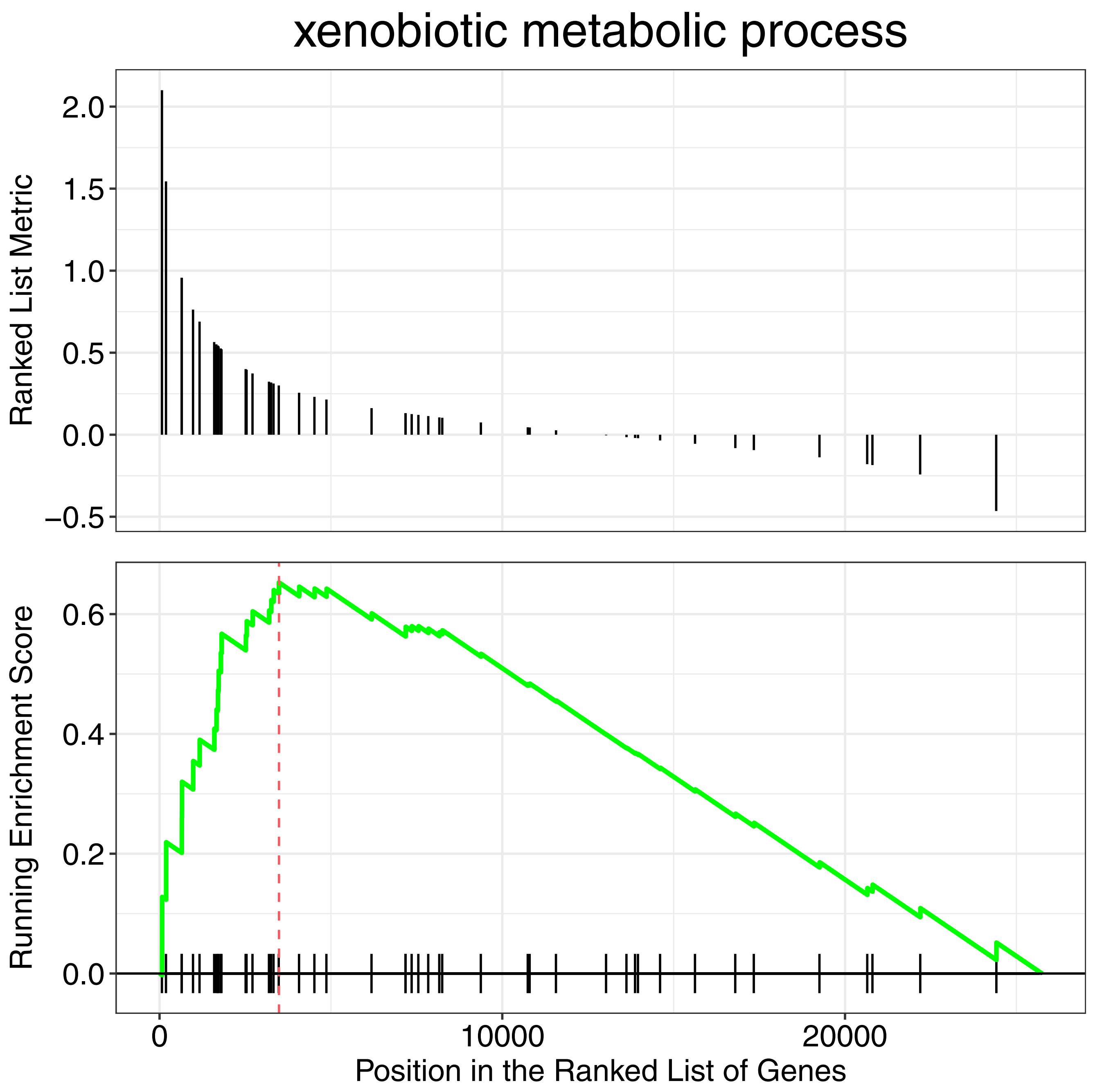

Biology Open • Supplementary information

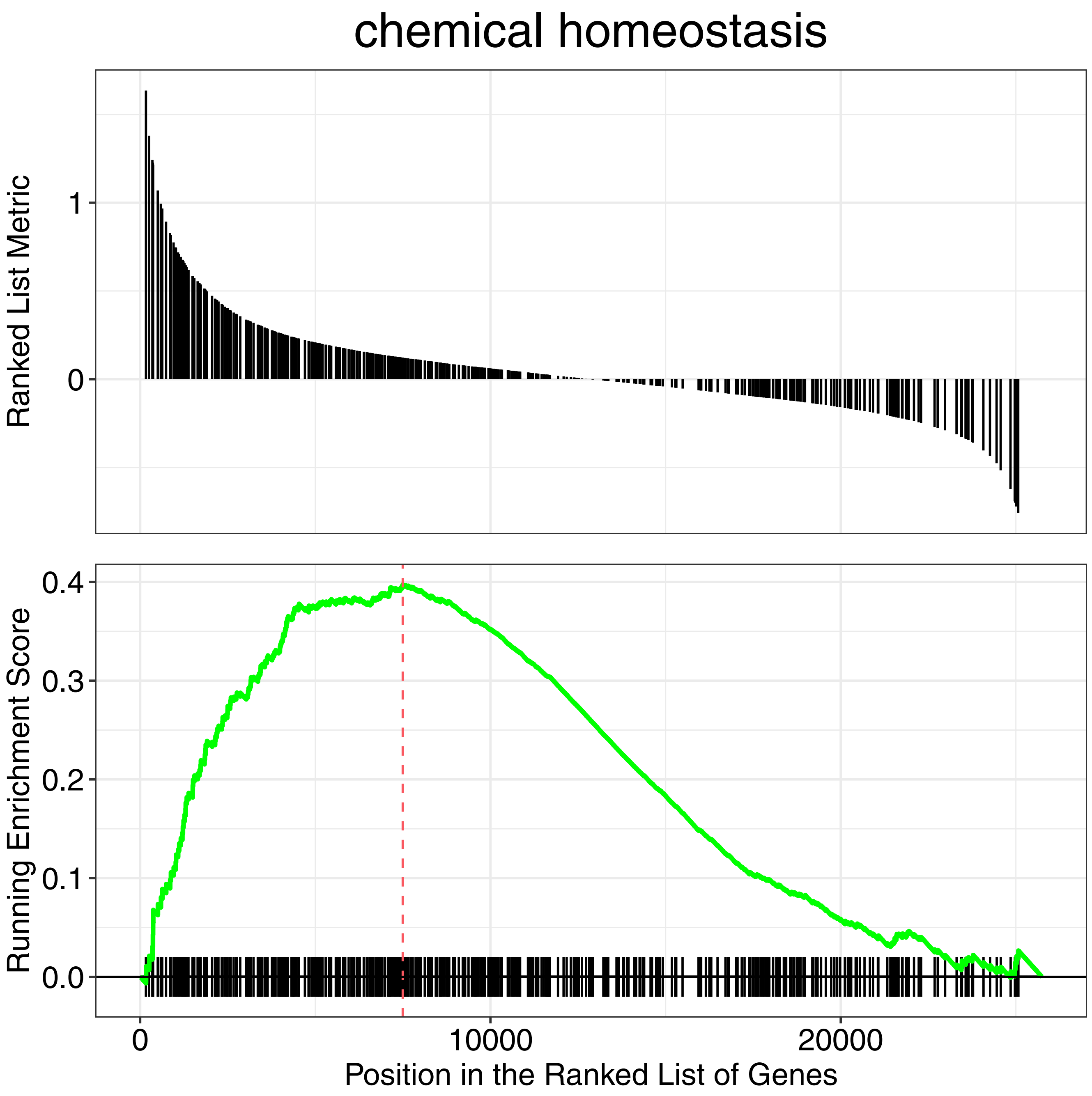

Biology Open • Supplementary information

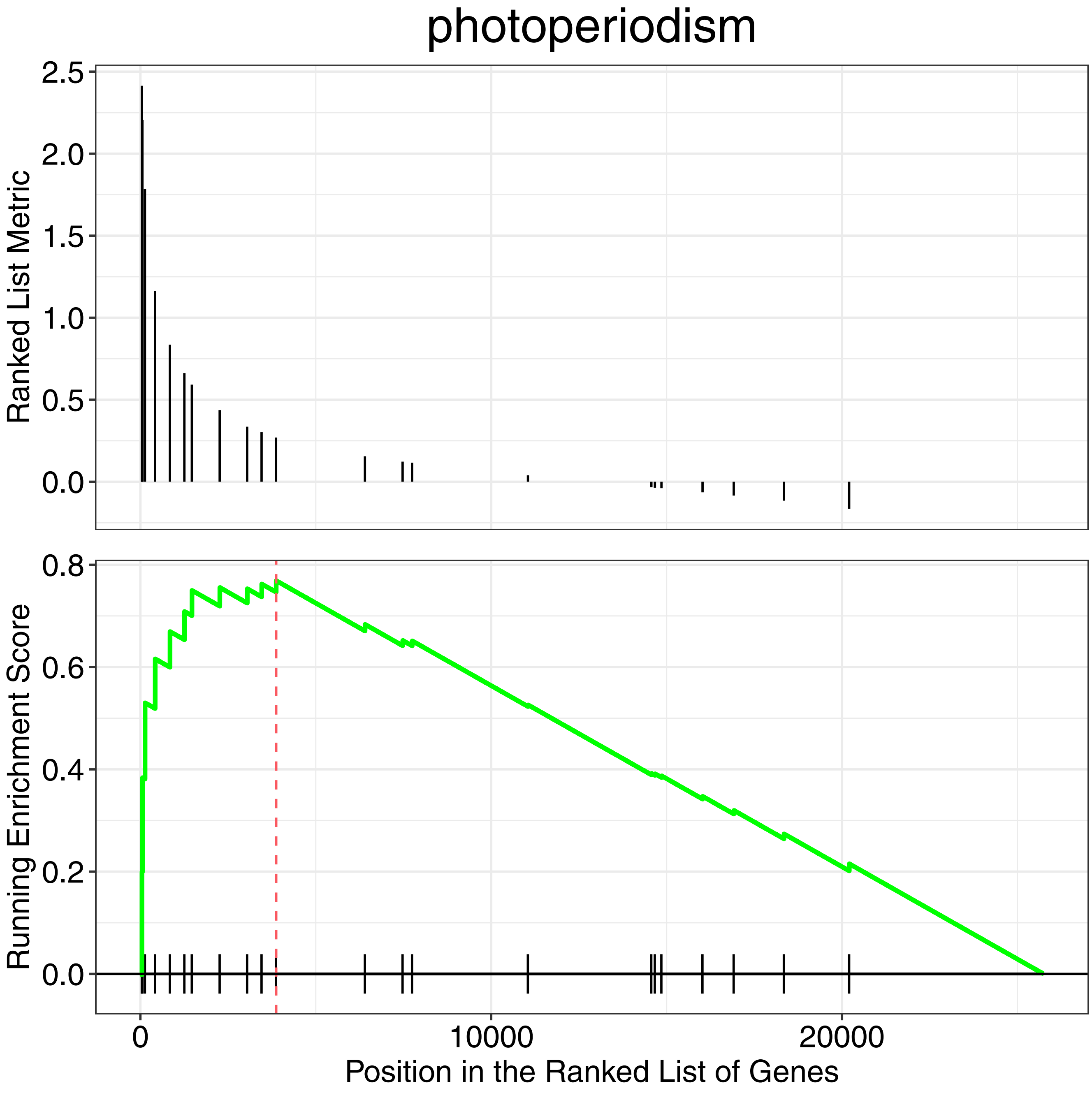

Biology Open • Supplementary information

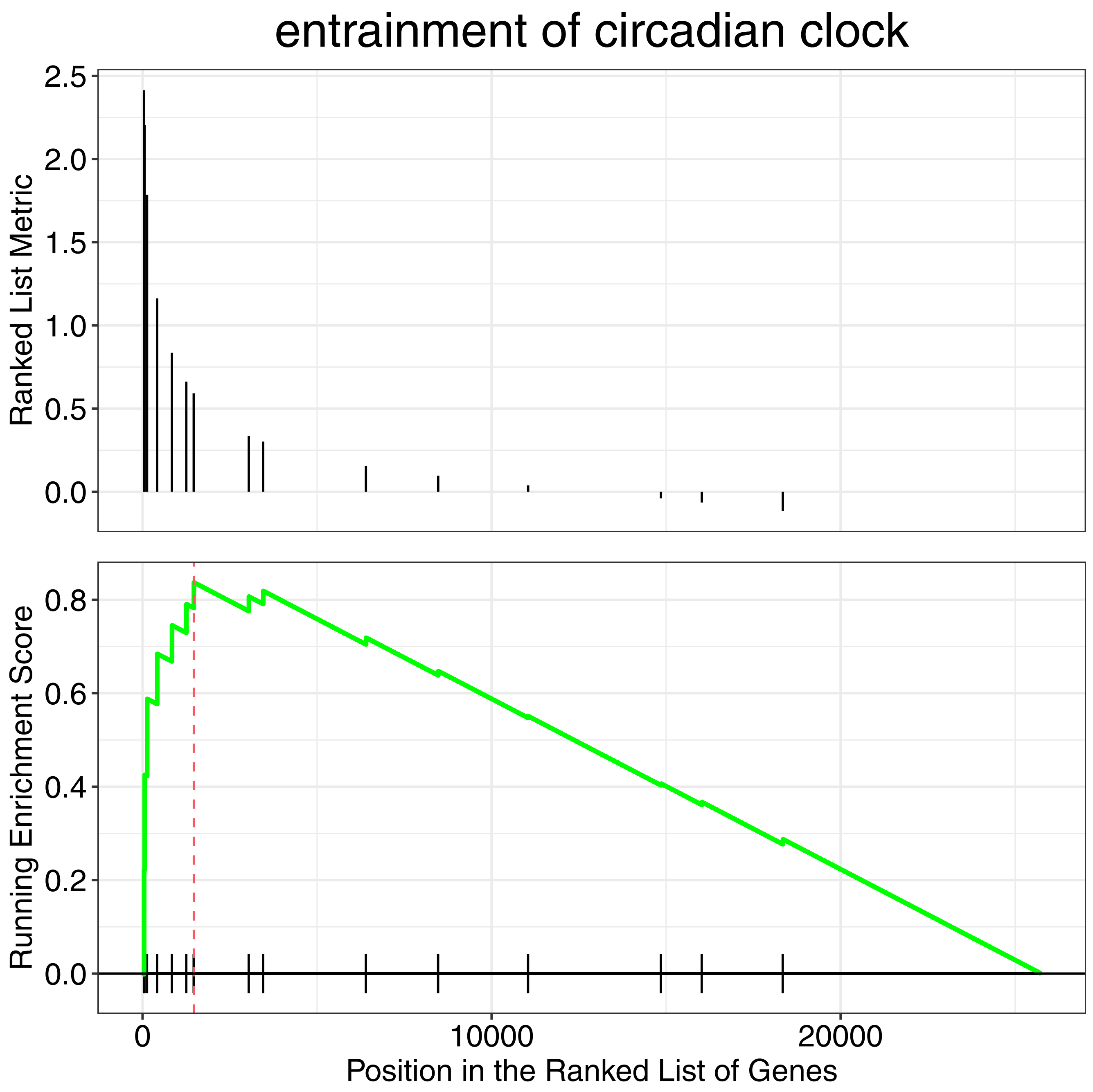

Biology Open • Supplementary information

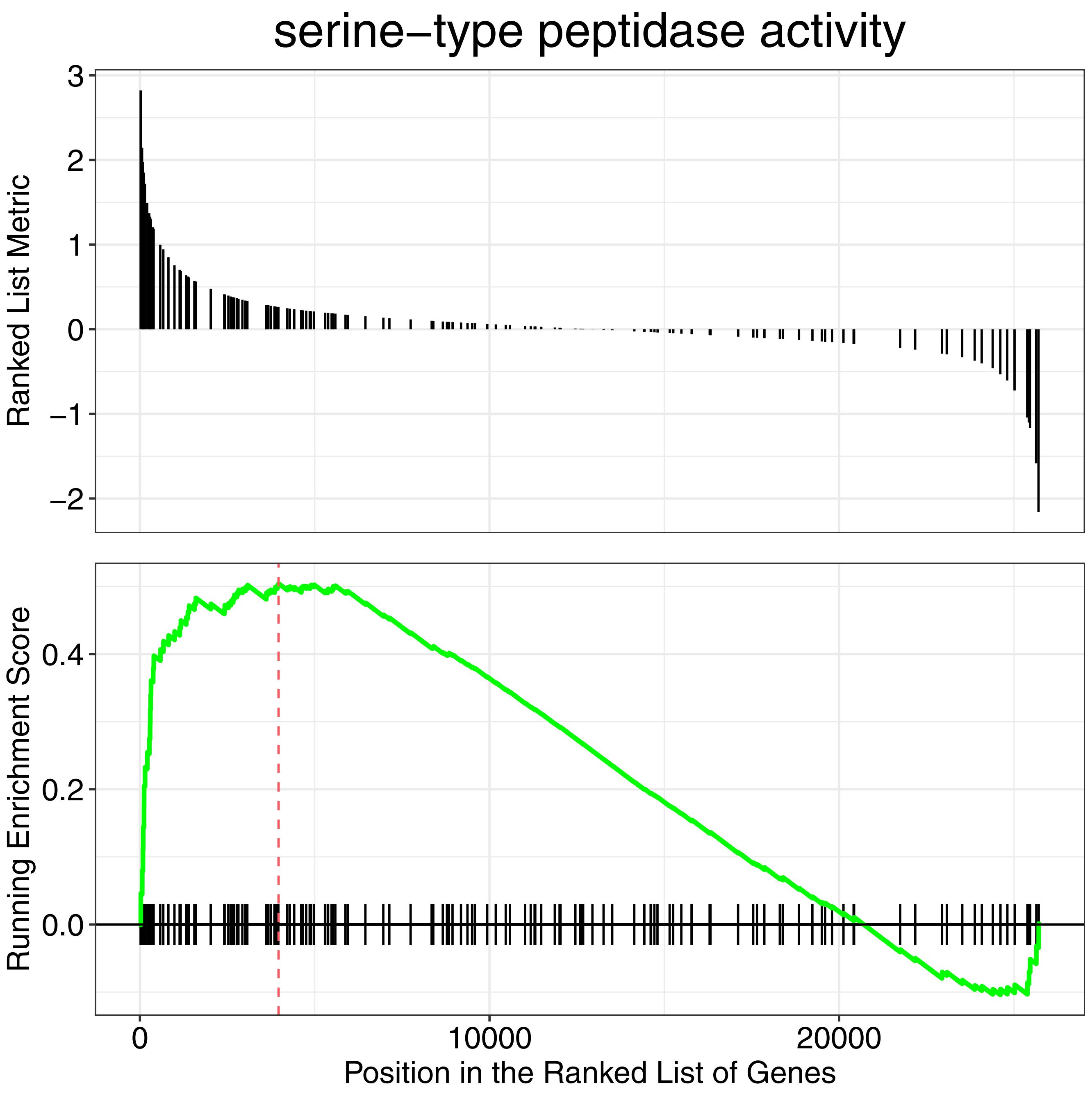

Biology Open • Supplementary information

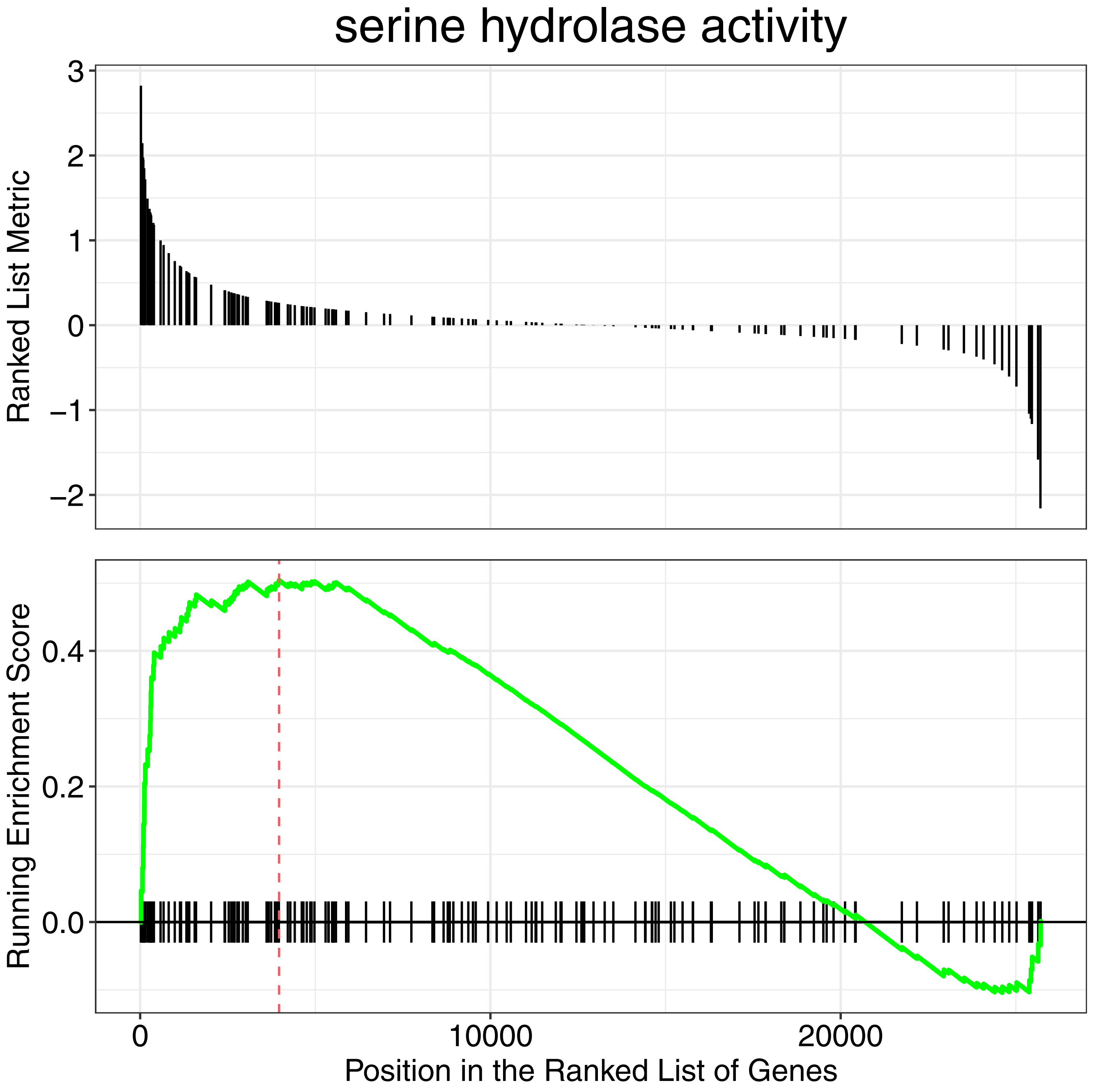

Biology Open • Supplementary information

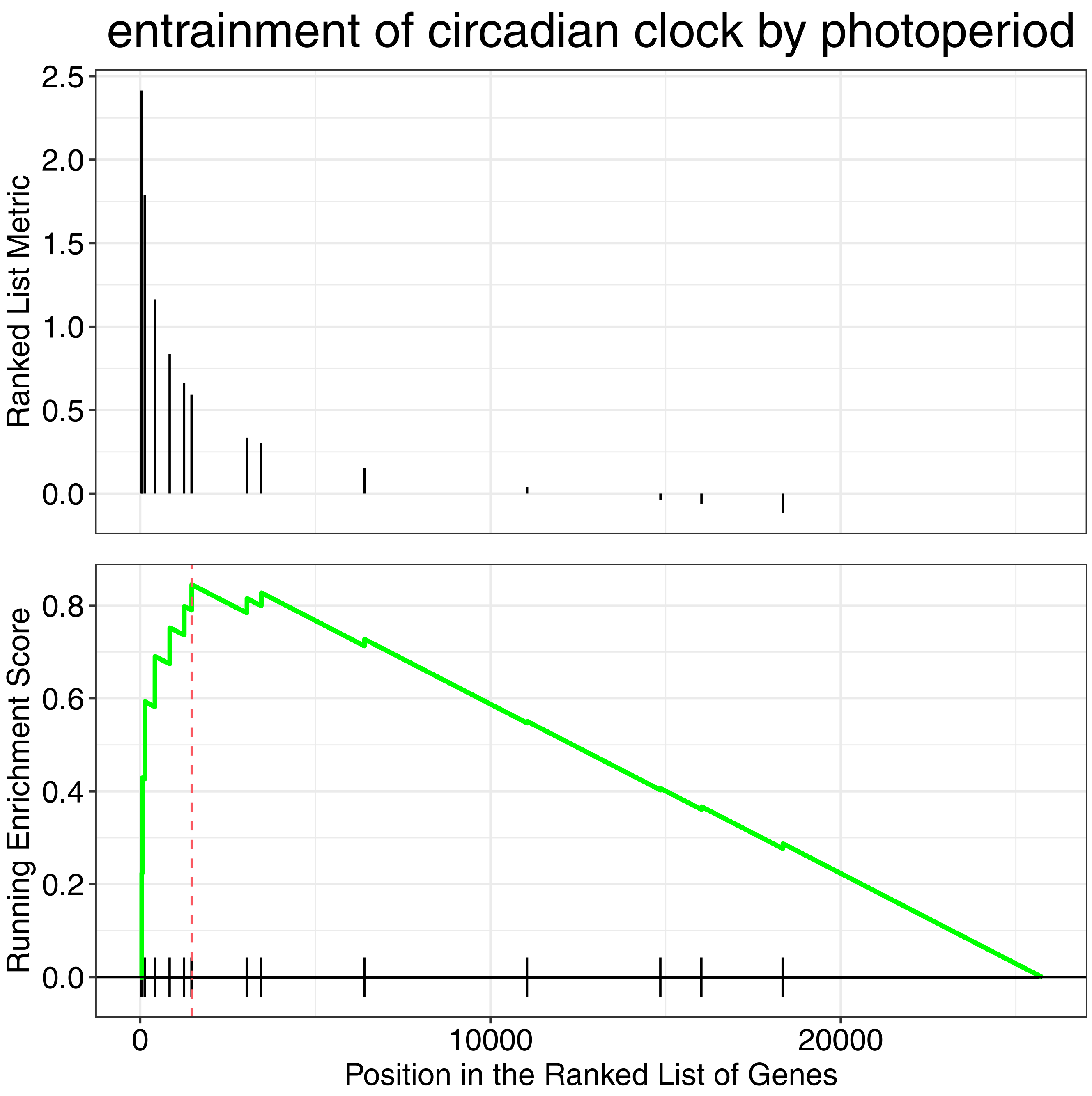

Biology Open • Supplementary information

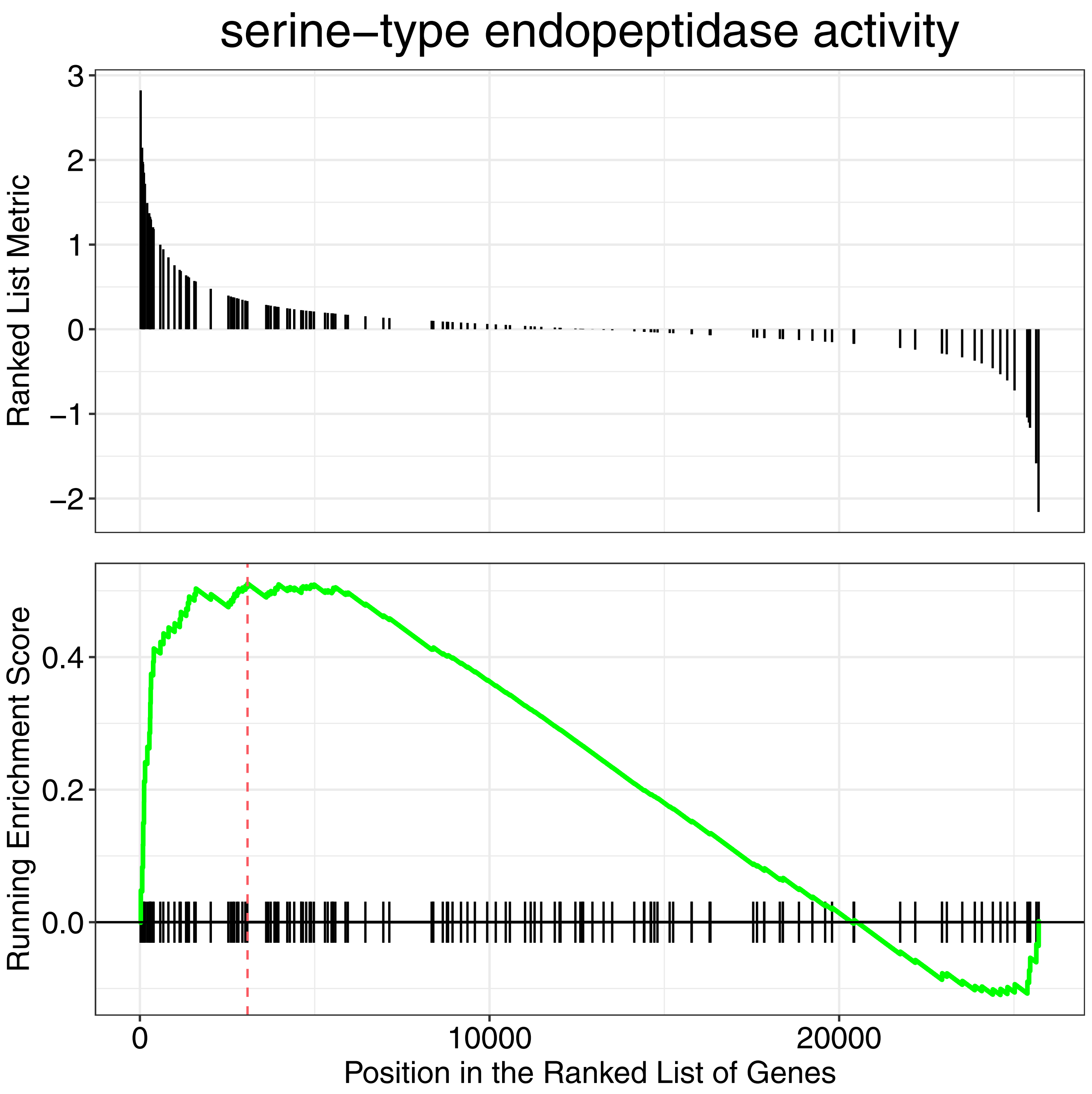

serine−type endopeptidase activity
Ranked List Metric
3
2
1
0
−1
−2
Running Enrichment Score
0.4
0.2
0.0
0
10000
20000
Position in the Ranked List of Genes

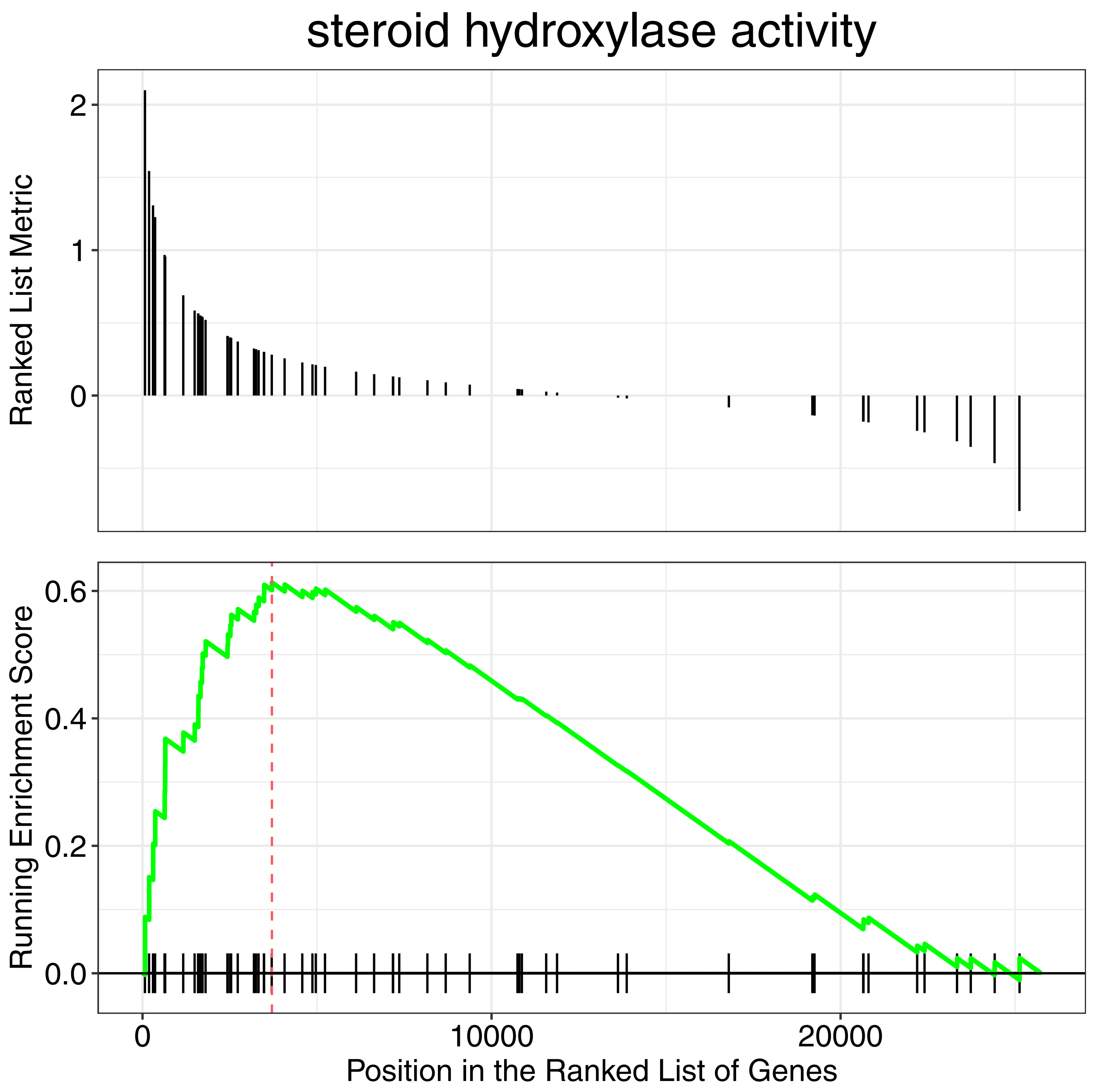

Biology Open • Supplementary information

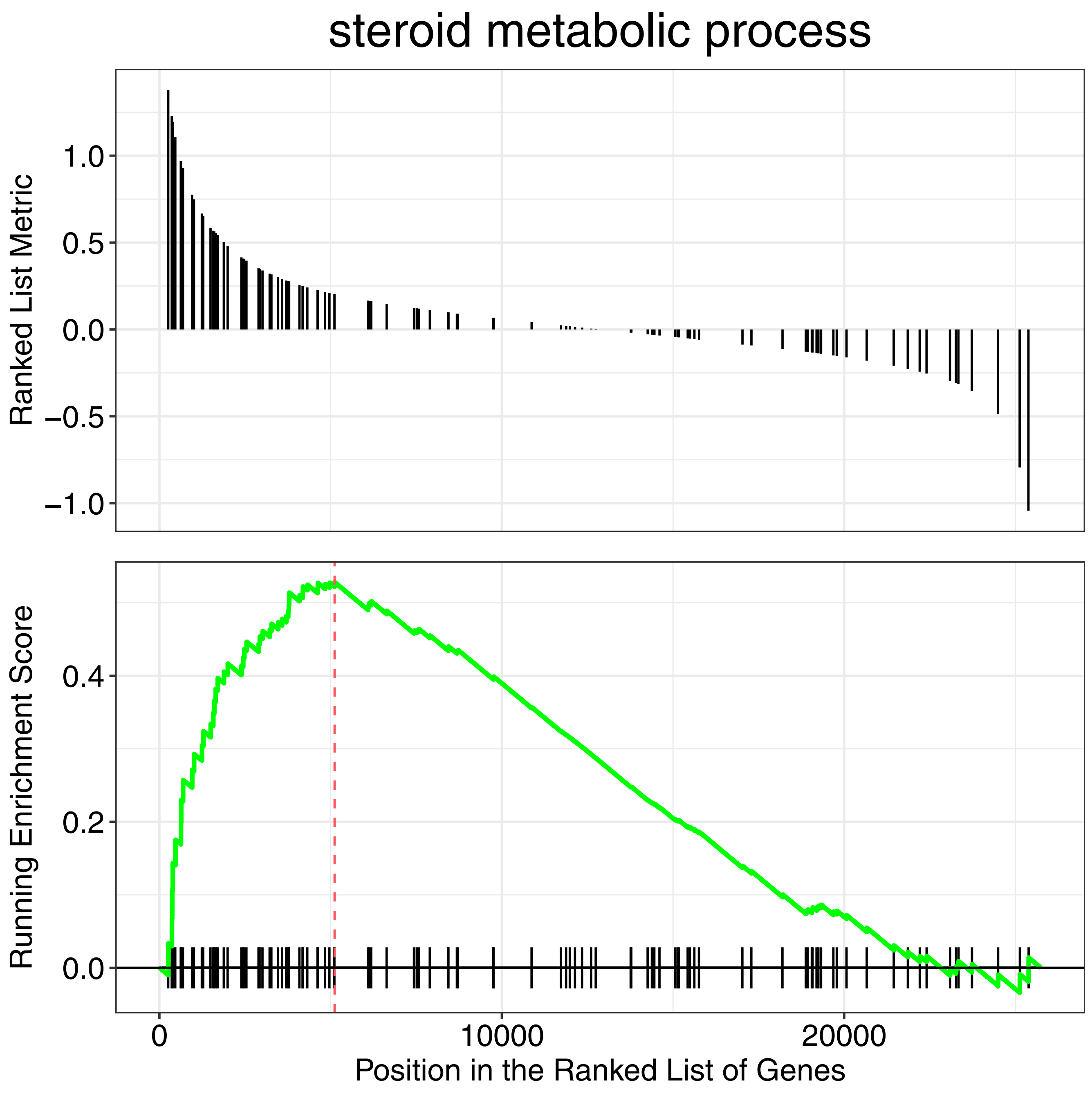

Biology Open • Supplementary information

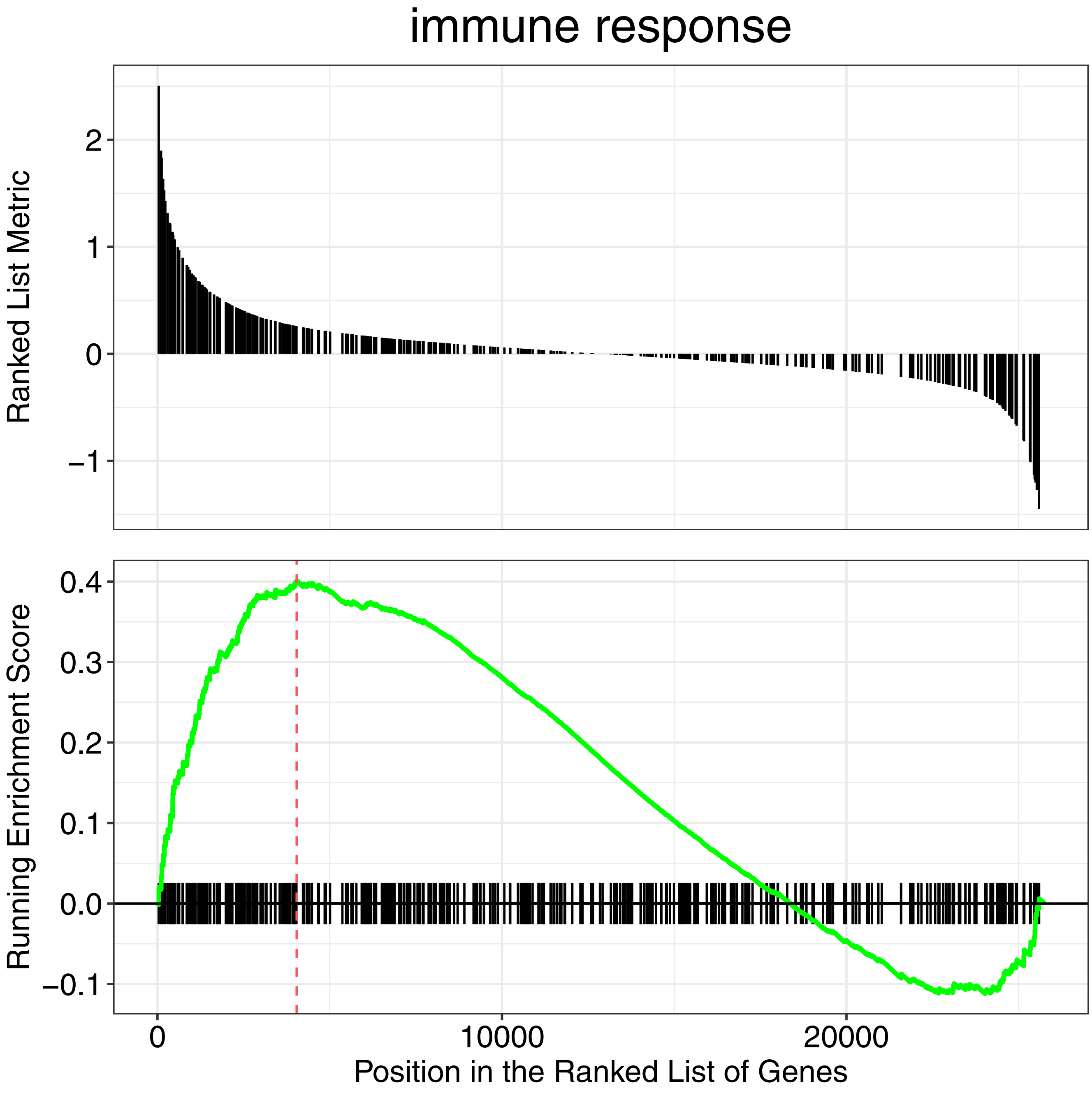

Biology Open • Supplementary information

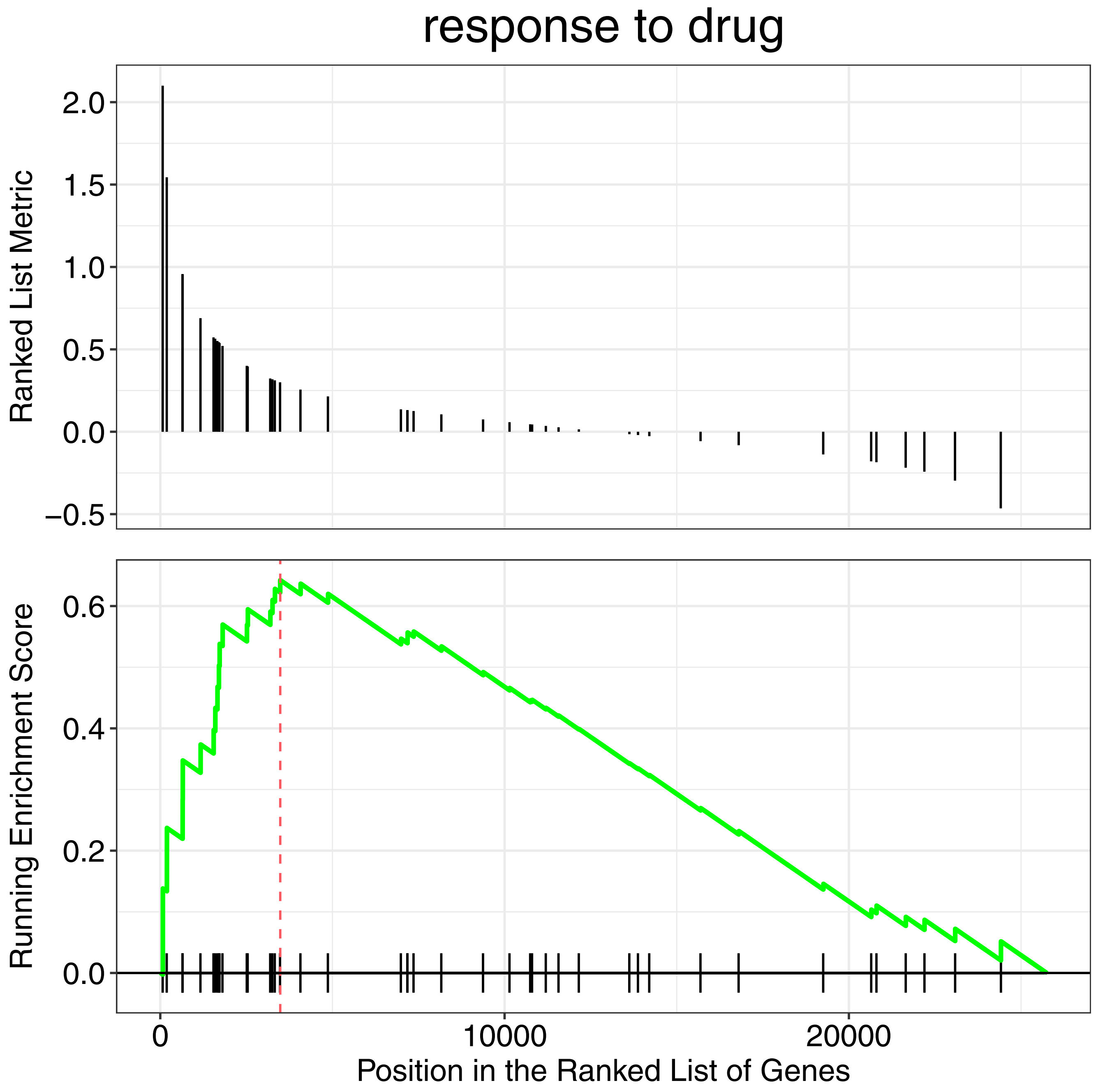

Biology Open • Supplementary information

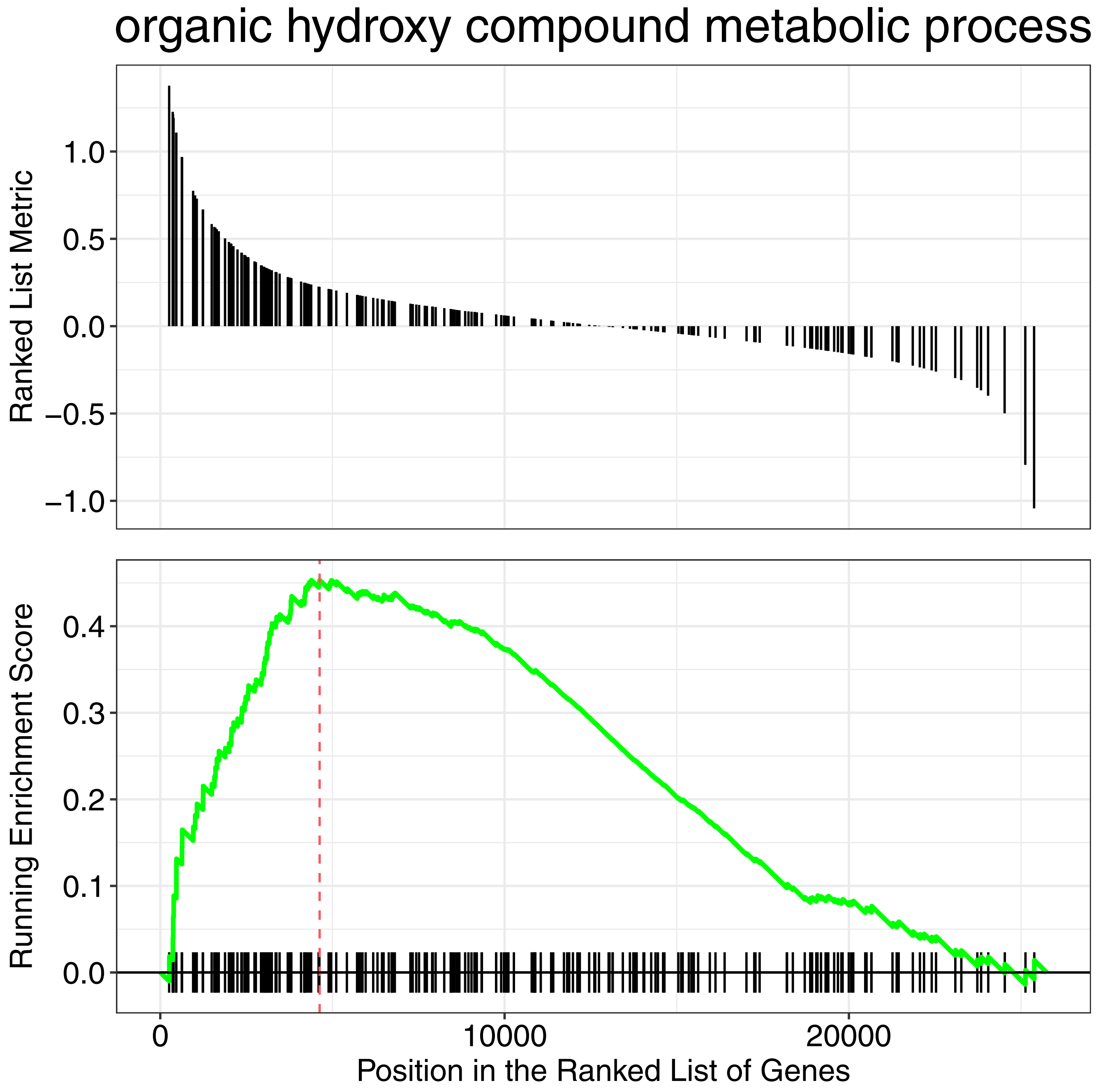

Biology Open • Supplementary information

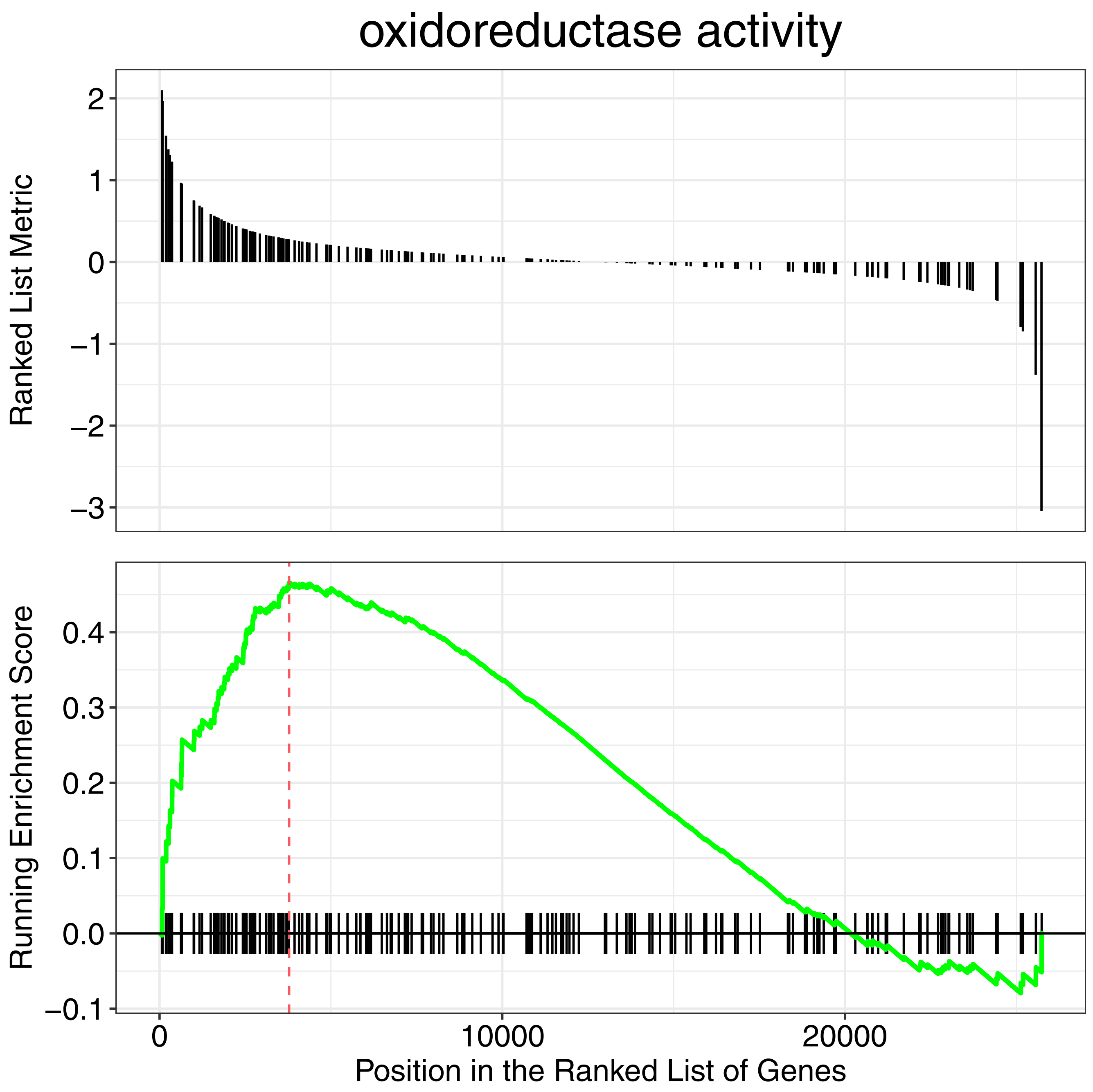

Biology Open • Supplementary information

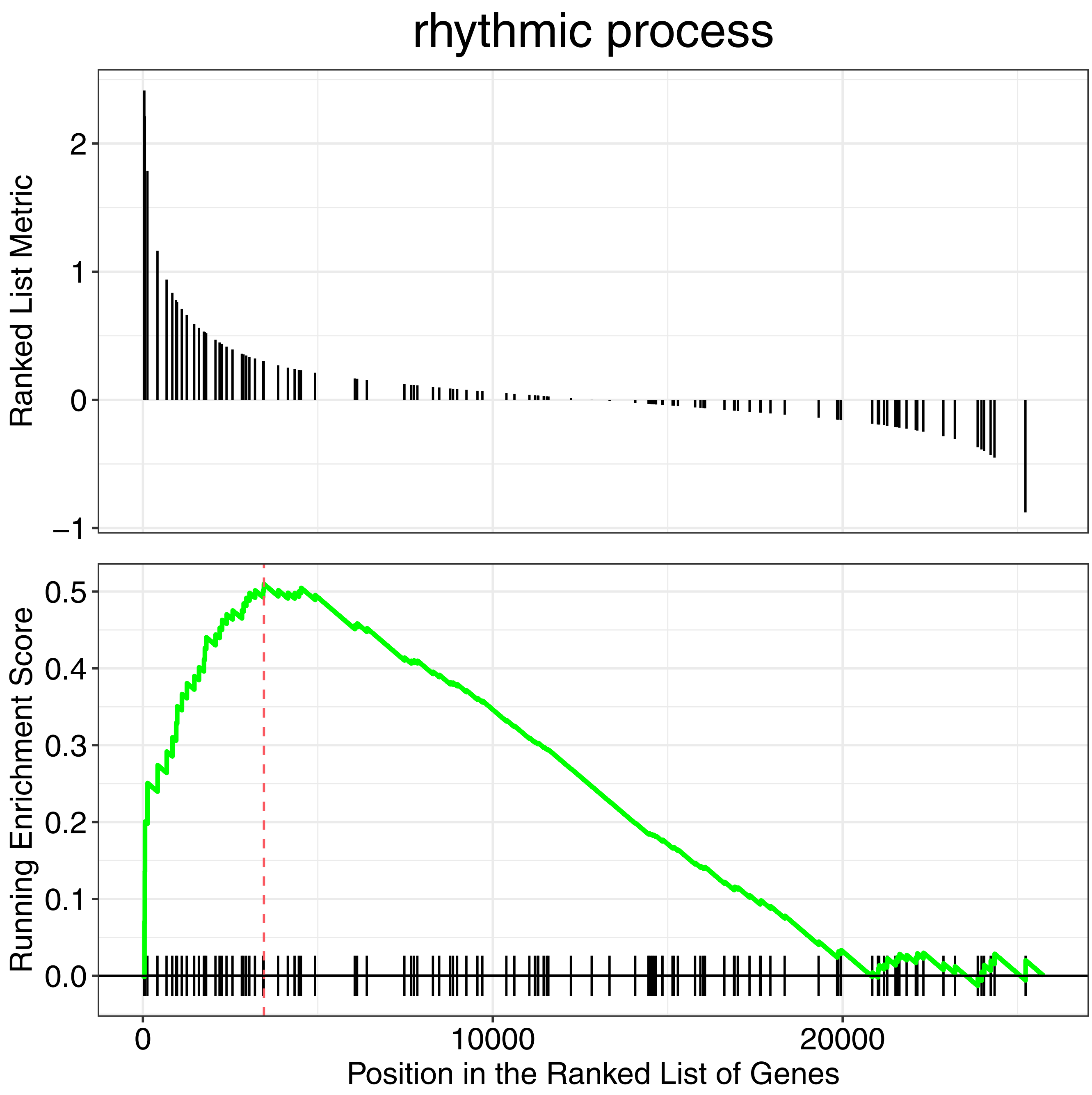

Fig. S8. Enrichment plots of Gene set enrichment analysis (GSEA) of differentially expressed genes in alx1;alx3 crispants. Running score plot and pre-ranked list of all activated GSEA terms.

Biology Open • Supplementary information

Table S1. Differentially expressed genes in alx1;alx3 mutants.

All differentially expressed genes with multiple-testing adjusted p-value smaller than or equal to 0.05 in 5dpf alx1;alx3 mutants when compared to age-matched alx ^{uw2016} siblings.

Click here to download Table S1

Table S2. Gene set enrichment analysis (GSEA) of differentially expressed genes in alx1;alx3 mutants.

All enriched GO terms with multiple-testing adjusted p-value smaller than or equal to 0.05 from all differentially expressed genes in 5dpf alx1;alx3 mutants when compared to age-matched alx ^{uw2016} siblings.

Click here to download Table S2

Table S3. Differentially expressed genes in alx1;alx3 crispants. All differentially expressed genes with multiple-testing adjusted p-value smaller than or equal to 0.05 in 3dpf **alx1;alx3 crispants** when compared to age-matched wildtype embryos.

Click here to download Table S3

Table S4. Gene set enrichment analysis (GSEA) of differentially expressed genes in alx1;alx3 crispants.

All enriched GO terms with multiple-testing adjusted p-value smaller than or equal to 0.05 from all differentially expressed genes in alx1;alx3 crispants when compared to age-matched wildtype.

Click here to download Table S4

Biology Open • Supplementary information
